# Duplication and divergence of the retrovirus restriction gene *Fv1* in *Mus caroli* allows protection from multiple retroviruses

**DOI:** 10.1371/journal.pgen.1008471

**Published:** 2020-06-11

**Authors:** Melvyn W. Yap, George R. Young, Renata Varnaite, Serge Morand, Jonathan P. Stoye

**Affiliations:** 1 The Francis Crick Institute, London, United Kingdom; 2 Centre National de la Recherche Scientifique-Centre de coopération Internationale en Recherche Agronomique pour le Développement Animal et Gestion Intégrée des Risques, Faculty of Veterinary Technology, Kasetsart University, Bangkok, Thailand; 3 Faculty of Medicine, Imperial College London, London, United Kingdom; Fred Hutchinson Cancer Research Center, UNITED STATES

## Abstract

Viruses and their hosts are locked in an evolutionary race where resistance to infection is acquired by the hosts while viruses develop strategies to circumvent these host defenses. Forming one arm of the host defense armory are cell autonomous restriction factors like Fv1. Originally described as protecting laboratory mice from infection by murine leukemia virus (MLV), Fv1s from some wild mice have also been found to restrict non-MLV retroviruses, suggesting an important role in the protection against viruses in nature. We surveyed the *Fv1* genes of wild mice trapped in Thailand and characterized their restriction activities against a panel of retroviruses. An extra copy of the *Fv1* gene, named Fv7, was found on chromosome 6 of three closely related Asian species of mice: *Mus caroli*, *M*. *cervicolor*, and *M*. *cookii*. The presence of flanking repeats suggested it arose by LINE-mediated retroduplication within their most recent common ancestor. A high degree of natural variation was observed in both *Fv1* and *Fv7* and, on top of positive selection at certain residues, insertions and deletions were present that changed the length of the reading frames. These genes exhibited a range of restriction phenotypes, with activities directed against gamma-, spuma-, and lentiviruses. It seems likely, at least in the case of *M*. *caroli*, that the observed gene duplication may expand the breadth of restriction beyond the capacity of Fv1 alone and that one or more such viruses have recently driven or continue to drive the evolution of the *Fv1* and *Fv7* genes.

## Author summary

During the passage of time all vertebrates will be exposed to infection by a variety of different kinds of virus. To meet this threat, a variety of genes for natural resistance to viral infection have evolved. The prototype of such so-called restriction factors is encoded by the mouse *Fv1* gene, which acts to block the life cycle of retroviruses at a stage between virus entry into the cell and integration of the viral genetic material into the nuclear DNA. We have studied the evolution of this gene in the wild mice from South East Asia and describe an example where a duplication of the *Fv1* gene has taken place. The two copies of the gene, initially identical, have evolved separately allowing the development of resistance to two rather different kinds of retroviruses, lenti- and spumaviruses. Independent selection for resistance to these two kinds of retrovirus suggests that such mice are repeatedly exposed to never-before-reported pathogenic retroviruses of these genera.

## Introduction

Retroviruses are obligate parasites that usurp the host machinery for propagation, inserting their genomes within those of their hosts as an integral part of their life cycles. As judged by the presence of fixed examples (endogenous retroviruses), all jawed vertebrates live under threat of infection. In response, the host has developed mechanisms to prevent viral infections [1, 2]. Forming part of the arsenal in the conflict with viruses are restriction factors, which inhibit various stages of the virus life cycle and act in a cell autonomous manner. Some of these, like TRIM5α [3], APOBEC3G [4], and SAMHD1 [5, 6], act at or before reverse transcription, while others, such as tetherin [7] and SERINC5 [8, 9], inhibit viral budding or fusion. In turn, viruses have developed measures to circumvent these blocks. The HIV-1 accessory genes *vif* and *vpu*, for example, specifically target APOBEC3G and tetherin for degradation, respectively [10, 11]. Alternatively, sequence changes in the targets for restriction may allow virus escape.

The prototypic restriction factor, Fv1 (Friend virus susceptibility gene 1), was first described to protect laboratory mice against lethal infection by murine leukemia virus (MLV) [12, 13]. Two alleles, *Fv1*^*n*^ and *Fv1*^*b*^, were originally described that act in a co-dominant fashion in heterozygous animals [14–16]. We have since found that certain Fv1 variants from wild mice can additionally restrict non-MLV retroviruses [17]. For example, an Fv1 from *M*. *caroli* can restrict feline foamy virus (FFV), a spumavirus, and those from *M*. *spretus* and *M*. *macedonicus* were shown to restrict equine infectious anemia virus (EIAV), a lentivirus. Indeed, between the four subgenera of *Mus* (*Mus*, *Coelomys*, *Pyromys*, and *Nannomys*) considerable variation is present in observed restriction profiles [17].

The molecular cloning of *Fv1* revealed it to be a co-opted retroviral *gag* with homology to ERV-L viruses [18, 19] although the remainder of the donor virus has been lost [20]. Such co-options of endogenous retroviruses, whilst not infrequent, most frequently involve products deriving from the *env* gene, thereby operating through receptor blockade [21]. Instead, Fv1 targets the capsid (CA) protein present in the cytoplasm at a stage in retrovirus replication that is post-entry but before nuclear entry [22–25], binding to CA in the context of the hexametric lattice forming the viral core [26] and interfering with events downstream of reverse transcription [25]. The specificity determinants of Fv1 map to the C-terminal domain (CTD) of the protein, indicating that this is the region that interacts with the viral capsid [27]. The N-terminal domain (NTD) of Fv1 contains a coiled coil that is involved in factor multimerization [26]. This apparent means of binding has obvious parallels to Trim5α [28], another CA-binding restriction factor, which forms a super-lattice over the viral core of infecting HIV-1 particles [29–31].

Viruses breaching both adaptive and innate host defenses have the ability to significantly reduce host fitness; viral burdens are, therefore, likely to have exerted substantial evolutionary pressures [32]. Surveys of the variation of host genes influencing susceptibility to viruses provide useful information about the nature of the evolutionary race between viruses and their hosts and can illuminate mechanisms of viral escape. For example, the positive selection of *Trim5*α in primates has occurred for at least 30 million years (my) and has been shaped by the presence of lentiviruses [33–35]. Similarly, we and others have uncovered equivalent forces acting upon *Fv1*, [17, 36–38] revealing a need for continuous or frequently reoccurring waves of retroviral infection for maintenance of the *Fv1* open reading frame (ORF) over its ~45 my lifetime [38].

To better understand the nature of the selective pressures operating on *Fv1*, we have now set out to examine its variability within three species of wild mice from South East Asia: *M*. *caroli*, *M*. *cervicolor*, and *M*. *cookii*. This work has revealed a retroduplication of the *Fv1* gene within this group of species to give *Fv7*. Both genes retain their expression capacity, show extensive variation, and restriction assays reveal alleles with activity against spuma-, lenti-, and gammaretroviruses. The results of these studies suggest that restriction factor duplication may, at least in the case of *M*. *caroli*, allow a broadening of intrinsic immunity to confer simultaneous protection against multiple retroviral genera.

## Results

### Duplication of *Fv1* in South East Asian mice

We have previously reported two Fv1 variants from *M*. *caroli*, differing in length by 8 amino acids [17]. The longer variant (previously termed CAR1) restricted FFV and, to a lesser extent, prototypic foamy virus (PFV), while the shorter variant (CAR2), did not restrict any of the viruses in our panel. Both variants were cloned from CAROLI/EiJ tissue samples purchased from The Jackson Laboratory. This strain has been maintained by closed colony breeding since 1994 and, as the mice were unlikely to be heterozygous, this led us to wonder if there could be two copies of the *Fv1* gene in *M*. *caroli*. This notion was encouraged by a separate report documenting two bands in a Southern hybridization experiment in which genomic DNA from *M*. *caroli* was probed with sequences corresponding to the 5’ end of Fv1 [36].

**Fig 1:**
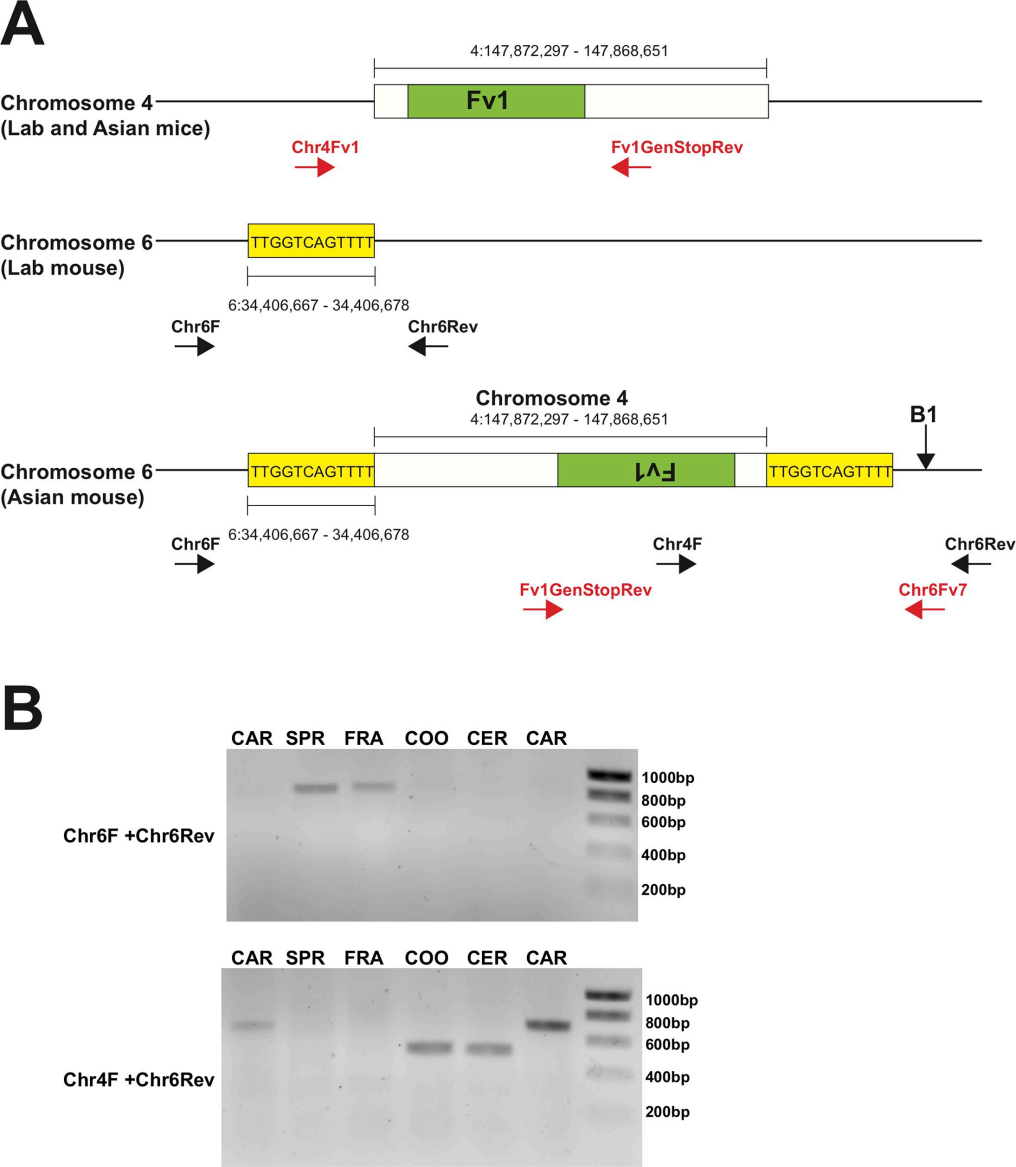
Duplication of *Fv1* in mice from South East Asia. **A**. A schematic representation of the insertion on Chr 6 and the region of Chr 4 duplicated. The direct repeat of the target sequence is highlighted in yellow and an arrow indicates the position of the *M*. *caroli* specific B1 insertion. The positions of primers used for the PCR in Fig 1B are shown in black and those used for cloning are indicated in red. **B**. PCR strategy to confirm the insertion of an *Fv1* CDS on Chr 6 showing products of the primers detailed on the left. From left to right, CAR (CAROLI/EiJ from The Jackson Laboratory), SPR (SPRET/EiJ from The Jackson Laboratory), FRA (*M*. *fragilicauda* R7254), COO (*M*. *cookii* R7121), CER (*M*. *cervicolor* R6223), CAR (*M*. *caroli* R6321).

To investigate this possibility, we initially made use of archived whole genome sequencing data made available under the Wellcome Sanger Institute’s Mouse Genomes Project, which includes CAROLI/EiJ [39]. Alignment of reads from the CAROLI/EiJ dataset to the C57BL/6J reference genome (GRCm38) revealed a doubling in the number of reads corresponding to *Fv1* compared to a C57BL/6NJ control, which stretched both 5’ and 3’ of the *Fv1* locus. Split-read and broken-pair data provided evidence of a second locus on Chr6 of CAROLI/EiJ and subsequent publication of the assembled CAROLI/EiJ genome confirmed these conclusions. Inspection of this region revealed the duplication corresponded to GRCm38 4:147868651–147872297 (3647 nts, extending 329 nts 5’ of the *Fv1* CDS and 1939 bp 3’ of the stop codon) (Fig 1A) and resulted in a new CDS corresponding to 6:29191993–29193375 of the *M*. *caroli* assembly (GenBank GCA_900094665.2). The insertion was flanked by a 12 nt tandem site duplication (TSD) (Fig 1A), suggesting that the duplication occurred through long interspersed nuclear element (LINE)-mediated retrotransposition of an *Fv1* mRNA. Supporting this possibility, the duplicated region 3’ of the *Fv1* CDS and immediately preceding the TSD was terminated by a region of low complexity that did not share homology with the corresponding area of Chr 4. This region was dominated by p(A) stretches, likely evidence of the polyadenylation of the *Fv1* mRNA reverse transcribed by the LINE machinery.

*M*. *caroli* is one of three closely related species, alongside *M*. *cervicolor* and *M*. *cookii*, that constitute an Asian clade of the *Mus Mus* subgenus, estimated to have had a most recent common ancestor (MRCA) around 4 million years ago (mya) [40]. To determine if the duplication of *Fv1* within inbred CAROLI/EiJ was also found within wild populations and to investigate its presence in the sister taxa, we designed a typing PCR for the novel integration (Fig 1A). PCR was performed using DNAs from wild-caught *M*. *cookii*, *M*. *cervicolor*, and *M*. *caroli* trapped in Thailand and, for comparison, with DNAs from wild-caught *M*. *fragilicauda* and with *M*. *caroli* and *M*. *spretus* samples sourced from The Jackson Laboratory.

Primers Chr6F and Chr6Rev anneal to the regions on Chr 6 flanking the novel insertion and, in the absence of the insertion, would yield a 900 bp PCR product (Fig 1A). If the insertion were present, however, its 3.6 kb length would prevent a PCR product from being formed when employing a short extension time. Fragments of the predicted size for a ‘wild-type’ chromosomal region were observed for the reactions with Chr6F/Chr6Rev using DNAs from *M*. *spretus* and *M*. *fragilicauda* (Fig 1B, top) and Sanger sequencing was conducted to verify that the correct chromosomal region had been amplified. This confirmed the absence of an insertion within these species. Conversely, no PCR product was observed using this primer set for *M*. *caroli*, *M*. *cookii*, or *M*. *cervicolor*, consistent with an insertion between the sequences where the primers anneal on Chr 6. A second primer pair, Chr4F/Chr6Rev, with one primer (Chr4F) annealing within the duplicated region of the *Fv1* locus was designed so that a fragment of 500 bp would be produced in the presence of an insert (Fig 1A). Using this primer pair, PCR products were observed with DNAs from *M*. *caroli*, *M*. *cookii*, and *M*. *cervicolor*, but not for *M*. *spretus* and *M*. *fragilicauda* (Fig 1B, bottom). The *M*. *caroli* samples yielded products around 200 bp larger than those of *M*. *cookii* and *M*. *cervicolor*, which, upon sequencing, was found to be due to the presence of a B1 short interspersed nuclear element (SINE) insertion upstream of the gene body.

These results showed that a region of Chr 4 containing *Fv1* had been retroduplicated onto Chr 6 within *M*. *caroli*, *M*. *cookii*, and *M*. *cervicolor* and, hence, that the gene duplication predated the divergence of these species rather than having occurred during inbreeding of the CAROLI/EiJ stocks. The new locus was termed *Fv7* following discussion with The Jackson Laboratory and in accordance with naming conventions. The two previously studied variants from *M*. *caroli*, CAR1 and CAR2 [17], could be assigned to *Fv1* and *Fv7*, respectively. By contrast, the *Fv1* gene previously isolated from *M*. *cervicolor* (CER) [17] was most probably a PCR-derived recombinant between the two genes.

### Genetic variation of *Fv1* and *Fv7* in the wild mouse populations of South East Asia

To test whether this gene duplication might play a role in protection against viruses endemic in South East Asia by allowing development of resistance to additional retroviruses, we set out to determine (a) the extent of sequence change in the novel gene, (b) whether it is transcribed, (c) whether sequence changes result in alterations of restriction specificity, and (d) whether its presence allows a widening of protection to an extent not possible with *Fv1* alone.

To investigate the extent of natural variation in these genes, *Fv1* and *Fv7* from 44 mice (27 *M*. *cervicolor*, 7 *M*. *caroli*, 7 *M*. *cookii*, and 3 *M*. *fragilicauda*), trapped in a range of locations across Thailand (Table 1), were PCR-amplified and cloned using primers specific to the individual loci. Eight clones from each amplification were then sequenced and we identified 7 new *Fv1* and 9 *Fv7* alleles from the *M*. *caroli* samples, 23 *Fv1* and 34 *Fv7* alleles within *M*. *cervicolor*, and 7 *Fv1* and 8 *Fv7* alleles within *M*. *cookii*. There were 4 *Fv1* alleles among the 3 *M*. *fragilicauda* samples. The variants were designated with gene name followed by a three letter species code (CAR, CER, COO, or FRA) followed by a numeric identifier. For example, Fv1COO6 refers to the sixth unique isolate of *Fv1* from *M*. *cookii*. Our previously described CAR1 and CAR2 [17] became Fv1CAR1 and Fv7CAR1, respectively.

**Table 1:** Fv1 and Fv7 variants present in mice from Thailand.

				Variant	
Sample ID	Species	Source / Location	Tissue	Fv1	Fv7
CAROLI/EiJ	*M*. *caroli*	Jackson Laboratory	Tail	1	1
R6321	*M*. *caroli*	Kalasin	Spleen	2, 3	2
R6657	*M*. *caroli*	Prachuapkirikhan	Spleen	4	3, 4
R6685	*M*. *caroli*	Prachuapkirikhan	Spleen	4, 5	3, 5
R7195	*M*. *caroli*	Nan	Liver	6, 7	6
R7225	*M*. *caroli*	Nan	Liver	6	7,8
R7236	*M*. *caroli*	Nan	Liver	6, 8	9, 7
R7264	*M*. *caroli*	Nakhon Ratchasima	Liver	5	10
R6223	*M*. *cervicolor*	Kalasin	Spleen	1, 2	1, (2)
R6243	*M*. *cervicolor*	Kalasin	Spleen	3*, 4	3, 4
R6244	*M*. *cervicolor*	Kalasin	Spleen	5	3, 5
R6254	*M*. *cervicolor*	Kalasin	Spleen	2	6, 7
R6255	*M*. *cervicolor*	Kalasin	Spleen	3*, 6	(8)
R6257	*M*. *cervicolor*	Kalasin	Spleen	1, 7	9, 10
R6262	*M*. *cervicolor*	Kalasin	Spleen	2, 8	3, 11
R6264	*M*. *cervicolor*	Kalasin	Spleen	4, 9	12, 13
R6278	*M*. *cervicolor*	Kalasin	Spleen	3*, (10*)	14, 15
R6279	*M*. *cervicolor*	Kalasin	Spleen	3*	14, 15
R6280 +	*M*. *cervicolor*	Kalasin	Spleen	2	16, 17
R6281	*M*. *cervicolor*	Kalasin	Spleen	11, 12	(18), 19
R6288 +	*M*. *cervicolor*	Kalasin	Spleen	3*, 13	20
R6293	*M*. *cervicolor*	Kalasin	Spleen	2	13, 21**
R6294	*M*. *cervicolor*	Kalasin	Spleen	14, 15	22, 23
R6295	*M*. *cervicolor*	Kalasin	Spleen	4	17, 24
R6297 +	*M*. *cervicolor*	Kalasin	Spleen	1, 16	4, 21**
R6298	*M*. *cervicolor*	Kalasin	Spleen	13, 17	23
R6320	*M*. *cervicolor*	Kalasin	Spleen	1, 16	10, 25
R6682	*M*. *cervicolor*	Prachuapkirikhan	Spleen	18	26^§^
R6683	*M*. *cervicolor*	Prachuapkirikhan	Spleen	19	26^§^, 27
R6684 +	*M*. *cervicolor*	Prachuapkirikhan	Spleen	2	26^§^
R6701	*M*. *cervicolor*	Prachuapkirikhan	Spleen	2, 20*	26^§^, 27
R7255 +	*M*. *cervicolor*	Nakhon Ratchasima	Liver	2, 21^§^	28^§^, 29
R7259	*M*. *cervicolor*	Nakhon Ratchasima	Liver	1, 2	30, 31
R7262 +	*M*. *cervicolor*	Nakhon Ratchasima	Liver	2, 22	14, 32
R7263	*M*. *cervicolor*	Nakhon Ratchasima	Liver	23	33, 34
R7121	*M*. *cookii*	Tak	Liver	1	1, (2)
R7180	*M*. *cookii*	Nan	Liver	2, (3)	(3), (2)
R7210	*M*. *cookii*	Nan	Liver	4	(4)
R7237	*M*. *cookii*	Nan	Liver	4, (5)	(4)
R7238	*M*. *cookii*	Nan	Liver	4	(4), (5)
R7239	*M*. *cookii*	Nan	Liver	(3), (6)	8, (6)
R7243	*M*. *cookii*	Nan	Liver	7	(7)
R7254	*M*. *fragilicauda*	Nakhon Ratchasima	Liver	1, 2	
R7260	*M*. *fragilicauda*	Nakhon Ratchasima	Liver	3, 4	
R7261	*M*. *fragilicauda*	Nakhon Ratchasima	Liver	3, 4	

Variants in parentheses denote the presence of a premature stop codon that results in a truncated reading frame.

^§^ indicates an extended C-terminus

* indicates internal repeats

** indicates variants preceded by 2 residues before the consensus start codon, + indicates samples analyzed twice. Fv1CAR1 and Fv7CAR1 have previously been reported and accessioned as KF975446 and KF975447 [17] and have not been re-sequenced here.

Extensive sequence differences were visible, including point mutations, short insertions and deletions, and a variety of duplications (Table 1, S1 Table, and below). To confirm that the observed levels of variation were not artefacts of PCR amplification, we repeated the PCR, cloning, and sequencing for 6 samples (Table 1), specifically including those with sequence duplications. In all cases, the clones sequenced exactly matched those seen originally. Moreover, we only once observed more than two sequences per animal with a given primer pair; this one exception could be explained by recombination between *Fv1* and *Fv7* and was, therefore, excluded from all further analysis and is not reported here. Thus, the variation seen truly reflects genetic variation in the natural population, with heterozygosity frequently observed for both genes.

**Fig 2:**
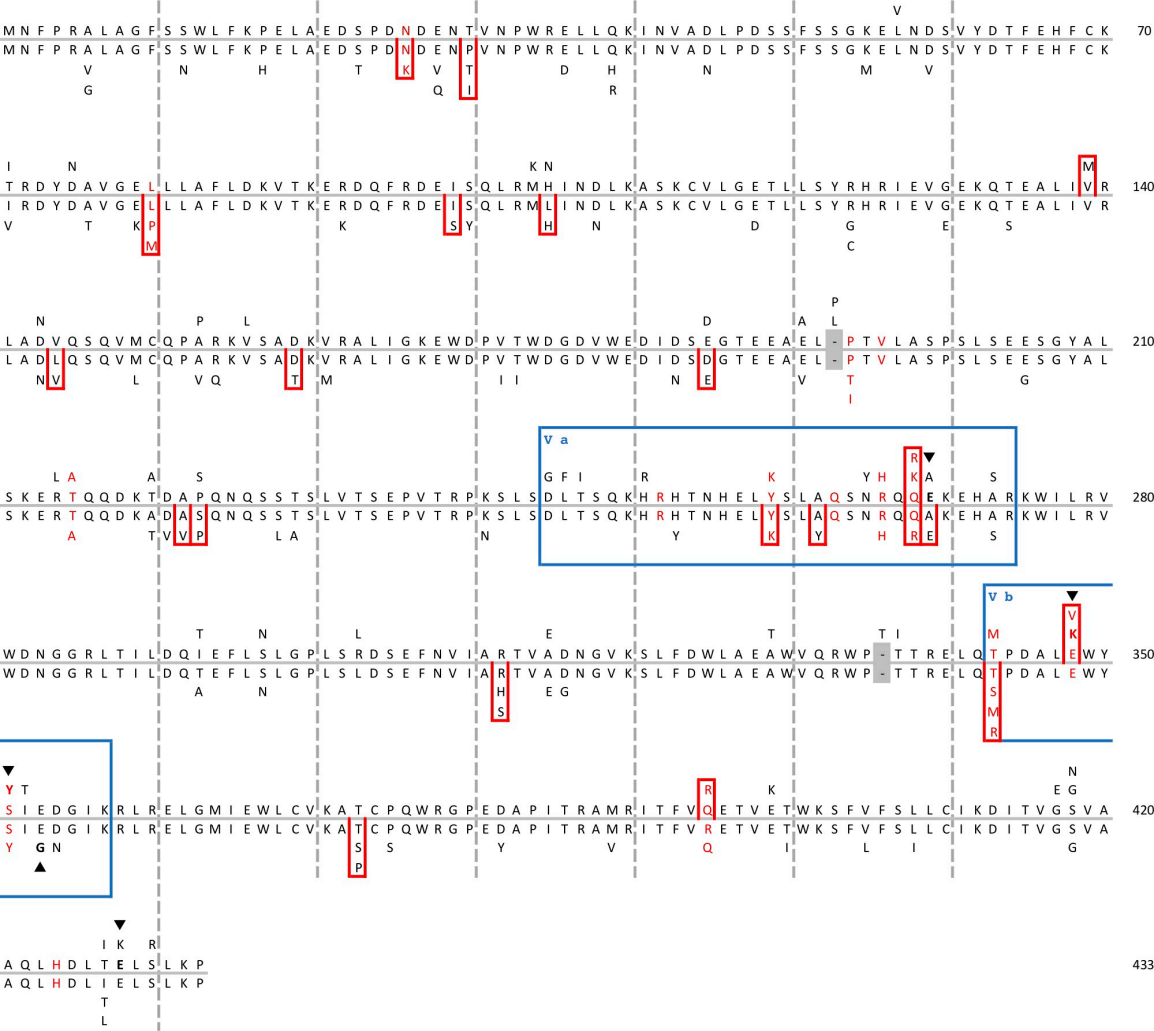
*Fv1* and *Fv7* variability across the South East Asian clade. Collapsed representation of the multiple sequence alignment of those *Fv1* (extending upward) and *Fv7* (extending downward) sequences with intact ORFs, with the most frequent residue toward the center. Alignment gaps are shaded gray. Sites under pervasive positive selection are boxed red for each species separately and, for comparison, residues previously identified as positively selected [38] are highlighted with red text. Restriction determinants newly determined or discussed within this study are highlighted in bold text and indicated by arrows. The previously identified variable regions, V_A_ and V_B_ [17], are boxed and labelled in blue.

Representative examples of novel *Fv1* and *Fv7* alleles from each species were compared with *Fv1*^n^ and *Fv1*^b^. Echoing our previous reports [17] and reflecting the basal position of the South East Asian clade within the *Mus* subgenus, all novel *Fv1* and *Fv7* alleles lacked the three amino acid insertion in the NTD otherwise characteristic of this group. All Fv1CAR alleles contained a single amino acid insertion at position 197 and the majority (5 of 7 novel alleles, along with CAR1/Fv1CAR1) contained another individual insertion at position 337. The most striking differences were visible at the C-terminus of the protein. In this region, all Fv1CAR alleles were 10 amino acids longer than *Fv1*^b^ and 7–8 longer than the majority of *Fv7* alleles from any species, whereas the *Fv1* alleles of *M*. *cervicolor*, *M*. *cookii*, and *M*. *fragilicauda* were around 20 amino acids shorter than the *Fv7*s (Fig 2, S1 Fig, S2 Fig, S1 Table).

The shortening of all *Fv1* alleles from *M*. *cervicolor* and *M*. *cookii* was the result of a B1 SINE insertion causing truncation of the ORF and termination with the sequence AG(G)RGGARF (S2 Fig). Consistent with current estimations of *Mus* phylogeny [41, 42], the absence of the B1 repeat in *M*. *caroli* indicated insertion after the separation of *M*. *caroli* from the MRCA of *M*. *cervicolor* and *M*. *cookii*. Interestingly, the *Fv1* alleles of *M*. *fragilicauda* also contained a B1 SINE apparently at the same position, yet other sequence differences that consistently segregate the genes of the species, as well as the earlier divergence of this species from the South East Asian clade, suggest its independent acquisition rather than through recombinational admixture as a result of introgression, although this cannot fully be excluded. Indeed, we have previously reported the presence of 3 independent B1 insertions in other mouse species (*M*. (*Mus*) *famulus*, *M*. (*Nannomys*) *minutoides*, and *M*. (*Pyromys*) *platythrix*) within a few nucleotides of those seen here [17] and, similarly, the phylogenetic and geographic separation of these species argued conclusively against these features being the result of introgression. Rather, our previous work indicated that minimization of the length of the C-terminus may provide enhanced restriction properties [17, 27] and, thus, this provides further suggestive evidence for a convergent exploitation of the mobility of B1 SINEs in realizing this adaptation across species.

A number of alleles encoded frameshifted or truncated proteins, which were particularly common amongst the *M*. *cookii* samples; indeed, only 2 of 8 Fv7COO alleles encoded an ORF (Table 1, S1 Table). We have previously shown that truncation of *Fv1* to 410 amino acids results in complete loss of restriction activity [27], making functionality of these truncations improbable. Nevertheless, taking each mouse sampled individually and considering the natural heterozygosity observed at both loci (Table 1), whilst all *M*. *cookii* harbored at least one defective allele, they all also possessed at least one allele of either gene with intact coding potential.

To examine the level of sequence variation within the South East Asian clade more comprehensively, we conducted an analysis of dN/dS ratios across separate trees of the *Fv1* and *Fv7* sequences determined to have intact ORFs and to be free from internal duplications (S3 Fig). Analyses were conducted for both pervasive selection (FUBAR, which assumes that selection pressures for each site are constant throughout a phylogeny and assesses selection across all branches) and episodic selection (MEME, which determines selection at individual sites within a subset of branches). Signatures of positive, diversifying, selection were visible within both *Fv1* and *Fv7* (Fig 2, S2 Table) and the positions identified corresponded well with previous observations [38]. In total, 19 sites displayed pervasive positive selection and a single additional site displayed episodic positive selection. Tandem, cyclical, evolution of viral pathogens and restriction factors can complicate selection analyses due to residue resampling at specific sites and can act to obscure evolutionary paths [38, 43]. Likely as a result of this issue, the monophyly of *Fv7* is not supported by these data when *Fv1* and *Fv7* are included in a single phylogenetic tree; in fact, we note that residue resampling is observed at 15 of 20 sites positively selected (S2 Table), the majority of which occur in branches whose separation is well-supported by bootstrapping, including between species (S3 Fig).

Overall, our results showed the presence of a rich variety of *Fv1* and Fv7 sequences in the wild mouse populations of South East Asia and a strong role for positive selection in their development, alongside a potential exploitation of retroelement mobility as a means of separation and diversification of their protein sequences.

### Expression of Fv1 and Fv7

Despite its retroviral origin, a viral long terminal repeat on the 5’ side of *Fv1* is not present within *Mus*, although degraded fragments can be noted in more distantly-related genera [38]. In its absence, transcription was, therefore, thought to be driven from the bidirectional promoter activity of the adjacent antisense gene, *Miip*. Neither the exact promoter region nor the point of transcript initiation has been defined, however, raising the question of whether the duplicated region on Chr 6 retained the potential to drive expression of *Fv7*. Hence, we set out to map the promoter region of the parental *Fv1* locus.

**Fig 3:**
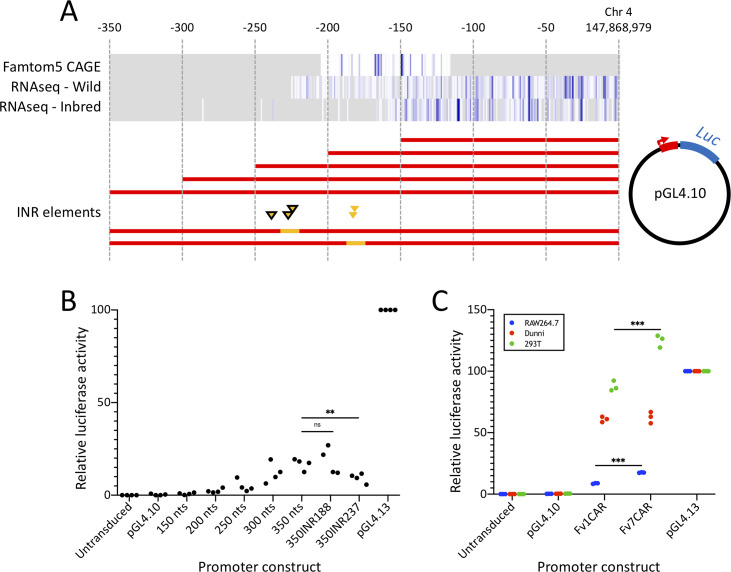
Analysis of *Fv1* transcription. **A**. Schematic of plasmid constructs produced to test for promoter activity within the region 5’ of the *Fv1* CDS. Heatmaps (white to blue, grey indicates no data) detail the position of identified transcription start sites within FANTOM5 CAGE data and, separately, for the positions of Illumina RNAseq reads from wild mice and inbred strains. Yellow triangles indicate the positions of INR element predictions (black borders indicate high confidence predictions) and accompanying yellow highlights indicate regions mutated to adenine. **B, C**. Relative luciferase intensity for control plasmids (Untransduced, pGL4.10, and pGL4.13) and experimental constructs tested for promoter activity. Data points are from independent experiments analyzed in triplicate and normalized to the intensity of pGL4.13. Paired two-tailed student's T-tests were used to determine significant differences. Lengths of the promoter regions from *M*. *caroli* included within Fv1CAR (363 nts) and Fv7CAR (703 nts) differ due to the presence of a B1 SINE upstream of the *Fv7* CDS.

Given that 329 nts of the region upstream of *Fv1* was duplicated alongside *Fv7*, fragments containing increasing lengths of the region 5’ of the C57BL/6J *Fv1*^b^ CDS on Chr 4, from 150 to 350 nts to encompass this region, were cloned into pGL4.10 ahead of a promoterless *Luc* gene (Fig 3A). The constructs were transfected into *M*. *dunni* tail fibroblast (MDTF) cells and the luciferase activities measured. Relative luciferase activity first increased above the background of the promoterless plasmid with the construct containing 250 nts upstream of the *Fv1* CDS and further increased with inclusion of regions up to 300 and 350 nts (Fig 3B).

To better determine the widest possible range of points of transcriptional initiation, we extracted cap analysis gene expression (CAGE) data from the FANTOM5 project [44] for endogenous *Fv1* expression, using pooled data from different tissues, sorted cell populations, treatments, and animal ages, as compiled and released by the project consortium. Dispersed transcription start sites were identified between 80 to 170 nucleotides 5’ of the Fv1 CDS (Fig 3A) but did not represent the full extent of transcription within the region determined in a complementary analysis of RNAseq reads from 9 inbred laboratory mouse strains (accession ERP000614 [45]), which identified dispersed points of initiation beyond 200 nts 5’ of the *Fv1* CDS (Fig 3A). Three partially-overlapping high-confidence initiator (INR) element predictions with 94–97% satisfaction of an INR position weight matrix (PWM) model [46] could be determined that supported the area additionally identified within the RNAseq data (Fig 3A), whereas only two, overlapping, low-confidence (81% PWM model satisfaction), predictions could be made within the areas identified by CAGE (Fig 3A). Whilst initiation is certainly dispersed, therefore, we sought to investigate any specific contributions of these regions with mutated constructs. Replacement of the high-confidence INR at 237 nts 5’ of the *Fv1* CDS with adenines produced a significant reduction in luciferase expression (Fig 3B), suggesting its likely involvement in *Fv1* transcription. By contrast, replacement of the low-confidence INR element at 188 seemed unimportant.

The promoter region of *Fv1* is likely cryptic and transcriptional initiation can be seen to occur across of range of sites. Nevertheless, these data confirmed that a region likely sufficient for expression had been retroduplicated onto Chr 6. To confirm explicitly that the sequence upstream of *Fv7* could drive expression, we further cloned this region, as well as that upstream of *Fv1*, from *M*. *caroli* and assayed promoter activity using the pGL4.10 system. Both regions robustly drove luciferase expression to around 60% of that of the pGL4.13 control (Fig 3C). Interestingly, therefore, whereas observed activity for the 350 nt construct was less than 25% of the pGL4.13 SV40 control for the C57BL/6 region in MDTF cells (Fig 3B), consistent with the low levels of endogenous *Fv1* expression previously described [47], the equivalent region upstream of *Fv1* in *M*. *caroli* (363 nts) drove notably higher luciferase expression (Fig 3C, Fv1CAR). Similarly, a longer region upstream of *Fv7* (703 nts), made to encompass the B1 SINE insertion, drove equally high expression (Fig 3C, Fv7CAR). To investigate this further we tested expression in two additional cell lines (murine RAW264.7 and human 293T), which revealed significant differences when comparing the region upstream of *Fv1* and *Fv7*, as well as greatly varying expression levels when comparing between cell lines (Fig 3C).

To ensure that co-expression of *Fv1* and *Fv7* could occur *in vivo*, we analyzed RNAseq data for the CAROLI/EiJ inbred mouse line (accessions ERP023198 and ERP005559 [48, 49]). The high levels of nucleotide identity between the genes, alongside the low levels of expression (resulting in incomplete gene coverage), complicated expression assessment due to ambiguity in assignation of multi-mapping reads. Instead, we turned to a qualitative means of confirming that both genes were expressed. For both experiments analyzed, reads aligning uniquely to either *Fv1* or *Fv7* were used to form consensus sequences across the regions represented. These fragments were included in a multiple sequence alignment alongside Fv1CAR1 and Fv7CAR1, the alleles of *Fv1* and *Fv7* from CAROLI/EiJ [17], and inspected at sites at which the two differ. Consensus sequences derived from both RNAseq experiments confirmed the expression of both *Fv1* and *Fv7* (S4 Fig). Further, this confirmed that the region retroduplicated onto Chr 6 is sufficient for *in vivo* expression and that co-expression occurs naturally.

### Restriction specificities of cloned Fv1s and Fv7s

As previously hypothesized, high levels of sequence variation within *Fv1* may be due to selection by a range of retroviruses, which are likely to have contributed to maintenance and diversification of the gene [38]. The extensive variation among the Fv1 and Fv7 sequences observed here thus led us to wonder if they were capable of recognizing multiple viruses and we tested a subset of these novel sequences for their ability to restrict a comprehensive panel of retroviruses (Tables 2 and 3).

**Table 2:** Restriction of MLVs by selected Fv1s and Fv7s.

Variant	N-MLV	B-MLV	Mo-MLV	NR-MLV
Fv1CAR2	1.16 ± 0.02	*0*.*34 ± 0*.*02*	1.01 ± 0.02	1.16 ± 0.10
Fv1CAR3	1.10 ± 0.14	*0*.*34 ± 0*.*02*	0.83 ± 0.06	1.12 ± 0.12
Fv1CAR4	1.19 ± 0.04	*0*.*53 ± 0*.*08*	1.08 ± 0.05	1.12 ± 0.12
Fv1CER1	*0*.*35 ± 0*.*02*	**0.03 ± 0.01**	1.06 ± 0.12	*0*.*43 ± 0*.*05*
Fv1CER2	**0.04 ± 001**	**0.04 ± 0.01**	1.03 ± 0.06	**0.05 ± 0.01**
Fv1CER19	**0.09 ± 0.03**	**0.06 ± 0.01**	1.03 ± 0.03	**0.09 ± 0.01**
Fv1CER21	**0.13 ± 0.01**	**0.12 ± 0.01**	1.06 ± 0.01	N.D.
Fv1CER22	1.16 ± 0.01	1.17 ± 0.12	1.16 ± 0.05	1.11 ± 0.08
Fv1COO1	1.13 ± 0.07	**0.15 ± 0.01**	1.23 ± 0.10	1.19 ± 0.21
Fv1COO2	**0.07 ± 0.01**	**0.06 ± 0.01**	*0*.*33 ± 0*.*05*	**0.07 ± 0.01**
Fv1COO4	1.11 ± 0.04	**0.03 ± 0.01**	1.14 ± 0.03	1.21 ± 0.07
Fv1COO7	**0.03 ± 0.01**	**0.03 ± 0.01**	**0.13 ± 0.06**	**0.03 ± 0.01**
Fv1FRA1	**0.09 ± 0.01**	**0.09 ± 0.01**	**0.08 ± 0.01**	**0.09 ± 0.00**
Fv1FRA2	**0.09 ± 0.00**	**0.09 ± 0.01**	**0.10 ± 0.01**	**0.10 ± 0.01**
Fv7CAR2	1.12 ± 0.17	0.70 ± 0.16	0.87 ± 0.09	1.16 ± 0.02
Fv7CAR3	1.18 ± 0.28	0.74 ± 0.28	0.82 ± 0.09	1.23 ± 0.20
Fv7CAR4	0.70 ± 0.02	1.03 ± 0.24	0.86 ± 0.07	1.14 ± 0.13
Fv7CER26	1.16 ± 0.04	1.14 ± 0.03	1.16 ± 0.02	1.09 ± 0.03
Fv7CER27	*0*.*41 ± 0*.*05*	1.41 ± 0.17	1.18 ± 0.03	1.15 ± 0.03
Fv7CER29	1.06 ± 0.01	1.14 ± 0.06	1.12 ± 0.03	1.14 ± 0.06
Fv7CER30	*0*.*33 ± 0*.*04*	1.22 ± 0.08	1.16 ± 0.08	1.18 ± 0.01
Fv7CER31	*0*.*37 ± 0*.*05*	1.23 ± 0.05	1.14 ± 0.02	1.16 ± 0.24
Fv7COO1	**0.02 ± 0.01**	1.04 ± 0.35	1.17 ± 0.25	1.21 ± 0.23
Fv7COO8	**0.06 ± 0.04**	0.97 ± 0.22	0.88 ± 0.34	1.13 ± 0.10

Restriction assays were performed as described in the Materials and Methods. Values are the means and standard deviations of at least 4 experiments. Bold type indicates full restriction (≤0.3) and italics indicates partial restriction (>0.3, ≤0.7).

**Table 3:** Restriction of non-MLV retroviruses by Fv1s and Fv7s.

Variant	FIV	EIAV	PFV	FFV
Fv1CAR2	0.82 ± 0.10	1.13 ± 0.11	*0*.*44 ± 0*.*01*	**0.12 ± 0.01**
Fv1CAR3	0.93 ± 0.04	1.07 ± 0.01	*0*.*31 ± 0*.*01*	**0.12 ± 0.01**
Fv1CAR4	0.85 ± 0.09	1.12 ± 0.01	*0*.*51 ± 0*.*03*	**0.19 ± 0.01**
Fv1CER1	1.10 ± 0.06	0.91 ± 0.12	1.01 ± 0.06	0.99 ± 0.02
Fv1CER2	1.05 ± 0.04	0.72 ± 0.03	0.99 ± 0.10	0.99 ± 0.03
Fv1CER19	1.04 ± 0.01	*0*.*60 ± 0*.*03*	1.00 ± 0.01	1.00 ± 0.01
Fv1CER21	1.09 ± 0.05	1.01 ± 0.02	0.98 ± 0.01	0.99 ± 0.01
Fv1CER22	1.04 ± 0.13	0.71 ± 0.06	0.93 ± 0.02	1.00 ± 0.01
Fv1COO1	0.92 ± 0.13	0.97 ± 0.17	0.89 ± 0.15	1.02 ± 0.02
Fv1COO2	0.96 ± 0.05	0.92 ± 0.14	0.93 ± 0.06	1.00 ± 0.01
Fv1COO4	1.12 ± 0.14	0.91 ± 0.05	0.94 ± 0.04	0.95 ± 0.01
Fv1COO7	0.95 ± 0.04	0.89 ± 0.31	0.95 ± 0.04	1.05 ± 0.01
Fv1FRA1	1.12 ± 0.01	**0.30 ± 0.02**	0.98 ± 0.02	1.02 ± 0.02
Fv1FRA2	1.08 ± 0.02	1.03 ± 0.01	1.00 ± 0.01	1.01 ± 0.01
Fv7CAR2	*0*.*47 ± 0*.*05*	**0.26 ± 0.04**	0.74 ± 0.01	1.03 ± 0.01
Fv7CAR3	*0*.*49 ± 0*.*06*	**0.23 ± 0.02**	0.87 ± 0.03	1.03 ± 0.01
Fv7CAR4	0.76 ± 0.03	*0*.*31 ± 0*.*08*	0.84 ± 0.03	0.99 ± 0.04
Fv7CER26	1.00 ± 0.01	0.89 ± 0.03	1.05 ± 0.04	1.02 ± 0.02
Fv7CER27	0.85 ± 0.04	*0*.*36 ± 0*.*04*	0.95 ± 0.01	0.95 ± 0.01
Fv7CER29	0.98 ± 0.02	0.90 ± 0.09	0.96 ± 0.04	1.02 ± 0.01
Fv7CER30	0.89 ± 0.08	*0*.*37 ± 0*.*07*	0.97 ± 0.01	1.02 ± 0.08
Fv7CER31	0.79 ± 0.02	*0*.*51 ± 0*.*03*	0.86 ± 0.01	1.02 ± 0.04
Fv7COO1	*0*.*34 ± 0*.*09*	**0.21 ± 0.07**	0.86 ± 0.12	0.99 ± 0.06
Fv7COO8	*0*.*33 ± 0*.*02*	**0.18 ± 0.04**	0.83 ± 0.11	1.03 ± 0.02

Restriction assays were performed as described in the Materials and Methods. Values are the means and standard deviations of at least 4 experiments. Bold type indicates full restriction (≤0.3) and italics indicates partial restriction (>0.3, ≤0.7).

Contrary to our previous report analyzing Fv1CAR1 (then termed CAR1) [17], which determined no anti-gammaretroviral activity, all three Fv1CAR alleles tested here gave partial restriction of B-MLV (Table 2). Comparison of Fv1CAR1 and Fv1CAR2 showed three amino acid differences and exchange of a single residue, Fv1CAR428, restored activity against B-MLV without affecting that seen against FFV (Table 3, S5 Fig, Fig 2). In contrast to the Fv1CAR alleles tested, which restricted only B-MLV, the majority of Fv1CER and Fv1COO alleles showed activity against a wider array of the gammaretroviruses tested (Table 2). Fv1COO4 and Fv1COO7, differed in their abilities to recognize N-MLV, NR-MLV, and Mo-MLV; this difference could, in the case of N-MLV and Mo-MLV, be mapped to a single amino acid (Fv1COO268) (S6 Fig, Fig 2). The same amino acid could also modulate restriction specificity in Fv1CER2 (S6 Fig). On several occasions, e.g. Fv1CAR2, 3, 4, and Fv1COO1, reduced restriction activity seemed correlated with a longer C-terminal region (Table 2, S1 Table) and it would be interesting to test the effect of artificially truncating the Fv1s from Fv1CAR2 and Fv1COO1 in a manner analogous to that seen with the B1 repeat in Fv1CER. Thus, all but one *Fv1* allele tested showed activity against at least one gammaretrovirus in the panel.

Consistent with our previous study [17], Fv1CAR2, Fv1CAR3, and Fv1CAR4 all showed anti-foamy virus activity, restricting FFV fully and PFV to a lesser extent (Table 3). None of the other factors tested had this effect. All Fv1CAR alleles in this study contained the determinants (K348 and Y351, Fig 2) previously identified as mediating this restriction profile [17], suggesting that activity against foamy viruses is a feature common across the *Fv1* alleles of *M*. *caroli*. In turn, this might suggest a widespread exposure to foamy viruses or to similar, unidentified, viruses in the current *M*. *caroli* population in Thailand–individual samples coming from Prachuapkirikhan in the South, Kalasin in the East, and Nan in the North (Table 1).

Interestingly, 1 of 2 Fv1FRA (*M*. *fragilicauda*) alleles tested, alongside 8 of 10 *Fv7* alleles from *M*. *caroli*, *M*. *cervicolor*, and *M*. *cookii* exhibited full or partial EIAV restriction (Table 3). We have previously mapped the ability of *M*. *spretus Fv1* to recognize EIAV to a R268C change [17] and, similarly here, we find that a C is again present at the analogous position in Fv1FRA1, which restricts, but not in Fv1FRA2, which does not. This amino acid is not found in any of the restricting Fv7s, however, where the change or changes responsible for restriction have remained elusive. These active Fv7s further differ from Fv1FRA1 in their ability to partially restrict FIV, highlighting that the observed activity spans multiple lentiviruses, rather than being a directed against a feature of a particular, individual, capsid (Table 3). We had previously cloned Fv7CAR1 from *M*. *caroli* (then termed CAR2) but had not noted an anti-lentiviral activity [17]. Comparison of Fv7CAR1 with the Fv7CAR alleles cloned here revealed the presence of E351 in EIAV-restricting variants and, indeed, G351E restored restriction in Fv7CAR1 (S7 Fig, Fig 2). These results would be consistent with the presence of a lentiviral pathogen endemic in the area and selecting for the observed restriction activity, although pressure exerted by a similar, unidentified, virus cannot be fully excluded.

### Combining EIAV and FFV restriction

Two individual *M*. *caroli* samples from different locations, identifiers R6321 and R6657 (Table 1), both carried an Fv1 that restricted FFV and MLV and an Fv7 that restricted EIAV and FIV. Indeed, based on the sequences described here, with the conserved features mentioned above (K358 and Y351 in Fv1CAR and the absence of E351 in Fv7CAR), it seems possible that this applies to all *M*. *caroli* sampled, suggesting conference of a certain selective advantage. This raised the question as to whether such differing restriction profiles could be achieved within a single gene or whether gene duplication and diversification was required to achieve such broad recognition.

To examine this idea further, we first tried creating a single restriction factor with the ability to recognize both lenti- and foamy viruses. Introduction of the residues conferring FFV restriction [17] into the Fv7s recognizing EIAV achieved only a very weak restriction in Fv7CAR2 and Fv7CAR3 but not in Fv7CER27 and Fv7COO8 (Table 4). Further, in all cases, the EIAV restriction was abolished. The alternate introduction of FFV determinants into Fv1^n^ carrying R268C, a construct previously shown to re-create anti-EIAV activity [17], again proved unsuccessful (Table 4).

**Table 4:** Incompatibility of FFV and EIAV restriction determinants.

Variant	FFV	EIAV	N-MLV	B-MLV
CAR1	**0.15 ± 0.06**	1.10 ± 0.14	1.09 ± 0.09	1.08 ± 0.06
Fv7CAR2	1.01 ± 0.05	**0.24 ± 0.07**	1.04 ± 0.07	1.08 ± 0.05
Fv7CAR2 E349K S352Y	*0*.*68 ± 0*.*18*	0.99 ± 0.08	1.12 ± 0.03	1.05 ± 0.04
Fv7CAR3	1.00 ± 0.04	**0.20 ± 0.02**	1.03 ± 0.10	1.09 ± 0.06
FvCAR3 E349K S352Y	*0*.*59 ± 0*.*11*	0.93 ± 0.11	1.06 ± 0.03	1.04 ± 0.09
Fv7CER27	1.01 ± 0.06	*0*.*36 ± 0*.*05*	*0*.*46 ± 0*.*04*	1.12 ± 0.05
Fv7CER27 E349K S352Y	1.04 ± 0.03	1.06 ± 0.03	1.07 ± 0.05	0.94 ± 0.07
Fv7COO8	0.99 ± 0.06	**0.22 ± 0.04**	**0.11 ± 0.04**	1.08 ± 0.09
Fv7COO8 E349K S352Y	1.02 ± 0.01	1.00 ± 0.12	1.08 ± 0.03	1.11 ± 0.05
Fv1n	1.04 ± 0.03	1.21 ± 0.05	1.12 ± 0.02	**0.09 ± 0.02**
Fv1n R268C	1.00 ± 0.02	**0.23 ± 0.02**	**0.15 ± 0.03**	**0.16 ± 0.02**
Fv1n E349K S352Y	**0.13 ± 0.03**	1.12 ± 0.08	1.07 ± 0.04	**0.04 ± 0.01**
Fv1n R268C E349K S352Y	0.99 ± 0.04	1.23 ± 0.04	*0*.*45 ± 0*.*02*	**0.21 ± 0.06**

Restriction assays were performed as described in the Materials and Methods. Values are the means and standard deviations of at least 4 experiments. Bold type indicates full restriction (≤0.3) and italics indicates partial restriction (>0.3, ≤0.7).

Alternatively, and considering that Fv1 activity was initially described as co-dominant, we sought to test whether co-expression of Fv1s with different restriction specificities could protect a cell against multiple viruses. For this purpose, a three-color flow cytometry restriction assay was established in which permissive MDTFs were transduced with two retroviral vectors expressing different restriction factors together with either EYFP or mScarlet, so that cells which were transduced with one restriction gene were either yellow or red while those containing both restriction genes were doubly labelled (S8 Fig). The mixed population was then challenged with tester viruses carrying an EGFP construct. Thus, each population could be individually identified by FACS, allowing infection susceptibility to be scored as the percentage of green cells within each population (S8 Fig). Restriction was expressed as the ratio of the percentage of infection in cells containing restriction factors (either yellow, red, or yellow and red) to those which did not (unlabelled).

**Fig 4:**
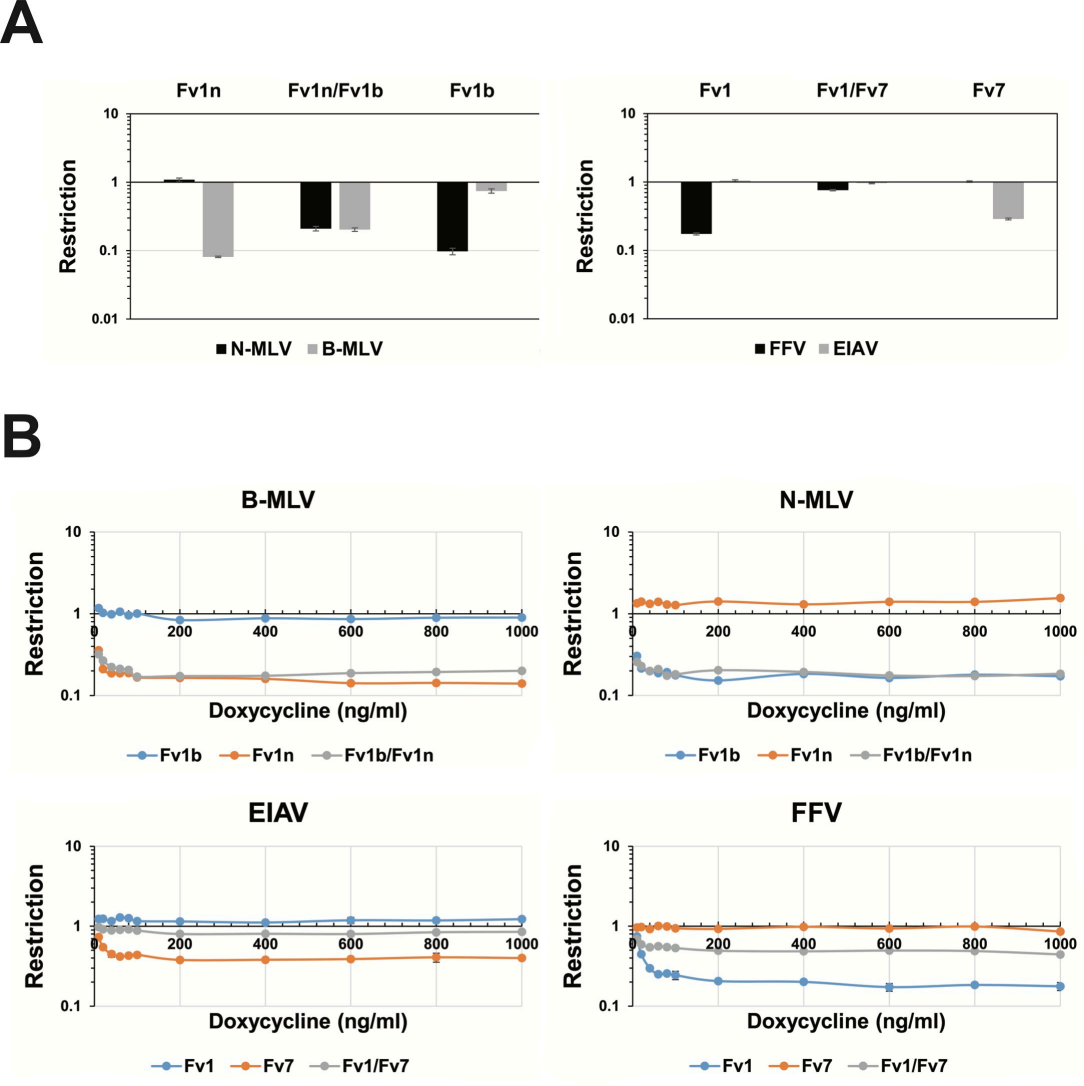
Co-expression of Fv1CAR and Fv7CAR. **A**. Expression from a retroviral promoter. MDTFs were transduced to co-express Fv1^b^/mScarlet and Fv1^n^/EYFP (left) or Fv1CAR2/mScarlet and Fv7CAR2/EYFP (right) and challenged with either N-MLV, B-MLV, FFV or EIAV carrying the EGFP gene. **B**. Expression from an inducible promoter over a range of induction. The same combinations of fluorescence genes and Fv1 or Fv7 were placed under the control of a doxycycline inducible promoter in retroviral vectors that have been previously described and used to transduce R18 cells. The cells were induced with doxycycline concentrations from 10 ng/ml to 1000 ng/ml for 24 hours before challenge. In both A and B, restriction is expressed as the ratio of the percentages of cells containing restriction factor(s) that were infected to those of cells that did not contain restriction factor and were infected.

To aid in developing the assay, we initially tested the two alleles of *Fv1* common amongst inbred laboratory mice, *Fv1*^*n*^ and *Fv1*^*b*^ (restricting B-MLV and N-MLV, respectively), which were originally described to be co-dominant in heterozygous animals [14–16]. Both variants individually provided strong restriction activity (Fig 4A) and, although slightly reduced in comparison, significant restriction of both N-MLV and B-MLV was observed when both alleles were co-expressed, as expected. By contrast, in cells expressing both Fv1CAR2 and Fv7CAR2 (selected as both originate from the same mouse, identifier R6321, Table 1), complete loss of restriction of both FFV and EIAV was observed, indicating apparent interference between the co-present factors. Equivalent interference has previously been reported between the TRIM5α proteins of human and rhesus macaque and, similarly, between human TRIM5α and owl monkey TRIMCyp, a TRIM5-cyclophillin fusion [50].

We have previously noted that levels of *Fv1* expression can impact determination of restriction activities, however, as endogenous levels of *Fv1*^*n*^ and *Fv1*^*b*^ are very low [47]. As the first set of experiments was performed with vectors expressing the restriction factors from retroviral promoters, it was possible, therefore, that the reduction in restriction activities observed was due to their relative overexpression. To test this hypothesis, we repeated the assay using inducible promoters to express the restriction genes. As before, the ability of Fv1^n^ or Fv1^b^ to restrict either B-MLV or N-MLV, respectively, was almost identical whether they were present individually or together, and over a wide range of doxycycline concentrations (Fig 4B) shown previously to induce much higher levels of Fv1 than required for full restriction activity [47]. Across all levels of induction, however, co-expression of Fv1CAR2 and Fv7CAR2 abolished anti-EIAV activity and markedly reduced anti-FFV activity (Fig 4B). Even at physiological levels of expression, co-expression of these factors resulted in interference, therefore.

## Discussion

Diverse retroviruses have undoubtedly exerted sustained selection pressures through both human [33–35] and murid [37, 38] evolution. For both, a variety of ecological considerations–population density and exposure to other co-endemic species, for example–have influenced exposure to circulating retroviruses. These, as well as other spaciotemporal factors have likely contributed to the wide array of restriction profiles now visible across species of *Mus* [17]. Previous work [17], however, as well as experiments within the present study, indicate limitations in the ability of differing Fv1-based restriction profiles to be additively merged within single proteins. For example, attempts to generate an Fv1 that restricts both FFV and EIAV have not proved successful. Such limitations, possibly visualized as separate peaks within an evolutionary landscape, potentially limit overall restriction plasticity. We now detail the first example of *Fv1* duplication and the acquisition of differing restriction profiles within Fv7 and Fv1 as a means of enhancing restriction range. There appears to be a clear parallel with the acquisition of an extended functional repertoire of the APOBEC3 restriction factor in primates, which has also been mediated by retroduplication [51].

Given the presence of a 12 nt tandem site duplication and the integration of a non-templated region likely resulting from mRNA polyadenylation [18], it is probable that the *Fv7* locus on Chr 6 results from LINE-mediated retroduplication. However, the definitive hallmark of retrogenes, exon merger as a result of splicing [52], is missing because *Fv1* comprises a single exon. The region duplicated contains 329 nt of sequence upstream of the *Fv1* CDS on Chr 4, thereby encompassing sufficient sequence for promoter activity.

Compared to the long history of *Fv1*, the fixation of *Fv7* within the MRCA of the South East Asian clade, around 4 mya, is a comparatively recent event. As such, their sequence similarity remains high and, where we have successfully mapped certain restriction activities to specific amino acids, all fall within the previously defined variable regions of Fv1 responsible for restriction of different viruses [17, 38]. Examples include Fv1CAR1 residue 428 (S5 Fig), Fv1COO4 residue 268 (S6 Fig) and Fv7CAR1 residue 351 (S7 Fig). However, it is noteworthy that residues 358 and 399 of *M*. *musculus* Fv1, key for distinguishing between N-MLV and B-MLV in Fv1^n^ and Fv1^b^ [27], are identical across all Fv1s and Fv7s cloned here, despite the differences in MLV restriction visible at both the individual and species level (Table 2).

Our attempts *in vitro* to introduce FFV- and EIAV-restricting Fv1 and Fv7 variants into the same cell, even at endogenous expression levels, have not resulted in dual restriction (Fig 4). Fv1 restriction is thought to involve formation of a multimeric lattice around incoming virions [28] in a manner analogous to the TRIM5α complexes engulfing incoming retroviruses [30, 31, 53]. The incoming cores of lentiviruses and foamy viruses have different arrays of Gag proteins and it is possible that, at least within our assay system, formation of mixed Fv1/Fv7 complexes does not result in stable binding when admixed factors have differing restriction profiles. Nevertheless, it is clear that the generation, genetic fixation, and maintenance of different activities within these species has taken place and evidence of strong positive selection is apparent for both genes. This implies (i) functional expression of the two genes and (ii) the presence of endemic viruses exerting selective pressure.

The first conclusion gives rise to a certain paradox, therefore, given the apparent interference between the two factors. Separate spatial or temporal expression would present a means of mitigating this interference and, in support of such an explanation, it is noteworthy that the Fv7CAR locus has accumulated a B1_Mus2 SINE element upstream of and in the same orientation as the CDS. This is one of only few B1 SINE families showing potential links to gene regulation [54] and indeed, on testing, the promoter regions of Fv1CAR and Fv7CAR show differential activity in two separate cell lines.

Across the species surveyed, the Fv1 and Fv7 proteins show substantial variation and adaptation to recognize viruses of different genera. Unfortunately, a sparsity of whole genome sequencing data from multiple individuals of diverse *Mus* species prevents the comparison of relative rates of polymorphism. Nevertheless, an indicative comparison to sequences previously determined for *M*. *domesticus* and *M*. *musculus* [17, 36, 55] suggests a higher extent of sequence variation than might be expected. Behind the levels of allelism detailed, it seems probable that manifold viruses circulate within Thai mice. Though the viruses driving these changes have not been identified, on the practical assumption that the driver viruses resemble those defining the observed activities, given current knowledge of retroviral diversity, it would seem reasonable to conclude that Thai mice are, or have been, exposed to both foamy and lentiviruses. However, given that a wide diversity of retroviruses may still remain to be discovered [56], it is impossible to exclude that unknown viruses, potentially also now extinct within these populations, may instead form the targets of Fv1 and Fv7 within these species. Regardless, these viruses must have been sufficiently pathogenic to provide the selection pressures required for the generation, fixation, and divergence of novel resistance genes, as well as for their continued maintenance; in the absence of such a pressure, they would otherwise be lost after ~1.2 million years of background mutation [38]. Indeed, it is possible that such loss is currently occurring within *M*. *cookii*, where only 4 of 7 *Fv1* alleles and 2 of 8 *Fv7* alleles retain ORFs. This may be due to loss of exposure to the selecting virus, for example through receptor escape, but may also result from reduced selection pressures due to the adaptation or acquisition of an alternate restriction factor acting at an earlier point in the retroviral entry pathway.

To the best of our knowledge, no mouse-tropic foamy or lentiviruses have ever been described but a recent report detailing the acquisition of *TrimCyp* fusion events within murids, including one with a solely anti-lentiviral activity [57], is consistent with their current or extremely recent presence. Given the potential for murids to act as vector species [58], the search for such viruses has been, and remains, of considerable interest.

## Materials and methods

### Ethics statement

Rodent species included in the study are neither on the CITES list, nor the Red List (IUCN). Animals were treated in accordance with the guidelines of the American Society of Mammalogists and within the European Union legislation guidelines (Directive 86/609/EEC). Each trapping campaign was validated by the national, regional and local health authorities. Approval notices for trapping and investigation of rodents were provided by the Ethical Committee of Mahidol University, Bangkok, Thailand, number 0517.1116/661.

### Mice

Wild mice were trapped in different provinces of Thailand as listed in Table 1; spleens or livers were removed and frozen for later DNA extraction using the Qiagen DNeasy Blood and Tissue kit according to the manufacturer’s instructions. Species identification was confirmed by PCR with a mitochondrial DNA bar-coding method. Briefly, a segment of the cytochrome oxidase subunit 1(COI) gene was amplified from gDNA using the primers BatL5310 (5’ CCTACTCRGCCATTTTACCTATG 3’) and R6036R (5’ ACTTCTGGGTGTCCAAAGAATCA 3’). The sequence of the PCR fragment (S1 Text) was then used in a BLAST search to identify the COI gene of the rodent species with the closest identity (ceropath.org/barcoding_tool/rodentsea). A phylogenetic tree showing the clustering of the different sequences is shown in S9 Fig.

Inbred CAROLI/EiJ and SPRET/EiJ DNAs were similarly prepared from tissues purchased from The Jackson Laboratory. Initial genotyping was performed by PCR using primers Chr6F (5' CAAGAGTCCTATGTGTACCTTC 3') and Chr6Rev (5' GCAGGCCAATCATAGCACTG 3') or Chr4F (5' CAGCAACCACATGGTGACTC 3') carried out in 50 μl reactions containing 2.5 U of Pfu ultra, 100 ng of template, 0.2 mM dNTPs and 0.5 μM each of the forward and reverse primer. The reaction was performed in a thermal cycler at 95°C for 2 minutes followed by 25 cycles of 95°C for 1 minute, 57°C for 2 minutes and 72°C for 3 minutes.

### *Fv1* and *Fv7* cloning

*Fv1* and *Fv7* were cloned using Q5 high fidelity polymerase (New England BioLabs) with primers Fv1GenStopRev (5’ CCTCCTGATTTTAAGCTCTTTAAC 3’) and either Chr4Fv1 (5’ CCAATTGACAGTGCCAGGACGCC 3’) or Chr6Fv7 (5’ CAGAAGCTCTGTCTTAGGGGAC 3’) to amplify *Fv1* and *Fv7* respectively. The bands were excised from a 1% agarose gel and purified with QIAquick Gel Extraction kit before cloning into the Zero Blunt Topo vector (Invitrogen). Eight colonies from each reaction were picked for sequencing and the resulting novel alleles deposited (accessions MT077217-MT077308, S1 Table, S2 Text).

Variants were amplified from this vector using Q5 high fidelity polymerase with primers GibsonFv1F (5’ GCCCCCATATGGCCATATGAGATCTGGACGCAGCAGCCGAGTT 3’) and GibsonFv1Rev (5’ ATCCCGGGCCCGCGGTACCGAGATCTCCTCCTGATTTTAAGCTCTTTAACTGTTGC 3’) and purified on 1% agarose gels before cloning into a BglII and SalI digested delivery vector using HiFi assembly (New England BioLabs), for use in restriction assays.

### Site directed mutagenesis

A PCR based strategy was used to introduce site directed changes to the *Fv1* or *Fv7* genes. 10 ng of plasmid carrying the gene was used together with 150 ng of each primer containing the altered sequence and spanning the site to be mutated. The reaction was performed using PfuUltra (Agilent) with 18 cycles of denaturation at 95°C for 30 seconds, 55°C for 1 minute and 68°C for 9 minutes 30 seconds. The reaction mixture was then digested with DpnI (New England BioLabs) for 1 hour before using 4 μl for the transformation of XL10 gold ultracompetent cells (Agilent). Colonies were screened for the mutation and verified by sequencing.

### Cells and virus production

MDTF and 293T cells were maintained in Dulbecco’s modified Eagle’s media containing 10% fetal calf serum and 1% penicillin/streptomycin. Viruses were made by transient transfection of 293T cells as described previously [17, 59, 60]. To make delivery viruses for transducing permissive MDTF with *Fv1/Fv7*, pczVSVG and pHIT60 were co-transfected with pLIEYFP carrying the *Fv1* or *Fv7* variant. Apart from the foamy viruses, the tester viruses were all pseudotyped with VSVG. N-tropic, B-tropic, Mo-MLV and NR-tropic MLV were made by co-transfecting pczVSVG and pfEGFPf with either pCIGN, pCIGB, pHIT60 or pCIGN(L117H) respectively. EIAV was made by co-transfection of pczVSVG, pONY3.1 and pONY8.4ZCG [61] while FIV was produced with pczVSVG, pFP93 and pGiNWF-G230 [62]. PFV and FFV were generated using pciSFV-1envwt and either pczDWP001 or pcDWF003 respectively [63]. MLVs and FIV were aliquoted and frozen at -80°C after harvesting while EIAV and foamy viruses were used fresh. Transduction using EIAV and FIV were performed in the presence of 10 μg/ml polybrene.

### Restriction assay

Restriction activity was measured using a flow cytometry-based assay as described previously [59, 60]. Briefly, the *Fv1* and *Fv7* genes were delivered into permissive MDTF cells using a Mo-MLV-based bi-cistronic vector which also contains EYFP in the same transcriptional unit so that all cells that express the restriction factor would also fluoresce yellow. Three days later, the cells were challenged with a tester virus that carried EGFP so that infected cells fluoresced green. Three days post-infection, the cells were analyzed by flow cytometry to obtain the ratio of the number of infected cells (green) containing restriction factors (yellow) to infected cells that did not contain restriction factors (non-yellow). A ratio of less than 0.3 was indicative of full restriction, a value between 0.3 and 0.7 was taken to represent partial restriction, and a ratio greater than 0.7 showed the absence of restriction.

In order to study the effect of expressing two different restriction factors in the same cell, the assay described above was modified by transducing MDTF (factors expressed from retroviral promoter) or R18 cells (factors expressed from inducible promoter) with the EYFP vector containing the first restriction gene together with a vector containing the second restriction gene and mScarlet. pmScarlet_C1 [64] was a gift from Dorus Gadelia (Addgene plasmid 85042; http://n2t.net/addgene:85042; RRID:Addgene_85042). Three days later, the cells were challenged with a tester virus that carried EGFP. Cells containing one factor were either yellow or red while those transduced with both factors were yellow and red (S8 Fig, center). The different populations, together with untransduced cells, were analyzed by flow cytometry to obtain the percentage of infected (green) cells in each population (S8 Fig, periphery).

### Phylogenetic analysis and determination of selection

Nucleotide sequences for alleles determined to have intact ORFs and to be free from internal duplications were trimmed of their variable tails (insertion of SINE elements results in incomparable sequences within this region), aligned with MAFFT v7.271 [65, 66] and used to build an ML tree with a GTR+CAT model using FastTree v2.1.11 [67] with 1000-replicate bootstrapping. Figure graphing was with FigTree v1.4.4 (tree.bio.ed.ac.uk/software/figtree). Selection analyses were conducted using the HyPhy suite v2.5.1 (FUBAR and MEME algorithms) according to published best practices and significance thresholds recommended in the user manual. Residue resampling was assessed within UGENE [68] using its ability to link the display of alignments and their trees, allowing for visualization of repeated reoccurrence of residues across separate branches.

### Analysis of Fv1 and Fv7 expression with RNAseq

Raw reads from published RNAseq experiments were downloaded and reads were adapter- and quality-trimmed using Trimmomatic 0.32 [69] and discarded if shorter than 30 nts. For determination of expression start sites, reads were then mapped to the mouse genome (GRCm38.78) with the splice-aware aligner HISAT2 [70]. For qualitative determination of *Fv1* and *Fv7* expression in *M*. *caroli*, trimmed reads originating from *Fv1* or *Fv7* were recruited using bbduk (BBTools, jgi.doe.gov/data-and-tools/bbtools/) and aligned instead to the sequences of Fv1CAR1 and Fv7CAR1. Consensus sequences were formed from the pileups of uniquely-aligning reads within UGENE [68] and multiple sequence alignments produced with MAFFT v7.271 [65, 66]. The alignment was inspected to compile positions discriminating the derived consensus sequences (S4 Fig).

### Analysis of Fv1 transcription

pGL4.10 (Promega) plasmids were produced with synthesized DNAs representing the region from 150 to 350 nucleotides 5’ of the *Fv1* ATG. Mutated constructs were produced for the putative initiator elements by replacing the sequences with adenine. These, and the control SV40-driven pGL4.13, were introduced to MDTF cells with GeneJuice (Merck) for harvest after 24 hours. 5x10^4^ cells were re-suspended in phenol-free media, mixed with Bright-Glo luciferin (Promega) and assayed according to the manufacturer’s instructions using opaque-walled black 96 well plates. Separate experiments were assayed with triplicate technical repeats. Constructs tested for *Fv1* and *Fv7* promoter activity for *M*. *caroli* were cloned using the Ch4Fv1 or Chr6Fv7 primers (see *Fv1* and *Fv7* cloning) alongside Fv1PRev (5’ CTTCAGACTTTTGTTTTCCCTAG 3’) and Fv7PRev (5’ CTTCAGATTTTTGTTTCCCTAGAAC 3’), respectively. Testing was conducted as above with MDTF, as well as with the murine RAW264.7 and human 293T cell lines.

### Prediction of INR elements

The sequence preceding the *Fv1* CDS was scanned with a predefined PWM [46] using inbuilt functionality within UGENE [68].

## Supporting information

S1 Fig Some examples of duplication or deletion within *Fv1* and *Fv7*. (Top) Alignment of the amino acid sequences of Fv1CER1 and Fv1CER3 showing a 3 residue / 9 nt duplication (green) of the adjacent target sequence (yellow). (Middle) Alignment of the amino acid sequences of Fv1CER1 and Fv1CER20 showing a 56 residue / 168 nt duplication (green) of the adjacent target sequence (yellow). (Bottom) Alignment of the amino acid sequence at the C-terminus of Fv7CER1 and Fv7CER26 showing the extension of the C-terminus of Fv7CER26 due to frameshifting following a deletion of 4 nucleotides. The alternative sequence caused by the frameshift is shown in yellow.
(TIF)

S2 Fig B1 truncation of the Fv1 C terminal region. The Fv1 C terminal region from *M*. *caroli*, *M*. *cookii*, *M*. *cervicolor*, and *M*. *fragilicauda* in comparison to *Fv1*^*b*^. Sequences deriving from B1 repeats are highlighted in red. Indel variation between sequences within each species are indicated by blue arrows and corresponding nucleotides and residues.
(TIF)

S3 Fig ML trees of *Fv1* and *Fv7* sequences. Separate ML trees produced by FastTree under a generalized time reversible model (GTR+CAT) from alignments of *Fv1* (LogL = -2389) and *Fv7* (LogL = -3220). Only nucleotide sequences with intact ORFs and without internal duplications were included and all had the variable tail removed (equivalent to truncation at Fv1^b^ residue 430) prior to alignment with MAFFT. The scale displays substitutions per site, species are separately colored, and numbering details the results of 1000-replicate bootstrapping.
(TIF)

S4 Fig Discrimination of RNAseq reads originating from *Fv1* and *Fv7*. Regions of alignments of consensus pileups for reads from ERP023198 and ERP005559 aligning to Fv1CAR1 and Fv7CAR1, the alleles of *Fv1* and *Fv7* found in CAROLI/EiJ, along with these two known sequences for reference. Regions are centered around bases that discriminate *Fv1* from *Fv7*. Due to the low coverage, not all areas of the genes are covered by consensus pileups, as indicated by alignment gaps (‘–’).
(TIF)

S5 Fig Mapping specificity residues: Fv1CAR1. Residues differing between the restricting and non-restricting variants in the C-terminal region are shown on the left while restriction data are presented on the right of the figure. These variants were introduced into permissive MDTF cells using a retroviral vector also containing the EYFP marker and challenged with EGFP-carrying virus to allow calculation of restriction capacity. Values are the means and standard deviations of at least 4 experiments.
(TIF)

S6 Fig Mapping specificity residues: Fv1COO4. Residues differing between the restricting and non-restricting variants in the C-terminal region are shown on the left while restriction data are presented on the right of the figure. These variants were introduced into permissive MDTF cells using a retroviral vector also containing the EYFP marker and challenged with EGFP-carrying virus to allow calculation of restriction capacity. Values are the means and standard deviations of at least 4 experiments.
(TIF)

S7 Fig Mapping specificity residues: Fv7CAR1. Residues differing between the restricting and non-restricting variants in the C-terminal region are shown on the left while restriction data are presented on the right of the figure. These variants were introduced into permissive MDTF cells using a retroviral vector also containing the EYFP marker and challenged with EGFP-carrying virus to allow calculation of restriction capacity. Values are the means and standard deviations of at least 4 experiments.
(TIF)

S8 Fig FACS profiles of a 3-color flow cytometry assay for measuring restriction. A pseudocolour plot of the mScarlet (Fv1 positive) vs YFP (Fv7 positive) populations is shown in the center. Each quadrant of this plot was gated and the GFP (infected) population measured as displayed around the periphery.
(TIF)

S9 Fig ML tree of COI gene sequences for sampled mice. ML tree produced by FastTree (LogL under a generalized time reversible model (GTR+CAT) = -1,646, scale as substitutions per site) from a MAFFT alignment of COI nucleotide sequences for the mice described in Table 1. Branches are colored according to species and nodes according to the location of sample collection. Numbering details the results of 1000-replicate bootstrapping.
(TIF)

S1 Table Alleles of *Fv1* and *Fv7*. Individual listing of the alleles reported and their accessions. Included is the length of the C terminal variable tail, details of any inactivating mutations, and of the presence of B1 SINEs.
(XLSX)

S2 Table HyPhy selection analyses. Listing of the alignment sites determined to be positively selected using FUBAR and MEME, along with the confidence values for each. For each, an assessment of whether selection results in repeated resampling of residues across different branches of the tree is included.
(XLSX)

S1 Text COI gene sequences determined and used for determination of species. (DOCX)

S2 Text All *Fv1* and *Fv7* gene sequences used and determined in this study. (DOCX)
