# Supplementary figures and images for: Duplication and divergence of the retrovirus restriction gene Fv1 in Mus caroli allows protection from multiple retroviruses
Source: PLoS Genet. 2020 Jun 11;16(6):e1008471. doi: 10.1371/journal.pgen.1008471 (PMC7313476; doi:10.1371/journal.pgen.1008471)

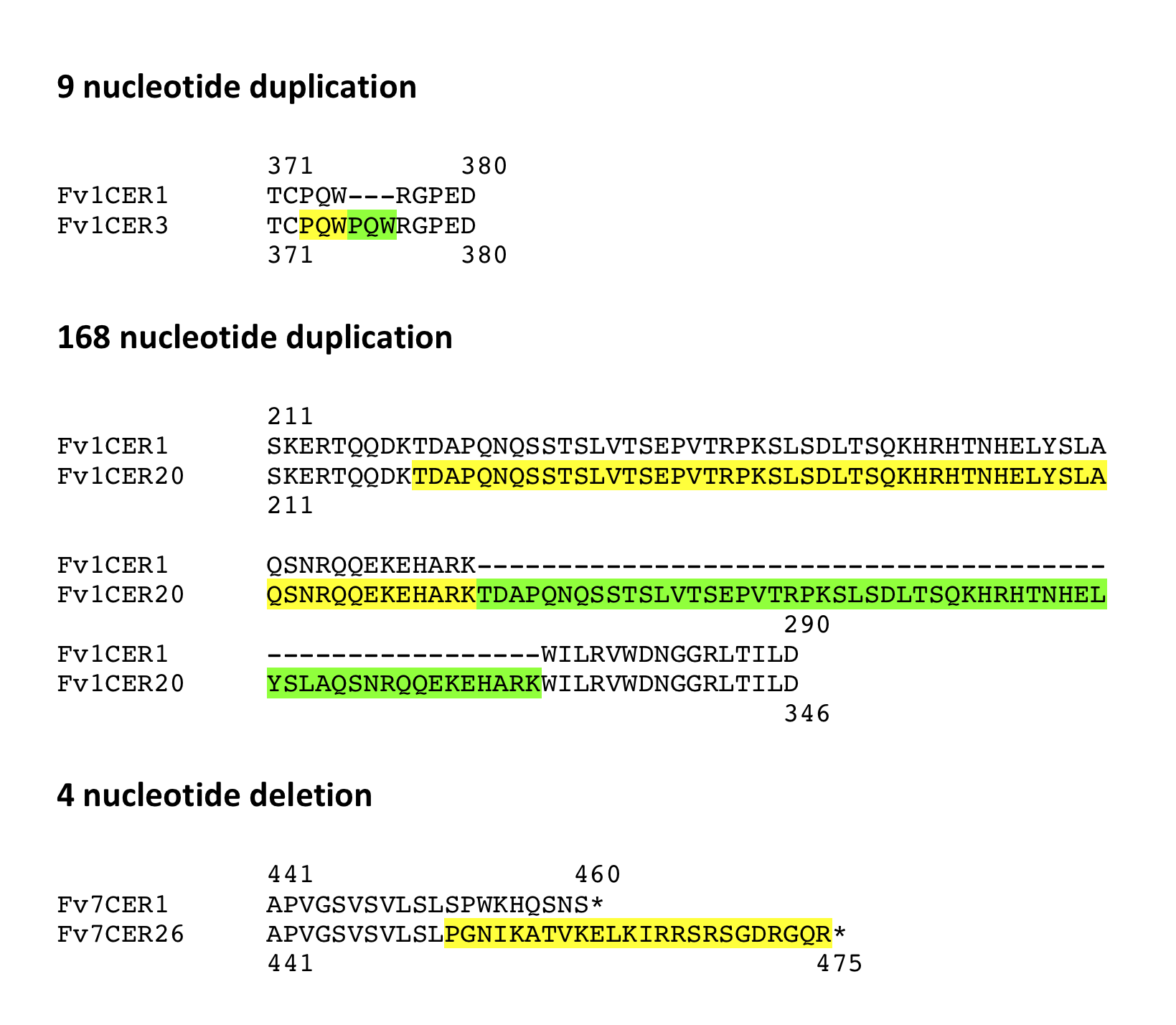

Supplement: S1 Fig — (Top) Alignment of the amino acid sequences of Fv1CER1 and Fv1CER3 showing a 3 residue / 9 nt duplication (green) of the adjacent target sequence (yellow). (Middle) Alignment of the amino acid sequences of Fv1CER1 and Fv1CER20 showing a 56 residue / 168 nt duplication (green) of the adjacent target sequence (yellow). (Bottom) Alignment of the amino acid sequence at the C-terminus of Fv7CER1 and Fv7CER26 showing the extension of the C-terminus of Fv7CER26 due to frameshifting following a deletion of 4 nucleotides. The alternative sequence caused by the frameshift is shown in yellow. (TIF) [file pgen.1008471.s001.tif]

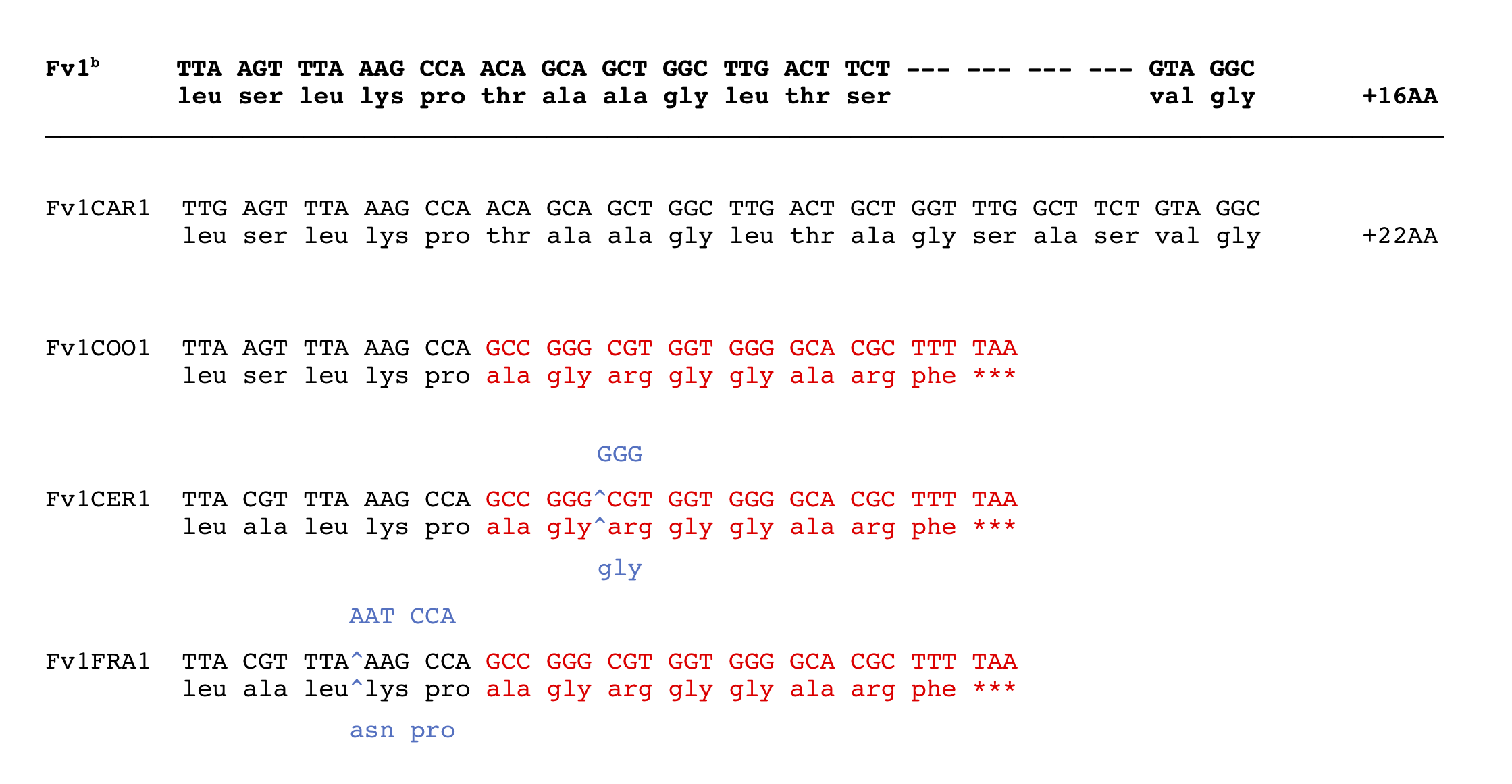

Supplement: S2 Fig — The Fv1 C terminal region from M. caroli, M. cookii, M. cervicolor, and M. fragilicauda in comparison to Fv1b. Sequences deriving from B1 repeats are highlighted in red. Indel variation between sequences within each species are indicated by blue arrows and corresponding nucleotides and residues. (TIF) [file pgen.1008471.s002.tif]

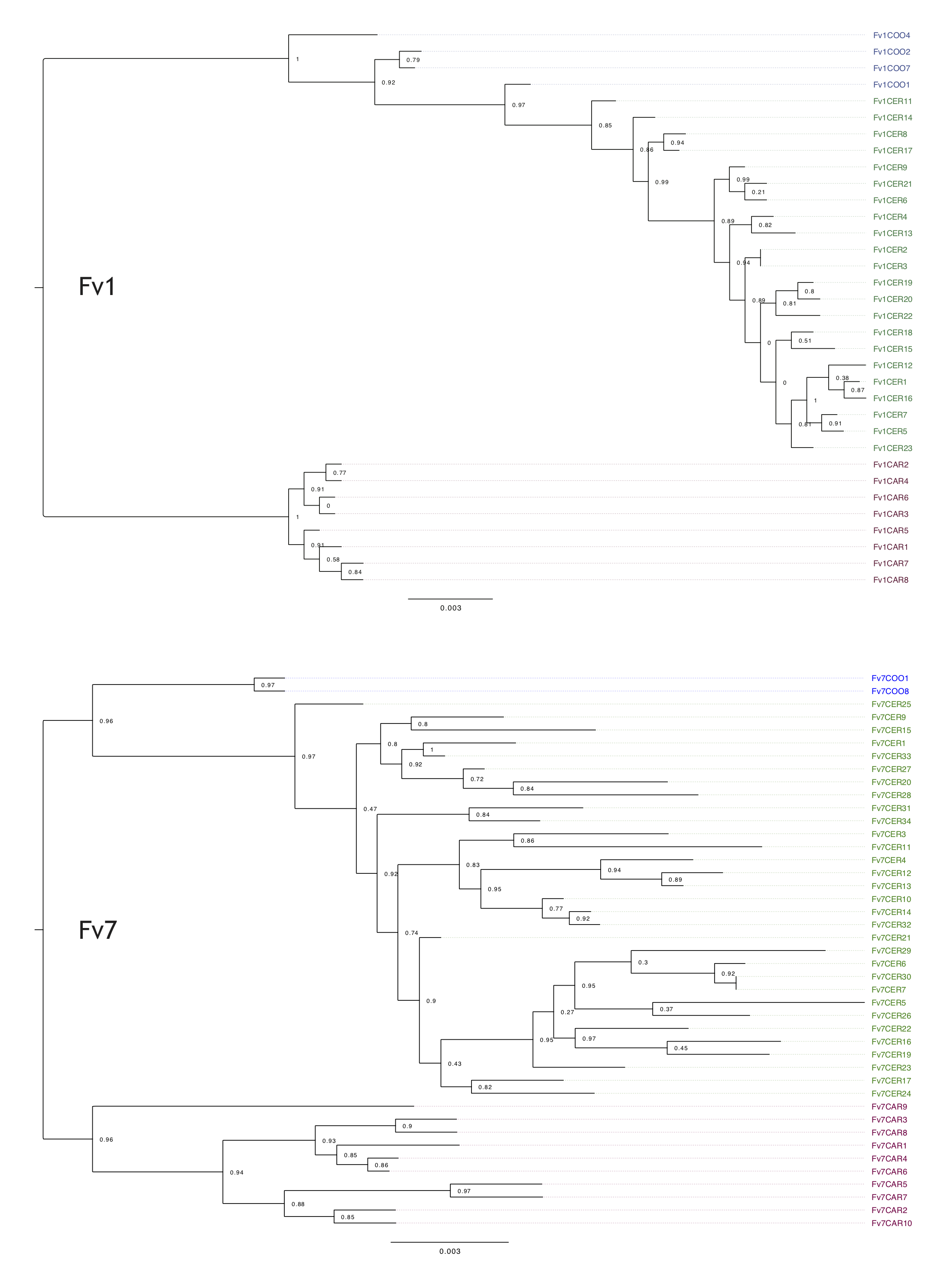

Supplement: S3 Fig — Separate ML trees produced by FastTree under a generalized time reversible model (GTR+CAT) from alignments of Fv1 (LogL = -2389) and Fv7 (LogL = -3220). Only nucleotide sequences with intact ORFs and without internal duplications were included and all had the variable tail removed (equivalent to truncation at Fv1b residue 430) prior to alignment with MAFFT. The scale displays substitutions per site, species are separately colored, and numbering details the results of 1000-replicate bootstrapping. (TIF) [file pgen.1008471.s003.tif]

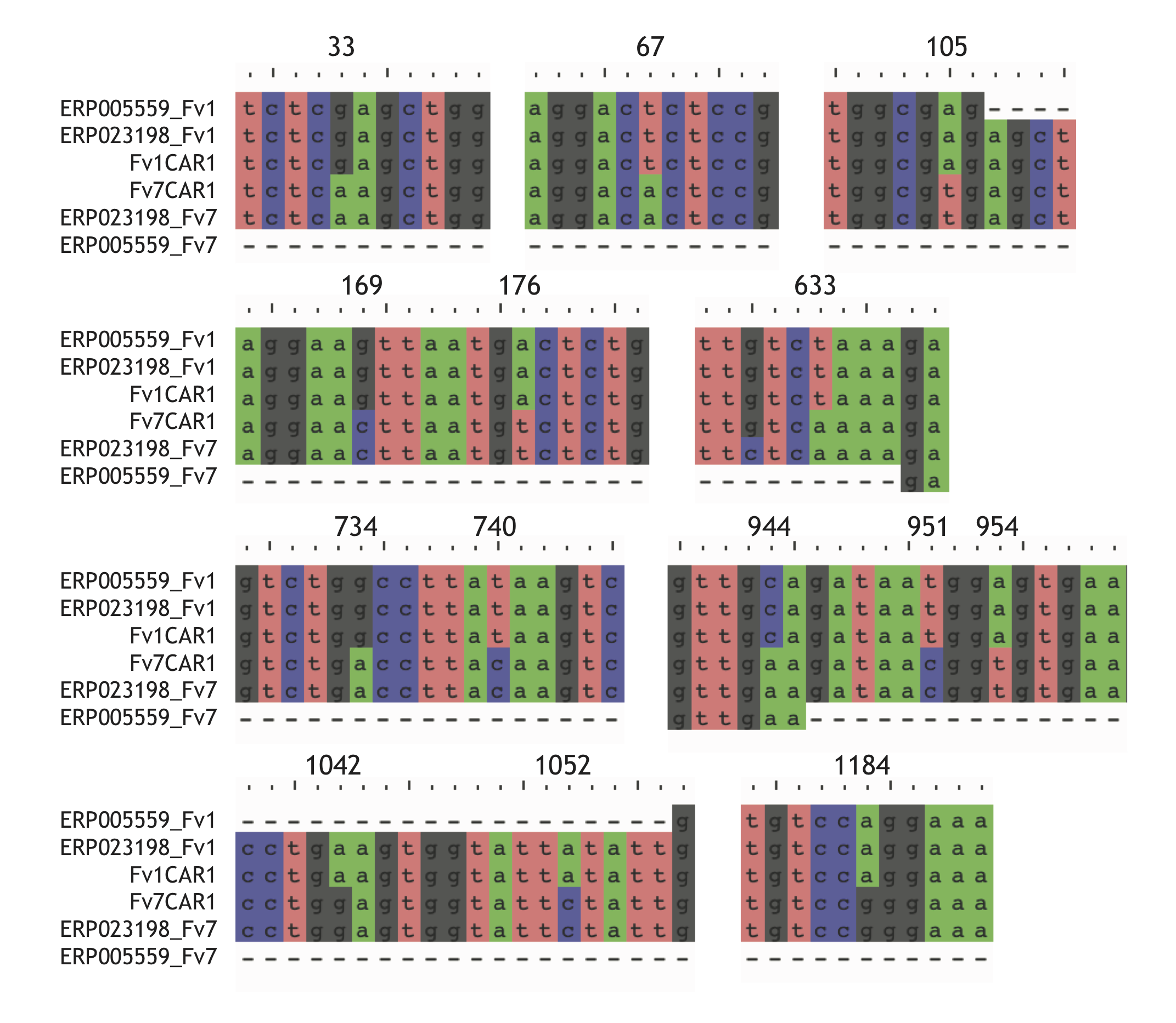

Supplement: S4 Fig — Regions of alignments of consensus pileups for reads from ERP023198 and ERP005559 aligning to Fv1CAR1 and Fv7CAR1, the alleles of Fv1 and Fv7 found in CAROLI/EiJ, along with these two known sequences for reference. Regions are centered around bases that discriminate Fv1 from Fv7. Due to the low coverage, not all areas of the genes are covered by consensus pileups, as indicated by alignment gaps (‘–’). (TIF) [file pgen.1008471.s004.tif]

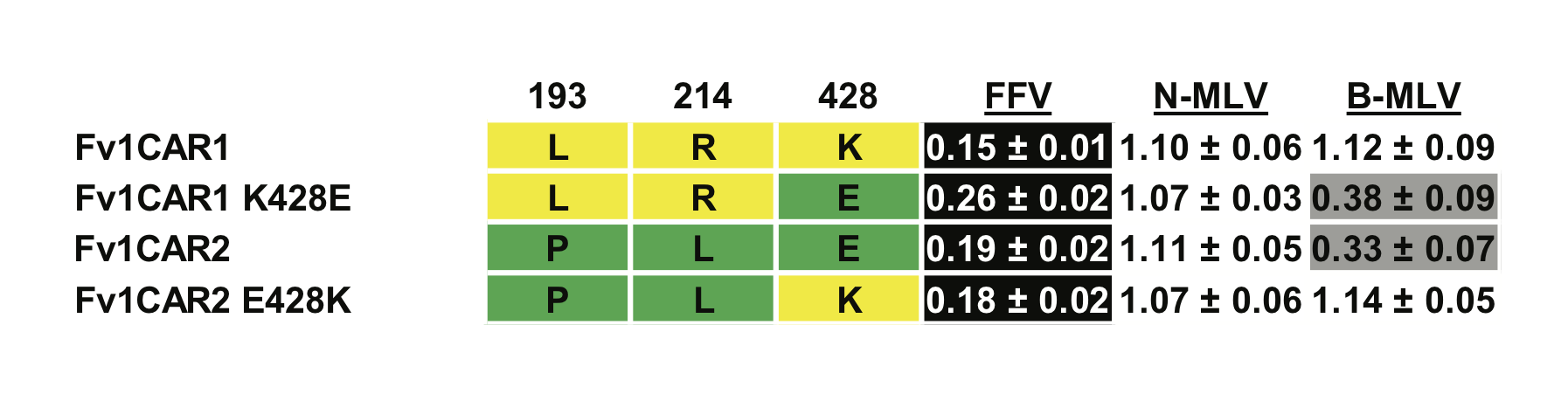

Supplement: S5 Fig — Residues differing between the restricting and non-restricting variants in the C-terminal region are shown on the left while restriction data are presented on the right of the figure. These variants were introduced into permissive MDTF cells using a retroviral vector also containing the EYFP marker and challenged with EGFP-carrying virus to allow calculation of restriction capacity. Values are the means and standard deviations of at least 4 experiments. (TIF) [file pgen.1008471.s005.tif]

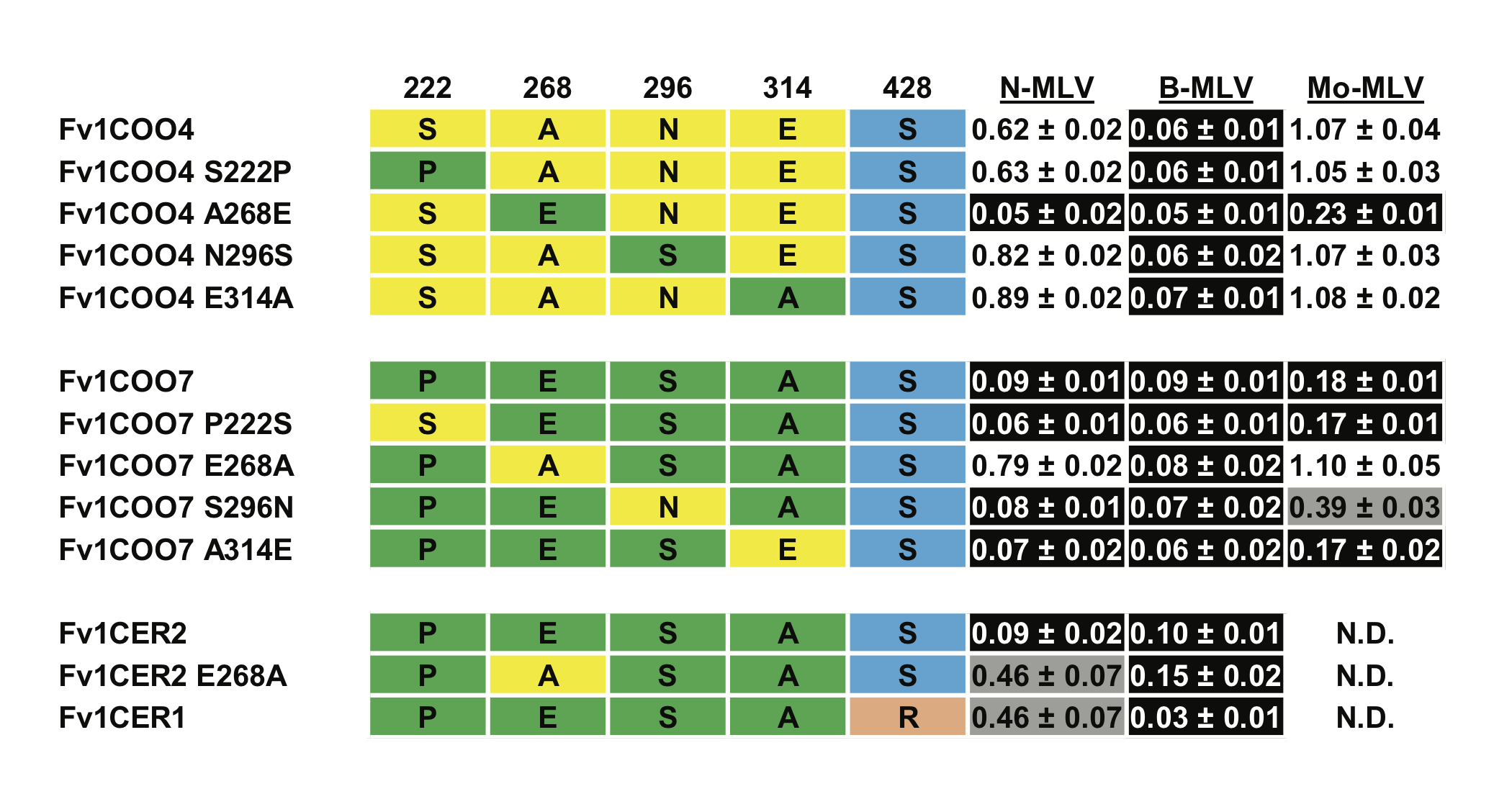

Supplement: S6 Fig — Residues differing between the restricting and non-restricting variants in the C-terminal region are shown on the left while restriction data are presented on the right of the figure. These variants were introduced into permissive MDTF cells using a retroviral vector also containing the EYFP marker and challenged with EGFP-carrying virus to allow calculation of restriction capacity. Values are the means and standard deviations of at least 4 experiments. (TIF) [file pgen.1008471.s006.tif]

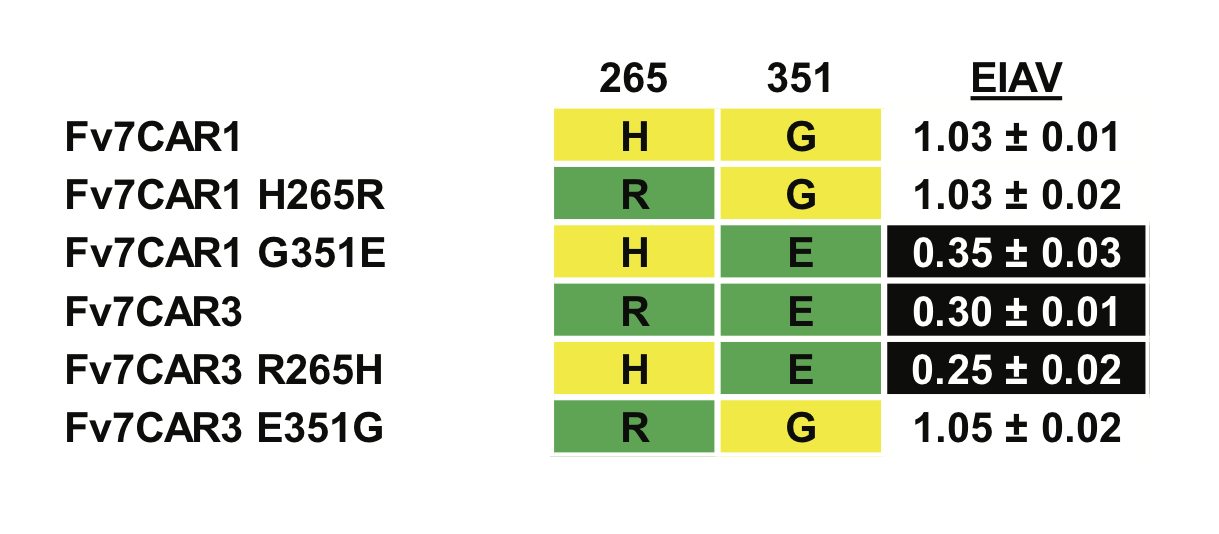

Supplement: S7 Fig — Residues differing between the restricting and non-restricting variants in the C-terminal region are shown on the left while restriction data are presented on the right of the figure. These variants were introduced into permissive MDTF cells using a retroviral vector also containing the EYFP marker and challenged with EGFP-carrying virus to allow calculation of restriction capacity. Values are the means and standard deviations of at least 4 experiments. (TIF) [file pgen.1008471.s007.tif]

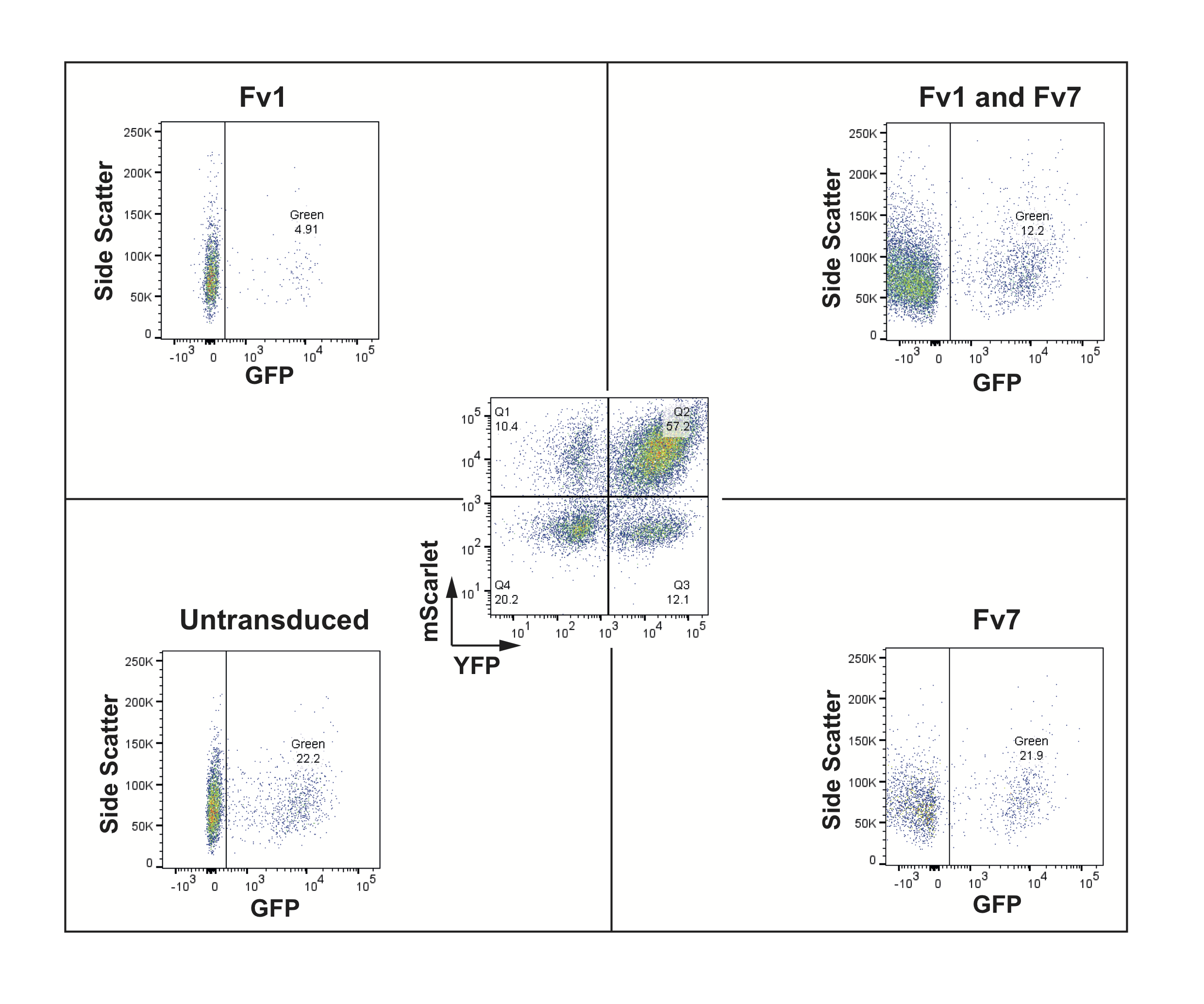

Supplement: S8 Fig — A pseudocolour plot of the mScarlet (Fv1 positive) vs YFP (Fv7 positive) populations is shown in the center. Each quadrant of this plot was gated and the GFP (infected) population measured as displayed around the periphery. (TIF) [file pgen.1008471.s008.tif]

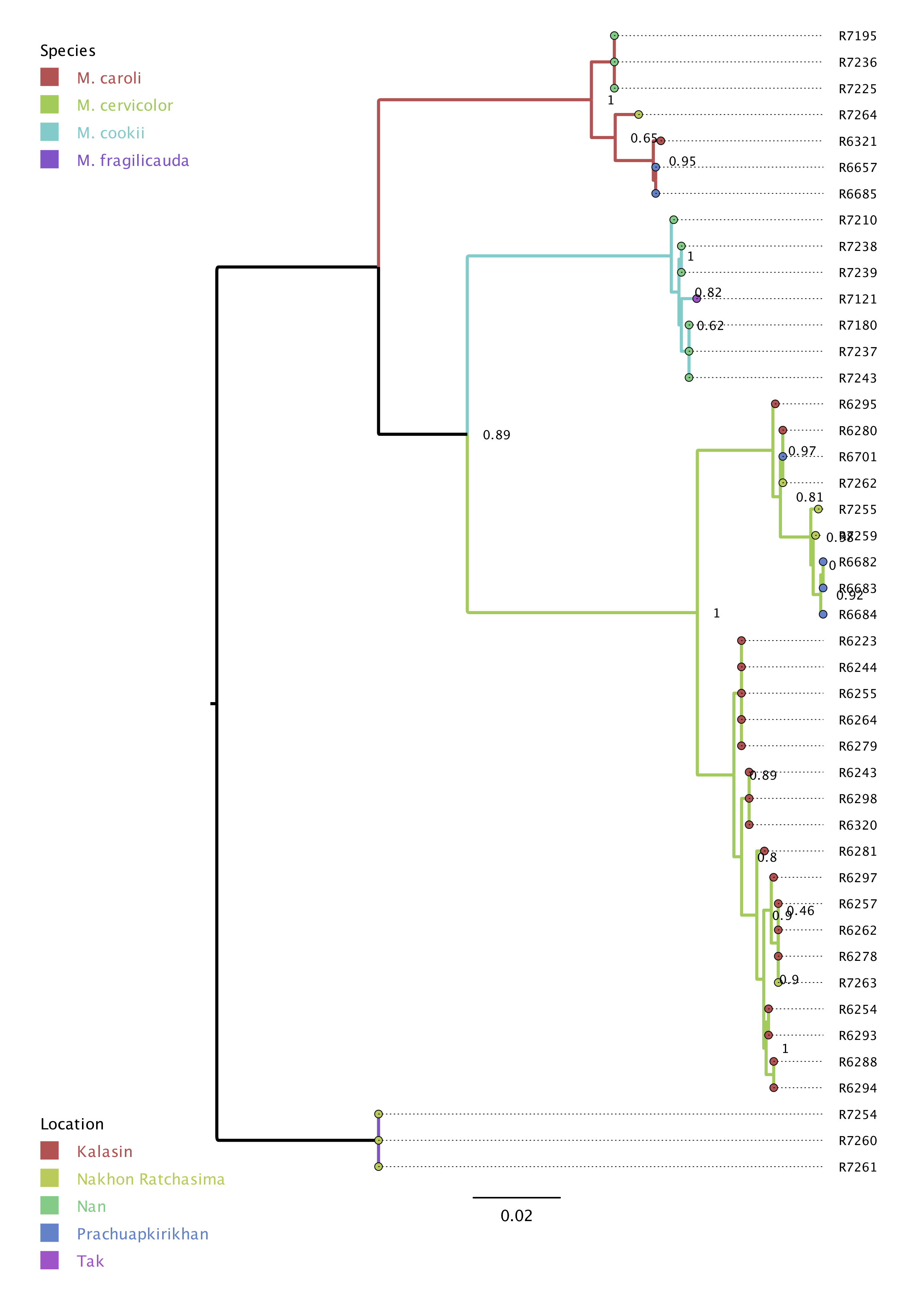

Supplement: S9 Fig — ML tree produced by FastTree (LogL under a generalized time reversible model (GTR+CAT) = -1,646, scale as substitutions per site) from a MAFFT alignment of COI nucleotide sequences for the mice described in Table 1. Branches are colored according to species and nodes according to the location of sample collection. Numbering details the results of 1000-replicate bootstrapping. (TIF) [file pgen.1008471.s009.tif]
