# Supplementary material for: Duplication and divergence of the retrovirus restriction gene Fv1 in Mus caroli allows protection from multiple retroviruses
Source: PLoS Genet. 2020 Jun 11;16(6):e1008471. doi: 10.1371/journal.pgen.1008471 (PMC7313476; doi:10.1371/journal.pgen.1008471)
Supplement: S1 Text — (DOCX) [file pgen.1008471.s012.docx]

>R6223

ggtgcttgagcaggaatagtaggaactgcattaagtatcttaattcgagcagaattaggacaaccaggtgcgctcctaggagatgaccagatttacaatgttattgttactgcccatgcatttgttataatctttttcatagtaataccaataataattggaggtttcggaaactgacttgtacccctaataatcggagccccagatatagcatttccccgaataaataatataagcttttgattacttcccccatcattcctccttctcctagcatcatcaatagttgaagctggagcaggaacaggatgaacagtttacccacctctagctggaaatttagcccatgcaggagcatctgtagacctaacaattttttcccttcacttagctggagtatcatccatcctaggtgctatcaactttatcacaacaattattaatataaaacctccagctataactcaatatcaaaccccactattcgtctgatctgtgctaattacagctgttttactcttactgtcacttccagtattagctgcaggaattactatactattaacagaccgaaacctaaatacaactttctttgaccctgc

>R6244

ggtgcttgagcaggaatagtaggaactgcattaagtatcttaattcgagcagaattaggacaaccaggtgcgctcctaggagatgaccagatttacaatgttattgttactgcccatgcatttgttataatctttttcatagtaataccaataataattggaggtttcggaaactgacttgtacccctaataatcggagccccagatatagcatttccccgaataaataatataagcttttgattacttcccccatcattcctccttctcctagcatcatcaatagttgaagctggagcaggaacaggatgaacagtttacccacctctagctggaaatttagcccatgcaggagcatctgtagacctaacaattttttcccttcacttagctggagtatcatccatcctaggtgctatcaactttatcacaacaattattaatataaaacctccagctataactcaatatcaaaccccactattcgtctgatctgtgctaattacagctgttttactcttactgtcacttccagtattagctgcaggaattactatactattaacagaccgaaacctaaatacaactttctttgaccctgc

>R6255

ggtgcttgagcaggaatagtaggaactgcattaagtatcttaattcgagcagaattaggacaaccaggtgcgctcctaggagatgaccagatttacaatgttattgttactgcccatgcatttgttataatctttttcatagtaataccaataataattggaggtttcggaaactgacttgtacccctaataatcggagccccagatatagcatttccccgaataaataatataagcttttgattacttcccccatcattcctccttctcctagcatcatcaatagttgaagctggagcaggaacaggatgaacagtttacccacctctagctggaaatttagcccatgcaggagcatctgtagacctaacaattttttcccttcacttagctggagtatcatccatcctaggtgctatcaactttatcacaacaattattaatataaaacctccagctataactcaatatcaaaccccactattcgtctgatctgtgctaattacagctgttttactcttactgtcacttccagtattagctgcaggaattactatactattaacagaccgaaacctaaatacaactttctttgaccctgc

>R6264

ggtgcttgagcaggaatagtaggaactgcattaagtatcttaattcgagcagaattaggacaaccaggtgcgctcctaggagatgaccagatttacaatgttattgttactgcccatgcatttgttataatctttttcatagtaataccaataataattggaggtttcggaaactgacttgtacccctaataatcggagccccagatatagcatttccccgaataaataatataagcttttgattacttcccccatcattcctccttctcctagcatcatcaatagttgaagctggagcaggaacaggatgaacagtttacccacctctagctggaaatttagcccatgcaggagcatctgtagacctaacaattttttcccttcacttagctggagtatcatccatcctaggtgctatcaactttatcacaacaattattaatataaaacctccagctataactcaatatcaaaccccactattcgtctgatctgtgctaattacagctgttttactcttactgtcacttccagtattagctgcaggaattactatactattaacagaccgaaacctaaatacaactttctttgaccctgc

>R6279

ggtgcttgagcaggaatagtaggaactgcattaagtatcttaattcgagcagaattaggacaaccaggtgcgctcctaggagatgaccagatttacaatgttattgttactgcccatgcatttgttataatctttttcatagtaataccaataataattggaggtttcggaaactgacttgtacccctaataatcggagccccagatatagcatttccccgaataaataatataagcttttgattacttcccccatcattcctccttctcctagcatcatcaatagttgaagctggagcaggaacaggatgaacagtttacccacctctagctggaaatttagcccatgcaggagcatctgtagacctaacaattttttcccttcacttagctggagtatcatccatcctaggtgctatcaactttatcacaacaattattaatataaaacctccagctataactcaatatcaaaccccactattcgtctgatctgtgctaattacagctgttttactcttactgtcacttccagtattagctgcaggaattactatactattaacagaccgaaacctaaatacaactttctttgaccctgc

>R6243

ggtgcttgagcaggaatagtaggaactgcattaagtattttaattcgagcagaattaggacaaccaggtgcgctcctaggagatgaccagatttacaatgttattgttactgcccatgcatttgttataatctttttcatagttataccaataataattggaggtttcggaaactgacttgtacccctaataatcggagccccagatatagcatttccccgaataaataatataagcttttgattacttcccccatcattcctccttctcctagcatcatcaatagttgaagctggagcaggaacaggatgaacagtttacccacctctagctggaaatttagcccatgcaggagcatctgtagacctgacaattttttcccttcacttagctggagtatcatccatcctaggtgctatcaactttatcacaacaattattaatataaaacctccagctataactcaatatcaaaccccactattcgtctgatctgtgctaattacagctgttttactcttactgtcacttccagtattagctgcaggaattactatactattaacagaccgaaacctaaatacaactttctttgaccctgc

>R6298

ggtgcttgagcaggaatagtaggaactgcattaagtattttaattcgagcagaattaggacaaccaggtgcgctcctaggagatgaccagatttacaatgttattgttactgcccatgcatttgttataatctttttcatagttataccaataataattggaggtttcggaaactgacttgtacccctaataatcggagccccagatatagcatttccccgaataaataatataagcttttgattacttcccccatcattcctccttctcctagcatcatcaatagttgaagctggagcaggaacaggatgaacagtttacccacctctagctggaaatttagcccatgcaggagcatctgtagacctgacaattttttcccttcacttagctggagtatcatccatcctaggtgctatcaactttatcacaacaattattaatataaaacctccagctataactcaatatcaaaccccactattcgtctgatctgtgctaattacagctgttttactcttactgtcacttccagtattagctgcaggaattactatactattaacagaccgaaacctaaatacaactttctttgaccctgc

>R6320

ggtgcttgagcaggaatagtaggaactgcattaagtattttaattcgagcagaattaggacaaccaggtgcgctcctaggagatgaccagatttacaatgttattgttactgcccatgcatttgttataatctttttcatagttataccaataataattggaggtttcggaaactgacttgtacccctaataatcggagccccagatatagcatttccccgaataaataatataagcttttgattacttcccccatcattcctccttctcctagcatcatcaatagttgaagctggagcaggaacaggatgaacagtttacccacctctagctggaaatttagcccatgcaggagcatctgtagacctgacaattttttcccttcacttagctggagtatcatccatcctaggtgctatcaactttatcacaacaattattaatataaaacctccagctataactcaatatcaaaccccactattcgtctgatctgtgctaattacagctgttttactcttactgtcacttccagtattagctgcaggaattactatactattaacagaccgaaacctaaatacaactttctttgaccctgc

>R6254

ggtgcttgagcaggaatagtaggaactgcattaagtattttaattcgagcagaattaggacaaccaggtgcgctcctaggagatgaccagatttacaatgttattgttactgcccatgcatttgttataatctttttcatagttataccaataataattggaggtttcggaaactgacttgtacccctaataatcggagccccagatatagcattcccccgaataaataatataagcttttgattacttcccccatcattcctccttctcctagcatcatcaatagttgaagctggagcaggaacaggatgaacagtttatccacctctagctggaaatttagcccatgcaggagcatctgtagacctaacaattttttcccttcacttagctggagtttcatccatcctaggtgctatcaactttatcacaacaattattaatataaaacctccagctataactcaatatcaaaccccactattcgtctgatctgtgctaattacagctgttttactcttactgtcacttccagtattagctgcaggaattactatactattaacagaccgaaacctaaatacaactttctttgaccctgc

>R6293

ggtgcttgagcaggaatagtaggaactgcattaagtattttaattcgagcagaattaggacaaccaggtgcgctcctaggagatgaccagatttacaatgttattgttactgcccatgcatttgttataatctttttcatagttataccaataataattggaggtttcggaaactgacttgtacccctaataatcggagccccagatatagcattcccccgaataaataatataagcttttgattacttcccccatcattcctccttctcctagcatcatcaatagttgaagctggagcaggaacaggatgaacagtttatccacctctagctggaaatttagcccatgcaggagcatctgtagacctaacaattttttcccttcacttagctggagtttcatccatcctaggtgctatcaactttatcacaacaattattaatataaaacctccagctataactcaatatcaaaccccactattcgtctgatctgtgctaattacagctgttttactcttactgtcacttccagtattagctgcaggaattactatactattaacagaccgaaacctaaatacaactttctttgaccctgc

>R6288

ggtgcttgagcaggaatagtaggaactgcattaagtattttaattcgagcagaattaggacaaccaggtgcgctcctaggagatgaccagatttacaatgttattgttactgcccatgcatttgttataatctttttcatagttataccaataataattggaggtttcggaaactgacttgtacccctaataatcggagccccagatatagcattcccccgaataaataatataagcttttgattacttcccccatcattcctccttctcctagcatcatcaatagttgaagctggagcaggaacaggatgaacagtttatccacctctagctggaaatttagcccatgcaggagcatctgtagacctaacaattttttcccttcacttagctggagtttcatccatcctaggtgctatcaactttatcacaacaattattaatataaaacctccagctataactcaatatcaaaccccactattcgtctgatctgtactaattacagctgttttactcttactgtcacttccagtattagctgcaggaattactatactattaacagaccgaaacctaaatacaactttctttgaccctgc

>R6294

ggtgcttgagcaggaatagtaggaactgcattaagtattttaattcgagcagaattaggacaaccaggtgcgctcctaggagatgaccagatttacaatgttattgttactgcccatgcatttgttataatctttttcatagttataccaataataattggaggtttcggaaactgacttgtacccctaataatcggagccccagatatagcattcccccgaataaataatataagcttttgattacttcccccatcattcctccttctcctagcatcatcaatagttgaagctggagcaggaacaggatgaacagtttatccacctctagctggaaatttagcccatgcaggagcatctgtagacctaacaattttttcccttcacttagctggagtttcatccatcctaggtgctatcaactttatcacaacaattattaatataaaacctccagctataactcaatatcaaaccccactattcgtctgatctgtactaattacagctgttttactcttactgtcacttccagtattagctgcaggaattactatactattaacagaccgaaacctaaatacaactttctttgaccctgc

>R6297

ggtgcttgagcaggaatagtaggaactgcattaagtattttaattcgagcagaattaggacaaccaggtgcgctcctaggagatgaccagatttacaatgttattgttactgcccatgcatttgttataatctttttcatagttataccaataataattggaggtttcggaaactgacttgtgcccctaataatcggagccccagatatagcattcccccgaataaataatataagcttttgattacttcccccatcattcctccttctcctagcatcatcaatagttgaagctggagcaggaacaggatgaacagtttatccacctctagctggaaatttagcccatgcaggagcatctgtagacctaacaattttttcccttcacttagctggagtttcatccatcctaggtgctatcaactttatcacaacaattattaatataaaacctccagctataactcaatatcaaaccccactattcgtctgatctgtgctaattacagctgttttactcttactgtcacttccagtattagctgcaggaattactatactattaacagaccgaaacctaaatacaactttctttgaccctgc

>R6257

ggtgcttgagcaggaatagtaggaactgcattaagtattttaattcgagcagaattaggacaaccaggtgcgctcctaggagatgaccagatttacaatgttattgttactgcccatgcatttgttataatctttttcatagttataccaataataattggaggtttcggaaactgacttgtgcccctaataatcggagccccagatatagcatttccccgaataaataatataagcttttgattacttcccccatcattcctccttctcctagcatcatcaatagttgaagctggagcaggaacaggatgaacagtttatccacctctagctggaaatttagcccatgcaggagcatctgtagacctaacaattttttcccttcacttagctggagtttcatccatcctaggtgctatcaactttatcacaacaattattaatataaaacctccagctataactcaatatcaaaccccactattcgtctgatctgtgctaattacagctgttttactcttactgtcacttccagtattagctgcaggaattactatactattaacagaccgaaacctaaatacaactttctttgaccctgc

>R6262

ggtgcttgagcaggaatagtaggaactgcattaagtattttaattcgagcagaattaggacaaccaggtgcgctcctaggagatgaccagatttacaatgttattgttactgcccatgcatttgttataatctttttcatagttataccaataataattggaggtttcggaaactgacttgtgcccctaataatcggagccccagatatagcatttccccgaataaataatataagcttttgattacttcccccatcattcctccttctcctagcatcatcaatagttgaagctggagcaggaacaggatgaacagtttatccacctctagctggaaatttagcccatgcaggagcatctgtagacctaacaattttttcccttcacttagctggagtttcatccatcctaggtgctatcaactttatcacaacaattattaatataaaacctccagctataactcaatatcaaaccccactattcgtctgatctgtgctaattacagctgttttactcttactgtcacttccagtattagctgcaggaattactatactattaacagaccgaaacctaaatacaactttctttgaccctgc

>R6278

ggtgcttgagcaggaatagtaggaactgcattaagtattttaattcgagcagaattaggacaaccaggtgcgctcctaggagatgaccagatttacaatgttattgttactgcccatgcatttgttataatctttttcatagttataccaataataattggaggtttcggaaactgacttgtgcccctaataatcggagccccagatatagcatttccccgaataaataatataagcttttgattacttcccccatcattcctccttctcctagcatcatcaatagttgaagctggagcaggaacaggatgaacagtttatccacctctagctggaaatttagcccatgcaggagcatctgtagacctaacaattttttcccttcacttagctggagtttcatccatcctaggtgctatcaactttatcacaacaattattaatataaaacctccagctataactcaatatcaaaccccactattcgtctgatctgtgctaattacagctgttttactcttactgtcacttccagtattagctgcaggaattactatactattaacagaccgaaacctaaatacaactttctttgaccctgc

>R7263

ggtgcttgagcaggaatagtaggaactgcattaagtattttaattcgagcagaattaggacaaccaggtgcgctcctaggagatgaccagatttacaatgttattgttactgcccatgcatttgttataatctttttcatagttataccaataataattggaggtttcggaaactgacttgtgcccctaataatcggagccccagatatagcatttccccgaataaataatataagcttttgattacttcccccatcattcctccttctcctagcatcatcaatagttgaagctggagcaggaacaggatgaacagtttatccacctctagctggaaatttagcccatgcaggagcatctgtagacctaacaattttttcccttcacttagctggagtttcatccatcctaggtgctatcaactttatcacaacaattattaatataaaacctccagctataactcaatatcaaaccccactattcgtctgatctgtgctaattacagctgttttactcttactgtcacttccagtattagctgcaggaattactatactattaacagaccgaaacctaaatacaactttctttgaccctgc

>R6281

ggtgcttgagcaggaatagtaggaactgcattaagtattttaattcgagcagaattaggacaaccaggtgcgctcctaggagatgaccagatttacaatgttattgttactgcccatgcatttgttataatctttttcatagttataccaataataattggaggtttcggaaactgacttgtacccctaataatcggagccccagatatagcatttccccgaataaataatataagcttttgattacttcccccatcattcctccttctcctagcatcatcaatagttgaagctggagcaggaacaggatgaacagtttatccacctctagctggaaatttagcccatgcaggagcatctgtagacctaacaattttttcccttcacttagctggagtttcatccatcctaggtgccatcaactttatcacaacaattattaatataaaacctccagctataactcaatatcaaaccccactattcgtctgatctgtgctaattacagctgttttactcttactgtcacttccagtattagctgcaggaattactatactattaacagaccgaaacctaaatacaactttctttgaccctgc

>R6280

ggtgcttgagcaggaatagtaggaaccgcattaagtattttaattcgagcagaattaggacaaccaggtgcgctcctaggagatgaccagatttacaatgttattgttactgcccatgcatttgttataatctttttcatagtaataccgataataattggaggtttcggaaactgacttgtacccctaataatcggagccccagatatagcatttccccgaataaataatataagcttttgattacttcccccatcattcctccttctcttagcatcatcaatagttgaagctggagcagggacaggatgaacagtctatccacctctagctggaaatttagcccacgcaggagcatctgtagatctaacaattttttcccttcacttagctggggtatcatccatcctaggtgctatcaactttatcacaacaattattaatataaaacctccagccataactcaatatcaaaccccactatttgtctgatctgtactaattacagctgttttactcttactctcacttccagtattagctgcaggaatcactatactattaacagaccgaaacctaaatacaaccttctttgaccctgc

>R6701

ggtgcttgagcaggaatagtaggaaccgcattaagtattttaattcgagcagaattaggacaaccaggtgcgctcctaggagatgaccagatttacaatgttattgttactgcccatgcatttgttataatctttttcatagtaataccgataataattggaggtttcggaaactgacttgtacccctaataatcggagccccagatatagcatttccccgaataaataatataagcttttgattacttcccccatcattcctccttctcttagcatcatcaatagttgaagctggagcagggacaggatgaacagtctatccacctctagctggaaatttagcccacgcaggagcatctgtagatctaacaattttttcccttcacttagctggggtatcatccatcctaggtgctatcaactttatcacaacaattattaatataaaacctccagccataactcaatatcaaaccccactatttgtctgatctgtactaattacagctgttttactcttactctcacttccagtattagctgcaggaatcactatactattaacagaccgaaacctaaatacaaccttctttgaccctgc

>R7262

ggtgcttgagcaggaatagtaggaaccgcattaagtattttaattcgagcagaattaggacaaccaggtgcgctcctaggagatgaccagatttacaatgttattgttactgcccatgcatttgttataatctttttcatagtaataccgataataattggaggtttcggaaactgacttgtacccctaataatcggagccccagatatagcatttccccgaataaataatataagcttttgattacttcccccatcattcctccttctcttagcatcatcaatagttgaagctggagcagggacaggatgaacagtctatccacctctagctggaaatttagcccacgcaggagcatctgtagatctaacaattttttcccttcacttagctggggtatcatccatcctaggtgctatcaactttatcacaacaattattaatataaaacctccagccataactcaatatcaaaccccactatttgtctgatctgtactaattacagctgttttactcttactctcacttccagtattagctgcaggaatcactatactattaacagaccgaaacctaaatacaaccttctttgaccctgc

>R6295

ggtgcttgagcaggaatagtaggaaccgcattaagtattttaattcgagcagaattaggacaaccaggtgcgctcctaggagatgaccagatttacaatgttattgttactgcccatgcatttgttataatctttttcatagtaataccaataataattggaggtttcggaaactgacttgtacccctaataatcggagccccagatatagcatttccccgaataaataatataagcttttgattacttcccccatcattcctccttctcttagcatcatcaatagttgaagctggagcagggacaggatgaacagtctatccacctctagctggaaatttagcccacgcaggagcatctgtagatctaacaattttttcccttcacttagctggggtatcatccatcctaggtgctatcaactttatcacaacaattattaatataaaacctccagccataactcaatatcaaaccccactatttgtctgatctgtactaattacagctgttttactcttactctcacttccagtattagctgcaggaatcactatactattaacagaccgaaacctaaatacaaccttctttgaccctgc

>R7255

ggtgcttgagcaggaatagtaggaaccgcattaagtattttaattcgagcagaattaggacaaccaggtgcgctcctaggagatgaccagatttacaatgttattgttactgcccatgcatttgttataatctttttcatagtaataccgataataattggaggtttcggaaactgacttgtacccctaataatcggagccccagatatagcgtttccccgaataaataatataagcttttgattacttcccccatcattcctccttctcttagcatcatcaatagttgaagctggggcagggacaggatgaacagtctatccacctctagctggaaacttagcccacgcaggagcatctgtagatctaacaattttttcccttcacttagctggggtatcatccatcctaggtgctattaactttattacaacaattattaatataaaacctccagccataactcaatatcaaaccccactatttgtctgatctgtactaattacagctgttttactcttactctcacttccagtattagctgcaggaatcactatactattaacagaccgaaacctaaatacaaccttctttgaccctgc

>R7259

ggtgcttgagcaggaatagtaggaaccgcattaagtattttaattcgagcagaattaggacaaccaggtgcgctcctaggagatgaccagatttacaatgttattgttactgcccatgcatttgttataatctttttcatagtaataccgataataattggaggtttcggaaactgacttgtacccctaataatcggagccccagatatagcgtttccccgaataaataatataagcttttgattacttcccccatcattcctccttctcttagcatcatcaatagttgaagctggggcagggacaggatgaacagtctatccacctctagctggaaacttagcccacgcaggagcatctgtagatctaacaattttttcccttcacttagctggggtatcatccatcctaggtgctatcaactttattacaacaattattaatataaaacctccagccataactcaatatcaaaccccactatttgtctgatctgtactaattacagctgttttactcttactctcacttccagtattagctgcaggaatcactatactattaacagaccgaaacctaaatacaaccttctttgaccctgc

>R6682

ggtgcttgagcaggaatagtaggaaccgcattaagtattttaattcgagcagaattaggacaaccaggtgcgctcctaggagatgaccagatttacaatgttattgttactgcccatgcatttgttataatctttttcatagtaataccgataataattggaggtttcggaaactgacttgtacccctaataatcggagccccagatatagcgtttccccgaataaataatataagcttttgattacttcctccatcattcctccttctcttagcatcatcaatagttgaagctggggcagggacaggatgaacagtctatccacctctagctggaaacttagcccacgcaggagcatctgtagatctaacaattttttcccttcacttagctggggtatcatccatcctaggtgctatcaactttattacaacaattattaatataaaacctccagccataactcaatatcaaaccccactatttgtctgatctgtactaattacagctgttttactcttactctcacttccagtattagctgcaggaatcactatactattaacagaccgaaacctaaatacaaccttctttgaccctgc

>R6683

ggtgcttgagcaggaatagtaggaaccgcattaagtattttaattcgagcagaattaggacaaccaggtgcgctcctaggagatgaccagatttacaatgttattgttactgcccatgcatttgttataatctttttcatagtaataccgataataattggaggtttcggaaactgacttgtacccctaataatcggagccccagatatagcgtttccccgaataaataatataagcttttgattacttcctccatcattcctccttctcttagcatcatcaatagttgaagctggggcagggacaggatgaacagtctatccacctctagctggaaacttagcccacgcaggagcatctgtagatctaacaattttttcccttcacttagctggggtatcatccatcctaggtgctatcaactttattacaacaattattaatataaaacctccagccataactcaatatcaaaccccactatttgtctgatctgtactaattacagctgttttactcttactctcacttccagtattagctgcaggaatcactatactattaacagaccgaaacctaaatacaaccttctttgaccctgc

>R6684

ggtgcttgagcaggaatagtaggaaccgcattaagtattttaattcgagcagaattaggacaaccaggtgcgctcctaggagatgaccagatttacaatgttattgttactgcccatgcatttgttataatctttttcatagtaataccgataataattggaggtttcggaaactgacttgtacccctaataatcggagccccagatatagcgtttccccgaataaataatataagcttttgattacttcctccatcattcctccttctcttagcatcatcaatagttgaagctggggcagggacaggatgaacagtctatccacctctagctggaaacttagcccacgcaggagcatctgtagatctaacaattttttcccttcacttagctggggtatcatccatcctaggtgctatcaactttattacaacaattattaatataaaacctccagccataactcaatatcaaaccccactatttgtctgatctgtactaattacagctgttttactcttactctcacttccagtat

>R7121

ggagcttgagcaggaatagtaggaactgcactaagtattttaattcgagcagaattaggacagccaggcgcactactaggagatgaccaaatttataatgttattgttaccgcccatgcatttgttataatcttttttatagtaatgccaataataatcggaggtttcggaaactgacttgtaccactaataattggagccccagatatagcattcccacgaataaataatataagtttttgactacttcccccatcatttcttctcctactagcatcatcaatagtagaagctggggcaggaacaggatgaacagtctacccacctctagccggaaatttagcccatgcaggagcatccgtagatttaacaattttttctctacatttagccggtgtctcatctattttaggtgcaatcaactttattacaacaattattaatataaaacccccagctataactcagtatcaaaccccactatttgtctgatccgtattaatcacagctgtattacttttattatcactgccggtattagctgcaggaattactatactattaacagaccgaaatcttaatacaactttctttgaccctgc

>R7180

ggagcttgagcaggaatagtaggaactgcactaagtattttaattcgagcagaattaggacagccaggcgcactactaggagatgaccaaatttataatgttattgttaccgcccatgcatttgttataatcttttttatagtaatgccaataataatcggaggtttcggaaactgacttgtaccactaataattggagccccagatatagcattcccacgaataaataatataagtttttgactacttcccccatcatttcttctcctattagcatcatcaatagtagaagctggggcaggaacaggatgaacagtctacccacctctagccggaaatttagcccatgcaggagcatctgtagatttaacaattttttctctacatttagccggtgtctcatctattttaggtgcaatcaactttattacaacaattattaatataaaacccccagctataactcagtatcaaaccccactatttgtctgatccgtattaattacagctgtattacttttattatcactgccggtattagctgcaggaattactatactattaacagaccgaaatcttaatacaactttctttgaccctgc

>R7210

ggagcttgagcaggaatagtaggaactgcactaagtattttaattcgagcagaattaggacagccaggcgcactactaggagatgaccaaatttataatgttattgttaccgcccatgcatttgttataatcttttttatagtaatgccaataataatcggaggtttcggaaactgacttgtaccactaataattggagccccagatatagcattcccacgaataaataatataagtttttgactacttcccccatcatttcttctcctactagcatcatcaatagtagaagctggggcaggaacaggatgaacagtctacccacctctagccggaaatttagcccatgcaggagcatctgtagatttaacaattttttctctacatttagccggtgtctcatctattttaggtgcaatcaactttattacaacaattattaatataaaacccccagctataactcaatatcaaaccccactatttgtctgatccgtattaattacagctgtattacttttattatcactgccggtattagctgcaggaattactatactattaacagaccgaaatcttaatacaactttctttgaccctgc

>R7237

ggagcttgagcaggaatagtaggaactgcactaagtattttaattcgagcagaattaggacagccaggcgcactactaggagatgaccaaatttataatgttattgttaccgcccatgcatttgttataatcttttttatagtaatgccaataataatcggaggtttcggaaactgacttgtaccactaataattggagccccagatatagcattcccacgaataaataatataagtttttgactacttcccccatcatttcttctcctattagcatcatcaatagtagaagctggggcaggaacaggatgaacagtctacccacctctagccggaaatttagcccatgcaggagcatctgtagatttaacaattttttctctacatttagccggtgtctcatctattttaggtgcaatcaactttattacaacaattattaatataaaacccccagctataactcagtatcaaaccccactatttgtctgatccgtattaattacagctgtattacttttattatcactgccggtattagctgcaggaattactatactattaacagaccgaaatcttaatacaactttctttgaccctgc

>R7238

ggagcttgagcaggaatagtaggaactgcactaagtattttaattcgagcagaattaggacagccaggcgcactactaggagatgaccaaatttataatgttattgttaccgcccatgcatttgttataatcttttttatagtaatgccaataataatcggaggtttcggaaactgacttgtaccactaataattggagccccagatatagcattcccacgaataaataatataagtttttgactacttcccccatcatttcttctcctactagcatcatcaatagtagaagctggggcaggaacaggatgaacagtctacccacctctagccggaaatttagcccatgcaggagcatctgtagatttaacaattttttctctacatttagccggtgtctcatctattttaggtgcaatcaactttattacaacaattattaatataaaacccccagctataactcagtatcaaaccccactatttgtctgatccgtattaattacagctgtattacttttattatcactgccggtattagctgcaggaattactatactattaacagaccgaaatcttaatacaactttctttgaccctgc

>R7239

ggagcttgagcaggaatagtaggaactgcactaagtattttaattcgagcagaattaggacagccaggcgcactactaggagatgaccaaatttataatgttattgttaccgcccatgcatttgttataatcttttttatagtaatgccaataataatcggaggtttcggaaactgacttgtaccactaataattggagccccagatatagcattcccacgaataaataatataagtttttgactacttcccccatcatttcttctcctactagcatcatcaatagtagaagctggggcaggaacaggatgaacagtctacccacctctagccggaaatttagcccatgcaggagcatctgtagatttaacaattttttctctacatttagccggtgtctcatctattttaggtgcaatcaactttattacaacaattattaatataaaacccccagctataactcagtatcaaaccccactatttgtctgatccgtattaattacagctgtattacttttattatcactgccggtattagctgcaggaattactatactattaacagaccgaaatcttaatacaactttctttgaccctgc

>R7243

ggagcttgagcaggaatagtaggaactgcactaagtattttaattcgagcagaattaggacagccaggcgcactactaggagatgaccaaatttataatgttattgttaccgcccatgcatttgttataatcttttttatagtaatgccaataataatcggaggtttcggaaactgacttgtaccactaataattggagccccagatatagcattcccacgaataaataatataagtttttgactacttcccccatcatttcttctcctattagcatcatcaatagtagaagctggggcaggaacaggatgaacagtctacccacctctagccggaaatttagcccatgcaggagcatctgtagatttaacaattttttctctacatttagccggtgtctcatctattttaggtgcaatcaactttattacaacaattattaatataaaacccccagctataactcagtatcaaaccccactatttgtctgatccgtattaattacagctgtattacttttattatcactgccggtattagctgcaggaattactatactattaacagaccgaaatcttaatacaactttctttgaccctgc

>R6321

ggagcctgagcaggaatagtaggaaccgcactaagtattctaattcgagcagaattaggacaaccaggtgcacttctaggagatgaccaaatttataatgttattgtaactgcccatgcattcgtaataattttttttatagttataccaataataattggaggcttcggaaactgacttgtaccactaataattggagccccagatatagcatttccacgaataaataatataagcttttgactacttcccccatctttcctccttcttctagcatcatctatagtagaagcaggagcaggaacgggatgaacagtttacccccctctagctggaaatttagctcatgcaggagcatcagtagacctaacaattttctccctccatttagctggtgtttcatctattctaggtgcaatcaactttattactacaattattaacataaaacccccagctataactcaatatcaaaccccgctatttgtttgatcagtactaattactgccgtattacttttactatccctaccagttctagctgcaggaattactatactgctaacagaccgtaaccttaatacaactttctttgatcctgc

>R6657

ggagcctgagcaggaatagtaggaaccgcactaagtattttaattcgagcagaattaggacaaccaggtgcacttctaggagatgaccaaatttataatgttattgtaactgcccatgcattcgtaataattttttttatagttataccaataataattggaggcttcggaaactgacttgtaccactaataattggagccccagatatagcatttccacgaataaataatataagcttttgactacttcccccatctttcctccttcttctagcatcatctatagtagaagcaggagcaggaacgggatgaacagtttacccccctctagctggaaatttagctcatgcaggagcatcagtagacctaacaattttctccctccatttagctggtgtttcatctattctaggtgcaatcaactttattactacaattattaacataaaacccccagctataactcaatatcaaaccccgctatttgtttgatcagtactaattactgccgtattacttttactatccctaccagttctagctgcaggaattactatactgctaacagaccgtaaccttaatacaactttctttgatcctgc

>R6685

ggagcctgagcaggaatagtaggaaccgcactaagtattttaattcgagcagaattaggacaaccaggtgcacttctaggagatgaccaaatttataatgttattgtaactgcccatgcattcgtaataattttttttatagttataccaataataattggaggcttcggaaactgacttgtaccactaataattggagccccagatatagcatttccacgaataaataatataagcttttgactacttcccccatctttcctccttcttctagcatcatctatagtagaagcaggagcaggaacgggatgaacagtttacccccctctagctggaaatttagctcatgcaggagcatcagtagacctaacaattttctccctccatttagctggtgtttcatctattctaggtgcaatcaactttattactacaattattaacataaaacccccagctataactcaatatcaaaccccgctatttgtttgatcagtactaattactgccgtattacttttactatccctaccagttctagctgcaggaattactatactgctaacagaccgtaaccttaatacaactttctttgatcctgc

>R7195

ggggcctgagcaggaatagtaggaaccgcactaagtattttaattcgagcagaattaggacaaccaggtgcacttctaggagatgaccaaatttataatgttattgtaactgcccatgcattcgtaataattttttttatagttataccaataataattggaggcttcggaaactgacttgtaccactaataattggagccccagatatagcattcccacgaataaataatataagcttttgactacttcccccatctttcctccttcttctagcatcatctatagtagaagcaggagcaggaacgggatgaactgtctacccccctctagctggaaatttagctcatgcaggagcatcagtagacctaacaattttctccctccatttagctggtgtatcatctattctaggtgcaatcaactttattaccacaattattaacataaaacccccagctataactcaatatcaaaccccactatttgtttgatcagtactaattactgctgtattacttttactatccctaccagttctagctgcaggaatcactatactgctaacagaccgaaatcttaatacaactttctttgatcctgc

>R7236

ggggcctgagcaggaatagtaggaaccgcactaagtattttaattcgagcagaattaggacaaccaggtgcacttctaggagatgaccaaatttataatgttattgtaactgcccatgcattcgtaataattttttttatagttataccaataataattggaggcttcggaaactgacttgtaccactaataattggagccccagatatagcattcccacgaataaataatataagcttttgactacttcccccatctttcctccttcttctagcatcatctatagtagaagcaggagcaggaacgggatgaactgtctacccccctctagctggaaatttagctcatgcaggagcatcagtagacctaacaattttctccctccatttagctggtgtatcatctattctaggtgcaatcaactttattaccacaattattaacataaaacccccagctataactcaatatcaaaccccactatttgtttgatcagtactaattactgctgtattacttttactatccctaccagttctagctgcaggaatcactatactgctaacagaccgaaatcttaatacaactttctttgatcctgc

>R7225

ggggcctgagcaggaatagtaggaaccgcactaagtattttaattcgagcagaattaggacaaccaggtgcacttctaggagatgaccaaatttataatgttattgtaactgcccatgcattcgtaataattttttttatagttataccaataataattggaggcttcggaaactgacttgtaccactaataattggagccccagatatagcattcccacgaataaataatataagcttttgactacttcccccatctttcctccttcttctagcatcatctatagtagaagcaggagcaggaacgggatgaactgtctacccccctctagctggaaatttagctcatgcaggagcatcagtagacctaacaattttctccctccatttagctggtgtatcatctattctaggtgcaatcaactttattaccacaattattaacataaaacccccagctataactcaatatcaaaccccactatttgtttgatcagtactaattactgctgtattacttttactatccctaccagttctagctgcaggaatcactatactgctaacagaccgaaatcttaatacaactttctttgatcctgc

>R7264

ggggcctgagcaggaatagtaggaactgcactaagtattttaattcgagcagaattaggacaaccaggtgcacttctaggagatgaccaaatttataatgttattgtaactgcccatgcattcgtaataattttttttatagttataccaataataattggaggcttcggaaactgacttgtaccactaataattggagccccagatatagcatttccacgaataaataatataagcttttgactgcttcccccatctttcctccttcttctagcatcatctatagtagaagcaggagcaggaacgggatgaacggtctacccccctctagctggaaatttagctcatgcaggagcatcagtagacctaacaattttctccctccatttagctggtgtttcatctattctaggtgcaatcaactttattactacaattattaacataaaacccccagctataactcaatatcaaaccccgctatttgtttgatcagtactaattactgctgtattacttttactatccctaccagttctagctgcaggaatcactatactgctaacagaccgaaaccttaatacaactttctttgatcctgc

>R7254

ggagcttgagcaggaatagtaggtaccgcactaagtattttaattcgagcagaattaggacagccaggtgcacttctaggagatgaccaaatttacaatgtcattgtcaccgcccacgcatttgttataattttcttcatagtaatgccaataataattggaggctttggaaactgacttgtgccactgataattggagccccagatatagcattcccacgaataaataatataagtttttgacttttacccccatcattccttctccttttagcatcatcaatagtagaagcaggagcaggtacaggatgaacagtctacccacctctagctggaaatctagcccatgcaggagcatcagtagatctaacaattttttccttacacttagctggagtatcatctatcctaggagcaatcaattttattactaccattattaacatgaaaccaccagccataactcaatatcaaaccccactatttgtatgatctgtacttatcacagccgtactacttttattatccttaccagttctagctgcaggaattactatactactgacagatcgaaacctaaacacaactttctttgaccctgc

>R7260

ggagcttgagcaggaatagtaggtaccgcactaagtattttaattcgagcagaattaggacagccaggtgcacttctaggagatgaccaaatttacaatgtcattgtcaccgcccacgcatttgttataattttcttcatagtaatgccaataataattggaggctttggaaactgacttgtgccactgataattggagccccagatatagcattcccacgaataaataatataagtttttgacttttacccccatcattccttctccttttagcatcatcaatagtagaagcaggagcaggtacaggatgaacagtctacccacctctagctggaaatctagcccatgcaggagcatcagtagatctaacaattttttccttacacttagctggagtatcatctatcctaggagcaatcaattttattactaccattattaacatgaaaccaccagccataactcaatatcaaaccccactatttgtatgatctgtacttatcacagccgtactacttttattatccttaccagttctagctgcaggaattactatactactgacagatcgaaacctaaacacaactttctttgaccctgc

>R7261

ggagcttgagcaggaatagtaggtaccgcactaagtattttaattcgagcagaattaggacagccaggtgcacttctaggagatgaccaaatttacaatgtcattgtcaccgcccacgcatttgttataattttcttcatagtaatgccaataataattggaggctttggaaactgacttgtgccactgataattggagccccagatatagcattcccacgaataaataatataagtttttgacttttacccccatcattccttctccttttagcatcatcaatagtagaagcaggagcaggtacaggatgaacagtctacccacctctagctggaaatctagcccatgcaggagcatcagtagatctaacaattttttccttacacttagctggagtatcatctatcctaggagcaatcaattttattactaccattattaacatgaaaccaccagccataactcaatatcaaaccccactatttgtatgatctgtacttatcacagccgtactacttttattatccttaccagttctagctgcaggaattactatactactgacagatcgaaacctaaacacaactttctttgaccctgc
