# Supplementary material for: Duplication and divergence of the retrovirus restriction gene Fv1 in Mus caroli allows protection from multiple retroviruses
Source: PLoS Genet. 2020 Jun 11;16(6):e1008471. doi: 10.1371/journal.pgen.1008471 (PMC7313476; doi:10.1371/journal.pgen.1008471)
Supplement: S2 Text — (DOCX) [file pgen.1008471.s013.docx]

>Fv1CAR1 KF975446 [organism=Mus caroli]

ATGAATTTCCCACGTGCGCTTGCTGGTTTCTCGAGCTGGCTCTTCAAACCTGAACTTGCCGAGGACTCTCCGGATAATGACGAGAACACTGTTAACCCATGGCGAGAGCTGCTGCAGAAGATAAATGTGGCCGATCTCCCCGATTCATCCTTTTCGAGCGGTAAGGAAGTTAATGACTCTGTGTACGATACTTTTGAACATTTCTGCAAGATTAGGGACTATGACGCAGTTGGCGAGCTGCTTCTGGCATTTCTGGATAAAGTAACAAAGGAAAGGGACCAATTCAGAGATGAAATTTCCCAGCTCCGAATGCACATAAATGATCTAAAGGCTTCTAAGTGTGTCCTGGGGGAGACTCTTCTCTCCTACCGCCACAGGATTGAAGTTGGGGAAAAACAGACTGAAGCCCTCATTGTGAGGTTAGCTGATGTGCAATCCCAGGTCATGTGTCAGCCTGCCCGGAAAGTGTCTGCAGATAAGGTGAGGGCACTGATTGGTAAAGAATGGGATCCGGTAACCTGGGATGGAGATGTGTGGGAGGACATAGATTCTGAGGGGACTGAGGAAGCAGCGTTGCTCCCCACTGTCTTGGCCTCTCCATCCTTGTCTGAGGAAAGTGGTTATGCCTTGTCTAAAGAACGCGCCCAGCAGGACAAAGCAGATGCTCCTCAGAACCAGTCTTCAACATCCTTAGTTACTTCTGAACCTGTCACCAGACCCAAGTCTCTGTCTGGCCTTATAAGTCAGAAACACCGCCATACTAACCATGAACTCAAATCACTTGCTCAATCATATCGCCAAAAGGCAAAGGAGCATGCGAGGAAATGGATTTTAAGGGTGTGGGATAATGGTGGGAGGCTCACAATACTGGATCAGACTGAATTTCTCAGTTTAGGTCCTTTGAGCCTTGATAGTGAGTTTAATGTCATAGCCCGCACGGTTGCAGATAATGGAGTGAAGAGTTTGTTTGATTGGTTGGCTGAAGCATGGGTCCAGAGATGGCCTACTACTACAAGAGAGCTGCAGACGCCTGACGCCCTGAAGTGGTATTATATTGAGGATGGGATTAAAAGGCTTAGGGAACTTGGAATGATAGAGTGGCTTTGTGTAAAAGCTACTTGTCCACAGTGGAGGGGCCCGGAAGATGCACCCATCACGAGAGCTATGAGGATAACTTTTGTCCAGGAAACTGTAGAGACTTGGAAGAGCTTCGTATTTAGCCTCCTCTGTATAAAGGACATAACAGTGGGGGGCGTGGCTGCCCAGTTGCATGATCTAATAAAATTGAGTTTAAAGCCAACAGCAGCTGGCTTGACTGCTGGTTCGGCTCCTGTGGGCTCGGTGGCTCCTGTGGGCTCGGTGGCGGTTCTCTCTCTCTCTCCCTGGAAACATCAAAGCAACAGTTAA

>Fv1CAR2 MT077217 [organism=Mus caroli]

ATGAATTTCCCACGTGCGCTTGCTGGTTTCTCGAGCTGGCTCTTCAAACCTGAACTTGCCGAGGACTCTCCGGATAATGACGAGAACACTGTTAACCCATGGCGAGAGCTGCTGCAGAAGATAAATGTGGCCGATCTCCCCGATTCATCCTTTTCGAGCGGTAAGGAAGTTAATGACTCTGTGTACGATACTTTTGAACATTTCTGCAAGATTAGGGACTATGACGCAGTTGGCGAGCTGCTTCTGGCATTTCTGGATAAAGTAACAAAGGAAAGGGACCAATTCAGAGATGAAATTTCCCAGCTCCGAATGCACATAAATGATCTAAAGGCTTCTAAGTGTGTCCTGGGGGAGACTCTTCTCTCCTACCGCCACAGGATTGAAGTTGGGGAAAAACAGACTGAAGCCCTCATTGTGAGGTTAGCTGATGTGCAATCCCAGGTCATGTGTCAGCCTGCCCGGAAAGTGTCTGCAGATAAGGTGAGGGCACTGATTGGTAAAGAATGGGATCCGGTAACCTGGGATGGAGATGTGTGGGAGGACATAGATTCTGAGGGGACTGAGGAAGCAGCGTTGCCCCCCACTGTCTTGGCCTCTCCATCCTTGTCTGAGGAAAGTGGTTATGCCTTGTCTAAAGAACTCGCCCAGCAGGACAAAGCAGATGCTCCTCAGAACCAGTCTTCAACATCCTTAGTTACTTCTGAACCTGTCACCAGACCCAAGTCTCTGTCTGGCCTTATAAGTCAGAAACACCGCCATACTAACCATGAACTCAAATCACTTGCTCAATCATATCGCCAAAAGGCAAAGGAGCATGCGAGGAAATGGATTTTAAGGGTGTGGGATAATGGTGGGAGGCTCACAATACTGGATCAGACTGAATTTCTCAGTTTAGGTCCTTTGAGCCTTGATAGTGAGTTTAATGTCATAGCCCGCACGGTTGCAGATAATGGAGTGAAGAGTTTGTTTGATTGGTTGGCTGAAGCATGGGTCCAGAGATGGCCTACTACTACAAGAGAGCTGCAGACGCCTGACGCCCTGAAGTGGTATTATATTGAGGATGGGATTAAAAGGCTTAGGGAACTTGGAATGATAGAGTGGCTTTGTGTAAAAGCTACTTGTCCACAGTGGAGGGGCCCGGAAGATGCACCCATCACGAGAGCTATGAGGATAACTTTTGTCCAGGAAACTGTAGAGACTTGGAAGAGCTTCGTATTTAGCCTCCTCTGTATAAAGGACATAACAGTGGGGGGCGTGGCTGCCCAGTTGCATGATCTAATAGAATTGAGTTTAAAGCCAACAGCAGCTGGCTTGACTGCTGGTTCGGCTCCTGTGGGCTCGGTGGCTCCTGTGGGCTCGGTGGCGGTTCTCTCTCTCTCTCCCTGGAAACATCAAAGCAACAGTTAA

>Fv1CAR3 MT077218 [organism=Mus caroli]

ATGAATTTCCCACGTGCGCTTGCTGGTTTCTCGAGCTGGCTCTTCAAACCTGAACTTGCCGAGGACTCTCCGGATAATGACGAGAACACTGTTAACCCATGGCGAGAGCTGCTGCAGAAGATAAATGTGGCCGATCTCCCCGATTCATCCTTTTCGAGCGGTAAGGAAGTTAATGACTCTGTGTACGATACTTTTGAACATTTCTGCAAGATTAGGGACTATGACGCAGTTGGCGAGCTGCTTCTGGCATTTCTGGATAAAGTAACAAAGGAAAGGGACCAATTCAGAGATGAAATTTCCCAGCTCCGAATGCACATAAATGATCTAAAGGCTTCTAAGTGTGTCCTGGGGGAGACTCTTCTCTCCTACCGCCACAGGATTGAAGTTGGGGAAAAACAGACTGAAGCCCTCATTGTGAGGTTAGCTGATGTGCAATCCCAGGTCATGTGTCAGCCTGCCCGGAAAGTGTCTGCAGATAAGGTGAGGGCACTGATTGGTAAAGAATGGGATCCGGTAACCTGGGATGGAGATGTGTGGGAGGACATAGATTCTGAGGGGACTGAGGAAGCAGCGTTGCCCCCCACTGTCTTGGCCTCTCCATCCTTGTCTGAGGAAAGTGGTTATGCCTTGTCTAAAGAACGCGCCCAGCAGGACAAAGCAGATGCTCCTCAGAACCAGTCTTCAACATCCTTAGTTACTTCTGAACCTGTCACCAGACCCAAGTCTCTGTCTGGCCTTATAAGTCAGAAACACCGCCATACTAACCATGAACTCAAATCACTTGCTCAATCATATCGCCAAAAGGCAAAGGAGCATGCGAGGAAATGGATTTTAAGGGTGTGGGATAATGGTGGGAGGCTCACAATACTGGATCAGACTGAATTTCTCAGTTTAGGTCCTTTGAGCCTTGATAGTGAGTTTAATGTCATAGCCCGCACGGTTGCAGATAATGGAGTGAAGAGTTTGTTTGATTGGTTGGCTGAAGCATGGGTCCAGAGATGGCCTACTACTACAAGAGAGCTGCAGACGCCTGACGCCCTGAAGTGGTATTATATTGAGGATGGGATTAAAAGGCTTAGGGAACTTGGAATGATAGAGTGGCTTTGTGTAAAAGCTACTTGTCCACAGTGGAGGGGCCCGGAAGATGCACCCATCACGAGAGCTATGAGGATAACTTTTGTCCAGGAAACTGTAGAGACTTGGAAGAGCTTCGTATTTAGCCTCCTCTGTATAAAGGACATAACAGTGGGGGGCGTGGCTGCCCAGTTGCATGATCTAATAGAATTGAGTTTAAAGCCAACAGCAGCTGGCTTGACTGCTGGTTCGGCTCCTGTGGGCTCGGTGGCTCCTGTGGGCTCGGTGGCGGTTCTCTCTCTCTCTCCCTGGAAACATCAAAGCAACAGTTAA

>Fv1CAR4 MT077219 [organism=Mus caroli]

ATGAATTTCCCACGTGCGCTTGCTGGTTTCTCGAGCTGGCTCTTCAAACCTGAACTTGCCGAGGACTCTCCGGATAATGACGAGAACACTGTTAACCCATGGCGAGAGCTGCTGCAGAAGATAAATGTGGCCGATCTCCCCGATTCATCCTTTTCGAGCGGTAAGGAAGTTAATGACTCTGTGTACGATACTTTTGAACATTTCTGCAAGATTAGGGACTATGACGCAGTTGGCGAGCTGCTTCTGGCATTTCTGGATAAAGTAACAAAGGAAAGGGACCAATTCAGAGATGAAATTTCCCAGCTCCGAATGCACATAAATGATCTAAAGGCTTCTAAGTGTGTCCTGGGGGAGACTCTTCTCTCCTACCGCCACAGGATTGAAGTTGGGGAAAAACAGACTGAAGCCCTCATTGTGAGGTTAGCTGATGTGCAATCCCAGGTCATGTGTCAGCCTGCCCGGAAAGTGTCTGCAGATAAGGTGAGGGCACTGATTGGTAAAGAATGGGATCCGGTAACCTGGGATGGAGATGTGTGGGAGGACATAGATTCTGAGGGGACTGAGGAAGCAGCGTTGCCCCCCACTGTCTTGGCCTCTCCATCCTTGTCTGAGGAAAGTGGTTATGCCTTGTCTAAAGAACTCGCCCAGCAGGACAAAGCAGATGCTCCTCAGAACCAGTCTTCAACATCCTTAGTTACTTCTGAACCTGTCACCAGACCCAAGTCTCTGTCTGGCCTTATAAGTCAGAAACACCGCCATACTAACCATGAACTCAAATCACTTGCTCAATCATATCGCCAAAAGGCAAAGGAGCATGCGAGGAAATGGATTTTAAGGGTGTGGGATAATGGTGGGAGGCTCACAATACTGGATCAGACTGAATTTCTCAGTTTAGGTCCTTTGAGCCTTGATAGTGAGTTTAATGTCATAGCCCGCACGGTTGCAGATAATGGAGTGAAGAGTTTGTTTGATTGGTTGGCTGAAGCATGGGTCCAGAGATGGCCTACTACAAGAGAGCTGCAGACGCCTGACGCCCTGAAGTGGTATTATATTGAGGATGGGATTAAAAGGCTTAGGGAACTTGGAATGATAGAGTGGCTTTGTGTAAAAGCTACTTGTCCACAGTGGAGGGGCCCGGAAGATGCACCCATCACGAGAGCTATGAGGATAACTTTTGTCCAGGAAACTGTAGAGACTTGGAAGAGCTTCGTATTTAGCCTCCTCTGTATAAAGGACATAACAGTGGGGGGCGTGGCTGCCCAGTTGCATGATCTAATAGAATTGAGTTTAAAGCCAACAGCAGCTGGCTTGACTGCTGGTTCGGCTCCTGTGGGCTCGGTGGCTCCTGTGGGCTCGGTGGCGGTTCTCTCTCTCTCTCCCTGGAAACATCAAAGCAACAGTTAA

>Fv1CAR5 MT077220 [organism=Mus caroli]

ATGAATTTCCCACGTGCGCTTGCTGGTTTCTCGAGCTGGCTCTTCAAACCTGAACTTGCCGAGGACTCTCCGGATAATGACGAGAACACTGTTAACCCATGGCGAGAGCTGCTGCAGAAGATAAATGTGGCCGATCTCCCCGATTCATCCTTTTCGAGCGGTAAGGAAGTTAATGACTCTGTGTACGATACTTTTGAACATTTCTGCAAGATTAGGGACTATGACGCAGTTGGCGAGCTGCTTCTGGCATTTCTGGATAAAGTAACAAAGGAAAGGGACCAATTCAGAGATGAAATTTCCCAGCTCCGAATGCACATAAATGATCTAAAGGCTTCTAAGTGTGTCCTGGGGGAGACTCTTCTCTCCTACCGCCACAGGATTGAAGTTGGGGAAAAACAGACTGAAGCCCTCATTGTGAGGTTAGCTGATGTGCAATCCCAGGTCATGTGTCAGCCTGCCCGGAAAGTGTCTGCAGATAAGGTGAGGGCACTGATTGGTAAAGAATGGGATCCGGTAACCTGGGATGGAGATGTGTGGGAGGACATAGATTCTGAGGGGACTGAGGAAGCAGCGTTGCTCCCCACTGTCTTGGCCTCTCCATCCTTGTCTGAGGAAAGTGGTTATGCCTTGTCTAAAGAACGCGCCCAGCAGGACAAAGCAGATGCTCCTCAGAACCAGTCTTCAACATCCTTAGTTACTTCTGAACCTGTCACCAGACCCAAGTCTCTGTCTGGCCTTATAAGTCAGAAACACCGCCATACTAACCATGAACTCAAATCACTTGCTCAATCATATCGCCAAAAGGCAAAGGAGCATGCGAGGAAATGGATTTTAAGGGTGTGGGATAATGGTGGGAGGCTCACAATACTGGATCAGACTGAATTTCTCAGTTTAGGTCCTTTGAGCCTTGATAGTGAGTTTAATGTCATAGCCCGCACGGTTGCAGATAATGGAGTGAAGAGTTTGTTTGATTGGTTGGCTGAAGCATGGGTCCAGAGATGGCCTACTACTACAAGAGAGCTGCAGACGCCTGACGCCCTGAAGTGGTATTATATTGAGGATGGGATTAAAAGGCTTAGGGAACTTGGAATGATAGAGTGGCTTTGTGTAAAAGCTACTTGTCCACAGTGGAGGGGCCCGGAAGATGCACCCATCACGAGAGCTATGAGGATAACTTTTGTCCAGGAAACTGTAGAGACTTGGAAGAGCTTCGTATTTAGCCTCCTCTGTATAAAGGACATAACAGTGGGGGGCGTGGCTGCCCAGTTGCATGATCTAATAGAATTGAGTTTAAAGCCAACAGCAGCTGGCTTGACTGCTGGTTCGGCTCCTGTGGGCTCGGTGGCTCCTGTGGGCTCGGTGGCGGTTCTCTCTCTCTCTCCCTGGAAACATCAAAGCAACAGTTAA

>Fv1CAR6 MT077221 [organism=Mus caroli]

ATGAATTTCCCACGTGCGCTTGCTGGTTTCTCGAGCTGGCTCTTCAAACCTGAACTTGCCGAGGACTCTCCGGATAATGACGAGAACACTGTTAACCCATGGCGAGAGCTGCTGCAGAAGATAAATGTGGCCGATCTCCCCGATTCATCCTTTTCGAGCGGTAAGGAAGTTAATGACTCTGTGTACGATACTTTTGAACATTTCTGCAAGATTAGGGACTATGACGCAGTTGGCGAGCTGCTTCTGGCATTTCTGGATAAAGTAACAAAGGAAAGGGACCAATTCAGAGATGAAATTTCCCAGCTCCGAATGCACATAAATGATCTAAAGGCTTCTAAGTGTGTCCTGGGGGAGACTCTTCTCTCCTACCGCCACAGGATTGAAGTTGGGGAAAAACAGACTGAAGCCCTCATTGTGAGGTTAGCTGATGTGCAATCCCAGGTCATGTGTCAGCCTGCCCGGAAAGTGTCTGCAGATAAGGTGAGGGCACTGATTGGTAAAGAATGGGATCCGGTAACCTGGGATGGAGATGTGTGGGAGGACATAGATTCTGAGGGGACTGAGGAAGCAGCGTTGCCCCCCACTGTCTTGGCCTCTCCATCCTTGTCTGAGGAAAGTGGTTATGCCTTGTCTAAAGAACGCGCCCAGCAGGACAAAGCAGATGCTCCTCAGAACCAGTCTTCAACATCCTTAGTTACTTCTGAACCTGTCACCAGACCCAAGTCTCTGTCTGGCCTTATAAGTCAGAAACACCGCCATACTAACCATGAACTCAAATCACTTGCTCAATCATATCGCCAAAAGGCAAAGGAGCATGCGAGGAAATGGATTTTAAGGGTGTGGGATAATGGTGGGAGGCTCACAATACTGGATCAGACTGAATTTCTCAGTTTAGGTCCTTTGAGCCTTGATAGTGAGTTTAATGTCATAGCCCGCACGGTTGCAGATAATGGAGTGAAGAGTTTGTTTGATTGGTTGGCTGAAGCATGGGTCCAGAGATGGCCTACTACAAGAGAGCTGCAGACGCCTGACGCCCTGAAGTGGTATTATATTGAGGATGGGATTAAAAGGCTTAGGGAACTTGGAATGATAGAGTGGCTTTGTGTAAAAGCTACTTGTCCACAGTGGAGGGGCCCGGAAGATGCACCCATCACGAGAGCTATGAGGATAACTTTTGTCCAGGAAACTGTAGAGACTTGGAAGAGCTTCGTATTTAGCCTCCTCTGTATAAAGGACATAACAGTGGGGGGCGTGGCTGCCCAGTTGCATGATCTAATAGAATTGAGTTTAAAGCCAACAGCAGCTGGCTTGACTGCTGGTTCTGCTCCTGTGGGCTCGGTGGCTCCTGTGGGCTCGGTGGCGGTTCTCTCTCTCTCTCCCTGGAAACATCAAAGCAACAGTTAA

>Fv1CAR7 MT077222 [organism=Mus caroli]

ATGAATTTCCCACGTGCGCTTGCTGGTTTCTCGAGCTGGCTCTTCAAACCTGAACTTGCCGAGGACTCTCCGGATAATGACGAGAACACTGTTAACCCATGGCGAGAGCTGCTGCAGAAGATAAATGTGGCCGATCTCCCCGATTCATCCTTTTCGAGCGGTAAGGAAGTTAATGACTCTGTGTACGATACTTTTGAACATTTCTGCAAGATTAGGGACTATGACGCAGTTGGCGAGCTGCTTCTGGCATTTCTGGATAAAGTAACAAAGGAAAGGGACCAATTCAGAGATGAAATTTCCCAGCTCCGAATGCACATAAATGATCTAAAGGCTTCTAAGTGTGTCCTGGGGGAGACTCTTCTCTCCTACCGCCACAGGATTGAAGTTGGGGAAAAACAGACTGAAGCCCTCATTGTGAGGTTAGCTGATGTGCAATCCCAGGTCATGTGTCAGCCTGCCCGGAAAGTGTCTGCAGATAAGGTGAGGGCACTGATTGGTAAAGAATGGGATCCGGTAACCTGGGATGGAGATGTGTGGGAGGACATAGATTCTGAGGGGACTGAGGAAGCAGCGTTGCTCCCCACTGTCTTGGCCTCTCCATCCTTGTCTGAGGAAAGTGGTTATGCCTTGTCTAAAGAACGCGCCCAGCAGGACAAAGCAGATGCTCCTCAGAACCAGTCTTCAACATCGTTAGTTACTTCTGAACCTGTCACCAGACCCAAGTCTCTGTCTGGCCTTATAAGTCAGAAACACCGCCATACTAACCATGAACTCAAATCACTTGCTCAATCATATCGCCAAAAGGCAAAGGAGCATTCGAGGAAATGGATTTTAAGGGTGTGGGATAATGGTGGGAGGCTCACAATACTGGATCAGACTGAATTTCTCAGTTTAGGTCCTTTGAGCCTTGATAGTGAGTTTAATGTCATAGCCCGCACGGTTGCAGATAATGGAGTGAAGAGTTTGTTTGATTGGTTGGCTGAAGCATGGGTCCAGAGATGGCCTACTACTACAAGAGAGCTGCAGACGCCTGACGCCCTGAAGTGGTATTATATTGAGGATGGGATTAAAAGGCTTAGGGAACTTGGAATGATAGAGTGGCTTTGTGTAAAAGCTACTTGTCCACAGTGGAGGGGCCCGGAAGATGCACCCATCACGAGAGCTATGAGGATAACTTTTGTCCAGGAAACTGTAGAGACTTGGAAGAGCTTCGTATTTAGCCTCCTCTGTATAAAGGACATAACAGTGGGGGGCGTGGCTGCCCAGTTGCATGATCTAATAGAATTGAGTTTAAAGCCAACAGCAGCTGGCTTGACTGCTGGTTCGGCTCCTGTGGGCTCGGTGGCTCCTGTGGGCTCGGTGGCGGTTCTCTCTCTCTCTCCCTGGAAACATCAAAGCAACAGTTAA

>Fv1CAR8 MT077223 [organism=Mus caroli]

ATGAATTTCCCACGTGCGCTTGCTGGTTTCTCGAGCTGGCTCTTCAAACCTGAACTTGCCGAGGACTCTCCGGATAATGACGAGAACACTGTTAACCCATGGCGAGAGCTGCTGCAGAAGATAAATGTGGCCGATCTCCCCGATTCATCCTTTTCGAGCGGTAAGGAAGTTAATGACTCTGTGTACGATACTTTTGAACATTTCTGCAAGATTAGGGACTATGACGCAGTTGGCGAGCTGCTTCTGGCATTTCTGGATAAAGTAACAAAGGAAAGGGACCAATTCAGAGATGAAATTTCCCAGCTCCGAATGCACATAAATGATCTAAAGGCTTCTAAGTGTGTCCTGGGGGAGACTCTTCTCTCCTACCGCCACAGGATTGAAGTTGGGGAAAAACAGACTGAAGCCCTCATTGTGAGGTTAGCTGATGTGCAATCCCAGGTCATGTGTCAGCCTGCCCGGAAAGTGTCTGCAGATAAGGTGAGGGCACTGATTGGTAAAGAATGGGATCCGGTAACCTGGGATGGAGATGTGTGGGAGGACATAGATTCTGAGGGGACTGAGGAAGCAGCGTTGCTCCCCACTGTCTTGGCCTCTCCATCCTTGTCTGAGGAAAGTGGTTATGCCTTGTCTAAAGAACGCGCCCAGCAGGACAAAGCAGATGCTCCTCAGAACCAGTCTTCAACATCCTTAGTTACTTCTGAACCTGTCACCAGACCCAAGTCTCTGTCTGGCCTTATAAGTCAGAAACACCGCCATACTAACCATGAACTCAAATCACTTGCTCAATCATATCGCCAAAAGGCAAAGGAGCATTCGAGGAAATGGATTTTAAGGGTGTGGGATAATGGTGGGAGGCTCACAATACTGGATCAGACTGAATTTCTCAGTTTAGGTCCTTTGAGCCTTGATAGTGAGTTTAATGTCATAGCCCGCACGGTTGCAGATAATGGAGTGAAGAGTTTGTTTGATTGGTTGGCTGAAGCATGGGTCCAGAGATGGCCTACTACTACAAGAGAGCTGCAGACGCCTGACGCCCTGAAGTGGTATTATATTGAGGATGGGATTAAAAGGCTTAGGGAACTTGGAATGATAGAGTGGCTTTGTGTAAAAGCTACTTGTCCACAGTGGAGGGGCCCGGAAGATGCACCCATCACGAGAGCTATGAGGATAACTTTTGTCCAGGAAACTGTAAAGACTTGGAAGAGCTTCGTATTTAGCCTCCTCTGTATAAAGGACATAACAGTGGGGGGCGTGGCTGCCCAGTTGCATGATCTAATAGAATTGAGTTTAAAGCCAACAGCAGCTGGCTTGACTGCTGGTTCGGCTCCTGTGGGCTCGGTGGCTCCTGTGGGCTCGGTGGCGGTTCTCTCTCTCTCTCCCTGGAAACATCAAAGCAACAGTTAA

>Fv7CAR1 KF975447 [organism=Mus caroli]

ATGAATTTCCCACGTGCGCTTGCTGGTTTCTCAAGCTGGCTCTTCAAACCTGAACTTGCCGAGGACACTCCGGATAATGACGAGAACACTGTTAACCCATGGCGTGAGCTGCTGCGGAAGATAAATGTGGCCGATCTCCCCGATTCATCCTTTTCGAGCGGTAAGGAACTTAATGTCTCTGTGTACGATACTTTTGAACATTTCTGCAAGATTAGGGACTATGACGCAGTTGGCGAGCTGCTTCTGGCATTTCTGGATAAAGTAACAAAGGAAAGGGACCAATTCAGAGATGAAATTTCCCAGCTCCGAATGCACATAAATGATCTAAAGGCTTCTAAGTGTGTCCTGGGGAGACTCTTCTCTCCTACCGCCACAGGATTGAAGTTGGGGAAAAACAGACTGAAGCCCTCATTGTGAGGTTAGCTGATTTGCAATCCCAGGTCATGTGTCAGCCTGCCCGGAAAGTGTCTGCAGATAAGGTGAGGGCACTGATTGGTAAAGAATGGGATCCGGTAACCTGGGATGGAGATGTGTGGGAGGACATAGATTCTGATGGGACTGAGGAAGCAGAGTTGATCACTGTCTTGGCCTCTCCATCCTTGTCTGAGGAAAGTGGTTATGCCTTGTCAAAAGAACGCGCCCAGCAGGACAAAGCAGATGCTCCTCAGAACCAGTCTTCAACATCCTTAGTTACTTCTGAACCTGTCACCAGACCCAAGTCTCTGTCTGACCTTACAAGTCAGAAACACCGCCATACTAACCATGAACTCAAATCACTTGCTCAATCAAATCACCAACAGGCAAAGGAACATGCGAGGAAATGGATTTTAAGGGTGTGGGATAATGGTGGGAGGCTCACAATACTGGATCAGACTGAATTTCTCAGTTTAGGTCCTTTGAGCCTTGATAGTGAGTTTAATGTCATAGCCCGCACGGTTGAAGATAACGGTGTGAAGAGTTTGTTTGATTGGTTGGCTGAAGCATGGGTCCAGAGATGGCCTACTACAAGAGAGCTGCAGACGCCTGACGCCCTGGAGTGGTATTCTATTGGGGATGGGATTAAAAGGCTTAGGGAACTTGGAATGATAGAGTGGCTTTGTGTAAAAGCTACTTGTCCACAGTGGAGGGGCCCGGAAGATGCACCCATCACGAGAGCTATGAGGATAACTTTTGTCCGGGAAACTGTAGAGACTTGGAAGAGCTTCGTATTTAGCCTCCTCTGTATAAAGGACATAACAGTGGGGGGCGTGGCTGCCCAGTTGCATGATCTAATAGAATTAAGTTTAAAGCCAACAGCAGCTGGCTTGACTGCTGGTTCAGCTCCTGTGGGCTCCGTGGCGGTTCTCTCTCTCTCCCCCTGGAAACATCAAAGCAACAGTTAA

>Fv7CAR2 MT077224 [organism=Mus caroli]

ATGAATTTCCCACGTGCGCTTGCTGGTTTCTCGAGCTGGCTCTTCAAACCTGAACTTGCTGAGGACTCTCCGGATAATGACGAGAACATTGTTAACCCATGGCGTGAGCTGCTGCAGAAGATAAATGTGGCCGATCTCCCCGATTCATCCTTTTCGAGCGGTAAGGAACTTAATGACTCTGTGTACGATACTTTTGAACATTTCTGCAAGATTAGGGACTATGACGCAGTTGGCGAGCTGCTTCTGGCATTTCTGGATAAAGTAACAAAGGAAAGGGACCAATTCAGAGATGAAATTTCCCAGCTCCGAATGCACATAAATGATCTAAAGGCTTCTAAGTGTGTCCTGGGGGAGACTCTTCTCTCCTACCGCCACAGGATTGAAGTTGGGGAAAAACAGACTGAAGCCCTCATTGTGAGGTTAGCTGATTTGCAATCCCAGGTCATGTGTCAGCCTGCCCGGAAAGTGTCTGCAGATAAGGTGAGGGCACTGATTGGTAAAGAATGGGATCCGGTAACCTGGGATGGAGATGTGTGGGAGGATATAGATTCTGATGGGACTGAGGAAGCAGAGTTGACCACTGTCTTGGCCTCTCCATCCTTGTCTGAGGAAAGTGGTTATGCCTTGTCAAAAGAACGCGCCCAGCAGGACAAAGCAGATGCTCCTCAGAACCAGTCTTTAACATCCTTAGTTACTTCTGAACCTGTCACCAGACCCAAGTCTCTGTCTGACCTTACAAGTCAGAAACACCGCCATACTAACCATGAACTCAAATCACTTGCTCAATCAAATCGCCAACAGGCAAAGGAGCATGCGAGGAAATGGATTTTAAGGGTGTGGGATAATGGTGGGAGGCTCACAATACTGGATCAGGCTGAATTTCTCAGTTTAGGTCCTTTGAGCCTTGATAGTGAGTTTAATGTCATAGCCCGCACGGTTGAAGATAACGGTGTGAAGAGTTTGTTTGATTGGTTGGCTGAAGCATGGGTCCAGAGATGGCCTACTACAAGAGAGCTGCAGACGCCTGACGCCCTGGAGTGGTATTCTATTGAGGATGGGATTAAAAGGCTTAGGGAACTTGGAATGATAGAGTGGCTTTGTGTAAAAGCTACTTGTCCACAGTGGAGGGGCCCGGAAGATGCACCCATCACGAGAGCTATGAGGATAACTTTTGTCCGGGAAACTGTAGAGACTTGGAAGAGCTTCGTATTTAGCCTCCTCTGTATAAAGGACATAACAGTGGGGGGCGTGGCTGCCCAGTTGCATGATCTAATAGAATTAAGTTTAAAGCCAACAGCAGCTGGCTTGACTGCTGGTTCGGCTCCTGTGGGCTCCGTGGGGGTTCTCTCTCTCTCCCCCTGGAAACATCAAAGCAACAGTTAA

>Fv7CAR3 MT077225 [organism=Mus caroli]

ATGAATTTCCCACGTGCGCTTGCTGGTTTCTCAAGCTGGCTCTTCAAACCTGAACTTGCTGAGGACACTCCGGATAATGATGAGAACACTGTTAACCCATGGCGTGAGCTGCTGCAGAAGATAAATGTGGCCGATCTCCCCGATTCATCCTTTTCGAGCGGTAAGGAACTTAATGTCTCTGTGTACGATACTTTTGAACATTTCTGCAAGATTAGGGACTATGACGCAGTTGGCGAGCTGCTTCTGGCATTTCTGGATAAAGTAACAAAGGAAAGGGACCAATTCAGAGATGAAATTTCCCAGCTCCGAATGCACATAAATGATCTAAAGGCTTCTAAGTGTGTCCTGGGGGAGACTCTTCTCTCCTACCGCCACAGGATTGAAGTTGGGGAAAAACAGACTGAAGCCCTCATTGTGAGGTTAGCTGATTTGCAATCCCAGGTCATGTGTCAGCCTGCCCGGAAAGTGTCTGCAGATAAGGTGAGGGCACTGATTGGTAAAGAATGGGATCCGGTAACCTGGGATGGAGATGTGTGGGAGGATATAGATTCTGATGGGACTGAGGAAGCAGAGTTGACCACTGTCTTGGCCTCTCCATCCTTGTCTGAGGAAAGTGGTTATGCCTTGTCAAAAGAACGCGCCCAGCAGGACAAAGCAGATGCTCCTCAGAACCAGTCTTCAACATCCTTAGTTACTTCTGAACCTGTCACCAGACCCAAGTCTCTGTCTGACCTTACAAGTCAGAAACACCGCCATACTAACCATGAACTCAAATCACTTGCTCAATCAAATCGCCAACAGGCAAAGGAGCATGCGAGGAAATGGATTTTAAGGGTGTGGGATAATGGTGGGAGGCTCACAATACTGGATCAGGCTGAATTTCTCAGTTTAGGTCCTTTGAGCCTTGATAGTGAGTTTAATGTCATAGCCCGCACGGTTGAAGATAACGGTGTGAAGAGTTTGTTTGATTGGTTGGCTGAAGCATGGGTCCAGAGATGGCCTACTACAAGAGAGCTGCAGACGCCTGACGCCCTGGAGTGGTATTCTATTGAGGATGGGATTAAAAGGCTTAGGGAACTTGGAATGATAGAGTGGCTTTGTGTAAAAGCTACTTGTCCACAGTGGAGGGGCCCGGAAGATGCACCCATCACGAGAGCTATGAGGATAACTTTTGTCCGGGAAACTGTAGAGACTTGGAAGAGCTTCGTATTTAGCCTCCTCTGTATAAAGGACATAACAGTGGGGGGCGTGGCTGCCCAGTTGCATGATCTAATAGAATTAAGTTTAAAGCCAACAGCAGCTGGCTTGACTGCTGGTTCGGCTCCTGTGGGCTCCGTGGGGGTTCTCTCTCTCTCCCCCTGGAAACATCAAAGCAACAGTTAA

>Fv7CAR4 MT077226 [organism=Mus caroli]

ATGAATTTCCCACGTGCGCTTGCTGGTTTCTCAAGCTGGCTCTTCAAACCTGAACTTGCCGAGGACACTCCGGATAATGACGAGAACACTGTTAACCCATGGCGTGAGCTGCTGCAGAAGATAAATGTGGCCGATCTCCCCGATTCATCCTTTTCGAGCGGTAAGGAACTTAATGTCTCTGTGTACGATACTTTTGAACATTTCTGCAAGATTAGGGACTATGACGCAGTTGGCGAGCTGCTTCTGGCATTTCTGGATAAAGTAACAAAGGAAAGGGACCAATTCAGAGATGAAATTTCCCAGCTCCGAATGCACATAAATGATCTAAAGGCTTCTAAGTGTGTCCTGGGGGAGACTCTTCTCTCCTACCGCCACAGGATTGAAGTTGGGGAAAAACAGACTGAAGCCCTCATTGTGAGGTTAGCTGATTTGCAATCCCAGGTCATGTGTCAGCCTGCCCGGAAAGTGTCTGCAGATAAGGTGAGGGCACTGATTGGTAAAGAATGGGATCCGATAACCTGGGATGGAGATGTGTGGGAGGACATAGATTCTGATGGGACTGAGGAAGCAGAGTTGACCACTGTCTTGGCCTCTCCATCCTTGTCTGAGGAAAGTGGTTATGCCTTGTCAAAAGAACGCGCCCAGCAGGACAAAGCAGATGCTCCTCAGAACCAGTCTTCAACATCCTTAGTTACTTCTGAACCTGTCACCAGACCCAAGTCTCTGTCTGACCTTACAAGTCAGAAACACCGCCATACTAACCATGAACTCAAATCACTTGCTCAATCAAATCGCCAACAGGCAAAGGAACATGCGAGGAAATGGATTTTAAGGGTGTGGGATAATGGTGGGAGGCTCACAATACTGGATCAGACTGAATTTCTCAGTTTAGGTCCTTTGAGCCTTGATAGTGAGTTTAATGTCATAGCCCGCACGGTTGAAGATAACGGTGTGAAGAGTTTGTTTGATTGGTTGGCTGAAGCATGGGTCCAGAGATGGCCTACTACAAGAGAGCTGCAGACGCCTGATGCCCTGGAGTGGTATTCTATTGAGGATGGGATTAAAAGGCTTAGGGAACTTGGAATGATAGAGTGGCTTTGTGTAAAAGCTACTTGTCCACAGTGGAGGGGCCCGGAAGATGCACCCATCACGAGAGCTATGAGGATAACTTTTGTCCGGGAAACTGTAGAGACTTGGAAGAGCTTCGTATTTAGCCTCCTCTGTATAAAGGACATAACAGTGGGGGGCGTGGCTGCCCAGTTGCATGATCTAATAGAATTAAGTTTAAAGCCAACAGCAGCTGGCTTGACTGCTGGTTCGGCTCCTGTGGGCTCCGTGGCGGTTCTCTCTCTCTCTCCCTGGAAACATCAAAGCAACAGTTAA

>Fv7CAR5 MT077227 [organism=Mus caroli]

ATGAATTTCCCACGTGCGCTTGCTGGTTTCTCGAGCTGGCTCTTCAAACCTGAACTTGCTGAGGACTCTCCGGATAATGACGAGAACATTGTTAACCCATGGCGTGAGCTGCTGCAGAAGATAAATGTGGCCGATCTCCCCGATTCATCCTTTTCGAGCGGTAAGGAACTTAATGACTCTGTGTACGATACTTTTGAACATTTCTGCAAGATTAGGGACTATGACGCAGTTGGCGAGCTGCTTCTGGCATTTCTGGATAAAGTAACAAAGGAAAGGGACCAATTCAGAGATGAAATTTCCCAGCTCCGAATGCACATAAATGATCTAAAGGCTTCTAAGTGTGTCCTGGGGGAGACTCTTCTCTCCTACCGCCACAGGATTGAAGTTGGGGAAAAACAGACTGAAGCCCTCATTGTGAGGTTAGCTGATGTGCAATCCCAGGTCATGTGTCAGCCTGCCCGGAAAGTGTCTGCAGATAAGGTGAGGGCACTGATTGGTAAAGAATGGGATCCGGTAATCTGGGATGGAGATGTGTGGGAGGACATAGATTCTGATGGGACTGAGGAAGCAGAGTTGACCACTGTCTTGGCCTCTCCATCCTTGTCTGAGGAAAGTGGTTATGCCTTGTCAAAAGAACGCGCCCAGCAGGACAAAGCAGATGCTCCTCAGAACCAGTCTTCAACATCCTTAGTTACTTCTGAACCTGTCACCAGACCCAAGTCTCTGTCTGACCTTACAAGTCAGAAACACCGCCATACTAACCATGAACTTAAATCACTTGCTCAGTCAAATCGCCAACAGGCAAAGGAACATTCGAGGAAATGGATTTTAAGGGTGTGGGATAATGGTGGGAGGCTCACAATACTGGATCAGACTGAATTTCTCAGTTTAGGTCCTTTGAGCCTTGATAGTGAGTTTAATGTCATAGCCCGCACGGTTGAAGATAACGGTGTGAAGAGTTTGTTTGATTGGTTGGCTGAAGCATGGGTCCAGAGATGGCCTACTACAAGAGAGCTGCAGTCGCCTGACGCCCTGGAGTGGTATTCTATTGAGGATGGGATTAAAAGGCTTAGGGAACTTGGAATGATAGAGTGGCTTTGTGTAAAAGCTACTTGTCCACAATGGAGGGGCCCGGAAGATGCACCCATCACGAGAGCTGTGAGGATAACTTTTGTCCGGGAAACTGTAGAGACTTGGAAGAGCTTCGTATTTAGCCTCCTCTGTATAAAGGACATAACAGTGGGGGGCGTGGCTGCCCAGTTGCATGATCTAATAGAATTAAGTTTAAAGCCAACAGCAGCTGGCTTGACTGCTGGTTCGGCTCCTGTGGGCTCCGTGGCGGTTCTCTCTCTCTCTCCCTGGAAACATCAAAGCAACAGTTAA

>Fv7CAR6 MT077228 [organism=Mus caroli]

ATGAATTTCCCACGTGCGCTTGCTGGTTTCTCAAGCTGGCTCTTCAAACCTGAACTTGCCGAGGACACTCCGGATAATGACGAGAACACTGTTAACCCATGGCGTGAGCTGCTGCAGAAGATAAATGTGGCCGATCTCCCCGATTCATCCTTTTCGAGCGGTAAGGAACTTAATGTCTCTGTGTACGATACTTTTGAACATTTCTGCAAGATTAGGGACTATGACGCAGTTGGCGAGCTGCTTCTGGCATTTCTGGATAAAGTAACAAAGGAAAGGGACCAATTCAGAGATGAAATTTCCCAGCTCCGAATGCACATAAATGATCTAAAGGCTTCTAAGTGTGTCCTGGGGGAGACTCTTCTCTCCTACCGCCACAGGATTGAAGTTGGGGAAAAACAGACTGAAGCCCTCATTGTGAGGTTAGCTGATTTGCAATCCCAGGTCATGTGTCAGCCTGCCCGGAAAGTGTCTGCAGATAAGGTGAGGGCACTGATTGGTAAAGAATGGGATCCGATAACCTGGGATGGAGATGTGTGGGAGGACATAGATTCTGATGGGACTGAGGAAGCAGAGTTGACCACTGTCTTGGCCTCTCCATCCTTGTCTGAGGAAAGTGGTTATGCCTTGTCAAAAGAACGCGCCCAGCAGGACAAAGCAGATGCTCCTCAGAACCAGTCTTCAACATCCTTAGTTACTTCTGAACCTGTCACCAGACCCAAGTCTCTGTCTGACCTTACAAGTCAGAAACACCGCCATACTAACCATGAACTCAAATCACTTGCTCAATCAAATCGCCAACAGGCAAAGGAACATGCGAGGAAATGGATTTTAAGGGTGTGGGATAATGGTGGGAGGCTCACAATACTGGATCAGACTGAATTTCTCAGTTTAGGTCCTTTGAGCCTTGATAGTGAGTTTAATGTCATAGCCCGCACGGTTGAAGATAACGGTGTGAAGAGTTTGTTTGATTGGTTGGCTGAAGCATGGGTCCAGAGATGGCCTACTACAAGAGAGCTGCAGACGCCTGACGCCCTGGAGTGGTATTCTATTGAGGATGGGATTAAAAGGCTTAGGGAACTTGGAATGATAGAGTGGCTTTGTGTAAAAGCTACTTGTCCACAGTGGAGGGGCCCGGAAGATGCACCCATCACGAGAGCTATGAGGATAACTTTTGTCCGGGAAACTGTAGAGACTTGGAAGAGCTTCGTATTTAGCCTCCTCTGTATAAAGGACATAACAGTGGGGGGCGTGGCTGCCCAGTTGCATGATCTAATAGAATTAAGTTTAAAGCCAACAGCAGCTGGCTTGACTGCTGGTTCGGCTCCTGTGGGCTCCGTGGCGGTTCTCTCTCTCTCTCCCTGGAAACATCAAAGCAACAGTTAA

>Fv7CAR7 MT077229 [organism=Mus caroli]

ATGAATTTCCCACGTGCGCTTGCTGGTTTCTCGAGCTGGCTCTTCAAACCTGAACTTGCTGAGGACTCTCCGGATAATGACGAGAACATTGTTAACCCATGGCGTGATCTGCTGCAGAAGATAAATGTGGCCGATCTCCCCGATTCATCCTTTTCGAGCGGTAAGGAACTTAATGACTCTGTGTACGATACTTTTGAACATTTCTGCAAGATTAGGGACTATGACGCAGTTGGCGAGCTGCTTCTGGCATTTCTGGATAAAGTAACAAAGGAAAGGGACCAATTCAGAGATGAAATTTCCCAGCTCCGAATGCACATAAATGATCTAAAGGCTTCTAAGTGTGTCCTGGGGGAGACTCTTCTCTCCTACCGCCACAGGATTGAAGTTGGGGAAAAACAGACTGAAGCCCTCATTGTGAGGTTAGCTGATGTGCAATCCCAGGTCATGTGTCAGCCTGCCCGGAAAGTGTCTGCAGATAAGGTGAGGGCACTGATTGGTAAAGAATGGGATCCGGTAACCTGGGATGGAGATGTGTGGGAGGACATAAATTCTGATGGGACTGAGGAAGCAGTGTTGACCACTGTCTTGGCCTCTCCATCCTTGTCTGAGGAAAGTGGTTATGCCTTGTCAAAAGAACGCGCCCAGCAGGACAAAGCAGATGCTCCTCAGAACCAGTCTTCAACATCCTTAGTTACTTCTGAACCTGTCACCAGACCCAAGTCTCTGTCTGACCTTACAAGTCAGAAACACCGCCATACTAACCATGAACTCAAATCACTTGCTCAGTCAAATCGCCAACAGGCAAAGGAACATTCGAGGAAATGGATTTTAAGGGTGTGGGATAATGGTGGGAGGCTCACAATACTGGATCAGACTGAATTTCTCAGTTTAGGTCCTTTGAGCCTTGATAGTGAGTTTAATGTCATAGCCCGCACGGTTGAAGATAACGGTGTGAAGAGTTTGTTTGATTGGTTGGCTGAAGCATGGGTCCAGAGATGGCCTACTACAAGAGAGCTGCAGTCGCCTGACGCCCTGGAGTGGTATTCTATTGAGGATGGGATTAAAAGGCTTAGGGAACTTGGAATGATAGAGTGGCTTTGTGTAAAAGCTACTTGTCCACAATGGAGGGGCCCGGAAGATGCACCCATCACGAGAGCTATGAGGATAACTTTTGTCCGGGAAACTGTAGAGACTTGGAAGAGCTTCGTATTTAGCCTCCTCTGTATAAAGGACATAACAGTGGGGGGCGTGGCTGCCCAGTTGCATGATCTAATAGAATTAAGTTTAAAGCCAACAGCAGCTGGCTTGACTGCTGGTTCGGCTCCTGTGGGCTCCGTGGCGGTTCTCTCTCTCTCTCCCTGGAAACATCAAAGCAACAGTTAA

>Fv7CAR8 MT077230 [organism=Mus caroli]

ATGAATTTCCCACGTGCGCTTGCTGGTTTCTCAAGCTGGCTCTTCAAACCTGAACTTGCCGAGGACACTCCGGATAATGACGAGAACACTGTTAACCCATGGCGTGAGCTGCTGCAGAAGATAAATGTGGCCGATCTCCCCGATTCATCCTTTTCGAGCGGTAAGGAACTTAATGTCTCTGTGTACGATACTTTTGAACATTTCTGCAAGATTAGGGACTATGACGCAGTTGGCGAGCTGCTTCTGGCATTTCTGGATAAAGTAACAAAGGAAAGGGACCAATTCAGAGATGAAATTTCCCAGCTCCGAATGCACATAAATGATCTAAAGGCTTCTAAGTGTGTCCTGGGGGATACTCTTCTCTCCTACCGCCACAGGATTGAAGTTGGGGAAAAACAGACTGAAGCCCTCATTGTGAGGTTAGCTGATTTGCAATCCCAGGTCATGTGTCAGCCTGCCCGGAAAGTGTCTGCAGATAAGGTGAGGGCACTGATTGGTAAAGAATGGGATCCGGTAACCTGGGATGGAGATGTGTGGGAGGATATAGATTCTGATGGGACTGAGGAAGCAGAGTTGACCACTGTCTTGGCCTCTCCATCCTTGTCTGAGGAAAGTGGTTATGCCTTGTCAAAAGAACGCGCCCAGCAGGACAAAGCAGATGCTCCTCAGAACCAGTCTTCAACATCCTTAGTTACTTCTGAACCTGTCACCAGACCCAAGTCTCTGTCTGACCTTACAAGTCAGAAACACCGCCATACTAACCATGAACTCAAATCACTTGCTCAATCAAATCGCCAACAGGCAAAGGAGCATGCGAGGAAATGGATTTTAAGGGTGTGGGATAATGGTGGGAGGCTCACAATACTGGATCAGGCTGAATTTCTCAGTTTAGGTCCTTTGAGCCTTGATAGTGAGTTTAATGTCATAGCCCGCACGGTTGAAGATAACGGTGTGAAGAGTTTGTTTGATTGGTTGGCTGAAGCATGGGTCCAGAGATGGCCTACTACAAGAGAGCTGCAGACGCCTGACGCCCTGGAGTGGTATTCTATTGAGGATGGGATTAAAAGGCTTAGGGAACTTGGAATGATAGAGTGGCTTTGTGTAAAAGCTACTTGTCCACAGTGGAGGGGCCCGGAAGATGCACCCATCACGAGAGCTATGAGGATAACTTTTGTCCGGGAAACTGTAGAAACTTGGAAGAGCTTCGTATTTAGCCTCCTCTGTATAAAGGACATAACAGTGGGGGGCGTGGCTGCCCAGTTGCATGATCTAATAGAATTAAGTTTAAAGCCAACAGCAGCTGGCTTGACTGCTGGTTCGGCTCCTGTGGGCTCCGTGGCGGTTCTCTCTCTCTCCCCCTGGAAACATCAAAGCAACAGTTAA

>Fv7CAR9 MT077231 [organism=Mus caroli]

ATGAATTTCCCACGTGCGCTTGCTGGTTTCTCGAGCTGGCTCTTCAAACCTGAACTTGCCGAGGACTCTCCGGATAATGACGAGAACACTGTTAACCCATGGCGTGAGCTGCTGCAGAAGATAAATGTGGCCGATCTCCCCGATTCATCCTTTTCGAGCGGTAAGGAACTTAATGACTCTGTGTACGATACTTTTGAACATTTCTGCAAGATTAGGGACTATGACGCAGTTGGCGAGCTGCTTCTGGCATTTCTGGATAAAGTAACAAAGGAAAGGGACCAATTCAGAGATGAAATTTCCCAGCTCCGAATGCACATAAATGATCTGAAGGCTTCTAAGTGTGTCCTGGGGGAGACTCTTCTCTCCTACCGCCACAGGATTGAAGTTGGGGAAAAACAGACTGAAGCCCTCATTGTGAGGTTAGCTGATGTGCAATCCCAGGTCATGTGTCAGCCTGCCCGGAAAGTGTCTGCAGATAAGGTGAGGGCACTGATTGGTAAAGAATGGGATCCGGTAACCTGGGATGGAGATGTGTGGGAGGACATAGATTCTGAGGGGACTGAGGAAGCAGAGTTGACCACTGTCTTGGCCTCTCCATCCTTGTCTGAGGAAAGTGGTTATGCCTTGTCTAAAGAACGCACCCAGCAGGACAAAGCAGATGCTTCTCAGAACCAGTCTTCAACATCCTTAGTTACTTCTGAACCTGTCACCAGACCCAAGTCTCTGTCTGACCTTACAAGTCAGAAACACCGCCATACTAACCATGAACTCAAATCACTTGCTCAGTCAAATCACCAACAGGCAAAGGAACATGCGAGGAAATGGATTTTAAGGGTGTGGGATAATGGTGGGAGGCTCACAATACTGGATCAGGCTGAATTTCTCAGTTTAGGTCCTTTGAGCCTTGATAGTGAGTTTAATGTCATAGCCCGCACTGTTGAAGGTAACGGTGTGAAGAGTTTGTTTGATTGGTTGGCTGAAGCATGGGTCCAGAGATGGCCTACTACAAGAGAGCTGCAGACGCCTGACGCCCTGGAGTGGTATTCTATTGAGGATGGGATTAAAAGGCTTAGGGAACTTGGAATGATAGAGTGGCTTTGTGTAAAAGCTACTTGTCCACAGTGGAGGGGTCCGGAAGATGCACCCATCACGAGAGCTATGAGGATAACTTTTGTCCGGGAAACTGTAGAGACTTGGAAGAGCTTCGTATTTAGCCTCCTCTGTATAAAGGACATAACAGTGGGGAGCGTGGCTGCCCAGTTGCATGATCTATTAGAATTGAGTTTAAAGCCAACAGCAGCTGGCTTGACTGCTGGTTCGGCTCCTGTGGGCTCGGTGGCGGTTCTCTCTCTCTCTCCCTGGAAACATCAAAGCAACAGTTAA

>Fv7CAR10 MT077232 [organism=Mus caroli]

ATGAATTTCCCACGTGCGCTTGCTGGTTTCTCGAGCTGGCTCTTCAAACCTGAACTTGCTGAGGACTCTCCGGATAATGACGAGAACATTGTTAACCCATGGCGTGAGCTGCTGCAGAAGATAAATGTGGCCAATCTCCCCGATTCATCCTTTTCGAGCGGTAAGGAACTTAATGACTCTGTGTACGATACTTTTGAACATTTCTGCAAGATTAGGGACTATGACGCAGTTGGCGAGCTGCTTCTGGCATTTCTGGATAAAGTAACAAAGGAAAGGGACCAATTCAGAGATGAAATTTCCCAGCTCCGAATGCACATAAATGATCTAAAGGCTTCTAAGTGTGTCCTGGGGGAGACTCTTCTCTCCTACCGCCACAGGATTGAAGTTGGGGAAAAACAGACTGAAGCCCTCATTGTGAGGTTAGCTGATTTGCAATCCCAGGTCATGTGTCAGCCTGCCCGGAAAGTGTCTGCAGATAAGGTGAGGGCACTGATTGGTAAAGAATGGGATCCGGTAACCTGGGATGGAGATGTGTGGGAGGACATAGATTCTGATGGGACTGAGGAAGCAGAGTTGACCACTGTCTTGGCCTCTCCATCCTTGTCTGAGGAAAGTGGTTATGCCTTGTCAAAAGAACGCGCCCAGCAGGACAAAGCAGATGCTCCTCAGAACCAGTCTTCAACATCCTTAGTTACTTCTGAACCTGTCACCAGACCCAACTCTCTGTCTGACCTTACAAGTCAGAAACACCGCCATACTAACCATGAACTCAAATCACTTGCTCAATCAAATCGCCAACAGGCAAAGGAGCATGCGAGGAAATGGATTTTAAGGGTGTGGGATAATGGTGGGAGGCTCACAATACTGGATCAGGCTGAATTTCTCAGTTTAGGTCCTTTGAGCCTTGATAGTGAGTTTAATGTCATAGCCCGCACGGTTGAAGATAACGGTGTGAAGAGTTTGTTTGATTGGTTGGCTGAAGCATGGGTCCAGAGATGGCCTACTACAAGAGAGCTGCAGACGCCTGACGCCCTGGAGTGGTATTCTATTGAGGATGGGATTAAAAGGCTTAGGGAACTTGGAATGATAGAGTGGCTTTGTGTAAAAGCTACTTGTCCACAGTGGAGGGGCCCGGAAGATGCACCCATCACGAGAGCTATGAGGATAACTTTTGTCCGGGAAACTGTAGAGACTTGGAAGAGCTTCGTATTTAGCCTCCTCTGTATAAAGGACATAACAGTGGGGGGCGTGGCTGCCCAGTTGCATGATCTAATAGAATTAAGTTTAAAGCCAACAGCAGCTGGCTTGACTGCTGGTTCGGCTCCTGTGGGCTCCCTGGCGGTTCTCTCTCTCTCTCCCTGGAAACATCAAAGCAACAGTTAA

>Fv1CER1 MT077233 [organism=Mus cervicolor]

ATGAATTTCCCACGTGCGCTTGCTGGTTTCTCGAGCTGGCTCTTCAAACCTGAACTTGCCGAGGACTCTCCGGATAATGACGAGAACACTGTTAACCCATGGCGTGAGCTGCTGCAGAAGATAAATGTGGCCGATCTCCCCGATTCATCCTTTTCGAGCGGTAAGGAACTTAATGACTCTGTGTACGATACTTTTGAACATTTCTGCAAGACTAGGGACTATAACGCAGTTGGCGAGCTGCTTCTGGCATTTCTGGATAAAGTAACAAAGGAAAGGGACCAATTCAGAGATGAAATTTCCCAGCTCCGAATGCACATAAATGATCTAAAGGCTTCTAAGTGTGTCCTGGGGGAGACTCTTCTATCCTACCGCCACAGGATTGAAGTTGGGGAAAAACAGACTGAAGCCCTCATTGTGAGGTTAGCTGATGTGCAATCCCAGGTCATGTGTCAGCCTGCCCGGAAAGTGTCTGCAGATAAGGTGAGGGCACTGATTGGTAAAGAATGGGATCCCGTTACCTGGGATGGAGATGTGTGGGAGGACATAGATTCTGAGGGAACTGAGGAAGCAGAGTTGCCCACTGTCTTGGCCTCTCCATCCTTGTCTGAGGAAAGTGGTTATGCCTTGTCTAAAGAACGCACCCAGCAGGACAAAACAGATGCTCCTCAGAACCAGTCTTCAACATCCTTAGTTACTTCTGAACCTGTCACCAGACCCAAGTCTCTGTCTGACCTTACAAGTCAGAAACACCGCCATACTAACCATGAACTCTATTCACTTGCTCAATCAAATCGCCAACAGGAAAAGGAACATGCTAGGAAATGGATTTTAAGGGTGTGGGATAATGGTGGGAGGCTCACAATACTGGATCAGATTGAATTTCTCAGTTTAGGTCCTTTGAGCCGAGATAGTGAGTTTAATGTCATAGCCCGCACTGTTGCAGATAATGGTGTGAAGAGTTTGTTTGATTGGTTGGCTGAAGCATGGGTCCAGAGATGGCCTACTACAAGAGAGCTACAGACGCCTGACGCCCTGGAGTGGTATTCTATTGAGGATGGGATTAAAAGGCTTAGGGAACTTGGAATGATAGAGTGGCTTTGTGTAAAAGCTACTTGTCCACAGTGGAGGGGCCCGGAAGATGCACCCATCACGAGAGCTATGAGGATAACTTTTGTCCAGGAAACTGTAGAGACTTGGAAGAGCTTCGTATTTAGCCTCCTCTGTATAAAGGACATAACAGTGGGGAGCGTGGCTGCTCAGTTGCATGATCTAACAGAATTACGTTTAAAGCCAGCCGGGGGGCGTGGTGGCGCACGCTTTTAA

>Fv1CER2 MT077234 [organism=Mus cervicolor]

ATGAATTTCCCACGTGCGCTTGCTGGTTTCTCGAGCTGGCTCTTCAAACCTGAACTTGCCGAGGACTCTCCGGATAATGACGAGAACACTGTTAACCCATGGCGTGAGCTGCTGCAGAAGATAAATGTGGCCGATCTCCCCGATTCATCCTTTTCGAGCGGTAAGGAACTTAATGACTCTGTGTACGATACTTTTGAACATTTCTGCAAGACTAGGGACTATAACGCAGTTGGCGAGCTGCTTCTGGCATTTCTGGATAAAGTAACAAAGGAAAGGGACCAATTCAGAGATGAAATTTCCCAGCTCCGAATGCACATAAATGATCTAAAGGCTTCTAAGTGTGTCCTGGGGGAGACTCTTCTATCCTACCGCCACAGGATTGAAGTTGGGGAAAAACAGACTGAAGCCCTCATTGTGAGGTTAGCTGATGTGCAATCCCAGGTCATGTGTCAGCCTGCCCGGAAAGTGTCTGCAGATAAGGTGAGGGCACTGATTGGTAAAGAATGGGATCCCGTTACCTGGGATGGAGATGTGTGGGAGGACATAGATTCTGAGGGAACTGAGGAAGCAGAGTTGCCCACTGTCTTGGCCTCTCCATCCTTGTCTGAGGAAAGTGGTTATGCCTTGTCTAAAGAACGCACCCAGCAGGACAAAACAGATGCTCCTCAGAACCAGTCTTCAACATCCTTAGTTACTTCTGAACCTGTCACCAGACCCAAGTCTCTGTCTGACCTTACAAGTCAGAAACACCGCCATACTAACCATGAACTCTATTCACTTGCTCAATCAAATCGCCAACAGGAAAAGGAACATGCTAGGAAATGGATTTTAAGGGTGTGGGATAATGGTGGGAGGCTCACAATACTGGATCAGATTGAATTTCTCAGTTTAGGTCCTTTGAGCCGAGATAGTGAGTTTAATGTCATAGCCCGCACTGTTGCAGATAATGGTGTGAAGAGTTTGTTTGATTGGTTGGCTGAAGCATGGGTCCAGAGATGGCCTACTACAAGAGAGCTACAGACGCCTGACGCCCTGGAGTGGTATTCTATTGAGGATGGGATTAAAAGGCTTAGGGAACTTGGAATGATAGAGTGGCTTTGTGTAAAAGCTACTTGTCCACAGTGGAGGGGCCCGGAAGATGCACCCATCACGAGAGCTATGAGGATAACTTTTGTCCAGGAAACTGTAGAGACTTGGAAGAGCTTCGTATTTAGCCTCCTCTGTATAAAGGACATAACAGTGGGGAGCGTGGCTGCTCAGTTGCATGATCTAACAGAATTAAGTTTAAAGCCAGCCGGGGGGCGTGGTGGCGCACGCTTTTAA

>Fv1CER3 MT077235 [organism=Mus cervicolor]

ATGAATTTCCCACGTGCGCTTGCTGGTTTCTCGAGCTGGCTCTTCAAACCTGAACTTGCCGAGGACTCTCCGGATAATGACGAGAACACTGTTAACCCATGGCGTGAGCTGCTGCAGAAGATAAATGTGGCCGATCTCCCCGATTCATCCTTTTCGAGCGGTAAGGAACTTAATGACTCTGTGTACGATACTTTTGAACATTTCTGCAAGACTAGGGACTATAACGCAGTTGGCGAGCTGCTTCTGGCATTTCTGGATAAAGTAACAAAGGAAAGGGACCAATTCAGAGATGAAATTTCCCAGCTCCGAATGCACATAAATGATCTAAAGGCTTCTAAGTGTGTCCTGGGGGAGACTCTTCTATCCTACCGCCACAGGATTGAAGTTGGGGAAAAACAGACTGAAGCCCTCATTGTGAGGTTAGCTGATGTGCAATCCCAGGTCATGTGTCAGCCTGCCCGGAAAGTGTCTGCAGATAAGGTGAGGGCACTGATTGGTAAAGAATGGGATCCCGTTACCTGGGATGGAGATGTGTGGGAGGACATAGATTCTGAGGGAACTGAGGAAGCAGAGTTGCCCACTGTCTTGGCCTCTCCATCCTTGTCTGAGGAAAGTGGTTATGCCTTGTCTAAAGAACGCACCCAGCAGGACAAAACAGATGCTCCTCAGAACCAGTCTTCAACATCCTTAGTTACTTCTGAACCTGTCACCAGACCCAAGTCTCTGTCTGACCTTACAAGTCAGAAACACCGCCATACTAACCATGAACTCTATTCACTTGCTCAATCAAATCGCCAACAGGAAAAGGAACATGCTAGGAAATGGATTTTAAGGGTGTGGGATAATGGTGGGAGGCTCACAATACTGGATCAGATTGAATTTCTCAGTTTAGGTCCTTTGAGCCGAGATAGTGAGTTTAATGTCATAGCCCGCACTGTTGCAGATAATGGTGTGAAGAGTTTGTTTGATTGGTTGGCTGAAGCATGGGTCCAGAGATGGCCTACTACAAGAGAGCTACAGACGCCTGACGCCCTGGAGTGGTATTCTATTGAGGATGGGATTAAAAGGCTTAGGGAACTTGGAATGATAGAGTGGCTTTGTGTAAAAGCTACTTGTCCACAGTGGCCACAGTGGAGGGGCCCGGAAGATGCACCCATCACGAGAGCTATGAGGATAACTTTTGTCCAGGAAACTGTAGAGACTTGGAAGAGCTTCGTATTTAGCCTCCTCTGTATAAAGGACATAACAGTGGGGAGCGTGGCTGCTCAGTTGCATGATCTAACAGAATTAAGTTTAAAGCCAGCCGGGGGGCGTGGTGGCGCACGCTTTTAA

>Fv1CER4 MT077236 [organism=Mus cervicolor]

ATGAATTTCCCACGTGCGCTTGCTGGTTTCTCGAGCTGGCTCTTCAAACCTGAACTTGCCGAGGACTCTCCGGATAATGACGAGAACACTGTTAACCCATGGCGTGAGCTGCTGCAGAAGATAAATGTGGCCGATCTCCCCGATTCATCCTTTTCGAGCGGTAAGGAACTTAATGACTCTGTGTACGATACTTTTGAACATTTCTGCAAGACTAGGGACTATAACGCAGTTGGCGAGCTGCTTCTGGCATTTCTGGATAAAGTAACAAAGGAAAGGGACCAATTCAGAGATGAAATTTCCCAGCTCCGAATGCACATAAATGATCTAAAGGCTTCTAAGTGTGTCCTGGGGGAGACTCTTCTATCCTACCGCCACAGGATTGAAGTTGGGGAAAAACAGACTGAAGCCCTCATTGTGAGGTTAGCTAATGTGCAATCCCAGGTCATGTGTCAGCCTGCCCGGAAAGTGTCTGCAGATAAGGTGAGGGCACTGATTGGTAAAGAATGGGATCCCGTTACCTGGGATGGAGATGTGTGGGAGGACATAGATTCTGAGGGAACTGAGGAAGCCGAGTTGCCCACTGTCTTGGCCTCTCCATCCTTGTCTGAGGAAAGTGGTTATGCCTTGTCTAAAGAACGCACCCAGCAGGACAAAACAGATGCTCCTCAGAACCAGTCTTCAACATCCTTAGTTACTTCTGAACCTGTCACCAGACCCAAGTCTCTGTCTGACCTTACAAGTCAGAAACACCGCCATACTAACCATGAACTCTATTCACTTGCTCAATCAAATCGCCAACAGGAAAAGGAACATGCTAGGAAATGGATTTTAAGGGTGTGGGATAATGGTGGGAGGCTCACAATACTGGATCAGATTGAATTTCTCAGTTTAGGTCCTTTGAGCCGAGATAGTGAGTTTAATGTCATAGCCCGCACTGTTGCAGATAATGGTGTGAAGAGTTTGTTTGATTGGTTGGCTGAAGCATGGGTCCAGAGATGGCCTACTACAAGAGAGCTACAGACGCCTGACGCCCTGGAGTGGTATTCTATTGAGGATGGGATTAAAAGGCTTAGGGAACTTGGAATGATAGAGTGGCTTTGTGTAAAAGCTACTTGTCCACAGTGGAGGGGCCCGGAAGATGCACCCATCACGAGAGCTATGAGGATAACTTTTGTCCAGGAAACTGTAGAGACTTGGAAGAGCTTCGTATTTAGCCTCCTCTGTATAAAGGACATAACAGTGGGGAGCGTGGCTGCTCAGTTGCATGATCTAACAGAATTAAGTTTAAAGCCAGCCGGGGGGCGTGGTGGCGCACGCTTTTAA

>Fv1CER5 MT077237 [organism=Mus cervicolor]

ATGAATTTCCCACGTGCGCTTGCTGGTTTCTCGAGCTGGCTCTTCAAACCTGAACTTGCCGAGGACTCTCCGGATAATGACGAGAACACTGTTAACCCATGGCGTGAGCTGCTGCAGAAGATAAATGTGGCCGATCTCCCCGATTCATCCTTTTCGAGCGGTAAGGAACTTAATGACTCTGTGTACGATACTTTTGAACATTTCTGCAAGACTAGGGACTATAACGCAGTTGGCGAACTGCTTCTGGCATTTCTGGATAAAGTAACAAAGGAAAGGGACCAATTCAGAGATGAAATTTCCCAGCTCCGAATGCACATAAATGATCTAAAGGCTTCTAAGTGTGTCCTGGGGGAGACTCTTCTATCCTACCGCCACAGGATTGAAGTTGGGGAAAAACAGACTGAAGCCCTCATTGTGAGGTTAGCTGATGTGCAATCCCAGGTCATGTGTCAGCCTGCCCGGAAAGTGTCTGCAGATAAGGTGAGGGCACTGATTGGTAAAGAATGGGATCCCGTTACCTGGGATGGAGATGTGTGGGAGGACATAGATTCTGAGGGAACTGAGGAAGCAGAGTTGCCCACTGTCTTGGCCTCTCCATCCTTGTCTGAGGAAAGTGGTTATGCCTTGTCTAAAGAACGCACCCAGCAGGACAAAACAGATGCTCCTCAGAACCAGTCTTCAACATCCTTAGTTACTTCTGAACCTGTCACCAGACCCAAGTCTCTGTCTGACCTTACAAGTCAGAAACACCGCCATACTAACCATGAACTCTATTCACTTGCTCAATCAAATCGCCAACGGGAAAAGGAACATGCTAGGAAATGGATTTTAAGGGTGTGGGATAATGGTGGGAGGCTCACAATACTGGATCAGATTGAATTTCTCAGTTTAGGTCCTTTGAGCCGAGATAGTGAGTTTAATGTCATAGCCCGCACTGTTGCAGATAATGGTGTGAAGAGTTTGTTTGATTGGTTGGCTGAAGCATGGGTCCAGAGATGGCCTACTACAAGAGAGCTACAGACGCCTGACGCCCTGGAGTGGTATTCTATTGAGGATGGGATTAAAAGGCTTAGGGAACTTGGAATGATAGAGTGGCTTTGTGTAAAAGCTACTTGTCCACAGTGGAGGGGCCCGGAAGATGCACCCATCACGAGAGCTATGAGGATAACTTTTGTCCAGGAAACTGTAGAGACTTGGAAGAGCTTCGTATTTAGCCTCCTCTGTATAAAGGACATAACAGTGGGGAGCGTGGCTGCTCAGTTGCATGATCTAACAGAATTAAGTTTAAAGCCAGCCGGGGGGCGTGGTGGCGCACGCTTTTAA

>Fv1CER6 MT077238 [organism=Mus cervicolor]

ATGAATTTCCCACGTGCGCTTGCTGGTTTCTCGAGCTGGCTCTTCAAACCTGAACTTGCCGAGGACTCTCCGGATAATGACGAGAACACTGTTAACCCATGGCGTGAGCTGCTGCAGAAGATAAATGTGGCCGATCTCCCCGATTCATCCTTTTCGAGCGGTAAGGAACTTAATGACTCTGTGTACGATACTTTTGAACATTTCTGCAAGACTAGGGACTATAACGCAGTTGGCGAGCTGCTTCTGGCATTTCTGGATAAAGTAACAAAGGAAAGGGACCAATTCAGAGATGAAATTTCCCAGCTCCGAATGCACATAAATGATCTAAAGGCTTCTAAGTGTGTCCTGGGGGAGACTCTTCTATCCTACCGCCACAGGATTGAAGTTGGGGAAAAACAGACTGAAGCCCTCATTGTGAGGTTAGCTGATGTGCAATCCCAGGTCATGTGTCAGCCTGCCCGGAAAGTGTCTGCAGATAAGGTGAGGGCACTGATTGGTAAAGAATGGGATCCCGTTACCTGGGATGGAGATGTGTGGGAGGACATAGATTCTGAGGGAACTGAGGAAGCAGAGTTGCCCACTGTCTTGGCCTCTCCATCCTTGTCTGAGGAAAGTGGTTATGCCTTGTCTAAAGAACGCACCCAGCAGGACAAAACAGATGCTCCTCAGAACCAGTCTTCAACATCHTTAGTTACTTCTGAACCTGTCACCAGACCCAAGTCTCTGTCTGACTTTACAAGTCAGAAACACCGCCATACTAACCATGAACTCTATTCACTTGCTCAATCAAATCGCCAACAGGAAAAGGAACATGCTAGGAAATGGATTTTAAGGGTGTGGGATAATGGTGGGAGGCTCACAATACTGGATCAGATTGAATTTCTCAGTTTAGGTCCTTTGAGCCGAGATAGTGAGTTTAATGTCATAGCCCGCACTGTTGCAGATAATGGTGTGAAGAGTTTGTTTGATTGGTTGGCTGAAGCATGGGTCCAGAGATGGCSTACTACAAGAGAGCTACAGACGCCTGACGCCCTGGTGTGGTATTCTATTGAGGATGGGATTAAAAGGCTTAGGGAACTTGGAATGATAGAGTGGCTTTGTGTAAAAGCTACTTGTCCACAGTGGAGGGGCCCGGAAGATGCACCCATCACGAGAGCTATGAGGATAACTTTTGTCCAGGAAACTGTAGAGACTTGGAAGAGCTTCGTATTTAGCCTCCTCTGTATAAAGGACATAACAGTGGGGAGCGTGGCTGCTCAGTTGCATGATCTAACAGAATTAAGTTTAAAGCCAGCCGGGGGGCGTGGTGGCGCACGCTTTTAA

>Fv1CER7 MT077239 [organism=Mus cervicolor]

ATGAATTTCCCACGTGCGCTTGCTGGTTTCTCGAGCTGGCTCTTCAAACCTGAACTTGCCGAGGACTCTCCGGATAATGACGAGAACACTGTTAACCCATGGCGTGAGCTGCTGCAGAAGATAAATGTGGCCGATCTCCCCGATTCATCCTTTTCGAGCGGTAAGGAACTTAATGACTCTGTGTACGATACTTTTGAACATTTCTGCAAGACTAGGGACTATAACGCAGTTGGCGAGCTGCTTCTGGCATTTCTGGATAAAGTAACAAAGGAAAGGGACCAATTCAGAGATGAAATTTCCCAGCTCCGAATGCACATAAATGATCTAAAGGCTTCTAAGTGTGTCCTGGGGGAGACTCTTCTATCCTACCGCCACAGGATTGAAGTTGGGGAAAAACAGACTGAAGCCCTCATTGTGAGGTTAGCTGATGTGCAATCCCAGGTCATGTGTCAGCCTGCCCGGAAAGTGTCTGCAGATAAGGTGAGGGCACTGATTGGTAAAGAATGGGATCCCGTTACCTGGGATGGAGATGTGTGGGAGGACATAGATTCTGAGGGAACTGAGGAAGCAGAGTTGCCCACTGTCTTGGCCTCTCCATCCTTGTCTGAGGAAAGTGGTTATGCCTTGTCTAAAGAACGCACCCAGCAGGACAAAACAGATGCTCCTCAGAACCAGTCTTCAACATCCTTAGTTACTTCTGAACCTGTCACCAGACCCAAGTCTCTGTCTGACCTTACAAGTCAGAAACACCGCCATACTAACCATGAACTCTATTCACTTGCTCAATCAAATCGCCAACGGGAAAAGGAACATGCTAGGAAATGGATTTTAAGGGTGTGGGATAATGGTGGGAGGCTCACAATACTGGATCAGATTGAATTTCTCAGTTTAGGTCCTTTGAGCCGAGATAGTGAGTTTAATGTCATAGCCCGCACTGTTGCAGATAATGGTGTGAAGAGTTTGTTTGATTGGTTGGCTGAAGCATGGGTCCAGAGATGGCCTACTACAAGAGAGCTACAGACGCCTGACGCCCTGGAGTGGTATTCTATTGAGGATGGGATTAAAAGGCTTAGGGAACTTGGAATGATAGAGTGGCTTTGTGTAAAAGCTACTTGTCCACAGTGGAGGGGCCCGGAAGATGCACCCATCACGAGAGCTATGAGGATAACTTTTGTCCAGGAAACTGTAGAGACTTGGAAGAGCTTCGTATTTAGCCTCCTCTGTATAAAGGACATAACAGTGGGGAGCGTGGCTGCTCAGTTGCATGATCTAACAGAATTAAGTTTAAAGCCAGCCGGGGGGCGTGGTGGCGCACGCTTTTAA

>Fv1CER8 MT077240 [organism=Mus cervicolor]

ATGAATTTCCCACGTGCGCTTGCTGGTTTCTCGAGCTGGCTCTTCAAACCTGAACTTGCCGAGGACTCTCCGGATAATGACGAGAACACTGTTAACCCATGGCGTGAGCTGCTGCAGAAGATAAATGTGGCCGATCTCCCCGATTCATCCTTTTCGAGCGGTAAGGAACTTAATGACTCTGTGTACGATACTTTTGAACATTTCTGCAAGATTAGGGACTATGACGCAGTTGGCGAGCTGCTTCTGGCATTTCTGGATAAAGTAACAAAGGAAAGGGACCAATTCAGAGATGAAATTTCCCAGCTCCGAATGCACATAAATGATCTAAAGGCTTCTAAGTGTGTCCTGGGGGAGACTCTTCTCTCCTACCGCCACAGGATTGAAGTTGGGGAAAAACAGACTGAAGCCCTCATTGTGAGGTTAGCTGATGTGCAATCCCAGGTCATGTGTCAGCCTGCCCGGAAAGTGTCTGCAGATAAGGTGAGGGCACTGATTGGTAAAGAATGGGATCCCGTTACCTGGGATGGAGATGTGTGGGAGGACATAGATTCTGAGGGAACTGAGGAAGCAGAGTTGCCCACTGTCTTGGCCTCTCCATCCTTGTCTGAGGAAAGTGGTTATGCCTTGTCTAAAGAACGCACCCAGCAGGACAAAACAGATGCTCCTCAGAACCAGTCTTCAACATCCTTAGTTACTTCTGAACCTGTCACCAGACCCAAGTCTCTGTCTGACCTTACAAGTCAGAAACACCGCCATACTAACCATGAACTCTATTCACTTGCTCAATCAAATCGCCAACAGGAAAAGGAACATGCTAGGAAATGGATTTTAAGGGTGTGGGATAATGGTGGGAGGCTCACAATACTGGATCAGATTGAATTTCTCAGTTTAGGTCCTTTGAGCCGAGATAGTGAGTTTAATGTCATAGCCCGCACTGTTGCAGATAATGGTGTGAAGAGTTTGTTTGATTGGTTGGCTGAAGCATGGGTCCAGAGATGGCCTACTACAAGAGAGCTGCAGACGCCTGACGCCCTGGAGTGGTATTCTATTGAGGATGGGATTAAAAGGCTTAGGGAACTTGGAATGATAGAGTGGCTTTGTGTAAAAGCTACTTGTCCACAGTGGAGGGGCCCGGAAGATGCACCCATCACGAGAGCTATGAGGATAACTTTTGTCCAGGAAACTGTAGAGACTTGGAAGAGCTTCGTATTTAGCCTCCTCTGTATAAAGGACATAACAGTGGGGAGCGTGGCTGCTCAGTTGCATGATCTAACAGAATTAAGTTTAAAGCCAGCCGGGGGGCGTGGTGGCGCACGCTTTTAA

>Fv1CER9 MT077241 [organism=Mus cervicolor]

ATGAATTTCCCACGTGCGCTTGCTGGTTTCTCGAGCTGGCTCTTCAAACCTGAACTTGCCGAGGACTCTCCGGATAATGACGAGAACACTGTTAACCCATGGCGTGAGCTGCTGCAGAAGATAAATGTGGCCGATCTCCCCGATTCATCCTTTTCGAGCGGTAAGGAACTTAATGACTCTGTGTACGATACTTTTGAACATTTCTGCAAGACTAGGGACTATAACGCAGTTGGCGAGCTGCTTCTGGCATTTCTGGATAAAGTAACAAAGGAAAGGGACCAATTCAGAGATGAAATTTCCCAGCTCCGAATGCACATAAATGATCTAAAGGCTTCTAAGTGTGTCCTGGGGGAGACTCTTCTATCCTACCGCCACAGGATTGAAGTTGGGGAAAAACAGACTGAAGCCCTCATTGTGAGGTTAGCTGATGTGCAATCCCAGGTCATGTGTCAGCCTGCCCGGAAAGTGTCTGCAGATAAGGTGAGGGCACTGATTGGTAAAGAATGGGATCCCGTTACCTGGGATGGAGATGTGTGGGAGGACATAGATTCTGAGGGAACTGAGGAAGCAGAGTTGCCCACTGTCTTGGCCTCTCCATCCTTGTCTGAGGAAAGTGGTTATGCCTTGTCTAAAGAACGCACCCAGCAGGACAAAACAGATGCTCCTCAGAACCAGTCTTCAACATCCTTAGTTACTTCTGAACCTGTCACCAGACCCAAGTCTCTGTCTGACCTTACAAGTCAGAAACACCGCCATACTAACCATGAACTCTATTCACTTGCTCAATCAAATCGCCAACAGGAAAAGGAACATGCTAGGAAATGGATTTTAAGGGTGTGGGATAATGGTGGGAGGCTCACAATACTGGATCAGATTGAATTTCTCAGTTTAGGTCCTTTGAGCCGAGATAGTGAGTTTAATGTCATAGCCCGCACTGTTGCAGATAATGGTGTGAAGAGTTTGTTTGATTGGTTGGCTGAAGCATGGGTCCAGAGATGGCCTACTACAAGAGAGCTACAGACGCCTGACGCCCTGGTGTGGTATTCTATTGAGGATGGGATTAAAAGGCTTAGGGAACTTGGAATGATAGAGTGGCTTTGTGTAAAAGCTACTTGTCCACAGTGGAGGGGCCCGGAAGATGCACCCATCACGAGAGCTATGAGGATAACTTTTGTCCAGGAAACTGTAGAGACTTGGAAGAGCTTCGTATTTAGCCTCCTCTGTATAAAGGACATAACAGTGGGGAGCGTGGCTGCTCAGTTGCATGATCTAACAGAATTAAGTTTAAAGCCAGCCGGGGGGCGTGGTGGCGCACGCTTTTAA

>Fv1CER10 MT077242 [organism=Mus cervicolor]

ATGAATTTCCCACGTGCGCTTGCTGGTTTCTCGAGCTGGCTCTTCAAACCTGAACTTGCCGAGGACTCTCCGGATAATGACGAGAACACTGTTAACCCATGGCGTGAGCTGCTGCAGAAGATAAATGTGGCCGATCTCCCCGATTCATCCTTTTCGAGCGGTAAGGAACTTAATGACTCTGTGTACGATACTTTTGAACATTTCTGCAAGACTAGGGACTATAACGCAGTTGGCGAGCTGCTTCTGGCATTTCTGGATAAAGTAACAAAGGAAAGGGACCAATTCAGAGATGAAATTTCCCAGCTCCGAATGCACATAAATGATCTAAAGGCTTCTAAGTGTGTCCTGGGGGAGACTCTTCTATCCTACCGCCACAGGATTGAAGTTGGGGAAAAACAGACTGAAGCCCTCCCTCATTGTGAGGTTAGCTGATGTGCAATCCCAGGTCATGTGTCAGCCTGCCCGGAAAGTGTCTGCAGATAAGGTGAGGGCACTGATTGGTAAAGAATGGGATCCCGTTACCTGGGATGGAGATGTGTGGGAGGACATAGATTCTGAGGGAACTGAGGAAGCAGAGTTGCCCACTGTCTTGGCCTCTCCATCCTTGTCTGAGGAAAGTGGTTATGCCTTGTCTAAAGAACGCACCCAGCAGGACAAAACAGATGCTCCTCAGAACCAGTCTTCAACATCCTTAGTTACTTCTGAACCTGTCACCAGACCCAAGTCTCTGTCTGACCTTACAAGTCAGAAACACCGCCATACTAACCATGAACTCTATTCACTTGCTCAATCAAATCGCCAACAGGAAAAGGAACATGCTAGGAAATGGATTTTAAGGGTGTGGGATAATGGTGGGAGGCTCACAATACTGGATCAGATTGAATTTCTCAGTTTAGGTCCTTTGAGCCGAGATAGTGAGTTTAATGTCATAGCCCGCACTGTTGCAGATAATGGTGTGAAGAGTTTGTTTGATTGGTTGGCTGAAGCATGGGTCCAGAGATGGCCTACTACAAGAGAGCTACAGACGCCTGACGCCCTGGAGTGGTATTCTATTGAGGATGGGATTAAAAGGCTTAGGGAACTTGGAATGATAGAGTGGCTTTGTGTAAAAGCTACTTGTCCACAGTGGAGGGGCCCGGAAGATGCACCCATCACGAGAGCTATGAGGATAACTTTTGTCCAGGAAACTGTAGAGACTTGGAAGAGCTTCGTATTTAGCCTCCTCTGTATAAAGGACATAACAGTGGGGAGCGTGGCTGCTCAGTTGCATGATCTAACAGAATTAAGTTTAAAGCCAGCCGGGGGGCGTGGTGGCGCACGCTTTTAA

>Fv1CER11 MT077243 [organism=Mus cervicolor]

ATGAATTTCCCACGTGCGCTTGCTGGTTTCTCGAGCTGGCTCTTCAAACCTGAACTTGCCGAGGACTCTCCGGATAATGACGAGAACACTGTTAACCCATGGCGTGAGCTGCTGCAGAAGATAAATGTGGCCGATCTCCCCGATTCATCCTTTTCGAGCGGTAAGGAACTTAATGACTCTGTGTACGATACTTTTGAACATTTCTGCAAGATTAGGGACTATGACGCAGTTGGCGAGCTGCTTCTGGCATTTCTGGATAAAGTAACAAAGGAAAGGGACCAATTCAGAGATGAAATTTCCCAGCTCCGAATGCACATAAATGATCTAAAGGCTTCTAAGTGTGTCCTGGGGGAGACTCTTCTCTCCTACCGCCACAGGATTGAAGTTGGGGAAAAACAGACTGAAGCCCTCATTGTGAGGTTAGCTGATGTGCAATCCCAGGTCATGTGTCAGCCTGCCCGGAAAGTGTCTGCAGATAAGGTGAGGGCACTGATTGGTAAAGAATGGGATCCCGTTACCTGGGATGGAGATGTGTGGGAGGACATAGATTCTGATGGGACTGAGGAAGCAGAGTTGCCCACTGTCTTGGCCTCTCCATCCTTGTCTGAGGAAAGTGGTTATGCCTTGTCTAAAGAACGCACCCAGCAGGACAAAACAGATGCTCCTCAGAACCAGTCTTCAACATCCTTAGTTACTTCTGAACCTGTCACCAGACCCAAGTCTCTGTCTGACCTTACAAGTCAGAAACGCCGCCATACTAACCATGAACTCTATTCACTTGCTCAATCAAATCGCCAACAGGAAAAGGAACATGCTAGGAAATGGATTTTAAGGGTGTGGGATAATGGTGGGAGGCTCACAATACTGGATCAGATTGAATTTCTCAGTTTAGGTCCTTTGAGCCGAGATAGTGAGTTTAATGTCATAGCCCGCACTGTTGCAGATAATGGTGTGAAGAGTTTGTTTGATTGGTTGGCTGAAGCATGGGTCCAGAGATGGCCTACTACAAGAGAGCTACAGACGCCTGACGCCCTGGTGTGGTATTCTATTGAGGATGGGATTAAAAGGCTTAGGGAACTTGGAATGATAGAGTGGCTTTGTGTAAAAGCTACTTGTCCACAGTGGAGGGGCCCGGAAGATGCACCCATCACGAGAGCTATGAGGATAACTTTTGTCCAGGAAACTGTAGAGACTTGGAAGAGCTTCGTATTTAGCCTCCTCTGTATAAAGGACATAACAGTGGGGAGCGTGGCTGCTCAGTTGCATGATCTAACAGAATTAAGTTTAAAGCCAGCCGGGGGGCGTGGTGGCGCACGCTTTTAA

>Fv1CER12 MT077244 [organism=Mus cervicolor]

ATGAATTTCCCACGTGCGCTTGCTGGTTTCTCGAGCTGGCTCTTCAAACCTGAACTTGCCGAGGACTCTCCGGATAATGACGAGAACACTGTTAACCCATGGCGTGAGCTGCTGCAGAAGATAAATGTGGCCGATCTCCCCGATTCATCCTTTTCGAGCGGTAAGGAACTTAATGACTCTGTGTACGATACTTTTGAACATTTCTGCAAGACTAGGGACTATAACGCAGTTGGCGAGCTGCTTCTGGCATTTCTGGATAAAGTAACAAAGGAAAGGGACCAATTCAGAGATGAAATTTCCCAGCTCCGAATGCACATAAATGATCTAAAGGCTTCTAAGTGTGTCCTGGGGGAGACTCTTCTATCCTACCGCCACAGGATTGAAGTTGGGGAAAAACAGACTGAAGCCCTCATTGTGAGGTTAGCTGATGTGCAATCCCAGGTCATGTGTCAGCCTGCCCGGAAAGTGTCTGCAGATAAGGTGAGGGCACTGATTGGTAAAGAATGGGATCCCGTTACCTGGGATGGAGATGTGTGGGAGGACATAGATTCTGAGGGAACTGAGGAAGCAGAGTTGCCCACTGTCTTGGCCTCTCCATCCTTGTCTGAGGAAAGTGGTTATGCCTTGTCTAAAGAACGCACCCAGCAGGACAAAACAGATGCTCCTCAGAACCAGTCTTCAACATCCTTAGTTACTTCTGAACCTGTCACCAGACCCAAGTCTCTGTCTGACCTTACAAGTCAGAAACACCGCCATACTAACCATGAACTCTATTCACTTGCTCAATCAAATCACCAACGGGAAAAGGAACATGCTAGGAAATGGATTTTAAGGGTGTGGGATAATGGTGGGAGGCTCACAATACTGGATCAGATTGAATTTCTCAGTTTAGGTCCTTTGAGCCGAGATAGTGAGTTTAATGTCATAGCCCGCACTGTTGCAGATAATGGTGTGAAGAGTTTGTTTGATTGGTTGGCTGAAGCATGGGTCCAGAGATGGCCTACTACAAGAGAGCTACAGACGCCTGACGCCCTGGAGTGGTATTCTATTGAGGATGGGATTAAAAGGCTTAGGGAACTTGGAATGATAGAGTGGCTTTGTGTAAAAGCTACTTGTCCACAGTGGAGGGGCCCGGAAGATGCACCCATCACGAGAGCTATGAGGATAACTTTTGTCCAGGAAACTGTAGAGACTTGGAAGAGCTTCGTATTTAGCCTCCTCTGTATAAAGGACATAACAGTGGGGAGCGTGGCTGCTCAGTTGCATGATCTAACAGAATTACGTTTAAAGCCAGCCGGGGGGCGTGGTGGCGCACGCTTTTAA

>Fv1CER13 MT077245 [organism=Mus cervicolor]

ATGAATTTCCCACGTGCGCTTGCTGGTTTCTCGAGCTGGCTCTTCAAACCTGAACTTGCCGAGGACTCTCCGGATAACGACGAGAACACTGTTAACCCATGGCGTGAGCTGCTGCAGAAGATAAATGTGGCCGATCTCCCCGATTCATCCTTTTCGAGCGGTAAGGAACTTAATGACTCTGTGTACGATACTTTTGAACATTTCTGCAAGACTAGGGACTATGACGCAGTTGGCGAGCTGCTTCTGGCATTTCTGGATAAAGTAACAAAGGAAAGGGACCAATTCAGAGATGAAATTTCCCAGCTCCGAATGCACATAAATGATCTAAAGGCTTCTAAGTGTGTCCTGGGGGAGACTCTTCTATCCTACCGCCACAGGATTGAAGTTGGGGAAAAACAGACTGAAGCCCTCATTGTGAGGTTAGCTGATGTGCAATCCCAGGTCATGTGTCAGCCTGCCCGGAAAGTGTCTGCAGATAAGGTGAGGGCACTGATTGGTAAAGAATGGGATCCCGTTACCTGGGATGGAGATGTGTGGGAGGACATAGATTCTGAGGGAACTGAGGAAGCCGAGTTGCCCACTGTCTTGGCCTCTCCATCCTTGTCTGAGGAAAGTGGTTATGCCTTGTCTAAAGAACGCACCCAGCAGGACAAAACAGATGCTCCTCAGAACCAGTCTTCAACATCCTTAGTTACTTCTGAACCTGTCACCAGACCCAAGTCTCTGTCTGACCTTACAAGTCAGAAACACCGCCATACTAACCATGAACTCTATTCACTTGCTCAATCAAATCGCCAACAGGAAAAGGAACATGCTAGGAAATGGATTTTAAGGGTGTGGGATAATGGTGGGAGGCTCACAATACTGGATCAGATTGAATTTCTCAGTTTAGGTCCTTTGAGCCGAGATAGTGAGTTTAATGTCATAGCCCGCACTGTTGCAGATAATGGTGTGAAGAGTTTGTTTGATTGGTTGGCTGAAGCATGGGTCCAGAGATGGCCTACTACAAGAGAGCTACAGACGCCTGACGCCCTGGAGTGGTATTCTATTGAGGATGGGATTAAAAGGCTTAGGGAACTTGGAATGATAGAGTGGCTTTGTGTAAAAGCTACTTGTCCACAGTGGAGGGGCCCGGAAGATGCACCCATCACGAGAGCTATGAGGATAACTTTTGTCCAGGAAACTGTAGAGACTTGGAAGAGCTTCGTATTTAGCCTCCTCTGTATAAAGGACATAACAGTGGGGAGCGTGGCTGCTCAGTTGCATGATCTAACAGAATTAAGTTTAAAGCCAGCCGGGGGGCGTGGTGGCGCACGCTTTTAA

>Fv1CER14 MT077246 [organism=Mus cervicolor]

ATGAATTTCCCACGTGCGCTTGCTGGTTTCTCGAGCTGGCTCTTCAAACCTGAACTTGCCGAGGACTCTCCGGATAATGACGAGAACACTGTTAACCCATGGCGTGAGCTGCTGCAGAAGATAAATGTGGCCGATCTCCCCGATTCATCCTTTTCGAGCGGTAAGGAACTTAATGACTCTGTGTACGATACTTTTGAACATTTCTGCAAGATTAGGGACTATGACGCAGTTGGCGAGCTGCTTCTGGCATTTCTGGATAAAGTAACAAAGGAAAGGGACCAATTCAGAGATGAAATTTCCCAGCTCCGAATGCACATAAATGATCTAAAGGCTTCTAAGTGTGTCCTGGGGGAGACTCTTCTCTCCTACCGCCACAGGATTGAAGTTGGGGAAAAACAGACTGAAGCCCTCATTGTGAGGTTAGCTGATGTGCAATCCCAGGTCATGTGTCAGCCTGCCCGGAAAGTGTCTGCAGATAAGGTGAGGGCACTGATTGGTAAAGAATGGGATCCCGTTACCTGGGATGGAGATGTGTGGGAGGACATAGATTCTGAGGGAACTGAGGAAGCAGAGTTGCCCACTGTCTTGGCCTCTCCATCCTTGTCTGAGGAAAGTGGTTATGCCTTGTCTAAAGAACGCACCCAGCAGGACAAAACAGATGCTCCTCAGAACCAGTCTTCAACATCCTTAGTTACTTCTGAACCTGTCACCAGACCCAAGTCTCTGTCTGACCTTACAAGTCAGAAACACCGCCATACTAACCATGAACTCTATTCACTTGCTCAATCAAATCGCCAACAGGAAAAGGAACATGCTAGGAAATGGATTTTAAGGGTGTGGGATAATGGTGGGAGACTCACAATACTGGATCAGATTGAATTTCTCAGTTTAGGTCCTTTGAGCCGAGATAGTGAGTTTAATGTCATAGCCCGCACTGTTGCAGATAATGGTGTGAAGAGTTTGTTTGATTGGTTGGCTGAAGCATGGGTCCAGAGATGGCCTACTACAAGAGAGCTACAGACGCCTGACGCCCTGGTGTGGTATTCTATTGAGGATGGGATTAAAAGGCTTAGGGAACTTGGAATGATAGAGTGGCTTTGTGTAAAAGCTACTTGTCCACAGTGGAGGGGCCCGGAAGATGCACCCATCACGAGAGCTATGAGGATAACTTTTGTCCAGGAAACTGTAGAGACTTGGAAGAGCTTCGTATTTAGCCTCCTCTGTATAAAGGACATAACAGTGGGGAGCGTGGCTGCTCAGTTGCATGATCTAACAGAATTAAGTTTAAAGCCAGCCGGGGGGCGTGGTGGCGCACGCTTTTAA

>Fv1CER15 MT077247 [organism=Mus cervicolor]

ATGAATTTCCCACGTGCGCTTGCTGGTTTCTCGAGCTGGCTCTTCAAACCTGAACTTGCCGAGGACTCTCCGGATAATGACGAGAACACTGTTAACCCATGGCGTGAGCTGCTGCAGAAGATAAATGTGGCCGATCTCCCCGATTCATCCTTTTCGAGCGGTAAGGAACTTAATGACTCTGTGTACGATACTTTTGAACATTTCTGCAAGACTAGGGACTATAACGCAGTTGGCGAGCTGCTTCTGGCATTTCTGGATAAAGTAACAAAGGAAAGGGACCAATTCAGAGATGAAATTTCCCAGCTCCGAATGCACATAAATGATCTAAAGGCTTCTAAGTGTGTCCTGGGGGAGACTCTTCTATCCTACCGCCACAGGATTGAAGTTGGGGAAAAACAGACTGAAGCCCTCATTGTGAGGTTAGCTGATGTGCAATCCCAGGTCATGTGTCAGCCTGCCCGGAAATTGTCTGCAGATAAGGTGAGGGCACTGATTGGTAAAGAATGGGATCCCGTTACCTGGGATGGAGATGTGTGGGAGGACATAGATTCTGAGGGAACTGAGGAAGCAGAGTTGCCCACTGTCTTGGCCTCTCCATCCTTGTCTGAGGAAAGTGGTTATGCCTTGTCTAAAGAACGCACCCAGCAGGACAAAACAGATGCTCCTCAGAACCAGTCTTCAACATCCTTAGTTACTTCTGAACCTGTCACCAGACCCAAGTCTCTGTCTGACCTTACAAGTCAGAAACACCGCCATACTAACCATGAACTCTATTCACTTGCTCAATCAAATCGCCAACAGGAAAAGGAACATGCTAGGAAATGGATTTTAAGGGTGTGGGATAATGGTGGGAGGCTCACAATACTGGATCAGATTGAATTTCTCAGTTTAGGTCCTTTGAGCCGAGATAGTGAGTTTAATGTCATAGCCCGCACTGTTGCAGATAATGGTGTGAAGAGTTTGTTTGATTGGTTGGCTGAAGCATGGGTCCAGAGATGGCCTACTACAAGAGAGCTACAGACGCCTGACGCCCTGGAGTGGTATTCTATTGAGGATGGGATTAAAAGGCTTAGGGAACTTGGAATGATAGAGTGGCTTTGTGTAAAAGCTACTTGTCCACAGTGGAGGGGCCCGGAAGATGCACCCATCACGAGAGCTATGAGGATAACTTTTGTCCAGGAAACTGTAGAGACTTGGAAGAGCTTCGTATTTAGCCTCCTCTGTATAAAGGACATAACAGTGGAGAGCGTGGCTGCTCAGTTGCATGATCTAACAGAATTAAGTTTAAAGCCAGCCGGGGGGCGTGGTGGCGCACGCTTTTAA

>Fv1CER16 MT077248 MT077248 [organism=Mus cervicolor]

ATGAATTTCCCACGTGCGCTTGCTGGTTTCTCGAGCTGGCTCTTCAAACCTGAACTTGCCGAGGACTCTCCGGATAATGACGAGAACACTGTTAACCCATGGCGTGAGCTGCTGCAGAAGATAAATGTGGCCGATCTCCCCGATTCATCCTTTTCGAGCGGTAAGGAACTTAATGACTCTGTGTACGATACTTTTGAACATTTCTGCAAGACTAGGGACTATAACGCAGTTGGCGAGCTGCTTCTGGCATTTCTGGATAAAGTAACAAAGGAAAGGGACCAATTCAGAGATGAAATTTCCCAGCTCCGAATGAACATAAATGATCTAAAGGCTTCTAAGTGTGTCCTGGGGGAGACTCTTCTATCCTACCGCCACAGGATTGAAGTTGGGGAAAAACAGACTGAAGCCCTCATTGTGAGGTTAGCTGATGTGCAATCCCAGGTCATGTGTCAGCCTGCCCGGAAAGTGTCTGCAGATAAGGTGAGGGCACTGATTGGTAAAGAATGGGATCCCGTTACCTGGGATGGAGATGTGTGGGAGGACATAGATTCTGAGGGAACTGAGGAAGCAGAGTTGCCCACTGTCTTGGCCTCTCCATCCTTGTCTGAGGAAAGTGGTTATGCCTTGTCTAAAGAACGCACCCAGCAGGACAAAACAGATGCTCCTCAGAACCAGTCTTCAACATCCTTAGTTACTTCTGAACCTGTCACCAGACCCAAGTCTCTGTCTGACCTTACAAGTCAGAAACACCGCCATACTAACCATGAACTCTATTCACTTGCTCAATCAAATCGCCAACAGGAAAAGGAACATGCTAGGAAATGGATTTTAAGGGTGTGGGATAATGGTGGGAGGCTCACAATACTGGATCAGATTGAATTTCTCAGTTTAGGTCCTTTGAGCCGAGATAGTGAGTTTAATGTCATAGCCCGCACTGTTGCAGATAATGGTGTGAAGAGTTTGTTTGATTGGTTGGCTGAAGCATGGGTCCAGAGATGGCCTACTACAAGAGAGCTACAGACGCCTGACGCCCTGGAGTGGTATTCTATTGAGGATGGGATTAAAAGGCTTAGGGAACTTGGAATGATAGAGTGGCTTTGTGTAAAAGCTACTTGTCCACAGTGGAGGGGCCCGGAAGATGCACCCATCACGAGAGCTATGAGGATAACTTTTGTCCAGGAAACTGTAGAGACTTGGAAGAGCTTCGTATTTAGCCTCCTCTGTATAAAGGACATAACAGTGGGGAGCGTGGCTGCTCAGTTGCATGATCTAACAGAATTACGTTTAAAGCCAGCCGGGGGGCGTGGTGGCGCACGCTTTTAA

>Fv1CER17 MT077249 [organism=Mus cervicolor]

ATGAATTTCCCACGTGCGCTTGCTGGTTTCTCGAGCTGGCTCTTCAAACCTGAACTTGCCGAGGACTCTCCGGATAATGACGAGAACACTGTTAACCCATGGCGTGAGCTGCTGCAGAAGATAAATGTGGCCGATCTCCCCGATTCATCCTTTTCGAGCGGTAAGGAACTTAATGACTCTGTGTACGATACTTTTGAACATTTCTGCAAGATTAGGGACTATGACGCAGTTGGCGAGCTGCTTCTGGCATTTCTGGATAAAGTAACAAAGGAAAGGGACCAATTCAGAGATGAAATTTCCCAGCTCCGAATGCACATAAATGATCTAAAGGCTTCTAAGTGTGTCCTGGGGGAGACTCTTCTCTCCTACCGCCACAGGATTGAAGTTGGGGAAAAACAGACTGAAGCCCTCATTGTGAGGTTAGCTGATGTGCAATCCCAGGTCATGTGTCAGCCTGCCCGGAAAGTGTCTGCAGATAAGGTGAGGGCACTGATTGGTAAAGAATGGGATCCCGTTACCTGGGATGGAGATGTGTGGGAGGACATAGATTCTGAGGGAACTGAGGAAGCAGAGTTGCCCACTGTCTTGGCCTCTCCATCCTTGTCTGAGGAAAGTGGTTATGCCTTGTCTAAAGAACGCACCCAGCAGGACAAAACAGATGCTCCTCAGAACCAGTCTTCAACATCCTTAGTTACTTCTGAACCTGTCACCAGACCCAAGTCTCTGTCTGACCTTACAAGTCAGAAACACCGCCATACTAACCATGAACTCTATTCACTTGCTCAATCAAATCGCCAACAGGAAAAGGAACATGCTAGGAAATGGATTTTAAGGGTGTGGGATAATGGTGGGAGGCTCACAATACTGGATCAGATTGAATTTCTCAGTTTAGGTCCTTTGAGCCGAGATAGTGAGTTTAATGTCATAGCCCGCACTGTTGCAGATAATGGTGTGAAGAGTTTGTTTGATTGGTTGGCTGAAGCATGGGTCCAGAGATGGCCTACTACAAGAGAGCTACAGACGCCTGACGCCCTGGAGTGGTATTCTATTGAGGATGGGATTAAAAGGCTTAGGGAACTTGGAATGATAGAGTGGCTTTGTGTAAAAGCTACTTGTCCACAGTGGAGGGGCCCGGAAGATGCACCCATCACGAGAGCTATGAGGATAACTTTTGTCCAGGAAACTGTAGAGACTTGGAAGAGCTTCGTATTTAGCCTCCTCTGTATAAAGGACATAACAGTGGGGAGCGTGGCTGCTCAGTTGCATGATCTAACAGAATTAAGTTTAAAGCCAGCCGGGGGGCGTGGTGGCGCACGCTTTTAA

>Fv1CER18 MT077250 [organism=Mus cervicolor]

ATGAATTTCCCACGTGCGCTTGCTGGTTTCTCGAGCTGGCTCTTCAAACCTGAACTTGCCGAGGACTCTCCGGATAATGACGAGAACACTGTTAACCCATGGCGTGAGCTGCTGCAGAAGATAAATGTGGCCGATCTCCCCGATTCATCCTTTTCGAGCGGTAAGGAACTTAATGACTCTGTGTACGATACTTTTGAACATTTCTGCAAGACTAGGGACTATAACGCAGTTGGCGAGCTGCTTCTGGCATTTCTGGATAAAGTAACAAAGGAAAGGGACCAATTCAGAGATGAAATTTCCCAGCTCCGAATGCACATAAATGATCTAAAGGCTTCTAAGTGTGTCCTGGGGGAGACTCTTCTATCCTACCGCCACAGGATTGAAGTTGGGGAAAAACAGACTGAAGCCCTCATTGTGAGGTTAGCTGATGTGCAATCCCAGGTCATGTGTCAGCCTGCCCGGAAAGTGTCTGCAGATAAGGTGAGGGCACTGATTGGTAAAGAATGGGATCCCGTTACCTGGGATGGAGATGTGTGGGAGGACATAGATTCTGAGGGAACTGAGGAAGCAGAGTTGCCCACTGTCTTGGCCTCTCCATCCTTGTCTGAGGAAAGTGGTTATGCCTTGTCTAAAGAACGCACCCAGCAGGACAAAACAGATGCTCCTCAGAACCAGTCTTCAACATCCTTAGTTACTTCTGAACCTGTCACCAGACCCAAGTCTCTGTCTGACCTTACAAGTCAGAAACACCGCCATACTAACCATGAACTCTATTCACTTGCTCAATCAAATCGCCAACAGGAAAAGGAACATGCTAGGAAATGGATTTTAAGGGTGTGGGATAATGGTGGGAGGCTCACAATACTGGATCAGATTGAATTTCTCAGTTTAGGTCCTTTGAGCCGAGATAGTGAGTTTAATGTCATAGCCCGCACTGTTGCAGATAATGGTGTGAAGAGTTTGTTTGATTGGTTGGCTGAAGCATGGGTCCAGAGATGGCCTATTACAAGAGAGCTACAGACGCCTGACGCCCTGGAGTGGTATTCTATTGAGGATGGGATTAAAAGGCTTAGGGAACTTGGAATGATAGAGTGGCTTTGTGTAAAAGCTACTTGTCCACAGTGGAGGGGCCCGGAAGATGCACCCATCACGAGAGCTATGAGGATAACTTTTGTCCAGGAAACTGTAGAGACTTGGAAGAGCTTCGTATTTAGCCTCCTCTGTATAAAGGACATAACAGTGGGGAGCGTGGCTGCTCAGTTGCATGATCTAACAGAATTAAGTTTAAAGCCAGCCGGGGGGCGTGGTGGCGCACGCTTTTAA

>Fv1CER19 MT077251 [organism=Mus cervicolor]

ATGAATTTCCCACGTGCGCTTGCTGGTTTCTCGAGCTGGCTCTTCAAACCTGAACTTGCCGAGGACTCTCCGGATAATGACGAGAACACTGTTAACCCATGGCGTGAGCTGCTGCAGAAGATAAATGTGGCCGATCTCCCCGATTCATCCTTTTCGAGCGGTAAGGAACTTAATGACTCTGTGTACGATACTTTTGAACATTTCTGCAAGACTAGGGACTATAACGCAGTTGGCGAGCTGCTTCTGGCATTTCTGGATAAAGTAACAAAGGAAAGGGACCAATTCAGAGATGAAATTTCCCAGCTCCGAATGCACATAAATGATCTAAAGGCTTCTAAGTGTGTCCTGGGGGAGACTCTTCTATCCTACCGCCACAGGATTGAAGTTGGGGAAAAACAGACTGAAGCCCTCATTGTGAGGTTAGCTGATGTGCAATCCCAGGTCATGTGTCAGCCTGCCCGGAAAGTGTCTGCAGATAAGGTGAGGGCACTGATTGGTAAAGAATGGGATCCCGTTACCTGGGATGGAGATGTGTGGGAGGACATAGATTCTGAGGGAACTGAGGAAGCAGAGTTGCCCACTGTCTTGGCCTCTCCATCCTTGTCTGAGGAAAGTGGTTATGCCTTGTCTAAAGAACGCACCCAGCAGGACAAAACAGATGCTCCTCAGAACCAGTCTTCAACATCYTTAGTTACTTCTGAACCTGTCACCAGACCCAAGTCTCTGTCTGACCTTACAAGTCAGAAACACCGCCATACTAACCATGAACTCTATTCACTTGCTCAATCAAATCGCCAACAGGAAAAGGAACATGCTAGGAAATGGATTTTAAGGGTGTGGGATAATGGTGGGAGGCTCACAATACTGGATCAGATTGAATTTCTCAGTTTAGGTCCTTTGAGCCGAGATAGTGAGTTTAATGTCATAGCCCGCACTGTTGCAGATAATGGTGTGAAGAGTTTGTTTGATTGGTTGGCTGAAACATGGGTCCAGAGATGGCCTACTACAAGAGAGCTACAGACGCCTGACGCCCTGGAGTGGTATTCTATTGAGGATGGGATTAAAAGGCTTAGGGAACTTGGAATGATAGAGTGGCTTTGTGTAAAAGCTACTTGTCCACAGTGGAGGGGCCCGGAAGATGCACCCATCACGAGAGCTATGAGGATAACTTTTGTCCAGGAAACTGTAGAGACTTGGAAGAGCTTCGTATTTAGCCTCCTCTGTATAAAGGACATAACAGTGGGGAGCGTGGCTGCTCAGTTGCATGATCTAACAGAATTAAGTTTAAAGCCAGCCGGGGGGCGTGGTGGCGCACGCTTTTAA

>Fv1CER20 MT077252 [organism=Mus cervicolor]

ATGAATTTCCCACGTGCGCTTGCTGGTTTCTCGAGCTGGCTCTTCAAACCTGAACTTGCCGAGGACTCTCCGGATAATGACGAGAACACTGTTAACCCATGGCGTGAGCTGCTGCAGAAGATAAATGTGGCCGATCTCCCCGATTCATCCTTTTCGAGCGGTAAGGAACTTAATGACTCTGTGTACGATACTTTTGAACATTTCTGCAAGACTAGGGACTATAACGCAGTTGGCGAGCTGCTTCTGGCATTTCTGGATAAAGTAACAAAGGAAAGGGACCAATTCAGAGATGAAATTTCCCAGCTCCGAATGCACATAAATGATCTAAAGGCTTCTAAGTGTGTCCTGGGGGAGACTCTTCTATCCTACCGCCACAGGATTGAAGTTGGGGAAAAACAGACTGAAGCCCTCATTGTGAGGTTAGCTGATGTGCAATCCCAGGTCATGTGTCAGCCTGCCCGGAAAGTGTCTGCAGATAAGGTGAGGGCACTGATTGGTAAAGAATGGGATCCCGTTACCTGGGATGGAGATGTGTGGGAGGACATAGATTCTGAGGGAACTGAGGAAGCAGAGTTGCCCACTGTCTTGGCCTCTCCATCCTTGTCTGAGGAAAGTGGTTATGCCTTGTCTAAAGAACGCACCCAGCAGGACAAAACAGATGCTCCTCAGAACCAGTCTTCAACATCCTTAGTTACTTCTGAACCTGTCACCAGACCCAAGTCTCTGTCTGACCTTACAAGTCAGAAACACCGCCATACTAACCATGAACTCTATTCACTTGCTCAATCAAATCGCCAACAGGAAAAGGAACATGCTAGGAAAACAGATGCTCCTCAGAACCAGTCTTCAACATCCTTAGTTACTTCTGAACCTGTCACCAGACCCAAGTCTCTGTCTGACCTTACAAGTCAGAAACACCGCCATACTAACCATGAACTCTATTCACTTGCTCAATCAAATCGCCAACAGGAAAAGGAACATGCTAGGAAATGGATTTTAAGGGTGTGGGATAATGGTGGGAGGCTCACAATACTGGATCAGATTGAATTTCTCAGTTTAGGTCCTTTGAGCCGAGATAGTGAGTTTAATGTCATAGCCCGCACTGTTGCAGATAATGGTGTGAAGAGTTTGTTTGATTGGTTGGCTGAAACATGGGTCCAGAGATGGCCTACTACAAGAGAGCTACAGACGCCTGACGCCCTGGAGTGGTATTCTACTGAGGATGGGATTAAAAGGCTTAGGGAACTTGGAATGATAGAGTGGCTTTGTGTAAAAGCTACTTGTCCACAGTGGAGGGGCCCGGAAGATGCACCCATCACGAGAGCTATGAGGATAACTTTTGTCCAGGAAACTGTAGAGACTTGGAAGAGCTTCGTATTTAGCCTCCTCTGTATAAAGGACATAACAGTGGGGAGCGTGGCTGCTCAGTTGCATGATCTAACAGAATTAAGTTTAAAGCCAGCCGGGGGGCGTGGTGGCGCACGCTTTTAA

>Fv1CER21 MT077253 [organism=Mus cervicolor]

ATGAATTTCCCACGTGCGCTTGCTGGTTTCTCGAGCTGGCTCTTCAAACCTGAACTTGCCGAGGACTCTCCGGATAATGACGAGAACACTGTTAACCCATGGCGTGAGCTGCTGCAGAAGATAAATGTGGCCGATCTCCCCGATTCATCCTTTTCGAGCGGTAAGGAACTTAATGACTCTGTGTACGATACTTTTGAACATTTCTGCAAGACTAGGGACTATAACGCAGTTGGCGAGCTGCTTCTGGCATTTCTGGATAAAGTAACAAAGGAAAGGGACCAATTCAGAGATGAAATTTCCCAGCTCCGAATGCACATAAATGATCTAAAGGCTTCTAAGTGTGTCCTGGGGGAGACTCTTCTATCCTACCGCCACAGGATTGAAGTTGGGGAAAAACAGACTGAAGCCCTCATTGTGAGGTTAGCTGATGTGCAATCCCAGGTCATGTGTCAGCCTGCCCGGAAAGTGTCTGCAGATAAGGTGAGGGCACTGATTGGTAAAGAATGGGATCCCGTTACCTGGGATGGAGATGTGTGGGAGGACATAGATTCTGAGGGAACTGAGGAAGCAGAGTTGCCCACTGTCTTGGCCTCTCCATCCTTGTCTGAGGAAAGTGGTTATGCCTTGTCTAAAGAACGCACCCAGCAGGACAAAACAGATGCTCCTCAGAACCAGTCTTCAACATCCTTAGTTACTTCTGAACCTGTCACCAGACCCAAGTCTCTGTCTGACCTTACAAGTCAGAAACACCGCCATACTAACCATGAACTCTATTCACTTGCTCAATCAAATCGCCAACAGGAAAAGGAACATGCTAGGAAATGGATTTTAAGGGTGTGGGATAATGGTGGGAGGCTCACAATACTGGATCAGATTGAATTTCTCAGTTTAGGTCCTTTGAGCCGAGATAGTGAGTTTAATGTCATAGCCCGCACTGTTGCAGATAATGGTGTGAAGAGTTTGTTTGATTGGTTGGCTGAAGCATGGGTCCAGAGATGGCCTACTACAAGAGAGCTACAGACGCCTGACGCCCTGGTGTGGTATTCTATTGAGGATGGGATTAAAAGGCTTAGGGAACTTGGAATGATAGAGTGGCTTTGTGTAAAAGCTACTTGTCCACAGTGGAGGGGCCCGGAAGATGCACCCATCACGAGAGCTATGAGGATAACTTTTGTCCGGGAAACTGTAGAGACTTGGAAGAGCTTCGTATTTAGCCTCCTCTGTATAAAGGACATAACAGTGGGGAGCGTGGCTGCTCAGTTGCATGATCTAACAGAATTAAGTTTAAAGCCAGCCGGGGGCGTGGTGGCGCACGCTTTTAATCCTAGCACTCGGGAGGCAGAGGCAGGCGGATTTCTGAGTTCAAGGCCAGCCTGGTCTACAAAGTGA

>Fv1CER22 MT077254 [organism=Mus cervicolor]

ATGAATTTCCCACGTGCGCTTGCTGGTTTCTCGAGCTGGCTCTTCAAACCTGAACTTGCCGAGGACTCTCCGGATAATGACGAGAACACTGTTAACCCATGGCGTGAGCTGCTGCAGAAGATAAATGTGGCCGATCTCCCCGATTCATCCTTTTCGAGCGGTAAGGAACTTAATGACTCTGTGTACGATACTTTTGAACATTTCTGCAAGACTAGGGACTATAACGCAGTTGGCGAGCTGCTTCTGGCATTTCTGGATAAAGTAACAAAGGAAAGGGACCAATTCAGAGATGAAATTTCCCAGCTCCGAATGCACATAAATGATCTAAAGGCTTCTAAGTGTGTCCTGGGGGAGACTCTTCTATCCTACCGCCACAGGATTGAAGTTGGGGAAAAACAGACTGAAGCCCTCATTGTGAGGTTAGCTGATGTGCAATCCCAGGTCATGTGTCAGCCTGCCCGGAAAGTGTCTGCAGATAAGGTGAGGGCACTGATTGGTAAAGAATGGGATCCCGTTACCTGGGATGGAGATGTGTGGGAGGACATAGATTCTGAGGGAACTGAGGAAGCAGAGTTGCCCACTGTCTTGGCCTCTCCATCCTTGTCTGAGGAAAGTGGTTATGCCTTGTCTAAAGAACGCACCCAGCAGGACAAAACAGATGCTCCTCAGAACCAGTCTTCAACATCCTTAGTTACTTCTGAACCTGTCACCAGACCCAAGTCTCTGTCTGACCTTACAAGTCAGAAACACCGCCATACTAACCATGAACTCTATTCACTTGCTCAATCAAATCGCCAACAGGAAAAGGAACATGCTAGGAAATGGATTTTAAGGGTGTGGGATAATGGTGGGAGGCTCACAATACTGGATCAGATTGAATTTCTCAGTTTAGGTCCTTTGAGCCGAGATAGTGAGTTTAATGTCATAGCCCGCACTGTTGCAGATAATGGTGTGAAGAGTTTGTTTGATTGGTTGGCTGAAGCATGGGTCCAGAGATGGCCTACTACAAGAGAGCTACAGATGCCTGACGCCCTGGAGTGGTATTCTATTGAGGATGGGATTAAAAGGCTTAGGGAACTTGGAATGATAGAGTGGCTTTGTGTAAAAGCTACTTGTCCACAGTGGAGGGGCCCGGAAGATGCACCCATCACGAGAGCTATGAGGATAACTTTTGTCCAGGAAACTGTAGAGACTTGGAAGAGCTTCGTATTTAGCCTCCTCTGTATAAAGGACATAACAGTGGGAAGCGTGGCTGCTCAGTTGCATGATCTAACAGAATTAAGTTTAAAGCCAGCCGGGGGGCGTGGTGGCGCACGCTTTTAA

>Fv1CER23 MT077255 [organism=Mus cervicolor]

ATGAATTTCCCACGTGCGCTTGCTGGTTTCTCGAGCTGGCTCTTCAAACCTGAACTTGCCGAGGACTCTCCGGATAATGACGAGAACACTGTTAACCCATGGCGTGAGCTGCTGCAGAAGATAAATGTGGCCGATCTCCCCGATTCATCCTTTTCGAGCGGTAAGGAACTTAATGACTCTGTGTACGATACTTTTGAACATTTCTGCAAGACTAGGGACTATAACGCAGTTGGCGAGCTGCTTCTGGCATTTCTGGATAAAGTAACAAAGGAAAGGGACCAATTCAGAGATGAAATTTCCCAGCTCCGAATGCACATAAATGATCTAAAGGCTTCTAAGTGTGTCCTGGGGGAGACTCTTCTATCCTACCGCCACAGGATTGAAGTTGGGGAAAAACAGACTGAAGCCCTCATTGTGAGGTTAGCTGATGTGCAATCCCAGGTCATGTGTCAGCCTGCCCGGAAAGTGTCTGCAGATAAGGTGAGGGCACTGATTGGTAAAGAATGGGATCCCGTTACCTGGGATGGAGATGTGTGGGAGGACATAGATTCTGAGGGAACTGAGGAAGCAGAGTTGCCCACTGTCTTGGCCTCTCCATCCTTGTCTGAGGAAAGTGGTTATGCCTTGTCTAAAGAACGCACCCAGCAGGACAAAACAGATGCTCCTCAGAACCAGTCTTCAACATCCTTAGTTACTTCTGAACCTGTCACCAGACCCAAGTCTCTGTCTGACCTTACAAGTCAGAAACACCGCCATACTAACCATGAACTCTATTCACTTGCTCAATCAAATCGCCAACAGGAAAAGGAACATGCTAGGAAATGGATTTTAAGGGTGTGGGATAATGGTGGGAGGCTCACAATACTGGATCAGATTGAATTTCTCAGTTTAGGTCCTTTGAGCCGAGATAGTGAGTTTAATGTCATAGCCCGCACTGTTGCAGATAATGGTGTGAAGAGTTTGTTTGATTGGTTGGCTGAAGCATGGGTCCAGAGATGGCCTACTACAAGAGAGCTACAGACGCCTGACGCCCTGGAGTGGTATTCTATTGAGGATGGGATTAAAAGGCTTAGGGAACTTGGAATGATAGAGTGGCTTTGTGTAAAAGCTACTTGTCCACAGTGGAGGGGCCCGGAAGATGCACCCATCACGAGAGCTATGAGGATAACTTTTGTCCAGGAAACTGTAGAGACTTGGAAGAGCTTCGTATTTAGCCTCCTCTGTATAAAGGACATAACAGTGGGGAACGTGGCTGCTCAGTTGCATGATCTAACAGAATTAAGTTTAAAGCCAGCCGGGGGGCGTGGTGGCGCACGCTTTTAA

>Fv7CER1 MT077256 [organism=Mus cervicolor]

ATGAATTTCCCACGTGGGCTTGCTGGTTTCTCGAGCTGGCTCTTCAAACCTGAACTTGCCGAGGACTCTCCAGATAAAGACGAGAACCCTGTTAACCCATGGCGTGAGCTGCTGCAGAAGATAAATGTGGCCGATCTCCCCGATTCATCCTTTTCGAGCGGTAAGGAACTTAATGACTCTGTGTACGATACTTTTGAACATTTCTGCAAGATTAGGGACTATGACGCAGTTGGCGAGCTGCTTCTGGCATTTCTGGATAAAGTAACAAAGGAAAGGGACCAATTCAGAGATGAAATTTCCCAGCTCCGAATGCTCATAAATGATCTAAAGGCTTCTAAGTGTGTCCTGGGGGAGACTCTTCTCTCCTACGGCCACAGGATTGAAGTTGGGGAAAAACAGACTGAAGCCCTCATTGTGAGGTTAGCTGATTTGCAATCCCAGGTCATGTGTCAGCCTGTCCGGAAAGTGTCTGCAGATAAGGTGAGGGCACTGATTGGTAAAGAATGGGATCCGGTAACCTGGGATGGAGATGTGTGGGAGGACATAGATTCTGATGGGACTGAGGAAGCAGAGTTGCCCACTGTCTTGGCCTCTCCATCCTTGTCTGAGGAAAGTGGTTATGCCTTGTCTAAAGAACGCACCCAGCAGGACAAAGCAGATGTTTCTCAGAACCAGTCTTCAACATCCTTAGTTACTTCTGAACCTGTCACCAGACCCAAGTCTCTGTCTGACCTTACAAGTCAGAAACACCGCCATACTAACCATGAACTCTATTCACTTGCTCAATCAAATCGCCAACAGGCAAAGGAACATGCTAGGAAATGGATTTTAAGGGTGTGGGATAATGGTGGGAGGCTCACAATACTGGATCAGACTGAATTTCTCAGTTTAGGTCCTTTGAGCCTCGATAGTGAGTTTAATGTCATAGCCCGCACTGTTGCAGATAATGGTGTGAAGAGTTTGTTTGATTGGTTGGCTGAAGCATGGGTCCAGAGATGGCCTACTACAAGAGAGCTGCAGACGCCTGACGCCCTGGAGTGGTATTCTATTGAGGATGGGATTAAAAGGCTTAGGGAACTTGGAATGATAGAGTGGCTTTGTGTAAAAGCTACTTGTCCACAGTGGAGGGGCCCGGAAGATGCACCCATCACGAGAGCTATGAGGATAACTTTTGTCCGGGAAACTGTAGAGACTTGGAAGAGCTTCGTTTTTAGCCTCCTCTGTATAAAGGACATAACAGTGGGGAGCGTGGCTGCTCAGTTGCATGATCTAATAGAATTAAGTTTAAAGCCAACAGCAGCTGGCTTGACTGCTGGTTTGGCTCCTGTGGGCTCTGTGTCGGTTCTCTCTCTCTCTCCCTGGAAACATCAAAGCAACAGTTAA

>Fv7CER2 MT077257 [organism=Mus cervicolor]

ATGAATTTCCCACGTGGGCTTGCTGGTTTCTCGAGCTGGCTCTTCAAACCTGAACTTGCCGAGGACTCTCCAGATAATGACAAGAACCCTGTTAACCCATGGCGTGAGCTGCTGCAGAAGATAAATGTGGCCGATCTCCCCGATTCATCCTTTTCGAGCGGTAAGGAACTTAATGACTCTGTGTACGATACTTTTGAACATTTCTGCAAGATTAGGGACTATGACGCAGTTGGCGAGCTGCTTCTGGCATTTCTGGATAAAGTAACAAAGGAAAGGGACCAATTCAGAGATGAAATTTCCCAGCTCCGAATGCTCATAAATGATCTAAAGGCTTCTAAGTGTGTCCTGGGGGAGACTCTTCTCTCCTACCGCCACAGGATTGAAGCTGGGGAAAAACAAACTGAAGCCCTCATTGTGAGGTTAGCTGATGTGCAATCCCAGGTCATGTGTCAGCCTGCCCGGAAAGTGTCTGCAGATAAGGTGAGGGCACTGATTGGTAAAGGATGGGATCCGGTAACCTGGGATGGAGATGTGTGGGAGGACATAGATTCTGAGGGGACTGAGGAAGCAGAGTTGCCCACTGTCTTGGCCTCTCCATCCTTGTCTGAGGAAAGTGGTTATGCCTTGTCTAAAGAACGCACCCAGCAGGACAAAGCAGATGTTTCTCAGAACCAGTCTTCAACATCCTTAGTTACTTCTGAACCTGTCACCAGACCCAAGTCTCTGTCTGACCTTACAAGTCAGAAACACCGCCATACTAACCATGAACTCTATTCACTTGCTCAATCAAATCGCCAACAGGCAAAGGAACATGCTAGGAAATGGATTTTAAAGGGTGTGGGATAATGGTGGGAGGCTCACAATACTGGATCAGACTGAATTTCTCAGTTTAGGTCCTTTGAGCCTCGATAGTGAGTTTAATGTCATAGCCCGCACTGTTGCAGATAATGGTGTGAAGAGTTTGTTTGATTGGTTGGGTGAAGCATGGGTCCAGAGATGGCCTACTACAAGAGAGCTGCAGACGCCTGACGCCCTGGAGTGGTATTCTATTGAGGATGGGATTAAAAGGCTTAGGGAACTTGGAATGATAGAGTGGCTTTGTGTAAAAGCTACTTGTCCACAGTGGAGGGGCCCGGAAGATGCACCCATCACGAGAGCTATGAGGATAACTTTTGTCCGGGAAACTGTAGAGACTTGGAAGAGCTTCGTTTTTAGCCTCCTCTGTATAAAGGACATAACAGTGGGGAGCGTGGCTGCTCAGTTGCATGATCTAATAGAATTAAGTTTAAAGCCAACAGCAGCTGGCTTGACTGCTGGTTTGGCTCCTGTGGGCTCTGTGTCGGTTCTCTCTCTCTCTCTCCCTGGAAACATCAAAGCAACAGTTAA

>Fv7CER3 MT077258 [organism=Mus cervicolor]

ATGAATTTCCCACGTGCGCTTGCTGGTTTCTCGAGCTGGCTCTTCAAACCTGAACTTGCCGAGGACTCTCCAGATAATGACGAGAACCCTGTTAACCCATGGCGTGAGCTGCTGCAGAAGATAAATGTGGCCGATCTCCCCGATTCATCCTTTTCGAGCGGTAAGGAACTTAATGACTCTGTGTACGATACTTTTGAACATTTCTGCAAGATTAGGGACTATGACGCAGTTGGCGAGATGCTTCTGGCATTTCTGGATAAAGTAACAAAGGAAAGGGACCAATTCAGAGATGAAATTTCCCAGCTCCGAATGCTCATAAATGATCTAAAGGCTTCTAAGTGTGTCCTGGGGGAGACTCTTCTCTCCTACCGCCACAGGATTGAAGTTGGGGAAAAACAAACTGAAGCCCTCATTGTGAGGTTAGCTGATTTGCAATCCCAGGTCATGTGTCAGCCTGCCCGGAAAGTGTCTGCAGATAAGGTGAGGGCACTGATTGGTAAAGAATGGGATCCCGTTACCTGGGATGGAGATGTGTGGGAGGACATAGATTCTGATGGGACTGAGGAAGCAGAGTTGCCCACTGTCTTGGCCTCTCCATCCTTGTCTGAGGAAAGTGGTTATGCCTTGTCTAAAGAACGCACCCAGCAGGACAAAGCAGTTGCTCCTCAGAACCAGTCTTCAACATCCTTAGTTACTTCTGAACCTGTCACCAGACCCAAGTCTCTGTCTGACCTTACAAGTCAGAAACACCGCCATACTAACCATGAACTCTATTCACTTGCTCAATCAAATCGCCAACAGGCAAAGGAACATGCTAGGAAATGGATTTTAAGGGTGTGGGATAATGGTGGGAGGCTCACAATACTGGATCAGACTGAATTTCTCAGTTTAGGTCCTTTGAGCCTCGATAGTGAGTTTAATGTCATAGCCCGCACTGTTGCAGATAATGGTGTGAAGAGTTTGTTTGATTGGTTGGCTGAAGCATGGGTCCAGAGATGGCCTACTACAAGAGAGCTGCAGACGCCTGACGCCCTGGAGTGGTATTCTATTGAGGATGGGATTAAAAGGCTTAGGGAACTTGGAATGATAGAGTGGCTTTGTGTAAAAGCTTCTTGTCCACAGTGGAGGGGCCCGGAAGATGCACCCATCACGAGAGCTATGAGGATAACTTTTGTCCAGGAAACTGTAGAGACTTGGAAGAGCTTCGTTTTTAGCCTCCTCTGTATAAAGGACATAACAGTGGGGAGCGTGGCTGCTCAGTTGCATGATCTAATAGAATTAAGTTTAAAGCCAACAGCAGCTGGCTTGACTGCTGGTTTGGCTCCTGTGGGCTCTGTGTCGGTTCTCTCTCTCTCTCCCTGGAAACATCAAAGCAACAGTTAA

>Fv7CER4 MT077259 [organism=Mus cervicolor]

ATGAATTTCCCACGTGCGCTTGCTGGTTTCTCGAACTGGCTCTTCAAACCTGAACTTGCCGAGGACTCTCCAGATAATGATGAGAACCCTGTTAACCCATGGCGTGAGCTGCTGCAGAAGATAAATGTGGCCGATCTCCCCGATTCATCCTTTTCGAGCGGTAAGGAACTTAATGACTCTGTGTACGATACTTTTGAACATTTCTGCAAGATTAGGGACTATGACGCAGTTGGCGAGCTGCTTCTGGCATTTCTGGATAAAGTAACAAAGGAAAGGGACCAATTCAGAGATGAAATTTCCCAGCTCCGAATGCTCATAAATGATCTAAAGGCTTCTAAGTGTGTCCTGGGGGAGACTCTTCTCTCCTACCGCCACAGGATTGAAGTTGGGGAAAAACAGACTGAAGCCCTCATTGTGAGGTTAGCTGATTTGCAATCCCAGGTCATGTGTCAGCCTGCCCGGAAAGTGTCTGCAGATAAGGTGAGGGCACTGATTGGTAAAGAATGGGATCCCGTTACCTGGGATGGAGATGTGTGGGAGGACATAGATTCTGAGGGGACTGAGGAAGCAGAGTTGCCCACTGTCTTGGCCTCTCCATCCTTGTCTGAGGAAAGTGGTTATGCCTTGTCTAAAGAACGCACCCAGCAGGACAAAGCAGATGTTTCTCAGAACCAGTCTTCAGCATCCTTAGTTACTTCTGAACCTGTCACCAGACCCAAGTCTCTGTCTGACCTTACAAGTCAGAAACACCGCCATACTAACCATGAACTCTATTCACTTGCTCAATCAAATCGCCAACGGGAAAAGGAACATGCTAGGAAATGGATTTTAAGGGTGTGGGATAATGGTGGGAGGCTCACAATACTGGATCAGACTGAATTTCTCAATTTAGGTCCTTTGAGCCTCGATAGTGAGTTTAATGTCATAGCCCGCACTGTTGCAGATAATGGTGTGAAGAGTTTGTTTGATTGGTTGGCTGAAGCATGGGTCCAGAGATGGCCTACTACAAGAGAGCTGCAGACGCCTGACGCCCTGGAGTGGTATTCTATTGAGGATGGGATTAAAAGGCTTAGGGAACTTGGAATGATAGAGTGGCTTTGTGTAAAAGCTACTTGTCCACAGTGGAGGGGCCCGGAAGATGCACCCATCACGAGAGCTATGAGGATAACTTTTGTCCGGGAAACTGTAGAGACTTGGAAGAGCTTCGTTTTTAGCCTCCTCTGTATAAAGGACATAACAGTGGGGAGCGTGGCTGCTCAGTTGCATGATCTAATAGAATTAAGTTTAAAGCCAACAGCAGCTGGCTTGACTGCTGGTTTGGCTCCTGTGGGCTCTGTGTCGGTTCTCTCTCTCTCTCCCTGGAAACATCAAAGCAACAGTTAA

>Fv7CER5 MT077260 [organism=Mus cervicolor]

ATGAATTTCCCACGTGCGCTTGCTGGTTTCTCGAGCTGGCTCTTCAAACCTGAACTTGCCGAGGACTCTCCAGATAATGACGAGAACCCTGTTAACCCATGGCGTGAGCTGCTGCAGAAGATAAATGTGGCCGATCTCCCCGATTCATCCTTTTCGAGCGGTATGGAACTTAATGACTCTGTGTACGATACTTTTGAACATTTCTGCAAGATTAGGGACTATGACGCAGTTGGCGAGCTGCTTCTGGCATTTCTGGATAAAGTAACAAAGGAAAGGGACCAATTCAGAGATGAAATTTCCCAGCTCCGAATGCTCATAAATGATCTAAAGGCTTCTAAGTGTGTCCTGGGGGAGACTCTTCTCTCCTACTGCCACAGGATTGAAGTTGGGGAAAAACAGACTGAAGCCCTCATTGTGAGGTTAGCTGATTTGCAATCCCAGGTCATGTGTCAGCCTGCCCGGAAAGTGTCTGCAGATAAGGTGAGGGCACTGATTGGTAAAGAATGGGATCCCGTTACCTGGGATGGAGATGTGTGGGAGGACATAGATTCTGATGGGACTGAGGAAGCAGAGTTGCCCACTGTCTTGGCCTCTCCATCCTTGTCTGAGGAAAGTGGTTATGCCTTGTCTAAAGAACGCACCCAGCAGGACAAAACAGATGCTCCTCAGAACCAGTCTTCAACATCCTTAGTTACTTCTGAACCTGTCACTAGACCCAAGTCTCTGTCTGACCTTACAAGTCAGAAACACCGCCATACTAACCATGAACTCTATTCACTTGCTCAATCAAATCGCCAACGGGCAAAGGAACATGCTAGGAAATGGATTTTAAGGGTGTGGGATAATGGTGGGAGGCTCACAATACTGGATCAGACTGAATTTCTCAGTTTAGGTCCTTTGAGCCTTGATAGTGAGTTTAATGTCATAGCCAGCACTGTTGCAGATAATGGTGTGAAGAGTTTGTTTGATTGGTTGGCTGAAGCATGGGTCCAGAGATGGCCTACTACAAGAGAGCTGCAGACGCCTGACGCCCTGGAGTGGTATTCTATTGAGGATGGGATTAAAAGGCTTAGGGAACTTGGAATGATAGAGTGGCTTTGTGTAAAAGCTACTTGTCCACAGTGGAGGGGCCCGGAAGATGCACCCATCACGAGAGCTATGAGGATAACTTTTGTCCGGGAAACTGTAGAGACTTGGAAGAGCTTCGTTTTTAGCCTCCTCTGTATAAAGGACATAACAGTGGGGAGCGTGGCTGCTCAGTTGCATGATCTAATAGAATTAAGTTTAAAGCCAACAGCAGCTGGCTTGACTGCTGGTTTGGCTCCTGTGGGCTCTGTGTCGGTTCTCTCTCTCTCTCCCTGGAAACATCAAAGCAACAGTTAA

>Fv7CER6 MT077261 [organism=Mus cervicolor]

ATGAATTTCCCACGTGCGCTTGCTGGTTTCTCGAACTGGCTCTTCAAACCTGAACTTGCCGAGGACTCTCCAGATAATGACGAGAACCCTGTTAACCCATGGCGTGAGCTGCTGCAGAAGATAAATGTGGCCGATCTCCCCGATTCATCCTTTTCGAGCGGTAAGGAACTTAATGACTCTGTGTACGATACTTTTGAACATTTCTGCAAGATTAGGGACTATGACGCAGTTGGCAAGCTGCTTCTGGCATTTCTGGATAAAGTAACAAAGGAAAGGGACCAATTCAGAGATGAAATTTCCCAGCTCCGAATGCTCATAAATGATCTAAAGGCTTCTAAGTGTGTCCTGGGGGAGACTCTTCTCTCCTACCGCCACAGGATTGAAGTTGGGGAAAAACAGACTGAAGCCCTCATTGTGAGGTTAGCTGATGTGCAATCCCAGGTCATGTGTCAGCCTGCCCGGAAAGTGTCTGCAGATAAGGTGAGGGCACTGATTGGTAAAGAATGGGATCCGGTAACCTGGGATGGAGATGTGTGGGAGGACATAGATTCTGAGGGGACTGAGGAAGCAGAGTTGCCCACTGTCTTGGCCTCTCCATCCTTGTCTGAGGAAAGTGGTTATGCCTTGTCTAAAGAACGCACCCAGCAGGACAAAACAGATGCTCCTCAGAACCAGTCTTCAACATCCTTAGTTACTTCTGAACCTGTCACCAGACCCAAGTCTCTGTCTGACCTTACAAGTCAGAAACACCGCCATACTAACCATGAACTCTATTCACTTGCTCAATCAAATCGCCAACGGGAAAAGGAACATGCTAGGAAATGGATTTTAAGGGTGTGGGATAATGGTGGGAGGCTCACAATACTGGATCAGACTGAATTTCTCAGTTTAGGTCCTTTGAGCCTCGATAGTGAGTTTAATGTCATAGCCCGCACTGTTGCAGATAATGGTGTGAAGAGTTTGTTTGATTGGTTGGCTGAAGCATGGGTCCAGAGATGGCCTACTACAAGAGAGCTGCAGACGCCTGACGCCCTGGAGTGGTATTCTATTGAGGATGGGATTAAAAGGCTTAGGGAACTTGGAATGATAGAGTGGCTTTGTGTAAAAGCTACTTGTCCACAGTGGAGGGGCCCGGAAGATGCACCCATCACGAGAGCTATGAGGATAACTTTTGTCCGGGAAACTGTAGAGACTTGGAAGAGCTTCGTTTTTAGCCTCCTCTGTATAAAGGACATAACAGTGGGGAGCGTGGCTGCTCAGTTGCATGATCTAATAGAATTAAGTTTAAAGCCAACAGCAGCTGGCTTGACTGCTGGTTTGGCTCCTGTGGGCTCTGTGTCGGTTCTCTCTCTCTCTCCCTGGAAACATCAAAGCAACAGTTAA

>Fv7CER7 MT077262 [organism=Mus cervicolor]

ATGAATTTCCCACGTGCGCTTGCTGGTTTCTCGAGCTGGCTCTTCAAACCTGAACTTGCCGAGGACTCTCCAGATAATGACGAGAACCCTGTTAACCCATGGCGTGAGCTGCTGCAGAAGATAAATGTGGCCGATCTCCCCGATTCATCCTTTTCGAGCGGTAAGGAACTTAATGACTCTGTGTACGATACTTTTGAACATTTCTGCAAGATTAGGGACTATGACGCAGTTGGCAAGCTGCTTCTGGCATTTCTGGATAAAGTAACAAAGGAAAGGGACCAATTCAGAGATGAAATTTCCCAGCTCCGAATGCTCATAAATGATCTAAAGGCTTCTAAGTGTGTCCTGGGGGAGACTCTTCTCTCCTACCGCCACAGGATTGAAGTTGGGGAAAAACAGACTGAAGCCCTCATTGTGAGGTTAGCTGATGTGCAATCCCAGGTCATGTGTCAGCCTGCCCGGAAAGTGTCTGCAGATAAGGTGAGGGCACTGATTGGTAAAGAATGGGATCCGGTAACCTGGGATGGAGATGTGTGGGAGGACATAGATTCTGAGGGGACTGAGGAAGCAGAGTTGCCCACTGTCTTGGCCTCTCCATCCTTGTCTGAGGAAAGTGGTTATGCCTTGTCTAAAGAACGCACCCAGCAGGACAAAACAGATGCTCCTCAGAACCAGTCTTCAACATCCTTAGTTACTTCTGAACCTGTCACCAGACCCAAGTCTCTGTCTGACCTTACAAGTCAGAAACACCGCCATACTAACCATGAACTCTATTCACTTGCTCAATCAAATCGCCAACGGGAAAAGGAACATGCTAGGAAATGGATTTTAAGGGTGTGGGATAATGGTGGGAGGCTCACAATACTGGATCAGACTGAATTTCTCAGTTTAGGTCCTTTGAGCCTCGATAGTGAGTTTAATGTCATAGCCCGCACTGTTGCAGATAATGGTGTGAAGAGTTTGTTTGATTGGTTGGCTGAAGCATGGGTCCAGAGATGGCCTACTACAAGAGAGCTGCAGACGCCTGACGCCCTGGAGTGGTATTCTATTGAGGATGGGATTAAAAGGCTTAGGGAACTTGGAATGATAGAGTGGCTTTGTGTAAAAGCTACTTGTCCACAGTGGAGGGGCCCGGAAGATGCACCCATCACGAGAGCTATGAGGATAACTTTTGTCCGGGAAACTGTAGAGACTTGGAAGAGCTTCGTTTTTAGCCTCCTCTGTATAAAGGACATAACAGTGGGGAGCGTGGCTGCTCAGTTGCATGATCTAATAGAATTAAGTTTAAAGCCAACAGCAGCTGGCTTGACTGCTGGTTTGGCTCCTGTGGGCTCTGTGTTGGTTCTCTCTCTCTCTCCCTGGAAACATCAAAGCAACAGTTAA

>Fv7CER8 MT077263 [organism=Mus cervicolor]

ATGAATTTCCCACGTGCGCTTGCTGGTTTCTCGAGCTGGCTCTTCAAACCTGAACTTGCCGAGGACTCTCCAGATAATGACGAGAACCCTGTTAACCCATGGCGTGAGCTGCTGCAGAAGATAAATGTGGCCGATCTCCCCGATTCATCCTTTTCGAGCGGTAAGGAACTTAATGACTCTGTGTACGATACTTTTGAACATTTCTGCAAGATTAGGGACTATGACGCAGTTGGCGAGCTGCTTCTGGCATTTCTGGATAAAGTAACAAAGGAAAGGGACCAATTCAGAGATGAAATTTCCCAGCTCCGAATGCTCATAAATGATCTAAAGGCTTCTAAGTGTGTCCTGGGGGAGACTCTTCTCTCCTACCGCCACAGGATTGAAGTTGGGGAAAAACAGACTGAAGCCCTCATTGTGAGGTTAGCTGATTTGCAATCCCAGGTCATGTGTCAGCCTGCCCGGAAAGTGTCTGCAACTAAGATGAGGGCACTGATTGGTAAAGAATGGGATCCGGTAACCTGGGATGGAGATGTGTGGGAGGACATAGATTCTGATGGGACTGAGGAAGCAGAGTTGCCCACTGTCTTGGCCTCTCCATCCTTGTCTGAGGAAAGTGGTTATGCCTTGTCTAAAGAACGCACCCAGCAGGACAAAGCAGATGTTTCTCAGAACCAGTCTTCAACATCYTTAGTTACTTCTGAACCTGTCACCAGACCCAAGTCTCTGTCTGACCTTACAAGTCAGAAACACCGCCATACTAACCATGAACTCTATTCACTTGCTCAATAACAGGCAAAGGAACATGCTAGGAAATGGATTTTAAGGGTGTGGGATAATGGTGGGAGGCTCACAATACTGGATCAGACTGAATTTCTCAGTTTAGGTCCTTTGAGCCTCGATAGTGAGTTTAATGTCATAGCCCGCACTGTTGCAGATAATGGTGTGAAGAGTTTGTTTGATTGGTTGGCTGAAGCATGGGTCCAGAGATGGCCTACTACAAGAGAGCTGCAGACGCCTGACGCCCTGGAGTGGTATTCTATTGAGGATGGGATTAAAAGGCTTAGGGAACTTGGAATGATAGAGTGGCTTTGTGTAAAAGCTTCTTGTCCACAGTGGAGGGGCCCGGAAGATGCACCCATCATGAGAGCTATGAGGATAACTTTTGTCCGGGAAACTGTAGAGACTTGGAAGAGCTTCGTTTTTAGCCTCCTCTGTATAAAGGACATAACAGTGGGGAGCGTGGCTGCTCAGTTGCATGATCTAGTAGAATTAAGTTTAAAGCCAACAGCAGCTGGCTTGACTGCTGGTTTGGCTCCTGTGGGCTCTGTGTCGGTTCTCTCTCTCTCTCCCTGGAAACATCAAAGCAACAGTTAA

>Fv7CER9 MT077264 [organism=Mus cervicolor]

ATGAATTTCCCACGTGCGCTTGCTGGTTTCTCGAGCTGGCTCTTCAAACCTGAACTTGCCGAGGACTCTCCAGATAATGACGAGAACCCTGTTAACCCATGGCGTGAGCTGCTGCAGAAGATAAATGTGGCCGATCTCCCCGATTCATCCTTTTCGAGCGGTAAGGAACTTAATGACTCTGTGTACGATACTTTTGAACATTTCTGCAAGATTAGGGACTATGACGCAGTTGGCGAGCTGCTTCTGGCATTTCTGGATAAAGTAACAAAGGAAAGGGACCAATTCAGAGATGAAAGTTCCCAGCTCCGAATGCTCATAAATGATCTAAAGGCTTCTAAGTGTGTCCTGGGGGAGACTCTTCTCTCCTACCGCCACAGGATTGAAGTTGGGGAAAAACAGACTGAAGCCCTCATTGTGAGGTTAGCTGATTTGCAATCCCAGGTCATGTGTCAGCCTGCCCGGAAAGTGTCTGCAGATAAGGTGAGGGCACTGATTGGTAAAGAATGGGATCCGGTAACCTGGGATGGAGATGTGTGGGAGGACATAGATTCTGAGGGGACTGAGGAAGCAGAGTTGCCCACTGTCTTGGCCTCTCCATCCTTGTCTGAGGAAAGTGGTTATGCCTTGTCTAAAGAACGCACCCAGCAGGACAAAGCAGATGTTTCTCAGAACCAGTCTTCAACATCCTTAGTTACTTCTGAACCTGTCACCAGACCCAAGTCTCTGTCTGACCTTACAAGTCAGAAACACCGCCATACTAACCATGAACTCTATTCACTTGCTCAATCAAATCGCCAACAGGCAAAGGAACATGCTAGGAAATGGATTTTAAGGGTGTGGGATAATGGTGGGAGGCTCACAATACTGGATCAGACTGAATTTCTCAGTTTAGGTCCTTTGAGCCTCGATAGTGAGTTTAATGTCATAGCCCGCACTGTTGCAGATAATGGTGTGAAGAGTTTGTTTGATTGGTTGGCTGAAGCATGGGTCCAGAGATGGCCTACTACAAGAGAGCTGCAGATGCCTGACGCCCTGGAGTGGTATTCTATTGAGGATGGGATTAAAAGGCTTAGGGAACTTGGAATGATAGAGTGGCTTTGTGTAAAAGCTACTTGTCCACAGTGGAGGGGCCCGGAAGATGCACCCATCACAAGAGCTATGAGGATAACTTTTGTCCGGGAAACTGTAGAGACTTGGAAGAGCTTCGTTTTTAGCCTCCTCTGTATAAAGGACATAACAGTGGGGAGCGTGGCTGCTCAGTTGCATGATCTAATAGAATTAAGTTTAAAGCCAACAGCAGCTGGCTTGACTGCTGGTTTGGCTCCTGTGGGCTCTGTGTCGGTTCTCTCTCTCTCTCCCTGGAAACATCAAAGCAACAGTTAA

>Fv7CER10 MT077265 [organism=Mus cervicolor]

ATGAATTTCCCACGTGCGCTTGCTGGTTTCTCGAGCTGGCTCTTCAAACCTGAACTTGCCGAGGACTCTCCAGATAAAGACGAGAACCCTGTTAACCCATGGCGTGAGCTGCTGCAGAAGATAAATGTGGCCGATCTCCCCGATTCATCCTTTTCGAGCGGTAAGGAACTTAATGACTCTGTGTACGATACTTTTGAACATTTCTGCAAGATTAGGGACTATGACGCAGTTGGCGAGCTGCTTCTGGCATTTCTGGATAAAGTAACAAAGGAAAGGGACCAATTCAGAGATGAAATTTCCCAGCTCCGAATGCTCATAAATGATCTAAAGGCTTCTAAGTGTGTCCTGGGGGAGACTCTTCTCTCCTACCGCCACAGGATTGAAGTTGGGGAAAAACAGACTGAAGCCCTCATTGTGAGGTTAGCTGATTTGCAATCCCAGGTCATGTGTCAGCCTGCCCGGAAAGTGTCTGCAGATAAGGTGAGGGCACTGATTGGTAAAGAATGGGATCCCGTTACCTGGGATGGAGATGTGTGGGAGGACATAGATTCTGAGGGAACTGAGGAAGCAGAGTTGCCCACTGTCTTGGCCTCTCCATCCTTGTCTGAGGAAAGTGGTTATGCCTTGTCTAAAGAACGCACCCAGCAGGACAAAGCAGATGTTTCTCAGAACCAGTCTTCAACATCCTTAGTTACTTCTGAACCTGTCACCAGACCCAAGTCTCTGTCTGACCTTACAAGTCAGAAACACCGCCATACTAACCATGAACTCTATTCACTTGCTCAATCAAATCGCCAACAGGCAAAGGAACATGCTAGGAAATGGATTTTAAGGGTGTGGGATAATGGTGGGAGGCTCACAATACTGGATCAGACTGAATTTCTCAGTTTAGGTCCTTTGAGCCTCGATAGTGAGTTTAATGTCATAGCCCGCACTGTTGCAGATAATGGTGTGAAGAGTTTGTTTGATTGGTTGGCTGAAGCATGGGTCCAGAGATGGCCTACTACAAGAGAGCTGCAGACGCCTGACGCCCTGGAGTGGTATTCTATTGAGGATGGGATTAAAAGGCTTAGGGAACTTGGAATGATAGAGTGGCTTTGTGTAAAAGCTTCTTGTCCACAGTGGAGGGGCCCGGAAGATGCACCCATCACGAGAGCTATGAGGATAACTTTTGTCCGGGAAACTGTAGAGACTTGGAAGAGCTTCGTTTTTAGCCTCCTCTGTATAAAGGACATAACAGTGGGGAGCGTGGCTGCTCAGTTGCATGATCTAATAGAATTAAGTTTAAAGCCAACAGCAGCTGGCTTGACTGCTGGTTTGGCTCCTGTGGACTCTGTGTCGGTTCTCTCTCTCTCTCCCTGGAAACATCAAAGCAACAGTTAA

>Fv7CER11 MT077266 [organism=Mus cervicolor]

ATGAATTTCCCACGTGCGCTTGCTGGTTTCTCGAGCTGGCTCTTCAAACCTGAACTTGCCGAGGACTCTCCAGATAATGACCAGAACCCTGTTAACCCATGGCGTGAGCTGCTGCAGAAGATAAATGTGGCCGATCTCCCCGATTCATCCTTTTCGAGCGGTAAGGAACTTAATGACTCTGTGTACGATACTTTTGAACATTTCTGCAAGATTAGGGACTATGACGCAGTTGGCGAGCTGCTTCTGGCATTTCTGGATAAAGTAACAAAGGAAAGGGACCAATTCAGAGATGAAATTTCCCAGCTCCGAATGCTCATAAATGATCTAAAGGCTTCTAAGTGTGTCCTGGGGGAGACTCTTCTCTCCTACCGCCACAGGATTGAAGTTGGGGAAAAACAATCTGAAGCCCTCATTGTGAGGTTAGCTGATGTGCAATCCCAGGTCATGTGTCAGCCTGCCCGGAAAGTGTCTGCAGATAAGGTGAGGGCACTGATTGGTAAAGAATGGGATCCCGTTACCTGGGATGGAGATGTGTGGGAGGACATAGATTCTGATGGGACTGAGGAAGCAGAGTTGCCCACTGTCTTGGCCTCTCCATCCTTGTCTGAGGAAAGTGGTTATGCCTTGTCTAAAGAACGCACCCAGCAGGACAAAGCAGATGTTTCTCAGAACCAGTCTTCAACATCCTTAGTTACTTCTGAACCTGTCACCAGACCCAAGTCTCTGTCTGACCTTACAAGTCAGAAACACCGCCATACTAACCATGAACTCTATTCACTTTATCAATCAAATCGCCAACAGGCAAAGGAACATGCTAGGAAATGGATTTTAAGGGTGTGGGATAATGGTGGGAGGCTCACAATACTGGATCAGACTGAATTTCTCAGTTTAGGTCCTTTGAGCCTCGATAGTGAATTTAATGTCATAGCCCGCACTGTTGCAGATAATGGTGTGAAGAGTTTGTTTGATTGGTTGGCTGAAGCATGGGTCCAGAGATGGCCTACTACAAGAGAGCTGCAGACGCCTGACGCCCTGGAGTGGTATTCTATTGAGGATGGGATTAAAAGGCTTAGGGAACTTGGAATGATAGAGTGGCTTTGTGTAAAAGCTCCTTGTCCACAGTGGAGGGGCCCGGAAGATGCACCCATCACGAGAGCTATGAGGATAACTTTTGTCCGGGAAACTGTAGAGACTTGGAAGAGCTTCGTTTTTAGCATCCTCTGTATAAAGGACATAACAGTGGGGAGCGTGGCTGCTCAGTTGCATGATCTAATAGAATTAAGTTTAAAGCCAACAGCAGCTGGCTTGACTGCTGGTTTGGCTCCTGTGGGCTCTGTGTCGGTTCTCTCTCTCTCTCCCTGGAAACATCAAAGCAACAGTTAA

>Fv7CER12 MT077267 [organism=Mus cervicolor]

ATGAATTTCCCACGTGCGCTTGCTGGTTTCTCGAGCTGGCTCTTCAAACCTGAACTTGCCGAGGACTCTCCAGATAATGACGAGAACCCTGTTAACCCATGGCGTGAGCTGCTGCAGAAGATAAATGTGGCCGATCTCCCCGATTCATCCTTTTCGAGCGGTAAGGAACTTAATGACTCTGTGTACGATACTTTTGAACATTTCTGCAAGATTAGGGACTATGACGCAGTTGGCAAGCTGCTTCTGGCATTTCTGGATAAAGTAACAAAGGAAAGGGACCAATTCAGAGATGAAATTTCCCAGCTCCGAATGCTCATAAATGATCTAAAGGCTTCTAAGTGTGTCCTGGGGGAGACTCTTCTCTCCTACCGCCACAGGATTGAAGTTGGGGAAAAACAGACTGAAGCCCTCATTGTGAGGTTAGCTGATTTGCAATCCCAGGTCATGTGTCAGCCTGCCCGGAAAGTGTCTGCAGATAAGGTGAGGGCACTGATTGGTAAAGAATGGGATCCCGTTACCTGGGATGGAGATGTGTGGGAGGACATAGATTCTGAGGGGACTGAGGAAGCAGAGTTGCCCACTGTCTTGGCCTCTCCATCCTTGTCTGAGGAAAGTGGTTATGCCTTGTCTAAAGAACGCACCCAGCAGGACAAAGCAGATGTTCCTCAGAACCAGTCTTCAACATCCTTAGTTACTTCTGAACCTGTCACCAGACCCAAGTCTCTGTCTGACCTTACAAGTCAGAAACACCGCCATACTAACCATGAACTCTATTCACTTGCTCAATCAAATCGCCAACGGGAAAAGGAACATGCTAGGAAATGGATTTTAAGGGTGTGGGATAATGGTGGGAGGCTCACAATACTGGATCAGACTGAATTTCTCAATTTAGGTCCTTTGAGCCTCGATAGTGAGTTTAATGTCATAGCCCGCACTGTTGCAGATAATGGTGTGAAGAGTTTGTTTGATTGGTTGGCTGAAGCATGGGTCCAGAGATGGCCTACTACAAGAGAGCTGCAGACGCCTGACGCCCTGGAGTGGTATTCTATTGAGGATGGGATTAAAAGGCTTAGGGAACTTGGAATGATAGAGTGGCTTTGTGTAAAAGCTACTTGTCCACAGTGGAGGGGCCCGGAAGATGCACCCATCACAAGAGCTATGAGGATAACTTTTGTCCGGGAAACTGTAGAGATTTGGAAGAGCTTCGTTTTTAGCCTCCTCTGTATAAAGGACATAACAGTGGGGAGCGTGGCTGCTCAGTTGCATGATCTAATAGAATTAAGTTTAAAGCCAACAGCAGCTGGCTTGACTGCTGGTTTGGCTCCTGTGGGCTCTGTGTCGGTTCTCTCTCTCTCTCCCTGGAAACATCAAAGCAACAGTTAA

>Fv7CER13 MT077268 [organism=Mus cervicolor]

ATGAATTTCCCACGTGCGCTTGCTGGTTTCTCGAGCTGGCTCTTCAAACCTGAACTTGCCGAGGACTCTCCAGATAATGACGAGAACCCTGTTAACCCATGGCGTGAGCTGCTGCAGAAGATAAATGTGGCCGATCTCCCCGATTCATCCTTTTCGAGCGGTAAGGAACTTAATGACTCTGTGTACGATACTTTTGAACATTTCTGCAAGATTAGGGACTATGACGCAGTTGGCGAGCTGCTTCTGGCATTTCTGGATAAAGTAACAAAGGAAAGGGACCAATTCAGAGATGAAATTTCCCAGCTCCGAATGCTCATAAATGATCTAAAGGCTTCTAAGTGTGTCCTGGGGGAGACTCTTCTCTCCTACCGCCACAGGATTGAAGTTGGGGAAAAACAGACTGAAGCCCTCATTGTGAGGTTAGCTGATTTGCAATCCCAGGTCATGTGTCAGCCTGCCCGGAAAGTGTCTGCAGATAAGGTGAGGGCACTGATTGGTAAAGAATGGGATCCCGTTACCTGGGATGGAGATGTGTGGGAGGACATAGATTCTGAGGGGACTGAGGAAGCAGAGTTGCCCACTGTCTTGGCCTCTCCATCCTTGTCTGAGGAAAGTGGTTATGCCTTGTCTAAAGAACGCACCCAGCAGGACAAAGCAGATGTTTCTCAGAACCAGTCTTCAACATCCTTAGTTACTTCTGAACCTGTCACCAGACCCAAGTCTCTGTCTGACCTTACAAGTCAGAAACACCGCCATACTAACCATGAACTCTATTCACTTGCTCAATCAAATCGCCAACGGGAAAAGGAACATGCTAGGAAATGGATTTTAAGGGTGTGGGATAATGGTGGGAGGCTCACAATACTGGATCAGACTGAATTTCTCAATTTAGGTCCTTTGAGCCTCGATAGTGAGTTTAATGTCATAGCCCGCACTGTTGCAGATAATGGTGTGAAGAGTTTGTTTGATTGGTTGGCTGAAGCATGGGTCCAGAGATGGCCTACTACAAGAGAGCTGCAGACGCCTGACGCCCTGGAGTGGTATTCTATTGAGGATGGGATTAAAAGGCTTAGGGAACTTGGAATGATAGAGTGGCTTTGTGTAAAAGCTACTTGTCCACAGTGGAGGGGCCCGGAAGATGCACCCATCACAAGAGCTATGAGGATAACTTTTGTCCGGGAAACTGTAGAGATTTGGAAGAGCTTCGTTTTTAGCCTCCTCTGTATAAAGGACATAACAGTGGGGAGCGTGGCTGCTCAGTTGCATGATCTAATAGAATTAAGTTTAAAGCCAACAGCAGCTGGCTTGACTGCTGGTTTGGCTCCTGTGGGCTCTGTGTCGGTTCTCTCTCTCTCTCCCTGGAAACATCAAAGCAACAGTTAA

>Fv7CER14 MT077269 [organism=Mus cervicolor]

ATGAATTTCCCACGTGCGCTTGCTGGTTTCTCGAGCTGGCTCTTCAAACCTGAACTTGCCGAGGACTCTCCAGATAAAGACGAGAACCCTGTTAACCCATGGCGTGAGCTGCTGCAGAAGATAAATGTGGCCGATCTCCCCGATTCATCCTTTTCGAGCGGTAAGGAACTTAATGACTCTGTGTACGATACTTTTGAACATTTCTGCAAGATTAGGGACTATGACGCAGTTGGCGAGCTGCTTCTGGCATTTCTGGATAAAGTAACAAAGGAAAGGGACCAATTCAGAGATGAAATTTCCCAGCTCCGAATGCTCATAAATGATCTAAAGGCTTCTAAGTGTGTCCTGGGGGAGACTCTTCTCTCCTACCGCCACAGGATTGAAGTTGGGGAAAAACAGACTGAAGCCCTCATTGTGAGGTTAGCTGATTTGCAATCCCAGGTCATGTGTCAGCCTGCCCGGAAAGTGTCTGCAGATAAGGTGAGGGCACTGATTGGTAAAGAATGGGATCCCGTTACCTGGGATGGAGATGTGTGGGAGGACATAGATTCTGAGGGAACTGAGGAAGCAGAGTTGCCCACTGTCTTGGCCTCTCCATCCTTGTCTGAGGAAAGTGGTTATGCCTTGTCTAAAGAACGCACCCAGCAGGACAAAGCAGATGTTTCTCAGAACCAGTCTTCAACATCCTTAGTTACTTCTGAACCTGTCACCAGACCCAAGTCTCTGTCTGACCTTACAAGTCAGAAACACCGCCATACTAACCATGAACTCTATTCACTTGCTCAATCAAATCGCCAACAGGCAAAGGAACATGCTAGGAAATGGATTTTAAGGGTGTGGGATAATGGTGGGAGGCTCACAATACTGGATCAGACTGAATTTCTCAGTTTAGGTCCTTTGAGCCTCGATAGTGAGTTTAATGTCATAGCCCGCACTGTTGCAGATAATGGTGTGAAGAGTTTGTTTGATTGGTTGGCTGAAGCATGGGTCCAGAGATGGCCTACTACAAGAGAGCTGCAGACGCCTGACGCCCTGGAGTGGTATTCTATTGAGGATGGGATTAAAAGGCTTAGGGAACTTGGAATGATAGAGTGGCTTTGTGTAAAAGCTACTTGTCCACAGTGGAGGGGCCCGGAAGATGCACCCATCACGAGAGCTATGAGGATAACTTTTGTCCGGGAAACTGTAGAGACTTGGAAGAGCTTCGTTTTTAGCCTCCTCTGTATAAAGGACATAACAGTGGGGAGCGTGGCTGCTCAGTTGCATGATCTAATAGAATTAAGTTTAAAGCCAACAGCAGCTGGCTTGACTGCTGGTTTGGCTCCTGTGGGCTCTGTGTCAGTTCTCTCTCTCTCTCCCTGGAAACATCAAAGCAACAGTTAA

>Fv7CER15 MT077270 [organism=Mus cervicolor]

ATGAATTTCCCACGTGCGCTTGCTGGTTTCTCGAGCTGGCTCTTCAAACCTGAACTTGCCGAGGACTCTCCAGATAATGACGAGAACCCTGTTAACCCATGGCGTGAGCTGCTGCAGAAGATAAATGTGGCCGATCTCCCCGATTCATCCTTTTCGAGCGGTAAGGAACTTAATGACTCTGTGTACGATACTTTTGAACATTTCTGCAAGATTAGGGACTATGACGCAGTTGGCGAGCTGCTTCTGGCATTTCTGGATAAAGTAACAAAGGAAAGGGACCAATTCAGAGATGAAAGTTACCAGCTCCGAATGCTCATAAATGATCTAAAGGCTTCTAAGTGTGTCCTGGGGGAGACTCTTCTCTCCTACCGCCACAGGATTGAAGTTGGGGAAAAACAGACTGAAGCCCTCATTGTAAGGTTAGCTGATTTGCAATCCCAGGTCATGTGTCAGCCTGCCCGGAAAGTGTCTGCAGATAAGGTGAGGGCACTGATTGGTAAAGAATGGGATCCGGTAACCTGGGATGGAGATGTGTGGGAGGACATAGATTYTGATGGGACTGAGGAAGCAGAGTTGCCCACTGTCTTGGCCTCTCCATCCTTGTCTGAGGAAAGTGGTTATGCCTTGTCTAAAGAACGCACCCAGCAGGACAAAGCAGATGTTTCTCAGAACCAGTCTTCAACATCCTTAGTTACTTCTGAACCTGTCACCAGACCCAAGTCTCTGTCTGACCTTACAAGTCAGAAACACCGCTATACTAACCATGAACTCTATTCACTTGCTCAATCAAATCGCCAACAGGCAAAGGAACATGCTAGGAAATGGATTTTAAGGGTGTGGGATAATGGTGGGAGGCTTACAATACTGGATCAGACTGAATTTCTCAGTTTAGGTCCTTTGAGCCTCGATAGTGAGTTTAATGTCATAGCCCGCACTGTTGCAGATAATGGTGTGAAGAGTTTGTTTGATTGGTTGGCTGAAGCATGGGTCCAGAGATGGCCTACTACAAGAGAGCTGCAGACGCCTGATGCCCTGGAGTGGTATTCTATTGAGGATGGGATTAAAAGACTTAGGGAACTTGGAATGATAGAGTGGCTTTGTGTAAAAGCTACTTGTCCACAGTGGAGGGGCCCGGAAGATGCACCCATCACGAGAGCTATGAGGATAACTTTTGTCCGGGAAACTGTAGAGACTTGGAAGAGCTTCGTTTTTAGCCTCCTCTGTATAAAGGACATAACAGTGGGGAGCGTGGCTGCTCAGTTGCATGATCTAATAGAATTAAGTTTAAAGCCAACAGCAGCTGGCTTGACTGCTGGTTTGGCTCCTGTGGGCTCTGTGTCGGTTCTCTCTCTCTCTCCCTGGAAACATCAAAGCAACAGTTAA

>Fv7CER16 MT077271 [organism=Mus cervicolor]

ATGAATTTCCCACGTGCGCTTGCTGGTTTCTCGAGCTGGCTCTTCAAACCTGAACTTGCCGAGGACTCTCCAGATAAAGACGAGAACCCTGTTAACCCATGGCGTGAGCTGCTGCAGAAGATAAATGTGGCCGATCTCCCCGATTCATCCTTTTCGAGCGGTAAGGAACTTAATGACTCTGTGTACGATACTTTTGAACATTTCTGCAAGATTAGGGACTATGACGCAGTTGGCGAGCTGCTTCTGGCATTTCTGGATAAAGTAACAAAGGAAAGGGACCAATTCAGAGATGAAATTTCCCAGCTCCGAATGCTCATAAATGATCTAAAGGCTTCTAAGTGTGTCCTGGGGGAGACTCTTCTCTCCTACCGCCACAGGATTGAAGTTGGGGAAAAACAAACTGAAGCCCTCATTGTGAGGTTAGCTGATTTGCAATCCCAGGTCATGTGTCAGCCTGCCCGGAAAGTGTCTGCAACTAAGATGAGGGCACTGATTGGTAAAGAATGGGATCCGGTAACCTGGGATGGAGATGTGTGGGAGGACATAGATTCTGATGGGACTGAGGAAGCAGAGTTGCCCACTGTCTTGGCCTCTCCATCCTTGTCTGAGGAAAGTGGTTATGCCTTGTCTAAAGAACGCACCCAGCAGGACAAAACAGATGCTCCTCAGAACCAGTCTTCAACATCCTTAGTTACTTCTGAACCTGTCACCAGACCCAAGTCTCTCTCTGACCTTACAAGTCAGAAACACCGCCATACTAACCATGAACTCTATTCACTTGCTCAATCAAATCGCCAACAGGCAAAGGAACATGCTAGGAAATGGATTTTAAGGGTGTGGGATAATGGTGGGAGGCTCACAATACTGGATCAGACTGAATTTCTCAGTTTAGGTCCTTTGAGCCTCGATAGTGAGTTTAATGTCATAGCCCGCACTGTTGCAGATAATGGTGTGAAGAGTTTGTTTGATTGGTTGGCTGAAGCATGGGTCCAGAGATGGCCTACTACAAGAGAGCTGCAGACGCCTGACGCCCTGGAGTGGTATTCTATTGAGGATGGGATTAAAAGGCTTAGGGAACTTGGAATGATAGAGTGGCTTTGTGTAAAAGCTTCTTGTCCACAGTGGAGGGGCCCGGAAGATGCACCCATCACGAGAGCTATGAGGATAACTTTTGTCCGGGAAACTGTAGAGACTTGGAAGAGCTTCGTTTTTAGCCTCCTCTGTATAAAGGACATAACAGTGGGGAGCGTGGCTGCTCAGTTGCATGATCTAATAGAATTAAGTTTAAAGCCAACAGCAGCTGGCTTGACTGCTGGTTTGGCTCCTGTGGGCTCTGTGTCGGTTCTCTCTCTCTCTCCCTGGAAACATCAAAGCAACAGTTAA

>Fv7CER17 MT077272 [organism=Mus cervicolor]

ATGAATTTCCCACGTGTGCTTGCTGGTTTCTCGAGCTGGCTCTTCAAACCTGAACTTGCCGAGGACTCTCCAGATAATGACGAGAATCCTGTTAACCCATGGCGTGAGCTGCTGCATAAGATAAATGTGGCCGATCTCCCCGATTCATCCTTTTCGAGCGGTAAGGAACTTAATGACTCTGTGTACGATACTTTTGAACATTTCTGCAAGATTAGGGACTATGACGCAGTTGGCGAGCTGCTTCTGGCATTTCTGGATAAAGTAACAAAGGAAAGGGACCAATTCAGAGATGAAATTTCCCAGCTCCGAATGCTCATAAATGATCTAAAGGCTTCTAAGTGTGTCCTGGGGGAGACTCTTCTCTCCTACCGCCACAGGATTGAAGTTGGGGAAAAACAGACTGAAGCCCTCATTGTGAGGTTAGCTGATTTGCAATCCCAGGTCATGTGTCAGCCTGCCCGGAAAGTGTCTGCAGATAAGGTGAGGGCACTGATTGGTAAAGAATGGGATCCGGTAACCTGGGATGGAGATGTGTGGGAGGACATAGATTCTGAGGGGACTGAGGAAGCAGAGTTGCCCACTGTCTTGGCCTCTCCATCCTTGTCTGAGGAAAGTGGTTATGCCTTGTCTAAAGAACGCACCCAGCAGGACAAAGCAGATGTTTCTCAGAACCAGTCTTCAACATCCTTAGTTACTTCTGAACCTGTCACCAGACCCAAGTCTCTGTCTGACCTTACAAGTCAGAAACACCGCCATACTAACCATGAACTCTATTCACTTGCTCAATCAAATCGCCAACAGGCAAAGGAACATGCTAGGAAATGGATTTTAAGGGTGTGGGATAATGGTGGGAGGCTCACAATACTGGATCAGACTGAATTTCTCAGTTTAGGTCCTTTGAGCCTCGATAGTGAGTTTAATGTCATAGCCCGCACTGTTGCAGATAATGGTGTGAAGAGTTTGTTTGATTGGTTGGCTGAAGCATGGGTCCAGAGATGGCCTACTACAAGAGAGCTGCAGACGCCTGACGCCCTGGAGTGGTATTCTATTGAGGATGGGATTAAAAGGCTTAGGGAACTTGGAATGATAGAGTGGCTTTGTGTAAAAGCTTCTTGTCCACAGTGGAGGGGCCCGGAAGATGCACCCATCACGAGAGCTATGAGGATAACTTTTGTCCGGGAAACTGTAGAGACTTGGAAGAGCTTCGTTTTTAGCCTCCTCTGTATAAAAGACATAACAGTGGGGAGCGTGGCTGCTCAGTTGCATGATCTAATAGAATTAAGTTTAAAGCCAACAGCAGCTGGCTTGACTGCTGGTTTGGCTCCTGTGGGCTCTGTGTCGGTTCTCTCTCTCTCTCCCTGGAAACATCAAAGCAACAGTTAA

>Fv7CER18 MT077273 [organism=Mus cervicolor]

ATGAATTTCCCACGTGCGCTTGCTGGTTTCTCGAGCTGGCTCTTCAAACCTGAACTTGCCGAGGACTCTCCAGATAATGACGAGAACCCTGTTAACCCATGGCGTGAGCTGCTGCAGAAGATAAATGTGGCCGATCTCCCCGATTCATCCTTTTCGAGCGGTAAGGAACTTAATGACTCTGTGTACGATACTTTTGAACATTTCTGCAAGATTAGGGACTATGACGCAGTTGGCGAGCTGCTTCTGGCATTTCTGGATAAAGTAACAAAGGAAAGGGACCAATTCAGAGATGAAATTTCCCAGCTCCGAATGCTCATAAATGATCTAAAGGCTTCTAAGTGTGTCCTGGGGGAGGCTCTTCTCTCCTACCGCCACAGGATTGAAGTTGGGGAAAAACAAACTGAAGCCCTCATTGTGAGGTTAGCTGATTTGCAATCCCAGGTCATGTGTCAGCCTGCCCGGAAAGTGTCTGCAGATAAGGTGAGGGCACTGATTGGTAAAGAATGGGATCCGGTAACCTGGGATGGAGATGTGTGGGATGACATAGATTCTGATGGGACTGAGGAAGCAGAGTTGCCCACTGTCTTGGCCTCTCCATCCTTGTCTGAGGAAAGTGGTTATGCCTTGTCTAAAGAACGCACCCAGCAGGACAAAGCAGATGTTTCTCAGAACCAGTCTTCAACATCCTTAGTTACTTCTGAACCTGTCACCAGACCCAAGTCTCTGTCTGACCTTACAAGTCAGAAACACCGCCATACTAACCATGAACTCTATTCACTTGCTCAATCAAATCGCCAACAGGCAAAGGAACATGCTTAGGAAATGGATTTTAAGGGTGTGGGATAATGGTGGGAGGCTCACAATACTGGATCAGACTGAATTTCTCAGTTTAGGTCCTTTGAGCCTCGATAGTGAGTTTAATGTCATAGCCCGCACTGTTGCAGATAATGGTGTGAAGAGTTTGTTTGATTGGTTGGCTGAAGCATGGGTCCAGAGATGGCCTACTACAAGAGAGCTGCAGACGCCTGACGCCCTGGAGTGGTATTCTATTGAGGATGGGATTAAAAGGCTTAGGGAACTTGGAATGATAGAGTGGCTTTGTGTAAAAGCTACTTGTCCACAGTGGAGGGGCCCGGAAGATGCACCCATCACGAGAGCTATGAGGATAACTTTTGTCCGGGAAACTGTAGAGATTTGGAAGAGCTTCGTTTTTAGCCTCCTCTGTATAAAGGACATAACAGTGGGGAGCGTGGCTGCTCAGTTGCATGATCTAATAGAATTAAGTTTAAAGCCAACAGCAGCTGGCTTGACTGCTGGTTTGGCTCCTGTGGGCTCTGTGTCGGTTCTCTCTCTCTCTCCCTGGAAACATCAAAGCAACAGTTAA

>Fv7CER19 MT077274 [organism=Mus cervicolor]

ATGAATTTCCCACGTGTGCTTGCTGGTTTCTCGAGCTGGCTCTTCAAACCTGAACTTGCCGAGGACTCTCCAGATAATGACGTGAACCCTGTTAACCCATGGCGTGAGCTGCTGCAGAAGATAAATGTGGCCGATCTCCCCGATTCATCCTTTTCGAGCGGTAAGGAACTTAATGACTCTGTGTACGATACTTTTGAACATTTCTGCAAGATTAGGGACTATGACGCAGTTGGCGAGCTGCTTCTGGCATTTCTGGATAAAGTAACAAAGGAAAAGGACCAATTCAGAGATGAAATTTCCCAGCTCCGAATGCTCATAAATGATCTAAAGGCTTCTAAGTGTGTCCTGGGGGAGACTCTTCTCTCCTACCGCCACAGGATTGAAGTTGGGGAAAAACAAACTGAAGCCCTCATTGTGAGGTTAGCTGATTTGCAATCCCAGGTCATGTGTCAGCCTGCCCGGAAAGTGTCTGCAACTAAGGTGAGGGCACTGATTGGTAAAGAATGGGATCCGGTAACCTGGGATGGAGATGTGTGGGAGGACATAGATTCTGAGGGGACTGAGGAAGCAGAGTTGCCCACTGTCTTGGCCTCTCCATCCTTGTCTGAGGAAAGTGGTTATGCCTTGTCTAAAGAACGCACCCAGCAGGACAAAACAGATGCTCCTCAGAACCAGTCTTCAACATCCTTAGTTACTTCTGAACCTGTCACCAGACCCAAGTCTCTGTCTGACCTTACAAGTCAGAAACACCGCCATACTAACCATGAACTCTATTCACTTGCTCAATCAAATCGCCAACAGGCAAAGGAACATGCTAGGAAATGGATTTTAAGGGTGTGGGATAATGGTGGGAGGCTCACAATACTGGATCAGACTGAATTTCTCAGTTTAGGTCCTTTGAGCCTCGATAGTGAGTTTAATGTCATAGCCCGCACTGTTGCAGATAATGGTGTGAAGAGTTTGTTTGATTGGTTGGCTGAAGCATGGGTCCAGAGATGGCCTACTACAAGAGAGCTGCAGACGCCTGACGCCCTGGAGTGGTATTCTATTGAGGATGGGATTAAAAGGCTTAGGGAACTTGGAATGATAGAGTGGCTTTGTGTAAAAGCTTCTTGTCCACAGTGGAGGGGCCCGGAAGATGCACCCATCACGAGAGCTATGAGGATAACTTTTGTCCGGGAAACTGTAGAGACTTGGAAGAGCTTCGTTTTTAGCCTCCTCTGTATAAAGGACATAACAGTGGGGAGCGTGGCTGCTCAGTTGCATGATCTAATAGAATTAAGTTTAAAGCCAACAGCAGCTGGCTTGACTGCTGGTTTGGCTCCTGTGGGCTCTGTGTCGGTTCTCTCTCTCTCTCCCTGGAAACATCAAAGCAACAGTTAA

>Fv7CER20 MT077275 [organism=Mus cervicolor]

ATGAATTTCCCACGTGCGCTTGCTGGTTTCTCGAGCTGGCTCTTCAAACCTGAACTTGCCGAGGACTCTCCAGATAATGACGAGAACCCTGTTAACCCATGGCGTGAGCTGCTGCAGAAGATAAATGTGGCCGATCTCCCCGATTCATCCTTTTCGAGCGGTAAGGAACTTAATGACTCTGTGTACGATACTTTTGAACATTTCTGCAAGATTAGGGACTATGACGCAGTTGGCGAGCTGCTTCTGGCATTTCTGGATAAAGTAACAAAGGAAAGGGACCAATTCAGAGATGAAATTTCCCAGCTCCGAATGCTCATAAATGATCTAAAGGCTTCTAAGTGTGTCCTGGGGGAGACTCTTCTCTCCTACCGCCACAGGATTGAAGTTGGGGAAAAACAATCTGAAGCCCTCATTGTGAGGTTAGCTGATTTGCAATCCCAGGTCATGTGTCAGCCTGCCCGGAAAGTGTCTGCAGATAAGGTGAGGGCACTGATTGGTAAAGAATGGGATCCGGTAACCTGGGATGGAGATGTGTGGGAGGACATAGATTCTGATGGGACTGAGGAAGCAGAGTTGCCCACTGTCTTGGCCTCTCCATCCTTGTCTGAGGAAAGTGGTTATGCCTTGTCTAAAGAACGCACCCAGCAGGACAAAACAGATGCTTCTCAGAACCAGTCTTCAACATCCTTAGTTACTTCTGAACCTGTCACCAGACCCAAGTCTCTCTCTGACCTTACAAGTCAGAAACACCGCCATACTAACCATGAACTCTATTCACTTGCTCAATCAAATCGCCAACGGGAAAAGGAACATGCTAGGAAATGGATTTTAAGGGTGTGGGATAATGGTGGGAGGCTCACAATACTGGATCAGACTGAATTTCTCAGTTTAGGTCCTTTGAGCCTCGATAGTGAATTTAATGTCATAGCCCGCACTGTTGCAGATAATGGTGTGAAGAGTTTGTTTGATTGGTTGGCTGAAGCATGGGTCCAGAGATGGCCTACTACAAGAGAGCTGCAGACGCCTGACGCCCTGGAGTGGTATTCTATTGAGGATGGGATTAAAAGGCTTAGGGAACTTGGAATGATAGAGTGGCTTTGTGTAAAAGCTACTTGTCCACAGTGGAGGGGCCCGGAAGATGCACCCATCACGAGAGCTATGAGGATAACTTTTGTCCGGGAAACTGTAGAGACTTGGAAGAGCTTCGTTTTTAGCCTCCTCTGTATAAAGGACATAACAGTGGGGAGCGTGGCTGCTCAGTTGCATGATCTAATAGAATTAAGTTTAAAGCCAACAGCAGCTGGCTTGACTGCTGGTTTGGCTCCTGTGGGCTCTGTGTTGGTTCTCTCTCTCTCTCCCTGGAAACATCAAAGCAACAGTTAA

>Fv7CER21 MT077276 [organism=Mus cervicolor]

ATGAAGATGAATTTCCCACGTGCGCTTGCTGGTTTCTCGAGCTGGCTCTTCAAACCTGAACTTGCCGAGGACTCTCCAGATAATGACGAGAACCCTGTTAACCCATGGCGTGAGCTGCTGCAGAAGATAAATGTGGCCGATCTCCCCGATTCATCCTTTTCGAGCGGTAAGGAACTTAATGACTCTGTGTACGATACTTTTGAACATTTCTGCAAGATTAGGGACTATGACGCAGTTGGCGAGCTGCTTCTGGCATTTCTGGATAAAGTAACAAAGGAAAGGGACCAATTCAGAGATGAAATTTCCCAGCTCCGAATGCTCATAAATGATCTAAAGGCTTCTAAGTGTGTCCTGGGGGAGACTCTTCTCTCCTACCGCCACAGGATTGAAGTTGGGGAAAAACAGACTGAAGCCCTCATTGTGAGGTTAGCTGATTTGCAATCCCAGGTCATGTGTCAGCCTGCCCGGAAAGTGTCTGCAGATAAGGTGAGGGCACTGATTGGTAAAGAATGGGATCCGGTAACCTGGGATGGAGATGTGTGGGAGGACATAGATTCTGAGGGGACTGAGGAAGCAGAGTTGCCCACTGTCTTGGCCTCTCCATCCTTGTCTGAGGAAAGTGGTTATGCCTTGTCTAAAGAACGCACCCAGCAGGACAAAGCAGATGTTTCTCAGAACCAGTCTTCAACATCCTTAGTTACTTCTGAACCTGTCACCAGACCCAAGTCTCTGTCTGACCTTACAAGTCAGAAACACCGCCATACTAACCATGAACTCTATTCACTTGCTCAATCAAATCGCCAACAGGCAAAGGAACATGCTAGGAAATGGATTTTAAGGGTGTGGGATAATGGTGGGAGGCTCACAATACTGGATCAGACTGAATTTCTCAGTTTAGGTCCTTTGAGCCTCGATAGTGAGTTTAATGTCATAGCCCGCACTGTTGCAGATAATGGTGTGAAGAGTTTGTTTGATTGGTTGGCTGAAGCATGGGTCCAGAGATGGCCTACTACAAGAGAGCTGCAGACGCCTGACGCCCTGGAGTGGTATTCTATTGAGGATGGGATTAAAAGGCTTAGGGAACTTGGAATGATAGAGTGGCTTTGTGTAAAAGCTTCTTGTCCACAGTGGAGGGGCCCGGAAGATGCACCCATCACGAGAGCTATGAGGATAACTTTTGTCCGGGAAACTGTAGAGACTTGGAAGAGCTTCGTTTTTAGCCTCCTCTGTATAAAGGACATAACAGTGGGGAGCGTGGCTGCTCAGTTGCATGATCTAATAGAATTAAGTTTAAAGCCAACAGCAGCTGGCTTGACTGCTGGTTTGGCTCCTGTGGGCTCTGTGTCGGTTCTCTCTCTCTCTCCCTGGAAACATCAAAGCAACAGTTAA

>Fv7CER22 MT077277 [organism=Mus cervicolor]

ATGAATTTCCCACGTGCGCTTGCTGGTTTCTCGAGCTGGCTCTTCAAACCTGAACTTGCCGAGGACTCTCCAGATAAAGACGAGAACCCTGTTAACCCATGGCGTGAGCTGCTGCAGAAGATAAATGTGGCCGATCTCCCCGATTCATCCTTTTCGAGCGGTAAGGAACTTAATGACTCTGTGTACGATACTTTTGAACATTTCTGCAAGATTAGGGACTATGACGCAGTTGGCGAGCTGCTTCTGGCATTTCTGGATAAAGTAACAAAGGAAAGGGACCAATTCAGAGATGAAAGTTCCCAGCTCCGAATGCTCATAAATGATCTAAAGGCTTCTAAGTGTGTCCTGGGGGAGACTCTTCTCTCCTACCGCCACAGGATTGAAGTTGGGGAAAAACAGACTGAAGCCCTCATTGTGAGGTTAGCTGATTTGCAATCCCAGGTCATGTGTCAGCCTGCCCGGAAAGTGTCTGCAGATAAGGTGAGGGCACTGATTGGTAAAGAATGGGATCCGGTAACCTGGGATGGAGATGTGTGGGAGGACATAGATTCTGAGGGGACTGAGGAAGCAGAGTTGCCCACTGTCTTGGCCTCTCCATCCTTGTCTGAGGAAAGTGGTTATGCCTTGTCTAAAGAACGCACCCAGCAGGACAAAACAGATGCTCCTCAGAACCAGTCTTCAACATCCTTAGTTACTTCTGAACCTGTCACCAGACCCAAGTCTCTCTCTGACCTTACAAGTCAGAAACACCGCCATACTAACCATGAACTCTATTCACTTGCTCAATCAAATCGCCAACAGGCAAAGGAACATGCTAGGAAATGGATTTTAAGGGTGTGGGATAATGGTGGGAGGCTCACAATACTGGATCAGACTGAATTTCTCAGTTTAGGTCCTTTGAGCCTCGATAGTGAGTTTAATGTCATAGCCCGCACTGTTGCAGATAATGGTGTGAAGAGTTTGTTTGATTGGTTGGCTGAAGCATGGGTCCAGAGATGGCCTACTACAAGAGAGCTGCAGACGCCTGACGCCCTGGAGTGGTATTCTATTGAGGATGGGATTAAAAGGCTTAGGGAACTTGGAATGATAGAGTGGCTTTGTGTAAAAGCTTCTTGTCCACAGTGGAGGGGCCCGGAATATGCACCCATCACGAGAGCTATGAGGATAACTTTTGTCCGGGAAACTGTAGAGACTTGGAAGAGCTTCGTTTTTAGCCTCCTCTGTATAAAGGACATAACAGTGGGGAGCGTGGCTGCTCAGTTGCATGATCTAATAGAATTAAGTTTAAAGCCAACAGCAGCTGGCTTGACTGCTGGTTTGGCTCCTGTGGGCTCTGTGCCGGTTCTCTCTCTCTCTCCCTGGAAACATCAAAGCAACAGTTAA

>Fv7CER23 MT077278 [organism=Mus cervicolor]

ATGAATTTCCCACGTGCTCTTGCTGGTTTCTCGAGCTGGCTCTTCAAACCTGAACTTGCCGAGGACTCTCCAGATAATGACGAGAACCCTGTTAACCCATGGCGTGAGCTGCTGCAGAAGATAAATGTGGCCGATCTCCCCGATTCATCCTTTTCGAGCGGTAAGGAACTTAATGACTCTGTGTACGATACTTTTGAACATTTCTGCAAGATTAGGGACTATGACACAGTTGGCGAGCTGCTTCTGGCATTTCTGGATAAAGTAACAAAGGAAAGGGACCAATTCAGAGATGAAATTTCCCAGCTCCGAATGCTCATAAATGATCTAAAGGCTTCTAAGTGTGTCCTGGGGGAGACTCTTCTCTCCTACCGCCACAGGATTGAAGTTGAGGAAAAACAGACTGAAGCCCTCATTGTGAGGTTAGCTGATTTGCAATCCCAGGTCATGTGTCAGCCTGCCCGGAAAGTGTCTGCAGATAAGGTGAGGGCACTGATTGGTAAAGAATGGGATCCGGTAACCTGGGATGGAGATGTGTGGGAGGACATAGATTCTGAGGGGACTGAGGAAGCAGAGTTGCCCACTGTCTTGGCCTCTCCATCCTTGTCTGAGGAAAGTGGTTATGCCTTGTCTAAAGAACGCACCCAGCAGGACAAAACAGATGCTCCTCAGAACCAGTCTTCAACATCCTTAGTTACTTCTGAACCTGTCACCAGACCCAAGTCTCTGTCTGACCTTACAAGTCAGAAACACCGCCATACTAACCATGAACTCTATTCACTTGCTCAATCAAATCGCCAACAGGCAAAGGAACATGCTAGGAAATGGATTTTAAGGGTGTGGGATAATGGTGGGAGGCTCACAATACTGGATCAGACTGAATTTCTCAGTTTAGGTCCTTTGAGCCTCGATAGTGAGTTTAATGTCATAGCCCGCACTGTTGCAGATAATGGTGTGAAGAGTTTGTTTGATTGGTTGGCTGAAGCATGGGTCCAGAGATGGCCTACTACAAGAGAGCTGCAGACGCCTGACGCCCTGGAGTGGTATTCTATTGAGGATGGGATTAAAAGGCTTAGGGAACTTGGAATGATAGAGTGGCTTTGTGTAAAAGCTTCTTGTCCACAGTGGAGGGGCCCGGAAGATGCACCCATCACGAGAGCTATGAGGATAACTTTTGTCCGGGAAACTGTAGAGACTTGGAAGAGCTTCGTTTTTAGCCTCCTCTGTATAAAGGACATAACAGTGGGGAGCGTGGCTGCTCAGTTGCATGATCTAATAGAATTAAGTTTAAAGCCAACAGCAGCTGGCTTGACTGCTGGTTTGGCTCCTGTGGGCTCTGTGTCGGTTCTCTCTCTCTCTCCCTGGAAACATCAAAGCAACAGTTAA

>Fv7CER24 MT077279 [organism=Mus cervicolor]

ATGAATTTCCCACGTGCGCTTGCTGGTTTCTCGAGCTGGCTCTTCAAACCTGAACTTGCCGAGGACTCTCCAGATAATGACGAGAACCCTGTTAACCCATGGCGTGAGCTGCTGCATAAGATAAATGTGGCCGATCTCCCCGATTCATCCTTTTCGAGCGGTAAGGAACTTAATGACTCTGTGTACGATACTTTTGAACATTTCTGCAAGATTAGGGACTATGACGCAGTTGGCGAGCCGCTTCTGGCATTTCTGGATAAAGTAACAAAGGAAAGGGACCAATTCAGAGATGAAATTTCCCAGCTCCGAATGCACATAAATGATCTAAAGGCTTCTAAGTGTGTCCTGGGGGAGACTCTTCTCTCCTACCGCCACAGGATTGAAGTTGGGGAAAAACAGACTGAAGCCCTCATTGTGAGGTTAGCTGATTTGCAATCCCAGGTCATGTGTCAGCCTGCCCGGAAAGTGTCTGCAGATAAGGTGAGGGCACTGATTGGTAAAGAATGGGATCCGGTAACCTGGGATGGAGATGTGTGGGAGGACATAGATTCTGAGGGAACTGAGGAAGCAGAGTTGCCCACTGTCTTGGCCTCTCCATCCTTGTCTGAGGAAAGTGGTTATGCCTTGTCTAAAGAACGCACCCAGCAGGACAAAGCAGATGTTTCTCAGAACCAGTCTTCAACATCCTTAGTTACTTCTGAACCTGTCACCAGACCCAAGTCTCTGTCTGACCTTACAAGTCAGAAACACCGCCATACTAACCATGAACTCTATTCACTTGCTCAATCAAATCGCCAACAGGCAAAGGAACATGCTAGGAAATGGATTTTAAGGGTGTGGGATAATGGTGGGAGGCTCACAATACTGGATCAGACTGAATTTCTCAGTTTAGGTCCTTTGAGCCTCGATAGTGAGTTTAATGTCATAGCCCGCACTGTTGCAGATAATGGTGTGAAGAGTTTGTTTGATTGGTTGGCTGAAGCATGGGTCCAGAGATGGCCTACTACAAGAGAGCTGCAGACGCCTGATGCCCTGGAGTGGTATTCTATTGAGGATGGGATTAAAAGGCTTAGGGAACTTGGAATGATAGAGTGGCTTTGTGTAAAAGCTTCTTGTCCACAGTGGAGGGGCCCGGAAGATGCACCCATCACGAGAGCTATGAGGATAACTTTTGTCCGGGAAACTGTAGAGACTTGGAAGAGCTTCGTTTTTAGCCTCCTCTGTATAAAGGACATAACAGTGGGGAGCGTGGCTGCTCAGTTGCATGATCTAATAGAATTAAGTTTAAAGCCAACAGCAGCTGGCTTGACTGCTGGTTTGGCTCCTGTGGGCTCTGTGTCGGTTCTCTCTCTCTCTCCCTGGAAACATCAAAGCAACAGTTAA

>Fv7CER25 MT077280 [organism=Mus cervicolor]

ATGAATTTCCCACGTGCGCTTGCTGGTTTCTCGAGCTGGCTCTTCAAACCTGAACTTGCCGAGGACTCTCCAGATAATGACGAGAACCCTGTTAACCCATGGCGTGAGCTGCTGCAGAAGATAAATGTGGCCGATCTCCCCGATTCATCCTTTTCGAGCGGTAAGGAACTTAATGACTCTGTGTACGATACTTTTGAACATTTCTGCAAGATTAGGGACTATGACGCAGTTGGCGAGCTGCTTCTGGCATTTCTGGATAAAGTAACAAAGGAAAGGGACCAATTCAGAGATGAAATTTCCCAGCTCCGAATGCACATAAATGATCTAAAGGCTTCTAAGTGTGTCCTGGGGGAGACTCTTCTCTCCTACCGCCACAGGATTGAAGTTGGGGAAAAACAGACTGAAGCCCTCATTGTGAGGTTAGCTGATTTGCAATCCCAGGTCATGTGTCAGCCTGCCCGGAAAGTGTCTGCAGATAAGGTGAGGGCACTGATTGGTAAAGAATGGGATCCGGTAACCTGGGATGGAGATGTGTGGGAGGACATAGATTCTGATGGGACTGAGGAAGCAGAGTTGCCCACTGTCTTGGCCTCTCCATCCTTGTCTGAGGAAAGTGGTTATGCCTTGTCTAAAGAACGCACCCAGCAGGACAAAGCAGATGTTTCTCAGAACCAGTCTTCAACATCCTTAGTTACTTCTGAACCTGTCACCAGACCCAAGTCTCTGTCTGACCTTACAAGTCAGAAACACCGCCATACTAACCATGAACTCTATTCACTTGCTCAATCAAATCGCCAACAGGCAAAGGAACATGCTAGGAAATGGATTTTAAGGGTGTGGGATAATGGTGGGAGGCTCACAATACTGGATCAGACTGAATTTCTCAGTTTAGGTCCTTTGAGCCTTGATAGTGAGTTTAATGTCATAGCCCGCACTGTTGCAGATAATGGTGTGAAGAGTTTGTTTGATTGGTTGGCTGAAGCATGGGTCCAGAGATGGCCTACTACAAGAGAGCTGCAGACGCCTGACGCCCTGGAGTGGTATTCTATTGAGAATGGGATTAAAAGGCTTAGGGAACTTGGAATGATAGAGTGGCTTTGTGTAAAAGCTTCTTGTCCACAGTGGAGGGGCCCGGAAGATGCACCCATCACGAGAGCTATGAGGATAACTTTTGTCCGGGAGACTGTAGAGACTTGGAAGAGCTTCGTTTTTAGCCTCCTCTGTATAAAGGACATAACAGTGGGGAGCGTGGCTGCTCAGTTGCATGATCTAATAGAATTAAGTTTAAAGCCAACAGCAGCTGGCTTGACTGCTGGTTTGGCTCCTGTGGGCTCTGTGTCGGTTCTCTCTCTCTCTCCCTGGAAACATCAAAGCAACAGTTAA

>Fv7CER26 MT077281 [organism=Mus cervicolor]

ATGAATTTCCCACGTGCGCTTGCTGGTTTCTCGAGCTGGCTCTTCAAACCTGAACTTGCCGAGGACTCTCCAGATAATGACGAGAACCCTGTTAACCCATGGCGTGAGCTGCTGCAGAAGATAAATGTGGCCGATCTCCCCGATTCATCCTTTTCGAGCGGTAAGGAACTTAATGACTCTGTGTACGATACTTTTGAACATTTCTGCAAGATTAGGGACTATGACGCAGTTGGCGAGCTGCTTCTGGCATTTCTGGATAAAGTAACAAAGGAAAGGGACCAATTCAGAGATGAAAGTTCCCAGCTCCGAATGCTCATAAATGATCTAAAGGCTTCTAAGTGTGTCCTGGGGGAGACTCTTCTCTCCTACCGCCACAGGATTGAAGTTGGGGAAAAACAGACTGAAGCCCTCATTGTGAGGTTAGCTGATTTGCAATCCCAGGTCATGTGTCAGCCTGCCCGGAAAGTGTCTGCAGATAAGGTGAGGGCACTGATTGGTAAAGAATGGGATCCGGTAACCTGGGATGGAGATGTGTGGGAGGACATAGATTCTGATGGGACTGAGGAAGCAGAGTTGCCCACTGTCTTGGCCTCTCCATCCTTGTCTGAGGAAAGTGGTTATGCCTTGTCTAAAGAACGCACCCAGCAGGACAAAACAGATGCTCCTCAGAACCAGTCTTCAACATCCTTAGTTACTTCTGAACCTGTCACCAGACCCAAGTCTCTGTCTGACCTTACAAGTCAGAAACACCGCCATACTAACCATGAACTCTATTCACTTGCTCAATCAAATCGCCAACAGGCAAAGGAACATGCTAGGAAATGGATTTTAAGGGTGTGGGATAATGGTGGGAGGCTCACAATACTGGATCAGACTGAATTTCTCAGTTTAGGTCCTTTGAGCCTTGATAGTGAGTTTAATGTCATAGCCCACACTGTTGCAGATAATGGTGTGAAGAGTTTGTTTGATTGGTTGGCTGAAGCATGGGTCCAGAGATGGCCTACTACAAGAGAGCTGCAGACGCCTGACGCCCTGGAGTGGTATTCTATTGAGGATGGGATTAAAAGGCTTAGGGAACTTGGAATGATAGAGTGGCTTTGTGTAAAAGCTACTTGTTCACAGTGGAGGGGCCCGGAAGATGCACCCATCACGAGAGCTATGAGGATAACTTTTGTCCGGGAAACTGTAGAGACTTGGAAGAGCTTCGTTTTTAGCCTCCTCTGTATAAAGGACATAACAGTGGGGAGCGTGGCTGCTCAGTTGCATGATCTAATAGAATTAAGTTTAAAGCCAACAGCAGCTGGCTTGACTGCTGGTTTGGCTCCTGTGGGCTCTGTGTCGGTTCTCTCTCTCCCTGGAAACATCAAAGCAACAGTTAAAGAGCTTAAAATCAGGAGGTCCCGCAGTGGAGACAGAGGACAGAGATGA

>Fv7CER27 MT077282 [organism=Mus cervicolor]

ATGAATTTCCCACGTGCGCTTGCTGGTTTCTCGAGCTGGCTCTTCAAACCTGAACTTGCCGAGGACTCTCCAGATAATGACGAGAACCCTGTTAACCCATGGCGTGAGCTGCTGCAGAAGATAAATGTGGCCGATCTCCCCGATTCATCCTTTTCGAGCGGTAAGGAACTTAATGACTCTGTGTACGATACTTTTGAACATTTCTGCAAGATTAGGGACTATGACGCAGTTGGCGAGCTGCTTCTGGCATTTCTGGATAAAGTAACAAAGGAAAGGGACCAATTCAGAGATGAAATTTCCCAGCTCCGAATGCTCATAAATGATCTAAAGGCTTCTAAGTGTGTCCTGGGGGAGACTCTTCTCTCCTACCGCCACAGGATTGAAGTTGGGGAAAAACAGACTGAAGCCCTCATTGTGAGGTTAGCTGATTTGCAATCCCAGGTCATGTGTCAGCCTGTCCGGAAAGTGTCTGCAGATAAGGTGAGGGCACTGATTGGTAAAGAATGGGATCCGGTAACCTGGGATGGAGATGTGTGGGAGGACATAGATTCTGATGGGACTGAGGAAGCAGAGTTGCCCACTGTCTTGGCCTCTCCATCCTTGTCTGAGGAAAGTGGTTATGCCTTGTCTAAAGAACGCACCCAGCAGGACAAAGCAGATGTTTCTCAGAACCAGTCTTCAACATCCTTAGTTACTTCTGAACCTGTCACCAGACCCAAGTCTCTGTCTGACCTTACAAGTCAGAAACACCGCCATACTAACCATGAACTCTATTCACTTGCTCAATCAAATCGCCAACGGGAAAAGGAACATGCTAGGAAATGGATTTTAAGGGTGTGGGATAATGGTGGGAGGCTCACAATACTGGATCAGACTGAATTTCTCAGTTTAGGTCCTTTGAGCCTCGATAGTGAGTTTAATGTCATAGCCCGCACTGTTGCAGATAATGGTGTGAAGAGTTTGTTTGATTGGTTGGCTGAAGCATGGGTCCAGAGATGGCCTACTACAAGAGAGCTGCAGACGCCTGACGCCCTGGAGTGGTATTCTATTGAGGATGGGATTAAAAGGCTTAGGGAACTTGGAATGATAGAGTGGCTTTGTGTAAAAGCTACTTGTCCACAGTGGAGGGGCCCGGAAGATGCACCCATCACGAGAGCTATGAGGATAACTTTTGTCCGGGAAACTGTAGAGACTTGGAAGAGCTTCGTTTTTAGCCTCCTCTGTATAAAGGACATAACAGTGGGGAGCGTGGCTGCTCAGTTGCATGATCTAATAGAATTAAGTTTAAAGCCAACAGCAGCTGGCTTGACTGCTGGTTTGGCTCCTGTGGGCTCTGTGTCGGTTCTCTCTCTCTCTCCCTGGAAACATCAAAGCAACAGTTAA

>Fv7CER28 MT077283 [organism=Mus cervicolor]

ATGAATTTCCCACGTGCGCTTGCTGGTTTCTCGAGCTGGCTCTTCAAACCTGAACTTGCCGAGGACTCTCCAGATAATGACGAGAACCCTGTTAACCCATGGCGTGAGCTGCTGCAGAAGATAAATGTGGCCGATCTCCCCGATTCATCCTTTTCGAGCGGTAAGGAACTTAATGACTCTGTGTACGATACTTTTGAACATTTCTGCAAGATTAGGGACTATGACGCAGTTGGCGAGCTGCTTCTGGCATTTCTGGATAAAGTAACAAAGGAAAGGGACCAATTCAGAGATGAAATTTCCCAGCTCCGAATGCTCATAAATGATCTAAAGGCTTCTAAGTGTGTCCTGGGGGAGACTCTTCTCTCCTACCGCCACAGGATTGAAGTTGGGGAAAAACAAACTGAAGCCCTCATTGTGAGGTTAGCTGATTTGCAATCCCAGGTCATGTGTCAGCCTGCCCGGAAAGTGTCTGCAGATAAGGTGAGGGCACTGATTGGTAAAGAATGGGATCCGGTAACCTGGGATGGAGATGTGTGGGAGGACATAGATTCTGAGGGGACTGAGGAAGCAGAGTTGCCCACTGTCTTGGCCTCTCCATCCTTGTCTGAGGGAAGTGGTTATGCCTTGTCTAAAGAACGCACCCAGCAGGACAAAGCAGATGTTTCTCAGAACCAGTCTTCAACATCCTTAGTTACTTCTGAACCTGTCACCAGACCCAAGTCTCTGTCTGACCTTACAAGTCAGAAACACCGCCATACTAACCATGAACTCAAATCACTTGCTCAATCAAATCGCCAACGGGAAAAGGAACATGCTAGGAAATGGATTTTAAGGGTGTGGGATAATGGTGGGAGGCTCACAATACTGGATCAGACTGAATTTCTCAGTTTAGGTCCTTTGAGCCTCGATAGTGAGTTTAATGTCATAGCCCGCACTGTTGCAGATAATGGTGTGAAGAGTTTGTTTGATTGGTTGGCTGAAGCATGGGTCCAGAGATGGCCTACTACAAGAGAGCTGCAGACGCCTGACGCCCTGGAATGGTATTATATTGAGGATGGGATTAAAAGGCTTAGGGAACTTGGAATGATAGAGTGGCTTTGTGTAAAAGCTACTTGTCCACAGTGGAGGGGCCCGGAAGATGCACCCATCACGAGAGCTATGAGGATAACTTTTGTCCGGGAAACTGTAGAGACTTGGAAGAGCTTCGTTTTTAGCCTCCTCTGTATAAAGGACATAACAGTGGGGAGCGTGGCTGCTCAGTTGCATGATCTAATAGAATTAAGTTTAAAGCCAACAGCAGCTGGCTTGACTGCTGGTTTGGCTCCTGTGGGCTCTGTGTCGGTTCTCTCTCTCTCTCTCCCTGGAAACATCAAAGCAACAGTTAAAGAGCTTAAAATCAGGAGG

>Fv7CER29 MT077284 [organism=Mus cervicolor]

ATGAATTTCCCACGTGCGCTTGCTGGTTTCTCGAGCTGGCTCTTCAAACCTGAACTTGCCGAGGACTCTCCAGATAATGACGAGAACACTGTTAACCCATGGCGTGAGCTGCTGCAGAAGATAAATGTGGCCGATCTCCCCGATTCATCCTTTTCGAGCGGTAAGGAACTTAATGACTCTGTGTACGATACTTTTGAACATTTCTGCAAGATTAGGGACTATGACGCAGTTGGCGAGCTGCTTCTGGCATTTCTGGATAAAGTAACAAAGGAAAGGGACCAATTCAGAGATGAAATTTCCCAGCTCCGAATGCACATAAATGATCTAAAGGCTTCTAAGTGTGTCCTGGGGGAGACTCTTCTCTCCTACCGCCACAGGATTGAAGTTGGGGAAAAACAGACTGAAGCCCTCATTGTGAGGTTAGCTGATTTGCAATCCCAGGTCATGTGTCAGCCTGCCCAGAAAGTGTCTGCAGATAAGGTGAGGGCACTGATTGGTAAAGAATGGGATCCGGTAACCTGGGATGGAGATGTGTGGGAGGACATAGATTCTGAGGGGACTGAGGAAGCAGAGTTGCCCACTGTCTTGGCCTCTCCATCCTTGTCTGAGGAAAGTGGTTATGCCTTGTCTAAAGAACGCACCCAGCAGGACAAAACAGATGCTCCTCAGAACCAGTCTTCAACATCCTTAGTTACTTCTGAACCTGTCACCAGACCCAAGTCTCTGTCTGACCTTACAAGTCAGAAACACCGCCATACTAACCATGAACTCTATTCACTTGCTCAATCAAATCGCCAACGGGAAAAGGAACATGCTAGGAAATGGATTTTAAGGGTGTGGGATAATGGTGGGAGGCTCACAATACTGGATCAGACTGAATTTCTCAGTTTAGGTCCTTTGAGCCTTGATAGTGAGTTTAATGTCATAGCCCACACTGTTGCAGATAATGGTGTGAAGAGTTTGTTTGATTGGTTGGCTGAAGCATGGGTCCAGAGATGGCCTACTACAAGAGAGCTGCAGAGGCCTGACGCCCTGGAGTGGTATTCTATTGAGGATGGGATTAAAAGGCTTAGGGAACTTGGAATGATAGAGTGGCTTTGTGTAAAAGCTTCTTGTCCACAGTGGAGGGGCCCGGAAGATGCACCCATCACGAGAGCTATGAGGATAACTTTTGTCCGGGAAACTGTAGAGACTTGGAAGAGCTTCGTTTTTAGCCTCCTCTGTATAAAGGACATAACAGTGGGGAGCGTGGCTGCTCAGTTGCATGATCTAATAGAATTAAGTTTAAAGCCAACAGCAGCTGGCTTGACTGCTGGTTTGGCTCCTGTGGGCTCTGTGTCGGTTCTCTCTCTCTCTCCCTGGAAACATCAAAGCAACAGTTAA

>Fv7CER30 MT077285 [organism=Mus cervicolor]

ATGAATTTCCCACGTGCGCTTGCTGGTTTCTCGAGCTGGCTCTTCAAACCTGAACTTGCCGAGGACTCTCCAGATAATGACGAGAACCCTGTTAACCCATGGCGTGAGCTGCTGCAGAAGATAAATGTGGCCGATCTCCCCGATTCATCCTTTTCGAGCGGTAAGGAACTTAATGACTCTGTGTACGATACTTTTGAACATTTCTGCAAGATTAGGGACTATGACGCAGTTGGCAAGCTGCTTCTGGCATTTCTGGATAAAGTAACAAAGGAAAGGGACCAATTCAGAGATGAAATTTCCCAGCTCCGAATGCTCATAAATGATCTAAAGGCTTCTAAGTGTGTCCTGGGGGAGACTCTTCTCTCCTACCGCCACAGGATTGAAGTTGGGGAAAAACAGACTGAAGCCCTCATTGTGAGGTTAGCTGATGTGCAATCCCAGGTCATGTGTCAGCCTGCCCGGAAAGTGTCTGCAGATAAGGTGAGGGCACTGATTGGTAAAGAATGGGATCCGGTAACCTGGGATGGAGATGTGTGGGAGGACATAGATTCTGAGGGGACTGAGGAAGCAGAGTTGCCCACTGTCTTGGCCTCTCCATCCTTGTCTGAGGAAAGTGGTTATGCCTTGTCTAAAGAACGCACCCAGCAGGACAAAACAGATGCTCCTCAGAACCAGTCTTCAACATCCTTAGTTACTTCTGAACCTGTCACCAGACCCAAGTCTCTGTCTGACCTTACAAGTCAGAAACACCGCCATACTAACCATGAACTCTATTCACTTGCTCAATCAAATCGCCAACGGGAAAAGGAACATGCTAGGAAATGGATTTTAAGGGTGTGGGATAATGGTGGGAGGCTCACAATACTGGATCAGACTGAATTTCTCAGTTTAGGTCCTTTGAGCCTCGATAGTGAGTTTAATGTCATAGCCCGCACTGTTGCAGATAATGGTGTGAAGAGTTTGTTTGATTGGTTGGCTGAAGCATGGGTCCAGAGATGGCCTACTACAAGAGAGCTGCAGACGCCTGACGCCCTGGAGTGGTATTCTATTGAGGATGGGATTAAAAGGCTTAGGGAACTTGGAATGATAGAGTGGCTTTGTGTAAAAGCTACTTGTCCACAGTGGAGGGGCCCGGAAGATGCACCCATCACGAGAGCTATGAGGATAACTTTTGTCCGGGAAACTGTAGAGACTTGGAAGAGCTTCGTTTTTAGCCTCCTCTGTATAAAGGACATAACAGTGGGGAGCGTGGCTGCTCAGTTGCATGATCTAATAGAATTAAGTTTAAAGCCAACAGCAGCTGGCTTGACTGCTGGTTTGGCTCCTGTGGGCTCTGTGTCGGTTCTCTCTCTCTCTCCCTGGAAACATCAAAGCAACAGTTAA

>Fv7CER31 MT077286 [organism=Mus cervicolor]

ATGAATTTCCCACGTGCGCTTGCTGGTTTCTCGAGCTGGCTCTTCAAACCTGAACTTGCCGAGGACTCTCCAGATAATGATGAGAACCCTGTTAACCCATGGCGTGAGCTGCTGCAGAAGATAAATGTGGCCGATCTCCCCGATTCATCCTTTTCGAGCGGTAAGGAACTTAATGACTCTGTGTACGATACTTTTGAACATTTCTGCAAGATTAGGGACTATGACGCAGTTGGCGAGCTGCTTCTGGCATTTCTGGATAAAGTAACAAAGGAAAGGGACCAATTCAGAGATGAAATTTCCCAGCTCCGAATGCTCATAAATGATCTAAAGGCTTCTAAGTGTGTCCTGGGGGAGACTCTTCTCTCCTACCGCCACAGGATTGAAGTTGAGGAAAAACAGACTGAAGCCCTCATTGTGAGGTTAGCTGATTTGCAATCCCAGGTCATGTGTCAGCCTGCCCGGAAAGTGTCTGCAACTAAGGTGAGGGCACTGATTGGTAAAGAATGGGATCCCGTAACCTGGGATGGAGATGTGTGGGAGGACATAGATTCTGAGGGGACTGAGGAAGCAGAGTTGCCCACTGTCTTGGCCTCTCCATCCTTGTCTGAGGAAAGTGGTTATGCCTTGTCTAAAGAACGCACCCAGCAGGACAAAGCAGATGTTTCTCAGAACCAGTCTTCAACATCCTTAGTTACTTCTGAACCTGTCACCAGACCCAAGTCTCTGTCTGACCTTACAAGTCAGAAACACCGCCATACTAACCATGAACTCTATTCACTTGCTCAATCAAATCGCCAACAGGCAAAGGAACATGCTAGGAAATGGATTTTAAGGGTGTGGGATAATGGTGGGAGGCTCACAATACTGGATCAGACTGAATTTCTCAGTTTAGGTCCTTTGAGCCTTGATAGTGAGTTTAATGTCATAGCCCGCACTGTTGCAGATAATGGTGTGAAGAGTTTGTTTGATTGGTTGGCTGAAGCATGGGTCCAGAGATGGCCTACTACAAGAGAGCTGCAGACGCCTGACGCCCTGGAGTGGTATTCTATTGAGGATGGGATTAAAAGGCTTAGGGAACTTGGAATGATAGAGTGGCTTTGTGTAAAAGCTTCTTGTCCACAGTGGAGGGGCCCGGAAGATGCACCCATCACGAGAGCTATGAGGATAACTTTTGTCCGGGAAACTGTAGAGACTTGGAAGAGCTTCGTTTTTAGCCTCCTCTGTATAAAGGACATAACAGTGGGGAGCGTGGCTGCTCAGTTGCATGATCTAATAGAATTAAGTTTAAAGCCAACAGCAGCTGGCTTGACTGCTGGTTTGGCTCCTGTGGGCTCTGTGTCGGTTCTCTCTCTCTCTCCCTGGAAACATCAAAGCAACAGTTAA

>Fv7CER32 MT077287 [organism=Mus cervicolor]

ATGAATTTCCCACGTGCGCTTGCTGGTTTCTCGAGCTGGCTCTTCAAACCTGAACTTGCCGAGGACTCTCCAGATAAAGACGAGAACCCTGTTAACCCATGGCGTGAGCTGCTGCAGAAGATAAATGTGGCCGATCTCCCCGATTCATCCTTTTCGAGCGGTAAGGAACTTAATGACTCTGTGTACGATACTTTTGAACATTTCTGCAAGATTAGGGACTATGACGCAGTTGGCGAGCTGCTTCTGGCATTTCTGGATAAAGTAACAAAGGAAAGGGACCAATTCAGAGATGAAATTTCCCAGCTCCGAATGCTCATAAATGATCTAAAGGCTTCTAAGTGTGTCCTGGGGGAGACTCTTCTCTCCTACCGCCACAGGATTGAAGTTGGGGAAAAACAGACTGAAGCCCTCATTGTGAGGTTAGCTAATTTGCAATCCCAGGTCATGTGTCAGCCTGCCCGGAAAGTGTCTGCAGATAAGGTGAGGGCACTGATTGGTAAAGAATGGGATCCCGTTACCTGGGATGGAGATGTGTGGGAGGACATAGATTCTGAGGGAACTGAGGAAGCAGAGTTGCCCACTGTCTTGGCCTCTCCATCCTTGTCTGAGGAAAGTGGTTATGCCTTGTCTAAAGAACGCACCCAGCAGGACAAAGCAGATGTTTCTCAGAACCAGTCTTCAACATCCTTAGTTACTTCTGAACCTGTCACCAGACCCAAGTCTCTGTCTGACCTTACAAGTCAGAAACACCGCCATACTAACCATGAACTCTATTCACTTGCTCAATCAAATCGCCAACAGGCAAAGGAACATGCTAGGAAATGGATTTTAAGGGTGTGGGATAATGGTGGGAGGCTCACAATACTGGATCAGACTGAATTTCTCAGTTTAGGTCCTTTGAGCCTCGATAGTGAGTTTAATGTCATAGCCCGCACTGTTGCAGATAATGGTGTGAAGAGTTTGTTTGATTGGTTGGCTGAAGCATGGGTCCAGAGATGGCCTACTACAAGAGAGCTGCAGACGCCTGACGCCCTGGAGTGGTATTCTATTGAGGATGGGATTAAAAGGCTTAGGGAACTTGGAATGATAGAGTGGCTTTGTGTAAAAGCTACTTGTCCACAGTGGAGGGGCCCGGAAGATGCACCCATCACGAGAGCTATGAGGATAACTTTTGTCCGGGAAACTGTAGAGACTTGGAAGAGCTTCGTTTTTAGCCTCCTCTGTATAAAGGACATAACAGTGGGGAGCGTGGCTGCTCAGTTGCATGATCTAATAGAATTAAGTTTAAAGCCAACAGCAGCTGGCTTGACTGCTGGTTTGGCTCCTGTGGGCTCTGTGTCAGTTCTCTCTCTCTCTCCCTGGAAACATCAAAGCAACAGTTAA

>Fv7CER33 MT077288 [organism=Mus cervicolor]

ATGAATTTCCCACGTGCGCTTGCTGGTTTCTCGAGCTGGCTCTTCAAACCTGAACTTGCCGAGGACTCTCCAGATAATGACGAGAACCCTGTTAACCCATGGCGTGAGCTGCTGCAGAAGATAAATGTGGCCGATCTCCCCGATTCATCCTTTTCGAGCGGTAAGGAACTTAATGACTCTGTGTACGATACTTTTGAACATTTCTGCAAGATTAGGGACTATGACGCAGTTGGCGAGCTGCTTCTGGCATTTCTGGATAAAGTAACAAAGGAAAGGGACCAATTCAGAGATGAAATTTCCCAGCTCCGAATGCTCATAAATGATCTAAAGGCTTCTAAGTGTGTCCTGGGGGAGACTCTTCTCTCCTACCGCCACAGGATTGAAGTTGGGGAAAAACAGACTGAAGCCCTCATTGTGAGGTTAGCTGATTTGCAATCCCAGGTCATGTGTCAGCCTGTCCGGAAAGTGTCTGCAGATAAGGTGAGGGCACTGATTGGTAAAGAATGGGATCCGGTAACCTGGGATGGAGATGTGTGGGAGGACATAGATTCTGATGGGACTGAGGAAGCAGAGTTGCCCACTGTCTTGGCCTCTCCATCCTTGTCTGAGGAAAGTGGTTATGCCTTGTCTAAAGAACGCACCCAGCAGGACAAAGCAGATGTTTCTCAGAACCAGTCTTCAACATCCTTAGTTACTTCTGAACCTGTCACCAGACCCAAGTCTCTGTCTGACCTTACAAGTCAGAAACACCGCCATACTAACCATGAACTCTATTCACTTGCTCAATCAAATCGCCAACAGGCAAAGGAACATGCTAGGAAATGGATTTTAAGGGTGTGGGATAATGGTGGGAGGCTCACAATACTGGATCAGACTGAATTTCTCAGTTTAGGTCCTTTGAGCCTCGATAGTGAGTTTAATGTCATAGCCCGCACTGTTGCAGATAATGGTGTGAAGAGTTTGTTTGATTGGTTGGCTGAAGCATGGGTCCAGAGATGGCCTACTACAAGAGAGCTGCAGACGCCTGACGCCCTGGAGTGGTATTCTATTGAGGATGGGATTAAAAGGCTTAGGGAACTTGGAATGATAGAGTGGCTTTGTGTAAAAGCTACTTGTCCACAGTGGAGGGGCCCGGAAGATGCACCCATCACGAGAGCTATGAGGATAACTTTTGTCCGGGAAACTGTAGAGACTTGGAAGAGCTTCGTTTTTAGCCTCCTCTGTATAAAGGACATAACAGTGGGGAGCGTGGCTGCTCAGTTGCATGATCTAATAGAATTAAGTTTAAAGCCAACAGCAGCTGGCTTGACTGCTGGTTTGGCTCCTGTGGGCTCTGTGTCGGTTCTCTCTCTCTCTCCCTGGAAACATCAAAGCAACAGTTAA

>Fv7CER34 MT077289 [organism=Mus cervicolor]

ATGAATTTCCCACGTGCGCTTGCTGGTTTCTCGAGCTGGCTCTTCAAACCTGAACTTGCCGAGGACTCTCCAGATAATGATGAGAACCCTGTTAACCCATGGCGTGAGCTGCTGCAGAAGATAAATGTGGCCGATCTCCCCGATTCATCCTTTTCGAGCGGTAAGGAACTTAATGACTCTGTGTACGATACTTTTGAACATTTCTGCAAGATTAGGGACTATGACGCAGTTGGCGAGCTGCTTCTGGCATTTCTGGATAAAGTAACAAAGGAAAGGGACCAATTCAGAGATGAAATTTCCCAGCTCCGAATGCTCATAAATGATCTAAAGGCTTCTAAGTGTGTCCTGGGGGAGACTCTTCTCTCCTACCGCCACAGGATTGAAGTTGGGGAAAAACAGACTGAAGCCCTCATTGTGAGGTTAGCTGATTTGCAATCCCAGGTCATGTGTCAGCCTGCCCGGAAAGTGTCTGCAACTAAGATGAGGGCACTGATTGGTAAAGAATGGGATCCGGTAACCTGGGATGGAGATGTGTGGGAGGACATAGATTCTGATGGGACTGAGGAAGCAGAGTTGCCCACTGTCTTGGCCTCTCCATCCTTGTCTGAGGAAAGTGGTTATGCCTTGTCTAAAGAACGCACCCAGCAGGACAAAGCAGATGTTTCTCAGAACCAGTCTTCAACATCCTTAGTTACTTCTGAACCTGTCACCAGACCCAAGTCTCTGTCTGACCTTACAAGTCAGAAACACCGCCATACTAACCATGAACTCTATTCACTTGCTCAATCAAATCGCCAACAGGCAAAGGAACATGCTAGGAAATGGATTTTAAGGGTGTGGGATAATGGTGGGAGGCTCACAATACTGGATCAGACTGAATTTCTCAGTTTAGGTCCTTTGAGCCTCGATAGTGAGTTTAATGTCATAGCCCGCACTGTTGCAGATAATGGTGTGAAGAGTTTGTTTGATTGGTTGGCTGAAGCATGGGTCCAGAGATGGCCTACTACAAGAGAGCTGCAGACGCCTGACGCCCTGGAGTGGTATTCTATTGAGGATGGGATTAAAAGGCTTAGGGAACTTGGAATGATAGAGTGGCTTTGTGTAAAAGCTTCTTGTCCACAGTGGAGGGGCCCGGAAGATGCACCCATCACGAGAGCTATGAGGATAACTTTTGTCCGGGAAACTGTAGAGACTTGGAAGAGCTTCCTTTTTAGCCTCCTCTGTATAAAGGACATAACAGTGGGGAGCGTGGCTGCTCAGTTGCATGATCTAATAGAATTAAGTTTAAAGCCAACAGCAGCTGGCTTGACTGCTGGTTTGGCTCCTGTGGGCTCTGTGTCGGTTCTCTCTCTCTCTCCCTGGAAACATCAAAGCAACAGTTAA

>Fv1COO1 MT077290 [organism=Mus cookii]

ATGAATTTCCCACGTGCGCTTGCTGGTTTCTCGAGCTGGCTCTTCAAACCTGAACTTGCCGAGGACTCTCCGGATAATGACGAGAACACTGTTAACCCATGGCGTGAGCTGCTGCAGAAGATAAATGTGGCCGATCTCCCCGATTCATCCTTTTCGAGCGGTAAGGAACTTAATGACTCTGTGTACGATACTTTTGAACATTTCTGCAAGATTAGGGACTATGACGCAGTTGGCGAGCTGCTTCTGGCATTTCTGGATAAAGTAACAAAGGAAAGGGACCAATTCAGAGATGAAATTTCCCAGCTCCGAATGCACATAAATGATCTAAAGGCTTCTAAGTGTGTCCTGGGGGAGACTCTTCTCTCCTACCGCCACAGGATTGAAGTTGGGGAAAAACAGACTGAAGCCCTCATTATGAGGTTAGCTGATGTGCAATCCCAGGTCATGTGTCAGCCTGCCCGGAAAGTGTCTGCAGATAAGGTGAGGGCACTGATTGGTAAAGAATGGGATCCCGTTACCTGGGATGGAGATGTGTGGGAGGACATAGATTCTGATGGGACTGAGGAAGCAGAGTTGCCCACTGTCTTGGCCTCTCCATCCTTGTCTGAGGAAAGTGGTTATGCCTTGTCTAAAGAACGCACCCAGCAGGACAAAGCAGATGCTCCTCAGAACCAGTCTTCAACATCCTTAGTTACTTCTGAACCTGTCACCAGACCCAAGTCTCTGTCTGACCTTACAAGTCAGAAACACCGCCATACTAACCATGAACTCTATTCACTTGCTCAATCAAATCGCCAACGGGAAAAGGAACATGCTAGGAAATGGATTTTAAGGGTGTGGGATAATGGTGGGAGGCTCACAATACTGGATCAGATTGAATTTCTCAGTTTAGGTCCTTTGAGCCGAGATAGTGAGTTTAATGTCATAGCCCGCACTGTTGCAGATAATGGTGTGAAGAGTTTGTTTGATTGGTTGGCTGAAGCATGGGTCCAGAGATGGCCTACTACAAGAGAGCTACAGACGCCTGACGCCCTGGTGTGGTATTCTATTGAGGATGGGATTAAAAGGCTTAGGGAACTTGGAATGATAGAGTGGCTTTGTGTAAAAGCTACTTGTCCACAGTGGAGGGGCCCGGAAGATGCACCCATCACGAGAGCTATGAGGATAACTTTTGTCCGGGAAACTGTAGAGACTTGGAAGAGCTTCGTATTTAGCCTCCTCTGTATAAAGGACATAACAGTGGGGAGCGTGGCTGCTCAGTTGCATGATCTAATAGAATTAAGTTTAAAGCCAGCCGGGGGCGTGGTGGCGCACGCTTTTAATCCTAGCACTCGGGAGGCAGAGGCAGGCGGATTTCTGAGTTCAAGGCCAGCCTGGTCTACAAAGTGA

>Fv1COO2 MT077291 [organism=Mus cookii]

ATGAATTTCCCACGTGCGCTTGCTGGTTTCTCGAGCTGGCTCTTCAAACCTGAACTTGCCGAGGACTCTCCGGATAATGACGAGAACACTGTTAACCCATGGCGTGAGCTGCTGCAGAAGATAAATGTGGCCGATCTCCCCGATTCATCCTTTTCGAGCGGTAAGGAACTTAATGACTCTGTGTACGATACTTTTGAACATTTCTGCAAGATTAGGGACTATGACGCAGTTGGCGAGCTGCTTCTGGCATTTCTGGATAAAGTAACAAAGGAAAGGGACCAATTCAGAGATGAAATTTCCCAGCTCCGAAAGCACATAAATGATCTAAAGGCTTCTAAGTGTGTCCTGGGGGAGACTCTTCTCTCCTACCGCCACAGGATTGAAGTTGGGGAAAAACAGACTGAAGCCCTCATTATGAGGTTAGCTGATGTGCAATCCCAGGTCATGTGTCAGCCTCCCCGGAAAGTGTCTGCAGATAAGGTGAGGGCACTGATTGGTAAAGAATGGGATCCCGTTACCTGGGATGGAGATGTGTGGGAGGACATAGATTCTGAGGGGACTGAGGAAGCAGAGTTGCCCACTGTCTTGGCCTCTCCATCCTTGTCTGAGGAAAGTGGTTATGCCTTGTCTAAAGAACGCACCCAGCAGGACAAAGCAGATGCTCCTCAGAACCAGTCTTCAACATCCTTAGTTACTTCTGAACCTGTCACCAGACCCAAGTCTCTGTCTGACCTTACAAGTCAGAAACACCGCCATACTAACCATGAACTCTATTCACTTGCTCAATCAAATCGCCAAAAGGAAAAGGAACATGCTAGGAAATGGATTTTAAGGGTGTGGGATAATGGTGGGAGGCTCACAATACTGGATCAGATTGAATTTCTCAGTTTAGGTCCTTTGAGCCTAGATAGTGAGTTTAATGTCATAGCCCGCACTGTTGCAGATAATGGTGTGAAGAGTTTGTTTGATTGGTTGGCTGAAGCATGGGTCCAGAGATGGCCTACTACAAGAGAGCTGCAGACGCCTGACGCCCTGGAGTGGTATTCTATTGAGGATGGGATTAAAAGGCTTAGGGAACTTGGAATGATAGAGTGGCTTTGTGTAAAAGCTACTTGTCCACAGTGGAGGGGCCCGGAAGATGCACCCATCACAAGAGCTATGAGGATAACTTTTGTCCGGGAAACTGTAGAGACTTGGAAGAGCTTCGTATTTAGCCTCCTCTGTATAAAGGACATAACAGTGGGGAGCGTGGCTGCTCAGTTGCATGATCTAATAGAATTAAGTTTAAAGCCAGCCGGGCGTGGTGGCGCACGCTTTTAA

>Fv1COO3 MT077292 [organism=Mus cookii]

ATGAATTTCCCACGTGCGCTTGCTGGTTTCTCGAGCTGGCTCTTCAAACCTGAACTTGCCGAGGACTCTCCGGATAATGACGAGAACACTGTTAACCCATGGCGTGAGCTGCTGCAGAAGATAAATGTGGCCGATCTCCCCGATTCATCCTTTTCGAGCGGTAAGGAACTTAATGACTCTGTGTACGATACTTTTGAACATTTCTGCAAGATTAGGGACTATGACGCAGTTGGCGAGCTGCTTCTGGCATTTCTGGATAAAGTAACAAAGGAAAGGGACCAATTCAGAGATGAAATTTCCCAGCTCCGAAAGCACATAAATGATCTAAAGGCTTCTTAGTGTGTCCTGGGGGAGACTCTTCTCTCCTACCGCCACAGGATTGAAGTTGGGGAAAAACAGACTGAAGCCCTCATTATGAGGTTAGCTGATGTGCAATCCCAGGTCATGTGTCAGCCTCCCCGGAAAGTGTCTGCAGATAAGGTGAGGGCACTGATTGGTAAAGAATGGGATCCCGTTACCTGGGATGGAGATGTGTGGGAGGACATAGATTCTGAGGGGACTGAGGAAGCAGAGTTGCCCACTGTCTTGGCCTCTCCATCCTTGTCTGAGGAAAGTGGTTATGCCTTGTCTAAAGAACGCACCCAGCAGGACAAAGCAGATGCTCCTCAGAACCAGTCTTCAACATCCTTAGTTACTTCTGAACCTGTCACCAGACCCAAGTCTCTGTCTGACCTTACAAGTCAGAAACACCGCCATACTAACCATGAACTCTATTCACTTGCTCAATCAAATCGCCAAAAGGAAAAGGAACATGCTAGGAAATGGATTTTAAGGGTGTGGGATAATGGTGGGAGGCTCACAATACTGGATCAGATTGAATTTCTCAGTTTAGGTCCTTTGAGCCTAGATAGTGAGTTTAATGTCATAGCCCGCACTGTTGCAGATAATGGTGTGAAGAGTTTGTTTGATTGGTTGGCTGAAGCATGGGTCCAGAGATGGCCTACTACAAGAGAGCTGCAGACGCCTGACGCCCTGGAGTGGTATTCTATTGAGGATGGGATTAAAAGGCTTAGGGAACTTGGAATGATAGAGTGGCTTTGTGTAAAAGCTACTTGTCCACAGTGGAGGGGCCCGGAAGATGCACCCATCACAAGAGCTATGAGGATAACTTTTGTCCGGGAAACTGTAGAGACTTGGAAGAGCTTCGTATTTAGCCTCCTCTGTATAAAGGACATAACAGTGGGGAGTGTGGCTGCTCAGTTGCATGATCTAATAGAATTAAGTTTAAAGCCAGCCGGGCGTGGTGGCGCACGCTTTTAA

>Fv1COO4 MT077293 [organism=Mus cookii]

ATGAATTTCCCACGTGCGCTTGCTGGTTTCTCGAGCTGGCTCTTCAAACCTGAACTTGCCGAGGACTCTCCGGATAATGACGAGAACACTGTTAACCCATGGCGTGAGCTGCTGCAGAAGATAAATGTGGCCGATCTCCCCGATTCATCCTTTTCGAGCGGTAAGGAACTTAATGACTCTGTGTACGATACTTTTGAACATTTCTGCAAGATTAGGGACTATGACGCAGTTGGCGAGCTGCTTCTGGCATTTCTGGATAAAGTAACAAAGGAAAGGGACCAATTCAGAGATGAAATTTCCCAGCTCCGAATGCACATAAATGATCTAAAGGCTTCTAAGTGTGTCCTGGGGGAGACTCTTCTCTCCTACCGCCACAGGATTGAAGTTGGGGAAAAACAGACTGAAGCCCTCATTGTGAGGTTAGCTGATGTGCAATCCCAGGTCATGTGTCAGCCTGCCCGGAAAGTGTCTGCAGATAAGGTGAGGGCACTGATTGGTAAAGAATGGGATCCCGTAACCTGGGATGGAGATGTGTGGGAGGACATAGATTCTGAGGGGACTGAGGAAGCAGAGTTGCCCACTGTCTTGGCCTCTCCATCCTTGTCTGAGGAAAGTGGTTATGCCTTGTCTAAAGAACGCACCCAGCAGGACAAAGCAGATGCTTCTCAGAACCAGTCTTCAACATCCTTAGTTACTTCTGAACCTGTCACCAGACCCAAGTCTCTGTCTGACCTTACAAGTCAGAAACACCGCCATACTAACCATGAACTCTATTCACTTGCTCAATCAAATCGCCAAAAGGCAAAGGAACATGCTAGGAAATGGATTTTAAGGGTGTGGGATAATGGTGGGAGGCTCACAATACTGGATCAGATTGAATTTCTCAATTTAGGTCCTTTGAGCCTTGATAGTGAGTTTAATGTCATAGCCCGAACTGTTGAAGATAATGGTGTGAAGAGTTTGTTTGATTGGTTGGCTGAAGCATGGGTCCAGAGATGGCCTACTACAAGAGAGCTGCAGACGCCTGACGCCCTGGAGTGGTATTCTATTGAGGATGGGATTAAAAGGCTTAGGGAACTTGGAATGATAGAGTGGCTTTGTGTAAAAGCTACTTGTCCACAGTGGAGGGGCCCGGAAGATGCACCCATCACAAGAGCTATGAGGATAACTTTTGTCCGGGAAACTGTAGAGACTTGGAAGAGCTTCGTATTTAGCCTCCTCTGTATAAAGGACATAACAGTGGGGAGCGTGGCTGCTCAGTTGCATGATCTAATAGAATTAAGTTTAAAGCCAGCCGGGCGTGGTGGCGCACGCTTTTAA

>Fv1COO5 MT077294 [organism=Mus cookii]

ATGAATTTCCCACGTGCGCTTGCTGGTTTCTCGAGCTGGCTCTTCAAACCTGAACTTGCCGAGGACTCTCCGGATAATGACGAGAACACTGTTAACCCATGGCGTGAGCTGCTGCAGAAGATAAATGTGGCCGATCTCCCCGATTCATCCTTTTCGAGCGGTAAGGAACTTAATGACTCTGTGTACGATACTTTTGAACATTTCTGCAAGATTAGGGACTATGACGCAGTTGGCGAGCTGCTTCTGGCATTTCTGGATAAAGTAACAAAGGAAAGGGACCAATTCAGAGATGAAATTTCCCAGCTCTGAATGCACATAAATGATCTAAAGGCTTCTAAGTGTGTCCTGGGGGAGACTCTTCTCTCCTACCGCCACAGGATTGAAGTTGGGGAAAAACAGACTGAAGCCCTCATTATGAGGTTAGCTGATGTGCAATCCCAGGTCATGTGTCAGCCTCCCCGGAAAGTGTCTGCAGATAAGGTGAGGGCACTGATTGGTAAAGAATGGGATCCCGTTACCTGGGATGGAGATGTGTGGGAGGACATAGATTCTGAGGGGACTGAGGAAGCAGAGTTGCCCACTGTCTTGGCCTCTCCATCCTTGTCTGAGGAAAGTGGTTATGCCTTGTCTAAAGAACGCACCCAGCAGGACAAAGCAGATGCTTCTCAGAACCAGTCTTCAACATCCTTAGTTACTTCTGAACCTGTCACCAGACCCAAGTCTCTGTCTGACCTTACAAGTCAGAAACACCGCCATACTAACCATGAACTCTATTCACTTGCTCAATCAAATCGCCAAAAGGCAAAGGAACATGCTAGGAAATGGATTTTAAGGGTGTGGGATAATGGTGGGAGGCTCACAATACTGGATCAGATTGAATTTCTCAGTTTAGGTCCTTTGAGCCTAGATAGTGAGTTTAATGTCATAGCCCGCACTGTTGCAGATAATGGTGTGAAGAGTTTGTTTGATTGGTTGGCTGAAGCATGGGTCCAGAGATGGCCTACTACAAGAGAGCTGCAGACGCCTGACGCCCTGGAGTGGTATTCTATTGAGGATGGGATTAAAAGGCTTAGGGAACTTGGAATGATAGAGTGGCTTTGTGTAAAAGCTACTTGTCCACAGTGGAGGGGCCCGGAAGATGCACCCATCACAAGAGCTATGAGGATAACTTTTGTCCGGGAAACTGTAGAGACTTGGAAGAGCTTCGTATTTAGCCTCCTCTGTATAAAGGACATAACAGTGGGGAGCGTGGCTGCTCAGTTGCATGATCTAATAGAATTAAGTTTAAAGCCAGCCGGGCGTGGTGGCGCACGCTTTTAA

>Fv1COO6 MT077295 [organism=Mus cookii]

ATGAATTTCCCACGTGCGCTTGCTGGTTTCTCGAGCTGGCTCTTCAAACCTGAACTTGCCGAGGACTCTCCGGATAATGACGAGAACACTGTTAACCCATGGCGTGAGCTGCTGCAGAAGATAAATGTGGCCGATCTCCCCGATTCATCCTTTTCGAGCGGTAAGGAACTTAATGACTCTGTGTACGATACTTTTGAACATTTCTGCAAGATTAGGGACTATGACGCAGTTGGCGAGCTGCTTCTGGCATTTCTGGATAAAGTAACAAAGGAAAGGGACCAATTCAGAGATGAAATTTCCCAGCTCTGAATGCACATAAGTGATCTAAAGGCTTCTAAGTGTGTCCTGGGGGAGACTCTTCTCTCCTACCGCCACAGGATTGAAGTTGGGGAAAAACAGACTGAAGCCCTCATTATGAGGTTAGCTGATGTGCAATCCCAGGTCATGTGTCAGCCTCCCCGGAAAGTGTCTGCAGATAAGGTGAGGGCACTGATTGGTAAAGAATGGGATCCCGTTACCTGGGATGGAGATGTGTGGGAGGACATAGATTCTGAGGGGACTGAGGAAGCAGAGTTGCCCACTGTCTTGGCCTCTCCATCCTTGTCTGAGGAAAGTGGTTATGCCTTGTCTAAAGAACGCACCCAGCAGGACAAAGCAGATGCTCCTCAGAACCAGTCTTCAACATCCTTAGTTACTTCTGAACCTGTCACCAGACCCAAGTCTCTGTCTGACCTTACAAGTCAGAAACACCGCCATACTAACCATGAACTCTATTCACTTGCTCAATCAAATCGCCAAAAGGAAAAGGAACATGCTAGGAAATGGATTTTAAGGGTGTGGGATAATGGTGGGAGGCTCACAATACTGGATCAGATTGAATTTCTCAGTTTAGGTCCTTTGAGCCTAGATAGTGAGTTTAATGTCATAGCCCGCACTGTTGCAGATAATGGTGTGAAGAGTTTGTTTGATTGGTTGGCTGAAGCATGGGTCCAGAGATGGCCTACTACAAGAGAGCTGCAGACGCCTGACGCCCTGGAGTGGTATTCTATTGAGGATGGGATTAAAAGGCTTAGGGAACTTGGAATGATAGAGTGGCTTTGTGTAAAAGCTACTTGTCCACAGTGGAGGGGCCCGGAAGATGCACCCATCACAAGAGCTATGAGGATAACTTTTGTCCGGGAAACTGTAGAGACTTGGAAGAGCTTCGTATTTAGCCTCCTCTGTATAAAGGACATAACAGTGGGGAGCGTGGCTGCTCAGTTGCATGATCTAATAGAATTAAGTTTAAAGCCAGCCGGGCGTGGTGGCGCACGCTTTTAA

>Fv1COO7 MT077296 [organism=Mus cookii]

ATGAATTTCCCACGTGCGCTTGCTGGTTTCTCGAGCTGGCTCTTCAAACCTGAACTTGCCGAGGACTCTCCGGATAATGACGAGAACACTGTTAACCCATGGCGTGAGCTGCTGCAGAAGATAAATGTGGCCGATCTCCCCGATTCATCCTTTTCGAGCGGTAAGGAACTTAATGACTCTGTGTACGATACTTTTGAACATTTCTGCAAGATTAGGGACTATGACGCAGTTGGCGAGCTGCTTCTGGCATTTCTGGATAAAGTAACAAAGGAAAGGGACCAATTCAGAGATGAAATTTCCCAGCTCCGAATGCACATAAATGATCTAAAGGCTTCTAAGTGTGTCCTGGGGGAGACTCTTCTCTCCTACCGCCACAGGATTGAAGTTGGGGAAAAACAGACTGAAGCCCTCATTATGAGGTTAGCTGATGTGCAATCCCAGGTCATGTGTCAGCCTCCCCGGAAAGTGTCTGCAGATAAGGTGAGGGCACTGATTGGTAAAGAATGGGATCCCGTTACCTGGGATGGAGATGTGTGGGAGGACATAGATTCTGAGGGGACTGAGGAAGCAGAGTTGCCCACTGTCTTGGCCTCTCCATCCTTGTCTGAGGAAAGTGGTTATGCCTTGTCTAAAGAACGCACCCAGCAGGACAAAGCAGATGCTCCTCAGAACCAGTCTTCAACATCCTTAGTTACTTCTGAACCTGTCACCAGACCCAAGTCTCTGTCTGACCTTACAAGTCAGAAACACCGCCATACTAACCATGAACTCTATTCACTTGCTCAATCAAATCGCCAAAAGGAAAAGGAACATGCTAGGAAATGGATTTTAAGGGTGTGGGATAATGGTGGGAGGCTCACAATACTGGATCAGATTGAATTTCTCAGTTTAGGTCCTTTGAGCCTAGATAGTGAGTTTAATGTCATAGCCCGCACTGTTGCAGATAATGGTGTGAAGAGTTTGTTTGATTGGTTGGCTGAAGCATGGGTCCAGAGATGGCCTACTACAAGAGAGCTGCAGACGCCTGACGCCCTGGAGTGGTATTCTATTGAGGATGGGATTAAAAGGCTTAGGGAACTTGGAATGATAGAGTGGCTTTGTGTAAAAGCTACTTGTCCACAGTGGAGGGGCCCGGAAGATGCACCCATCACAAGAGCTATGAGGATAACTTTTGTCCGGGAAACTGTAGAGACTTGGAAGAGCTTCGTATTTAGCCTCCTCTGTATAAAGGACATAACAGTGGGGAGCGTGGCTGCTCAGTTGCATGATCTAATAGAATTAAGTTTAAAGCCAGCCGGGCGTGGTGGCGCACGCTTTTAA

>Fv7COO1 MT077297 [organism=Mus cookii]

ATGAATTTCCCACGTGCGCTTGCTGGTTTCTCGAGCTGGCTCTTCAAACCTGAACTTGCCGAGGACTCTCCAGATAATGACGAGAACCCTGTTAACCCATGGCGTGAGCTGCTGCAGAAGATAAATGTGGCCGATCTCCCCGATTCATCCTTTTCGAGCGGTAAGGAACTTAATGACTCTGTGTACGATACTTTTGAACATTTCTGCAAGGTTAGGGACTATGACGCAGTTGGCGAGCTGCTTCTGGCATTTCTGGATAAAGTAACAAAGGAAAGGGACCAATTCAGAGATGAAATTTCCCAGCTCCGAATGCACATAAATAATCTAAAGGCTTCTAAGTGTGTCCTGGGGGAGACTCTTCTCTCCTACCGCCACAGGATTGAAGTTGGGGAAAAACAGACTGAAGCCCTCATTGTGAGGTTAGCTGATTTGCAATCCCAGGTCCTGTGTCAGCCTGCCCGGAAAGTGTCTGCAGATAAGGTGAGGGCACTGATTGGTAAAGAATGGGATCCGGTAACCTGGGATGGAGATGTGTGGGAGGACATAGATTCTGATGGGACTGAGGAAGCAGAGTTGCCCACTGTCTTGGCCTCTCCATCCTTGTCTGAGGAAAGTGGTTATGCCTTGTCAAAAGAACGCACCCAGCAGGACAAAGCAGATGCTTCTCAGAACCAGTCTTCAACATCCTTAGTTACTTCTGAACCTGTCACCAGACCCAAGTCTCTGTCTGACCTTACAAGTCAGAAACACCGCCACACTAACCATGAACTCAAATCACTTGCTCAATCAAATCGCCAACAGGCAAAGGAACATGCTAGGAAATGGATTTTAAGGGTGTGGGATAATGGTGGGAGGCTCACAATACTGGATCAGACCGAATTTCTCAGTTTAGGTCCTTTGAGCCTTGATAGTGAGTTTAATGTCATAGCCCGCACTGTTGAAGATAATGGTGTGAAGAGTTTGTTTGATTGGTTGGCTGAAGCATGGGTCCAGAGATGGCCTACTACAAGAGAGCTGCAGACGCCTGACGCCCTGGAGTGGTATTCTATTGAGGATGGGATTAAAAGGCTTAGGGAACTTGGAATGATAGAGTGGCTTTGTGTAAAAGCTACTTGTCCACAGTGGAGGGGCCCGGAAGATGCACCCATCACGAGAGCTATGAGGATAACTTTTGTCCGGGAAACTGTAGAGACTTGGAAGAGCTTCGTATTTAGCCTCCTCTGTATAAAGGACATAACAGTGGGGAGCGTGGCTGCTCAGTTGCATGATCTAACAGAATTAAGTTTAAAGCCAACAGCAGCTGGCTTGACTGCTGGTTTGGCTCCTGTGGGCTCCTTGTCGGTTCTCTCTCTCTCTCCCTGGAAACATCAAAGCAACAGTTAA

>Fv7COO2 MT077298 [organism=Mus cookii]

ATGAATTTCCCACGTGCGCTTGCTGGTTTCTCGAGCTGGCTCTTCAAACCTGAACTTGCCGAGGACTCTCCAGATAATGACGAGAACACTGTTAACCCATGGCGTGAGCTGCTGCAGAAGATAAATGTGGCCGATCTCCCCGATTCATCCTTTTCGAGCGGTAAGGAACTTAATGACTCTGTGTACGATACTTTTGAACATTTCTGCAAGATTAGGGACTATGACGCAGTTGGCGAGCTGCTTCTGGCATTTCTGGATAAAGTAACAAAGGAAAGGGACCAATTCAGAGATGAAATTTCCCAGCTCCGAATGCACATAAATGATCTAAAGGCTTCTAAGTGTGTCCTGGGGGAGACTCTTCTCTCCTACCGCCACAGGATTGAAGTTGGGGAAAAACAGACTGAAGCCCTCATTGTGAGGTTAGCTGATTTGCAATCCCAGGTCATGTGTCAGCCTGCCCAGAAAGTGTCTGCAGATAAGGTGAGGGCACTGATTGGTAAAGAATGGGATCCGGTAACCTGGGATGGAGATGTGTGGGAGGACATAGATTCTGATGGGACTGAGGAAGCAGAGTTGCCCACTGTCTTGGCCTCTCCATCTTTGTCTGAGGAAAGTGGTTATGCCTTGTCAAAAGAACGCATCTGACAAAGCAGATGCTTCTCAGAACCAGTCTTCAACATCCTTAGTTACTTCTGAACCTGTCACCAGACCCAAGTCTCTGTCTGACCTTACAAGTCAGAAACACCGCCATACTAACCATGAACTCAAATCACTTGCTCAATCAAATCGCCAACAGGCAAAGGAACATGCTAGGAAATGGATTTTAAGGGTGTGGGATAATGGTGGGAGGCTCACAATACTGGATCAGACTGAATTTCTCAGTTTAGGTCCTTTGAGCCTTGATAGTGAGTTTAATGTCATAGCCCGCACTGTTGCAGATAATGGTGTGAAGAGTTTGTTTGATTGGTTGGCTGAAGCATGGGTCCAGAGATGGCCTACTACAAGAGAGCTGCAGACGCCTGACGCCCTGGAGTGGTATTCTATTGAGGATGGGATTAAAAGGCTTAGGGAACTTGGAATGATAGAGTGGCTTTGTGTAAAAGCTACTTGTCCACAGTGGAGGGGCCCGGAAGATGCACCCATCACGAGAGCTATGAGGATAACTTTTGTCCGGGAAACTGTAGAGACTTGGAAGAGCTTCGTTTTTAGCCTCCTCTGTATAAAGGACATAACAGTGGGGAGCGTGGCTGCTCAGTTGCATGATCTAATAGAATTAAGTTTAAAGCCAACAGCAGCTGGCTTGACTGCTGGTTTGGCTCCTGTGGGCTCTGTGTCGGTTCTCTCTCTCTCTCCCTGGAAACATCAAAGCAACAGTTAA

>Fv7COO3 MT077299 [organism=Mus cookii]

ATGAATTTCCCACGTGCGCTTGCTGGTTTCTCGAGCTGGCTCTTCAAACCTGAACTTGCCGAGGACTCTCCAGATAATGACGAGAACACTGTTAACCCATGGCGTGAGCTGCTGCAGAAGATAAATGTGGCCGATCTCCCCGATTCATCCTTTTCGAGCGGTAAGGAACTTAATGACTCTGTGTACGATACTTTTGAACATTTCTGCAAGATTAGGGACTATGACGCAGTTGGCGAGCTGCTTCTGGCATTTCTGGATAAAGTAACAAAGGAAAGGGACCAATTCAGAGATGAAATTTCCCAGCTCCGAATGCACATAAATGATCTAAAGGCTTCTAAGTGTGTCCTGGGGGAGACTCTTCTCTCCTACCGCCACAGGATTGAAGTTGGGGAAAAACAGACTGAAGCCCTCATTGTGAGGTTAGCTGATTTGCAATCCCAGGTCATGTGTCAGCCTGCCCAGAAAGTGTCTGCAGATAAGGTGAGGGCACTGATTGGTAAAGAATGGGATCTGGTAACCTGGGATGGAGATGTGTGGGAGGACATAGATTCTGATGGGACTGAGGAAGCAGAGTTGCCCACTGTCTTGGCCTCTCCATCTTTGTCTGAGGAAAGTGGTTATGCCTTGTCAAAAGAACGCATCTGACAAAGCAGATGCTTCTCAGAACCAGTCTTCAACATCCTTAGTTACTTCTGAACCTGTCACCAGACCCAAGTCTCTGTCTGACCTTACAAGTCAGAAACACCGCCATACTAACCATGAACTCAAATCACTTGCTCAATCAAATCGCCAACAGGCAAAGGAACATGCTAGGAAATGGATTTTAAGGGTGTGGGATAATGGTGGGAGGCTCACAATACTGGATCAGACTGAATTTCTCAGTTTAGGTCCTTTGAGCCTTGATAGTGAGTTTAATGTCATAGCCCGCACTGTTGCAGATAATGGTGTGAAGAGTTTGTTTGATTGGTTGGCTGAAGCATGGGTCCAGAGATGGCCTACTACAAGAGAGCTGCAGACGCCTGACGCCCTGGAGTGGTATTCTATTGAGGATGGGATTAAAAGGCTTAGGGAACTTGGAATGATAGAGTGGCTTTGTGTAAAAGCTACTTGTCCACAGTGGAGGGGCCCGGAAGATGCACCCATCACGAGAGCTATGAGGATAACTTTTGTCCGGGAAACTGTAGAGACTTGGAAGAGCTTCGTTTTTAGCCTCCTCTGTATAAAGGACATAACAGTGGGGAGCGTGGCTGCTCAGTTGCATGATCTAATAGAATTAAGTTTAAAGCCAACAGCAGCTGGCTTGACTGCTGGTTTGGCTCCTGTGGGCTCTGTGTCGGTTCTCTCTCTCTCTCCCTGGAAACATCAAAGCAACAGTTAA

>Fv7COO4 MT077300 [organism=Mus cookii]

ATGAATTTCCCACGTGCGCTTGCTGGTTTCTCGAGCTGGCTCTTCAAACCTGAACTTGCCGAGGACTCTCCAGATAATGACGAGAACCCTGTTAACCCATGGCGTGAGCTGCTGCAGAAGATAAATGTGGCCGGTCTCCCCGATTCATCCTTTTCGAGCGGTAAGGAACTTAATGACTCTGTGTACGATACTTTTGAACATTTCTGCAAGATTAGGGACTATGACGCAGTTGGCGAGCTGCTTCTGGCATTTCTGGATAAAGTAACAAAGGAAAGGGACCAATTCAGAGATGAAATTTCCCAGCTCCGAATGCACATAAATGATCTAAAGGCTTCTAAGTGTGTCCTGGGGGAGACTCTTCTCTCCTACCGCCACAGGATTGAAGTTGGGGAAAAACAGACTGAAGCCCTCATTGTGAGGTTAGCTGATTTGCAATCCCAGGTCCTGTGTCAGCCTGCCCGGAAAGTGTCTGCAGATAAGGTGAGGGCACTGATTGGTAAAGAATGGGATCCGGTAACCTGGGATGGAGATGTGTGGGAGGACATAGATTCTGATGGGACTGAGGAAGCAGAGTTGCCCACTGTCTTGGCCTCTCCATCCTTGTCTGAGGAAAGTGGTTATGCCTTGTCAAAAGAACGCACCCAGCAGGACAAAGCAGATGCTTCTCAGAACCAGTCTTCAACATCCTTAGTTACTTCTGAACCTGTCACCAGACCCAAGTCTCTGTCTGACCTTACAAGTCAGAAACACCGCCACACTAACCATGAACTCAAATCACTTGCTCAATCAAATCGCCAACAGGCAAAGGAACATGCTAGGAAATGGATTTTAAGGGTGTGGGATAATGGTGGGAGGCTCACAATACTGGATCAGACCGAATTTCTCAGTTTAGGTCCTTTGAGCCTTGATAGTGAGTTTAATGTCATAGCCCGCACTGTTGAAGATAATGGTGTGAAGAGTTTGTTTGATTGGTTGGCTGAAGCATGGGTCCAGAGATGGCCTACTAAAAGAGAGCTGCAGACGCCTGACGCCCTGGAGTGGTATTCTATTGAGGATGGGATTAAAAGGCTTAGGGAACTTGGAATGATAGAGTGGCTTTGTGTAAAAGCTACTTGTCCACAGTGGAGGGGCCCCGGAAGATGCACCCATCACGAGAGCTATGAGGATAACTTTTGTCCGGGAAACTGTAGAGACTTGGAAGAGCTTCGTATTTAGCCTCCTCTGTATAAAGGACATAACAGTGGGGAGCGTGGCTGCTCAGTTGCATGATCTAACAGAATTAAGTTTAAAGCCAACAGCAGCTGGCTTGACTGCTGGTTTGGCTCCTGTGGGCTCCTTGTCGGTTCTCTTTCTCTCTCCCTGGAAACATCAAAGCAACAGTTAA

>Fv7COO5 MT077301 [organism=Mus cookii]

ATGAATTTCCCACGTGCGCTTGCTGGTTTCTCGAGCTGGCTCTTCAAACCTGAACTTGCCGAGGACTCTCCAGATAATGACGAGAACACTGTTAACCCATGGCGTGAGCTGCTGCAGAAGATAAATGTGGCCGATCTCCCCGATTCATCCTTTTCGAGCGGTAAGGAACTTAATGACTCTGTGTACGATACTTTTGAACATTTCTGCAAGATTAGGGACTATGACGCAGTTGGCGAGCTGCTTCTGGCATTTCTGGATAAAGTAACAAAGGAAAGGGACCAATTCAGAGATGAAATTTCCCAGCTCCGAATGCACATAAATGATCTAAAGGCTTCTAAGTGTGTCCTGGGGGAGACTCTTCTCTGCTACCGCCACAGGATTGAAGTTGGGGAAAAACAGACTGAAGCCCTCATTGTGAGGTTAGCTGATTTGCAATCCCAGGTCATGTGTCAGCCTGCCCAGAAAATGTCTGCAGATAAGGTGAGGGCACTGATTGGTAAAGAATGGGATCCGGTAACCTGGGATGGAGATGTGTGGGAGGACATAGATTCTGATGGGACTGAGGAAGCAGAGTTGCCCACTGTCTTGGCCTCTCCATCTTTGTCTGAGGAAAGTGGTTATGCCTTGTCAAAAGAACGCATCTGACAAAGCAGATGCTTCTCAGAACCAGTCTTCAACATCCTTAGTTACTTCTGAACCTGTCACCAGACCCAAGTCTCTGTCTGACCTTACAAGTCAGAAACACCGCCATACTAACCATGAACTCAAATCACTTGCTCAATCAAATCGCCAACAGGCAAAGGAACATGCTAGGAAATGGATTTTAAGGGTGTGGGATAATGGTGGGAGGCTCACAATACTGGATCAGACTGAATTTCTCAGTTTAGGTCCTTTGAGCCTTGATAGTGAGTTTAATGTCATAGCCCGCACTGTTGCAGATAATGGTGTGAAGAGTTTGTTTGATTGGTTGGCTGAAGCATGGGTCCAGAGATGGCCTACTAAAAGAGAGCTGCAGACGCCTGACGCCCTGGAGTGGTATTCTATTGAGGATGGGATTAAAAGGCTTAGGGAACTTGGAATGATAGAGTGGCTTTGTGTAAAAGCTACTTGTCCACAGTGGAGGGGCCCGGAAGATGCACCCATCACGAGAGCTATGAGGATAACTTTTGTCCGGGAAACTGTAGAGACTTGGAAGAGCTTCGTTTTTAGCCTCCTCTGTATAAAGGACATAACAGTGGGGAGCGTGGCTGCTCAGTTGCATGATCTAATAGAATTAAGTTTAAAGCCAACAGCAGCTGGCTTGACTGCTGGTTTGGCTCCTGTGGGCTCTGTGTCGGTTCTCTCTCTCTCTCCCTGGAAACATCAAAGCAACAGTTAA

>Fv7COO6 MT077302 [organism=Mus cookii]

ATGAATTTCCCACGTGCGCTTGCTGGTTTCTCGAGCTGGCTCTTCAAACCTGAACTTGCCGAGGACTCTCCAGATAATGACGAGAACACTGTTAACCCATGGCGTGAGCTGCTGCAGAAGATAAATGTGGCCGATCTCCCCGATTCATCCTTTTCGAGCGGTAAGGAACTTAATGACTCTGTGTACGATACTTTTGAACATTTCTGCAAGATTAGGGACTATGACGCAGTTGGCGAGCTGCTTCTGGCATTTCTGGATAAAGTAACAAAGGAAAGGGACCAATTCAGAGATGAAATTTCCCAGCTCCGAATGCACATAAATGATCTAAAGGCTTCTAAGTGTGTCCTGGGGGAGACTCTTCTCTCCTACCGCCACAGGATTGAAGTTGGGGAAAAACAGACTGAAGCCCTCATTGTGAGGTTAGCTGATTTGCAATCCCAGGTCATGTGTCAGCCTGCCCAGAAAGTGTCTGCAGATAAGGTGAGGGCACTGATTGGTAAAGAATGGGATCCGGTAACCTGGGATGGAGATGTGTGGGAGGACATAGATTCTGATGGGACTGAGGAAGCAGAGTTGCCCACTGTCTTGGCCTCTCCATCTTTGTCTGAGGAAAGTGGTTATGCCTTGTCAAAAGAACGCATCTGACAAAGCAGATGCTTCTCAGAACCAGTCTTCAACATCCTTAGTTACTTCTGAACCTGTCACCAGACCCAAGTCTCTGTCTGACCTTACAAGTCAGAAACACCGCCATACTAACCATGAACTCAAATCACTTGCTCAATCAAATCGCCAACAGGCAAAGGAACATGCTAGGAAATGGATTTTAAGGGTGTGGGATAATGGTGGGAGGCTCACAATACTGGATCAGACTGAATTTCTCAGTTTAGGTCCTTTGAGCCTTGATAGTGAGTTTAATGTCATAGCCCGCACTGTTGCAGATAATGGTGTGAAGAGTTTGTTTGATTGGTTGGCTGAAGCATGGGTCCAGAGATGGCCTACTACAAGAGAGCTGCAGACGCCTGATGCCCTGGAGTGGTATTCTATTGAGGATGGGATTAAAAGGCTTAGGGAACTTGGAATGATAGAGTGGCTTTGTGTAAAAGCTACTTGTCCACAGTGGAGGGGCCCGGAAGATGCACCCATCACGAGAGCTATGAGGATAACTTTTGTCCGGGAAACTGTAGAGACTTGGAAGAGCTTCGTTTTTAGCCTCCTCTGTATAAAGGACATAACAGTGGGGAGCGTGGCTGCTCAGTTGCATGATCTAATAGAATTAAGTTTAAAGCCAACAGCAGCTGGCTTGACTGCTGGTTTGGCTCCTGTGGGCTCTGTGTCGGTTCTCTCTCTCTCTCCCTGGAAACATCAAAGCAACAGTTAA

>Fv7COO7 MT077303 [organism=Mus cookii]

ATGAATTTCCCACGTGCGCTTGCTGGTTTCTCGAGCTGGCTCTTCAAACCTGAACTTGCCGAGGACTCTCCAGATAATGACGAGAACCCTGTTAACCCATGGCGTGAGCTGCTGCAGAAGATAAATGTGGCCGGTCTCCCCGATTCATCCTTTTCGAGCGGTAAGGAACTTAATGACTCTGTGTACGATACTTTTGAACATTTCTGCAAGATTAGGGACTATGACGCAGTTGGCGAGCTGCTTCTGGCATTTCTGGATAAAGTAACAAAGGAAAGGGACCAATTCAGAGATGAAATTTCCCAGCTCCGAATGCACATAAATGATCTAAAGGCTTCTAAGTGTGTCCTGGGGGAGACTCTTCTCTCCTACCGCCACAGGATTGAAGTTGGGGAAAAACAGACTGAAGCCCTCATTGTGAGGTTAGCTGATTTGCAATCCCAGGTCCTGTGTCAGCCTGCCCGGAAAGTGTCTGCAGATAAGGTGAGGGCACTGATTGGTAAAGAATGGGATCCGGTAACCTGGGATGGAGATGTGTGGGAGGACATAGATTCTGATGGGACTGAGGAAGCAGAGTTGCCCACTGTCTTGGCCTCTCCATCTTTGTCTGAGGAAAGTGGTTATGCCTTGTCAAAAGAACGCATCTGACAAAGCAGATGCTTCTCAGAACCAGTCTTCAACATCCTTAGTTACTTCTGAACCTGTCACCAGACCCAAGTCTCTGTCTGACCTTACAAGTCAGAAACACCGCCATACTAACCATGAACTCAAATCACTTGCTCAATCAAATCGCCAACAGGCAAAGGAACATGCTAGGAAATGGATTTTAAGGGTGTGGGATAATGGTGGGAGGCTCACAATACTGGATCAGACTGAATTTCTCAGTTTAGGTCCTTTGAGCCTTGATAGTGAGTTTAATGTCATAGCCCGCACTGTTGCAGATAATGGTGTGAAGAGTTTGTTTGATTGGTTGGCTGAAGCATGGGTCCAGAGATGGCCTACTACAAGAGAGCTGCAGACGCCTGACGCCCTGGAGTGGTATTCTATTGAGGATGGGATTAAAAGGCTTAGGGAACTTGGAATGATAGAGTGGCTTTGTGTAAAAGCTACTTGTCCACAGTGGAGGGGCCCGGAAGATGCACCCATCACGAGAGCTATGAGGATAACTTTTGTCCGGGAAACTGTAGAGACTTGGAAGAGCTTCGTTTTTAGCCTCCTCTGTATAAAGGACATAACAGTGGGGAGCGTGGCTGCTCAGTTGCATGATCTAATAGAATTAAGTTTAAAGCCAACAGCAGCTGGCTTGACTGCTGGTTTGGCTCCTGTGGGCTCTGTGTCGGTTCTCTCTCTCTCTCCCTGGAAACATCAAAGCAACAGTTAA

>Fv7COO8 MT077304 [organism=Mus cookii]

ATGAATTTCCCACGTGCGCTTGCTGGTTTCTCGAGCTGGCTCTTCAAACATGAACTTGCCGAGGACTCTCCAGATAATGACGAGAACCCTGTTAACCCATGGCGTGAGCTGCTGCAGAAGATAAATGTGGCCGATCTCCCCGATTCATCCTTTTCGAGCGGTAAGGAACTTAATGACTCTGTGTACGATACTTTTGAACATTTCTGCAAGGTTAGGGACTATGACGCAGTTGGCGAGCTGCTTCTGGCATTTCTGGATAAAGTAACAAAGGAAAGGGACCAATTCAGAGATGAAATTTCCCAGCTCCGAATGCACATAAATGATCTAAAGGCTTCTAAGTGTGTCCTGGGGGAGACTCTTCTCTCCTACCGCCACAGGATTGAAGTTGGGGAAAAACAGACTGAAGCCCTCATTGTGAGGTTAGCTGATTTGCAATCCCAGGTCCTGTGTCAGCCTGCCCGGAAAGTGTCTGCAGATAAGGTGAGGGCACTGATTGGTAAAGAATGGGATCCGGTAACCTGGGATGGAGATGTGTGGGAGGACATAGATTCTGATGGGACTGAGGAAGCAGAGTTGCCCACTGTCTTGGCCTCTCCATCCTTGTCTGAGGAAAGTGGTTATGCCTTGTCAAAAGAACGCACCCAGCAGGACAAAGCAGATGCTTCTCAGAACCAGTCTTCAACATCCTTAGTTACTTCTGAACCTGTCACCAGACCCAAGTCTCTGTCTGACCTTACAAGTCAGAAACACCGCCACACTAACCATGAACTCAAATCACTTGCTCAATCAAATCGCCAACAGGCAAAGGAACATGCTAGGAAATGGATTTTAAGGGTGTGGGATAATGGTGGGAGGCTCACAATACTGGATCAGACCGAATTTCTCAGTTTAGGTCCTTTGAGCCTTGATAGTGAGTTTAATGTCATAGCCCGCACTGTTGAAGATAATGGTGTGAAGAGTTTGTTTGATTGGTTGGCTGAAGCATGGGTCCAGAGATGGCCTACTACAAGAGAGCTGCAGACGCCTGACGCCCTGGAGTGGTATTCTATTGAGGATGGGATTAAAAGGCTTAGGGAACTTGGAATGATAGAGTGGCTTTGTGTAAAAGCTACTTGTCCACAGTGGAGGGGCCCGGAAGATGCACCCATCACGAGAGCTATGAGGATAACTTTTGTCCGGGAAACTGTAGAGACTTGGAAGAGCTTCGTATTTAGCCTCCTCTGTATAAAGGACATAACAGTGGGGAGCGTGGCTGCTCAGTTGCATGATCTAACAGAATTAAGTTTAAAGCCAACAGCAGCTGGCTTGACTGCTGGTTTGGCTCCTGTGGGCTCCTTGTCGGTTCTCTCTCTCTCTCCCTGGAAACATCAAAGCAACAGTTAA

>Fv1FRA1 MT077305 [organism=Mus fragilicauda]

ATGAATTTCCCACGTGCGCTTGCTGGTTTCTCCAGCTGGCTCTTCAAACCTGAACTTGCCGAGGACTCTCCGGATAATGACGAGAACACTGTTAACCCATGGCGTGAGCTGCTGCAGAAGATAAATGTGGCCGATCTCCCCGATTCATCCTTTTCGAGCGGTAAGGAACTTAATGACTCTGTGTACGATACTTTTGAACATTTTTGCAAGATTAGGGACTATGACGCAGTTGGCGAGCTGCTTCTGGCATTTCTGGATAAAGTAACAAAGGAAAGGGACCAATTCAGAGATGAAATTTCCCAGCTCCGAATGCACATAAATGATCTAAAGGCTTCTAAGTGTGTCCTGGGGGAGACTCTTCTCTCTTACCGCCACAGGATTGAAGTTGGGGAAAAACAGACTGAAGCCCTCATTGTGAGGTTAGCTGATTTGCAATCCCAGGTCATGTGTCAGCCTGCCCGGAAAGTGTCTGCAGATAAGGTGAGGGCACTGATTGGTAAAGAATGGGATCCCGTAACCTGGGATGGAGATGTGTGGGAGGACATAGATTCTGATGGGACTGAGGACGCAGAGTTGCCCACTGTCTTGGCCTCTCCATCCTTGTCTGAGGAAAGTGGTTATGCCTCGTCTAAAGAACGCACCCAGCAGGACAAAGCAGATGCTCCTCAGAACCAGTCTTCAACATCCTTAGTTACTTCTGAACCTGTCACCAGACCCAAGTCTCTGTCTGACCTTACAAGTCAGAAACACCGCCATACTAACCATGAACTCAATTTACTTGCTCACTCAAATTGCCAACAGGCAAAGGAACATGCTAGGAAATGGATTTTAAGGGTGTGGGATAATGGTGGGAGGCTCACGATACTGGATCAGATTGAATTTCTCAGTTTAGGTCCTTTGAGCCTTGATAGTGAGTTTAATGTCATAGCCCGCACTGTTGAAGATAATGGTGTGAAGAGTTTGTTTGATTGGTTGGCTGAAGCATGGGTCCAGAGATGGCCTACTACAAGAGAGCTGCAGACGCCTGACACCCTGGAGTGGTATTCTATTGAGGATGGGATTAAAAGGCTTAGGGAACTTGGAATGATAGAGTGGCTTTGTGTAAAAGCTACTTGTCCACAGTGGAGGGGCCCGGAAGATGTACCCATCACGAGAGCTATGAGGATAACTTTTGTCCGGGAAACTGTAGAGACTTGGAAGAGCTTTGTATTTAGCCTCCTCTGTATAAAGGACATAACAGTGGGGAGCGTGGCTGCTCAGTTGCATGATCTAATAGAATTACGTTTAAATCCAAAGCCAGCCGGGCGTGGTGGCGCACACTTTTAA

>Fv1FRA2 MT077306 [organism=Mus fragilicauda]

ATGAATTTCCCACGTGCGCTTGCTGGTTTCTCCAGCTGGCTCTTCAAACCTGAACTTGCCGAGGACTCTCCGGATAATGACGAGAACACTGTTAACCCATGGCGTGAGCTGCTGCAGAAGATAAATGTGGCCGATCTCCCCGATTCATCCTTTTCGAGCGGTAAGGAACTTAATGACTCTGTGTACGATACTTTTGAACATTTCTGCAAGATTAGGGACTATGACGCAGTTGGCGAGCTGCTTCTGGCATTTCTGGATAAAGTAACAAAGGAAAGGGACCAATTCAGAGATGAAATTTCCCAGCTCCGAATGCACATAAATGATCTAAAGGCTTCTAAGTGTGTCCTGGGGGAGACTCTTCTCTCTTACCGCCACAGGATTGAAGTTGGGGAAAAACAGACTGAAGCCCTCATTGTGAGGTTAGCTGATTTGCAATCCCAGGTCATGTGTCAGCCTGTCCGGAAAGTGTCTGCAGATAAGGTGAGGGCACTGATTGGTAAAGAATGGGATCCCGTAACCTGGGATGGAGATGTGTGGGAGGACATAGATTCTGATGGGACTGAGGACGCAGAGTTGCCCACTGTCTTGGCCTCTCCATCCTTGTCTGAGGAAAGTGGTTATGCCTCGTCTAAAGAACGCACCCAGCAGGACAAAGCAGATGCTCCTCAGAACCAGTCTTCAACATCCTTAGTTACTTCTGAACCTGTCACCAGACCCAAGTCTCTGTCTGACCTTACAAGTCAGAAACACCGCCATACTAACCATGAACTCAATTTACTTGCTCACTCAAATCACCAACAGGCAAAGGAACATGCTAGGAAATGGATTTTAAGGGTGTGGGATAATGGTGGGAGGCTCACGATACTGGATCAGATTGAATTTCTCAGTTTAGGTCCTTTGAGCCTTGATAGTGAGTTTAATGTCATAGCCCGCACTGTTGAAGATAATGGTGTGAAGAGTTTGTTTGATTGGTTGGCTGAAGCATGGGTCCAGAGATGGCCTACTACAAGAGAGCTGCAGACGCCTGACACCCTGGAGTGGTATTCTATTGAGGATGGGATTAAAAGGCTTAGGGAACTTGGAATGATAGAGTGGCTTTGTGTAAAAGCTACTTGTCCACAGTGGAGGGGCCCGGAAGATGTACCCATCACGAGAGCTATGAGGATAACTTTTGTCCGGGAAACTGTAGAGACTTGGAAGAGCTTTGTATTTAGCCTCCTCTGTATAAAGGACATAACAGTGGGGAGCGTGGCTGCTCAGTTGCATGATCTAATAGAATTACGTTTAAATCCAAAGCCAGCCGGGCGTGGTGGCGCACACTTTTAA

>Fv1FRA3 MT077307 [organism=Mus fragilicauda]

ATGAATTTCCCACGTGCGCTTGCTGGTTTCTCCAGCTGGCTCTTCAAACCTGAACTTGCCGAGGACTCTCCGGATAATGACGAGAACACTGTTAACCCATGGCGTGAGCTGCTGCAGAAGATAAATGTGGCCGATCTCCCCGATTCATCCTTTTCGAGCGGTAAGGAACTTAATGACTCTGTGTACGATACTTTTGAACATTTCTGCAAGATTAGGGACTATGACGCAGTTGGCGAGCTGCTTCTGGCATTTCTGGATAAAGTAACAAAGGAAAGGGACCAATTCAGAGATGAAATTTCCCAGCTCCGAATGCACATAAATGATCTAAAGGCTTCTAAGTGTGTCCTGGGGGAGACTCTTCTCTCTTACCGCCACAGGATTGAAGTTGGGGAAAAACAGACTGAAGCCCTCATTGTGAGGTTAGCTGATTTGCAATCCCAGGTCATGTGTCAGCCTGCCCGGAAAGTGTCTGCAGATAAGGTGAGGGCACTGATTGGTAAAGAATGGGATCCCGTAACCTGGGATGGAGATGTGTGGGAGGACATAGATTCTGATGGGACTGAGGACGCAGAGTTGCCCACTGTCTTGGCCTCTCCATCCTTGTCTGAGGAAAGTGGTTATGCCTCGTCTAAAGAACGCACCCAGCAGGACAAAGCAGATGCTCCTCAGAACCAGTCTTCAACATCCTTAGTTACTTCTGAACCTGTCACCAGACCCAAGTCTCTGTCTGACCTTACAAGTCAGAAACACCGCCATACTAACCATGAACTCAATTTACTTGCTCACTCAAATCGCCAACAGGCAAAGGAACATGCTAGGAAATGGATTTTAAGGGTGTGGGATAATGGTGGGAGGCTCACGATACTGGATCAGATTGAATTTCTCAGTTTAGGTCCTTTGAGCCTTGATAGTGAGTTTAATGTCATAGCCCGCACTGTTGAAGATAATGGTGTGAAGAGTTTGTTTGATTGGTTGGCTGAAGCATGGGTCCAGAGATGGCCTACTACAAGAGAGCTGCAGACGCCTGACACCCTGGAGTGGTATTCTATTGAGGATGGGATTAAAAGGCTTAGGGAACTTGGAATGATAGAGTGGCTTTGTGTAAAAGCTACTTGTCCACAGTGGAGGGGCCCGGAAGATGTACCCATCACGAGAGCTATGAGGATAACTTTTGTCCGGGAAACTGTAGAGACTTGGAAGAGCTTTGTATTTAGCCTCCTCTGTATAAAGGACATAACAGTGGGGAGCGTGGCTGCTCAGTTGCATGATCTAATAGAATTACGTTTAAATCCAAAGCCAGCCGGGCGTGGTGGCGCACACTTTTAA

>Fv1FRA4 MT077308 [organism=Mus fragilicauda]

ATGAATTTCCCACGTGCGCTTGCTGGTTTCTCCAGCTGGCTCTTCAAACCTGAACTTGCCGAGGACTCTCCGGATAATGACGAGAACACTGTTAACCCATGGCGTGAGCTGCTGCAGAAGATAAATGTGGCCGATCTCCCCGATTCATCCTTTTCGAGCGGTAAGGAACTTAATGACTCTGTGTACGATACTTTTGAACATTTCTGCAAGATTAGGGACTATGACGCAGTTGGCGAGCTGCTTCTGGCATTTCTGGATAAAGTAACAAAGGAAAGGGACCAATTCAGAGATGAAATTTCCCAGCTCCGAATGCACATAAATGATCTAAAGGCTTCTAAGTGTGTCCTGGGGGAGACTCTTCTCTCTTACCGCCACAGGATTGAAGTTGGGGAAAAACAGACTGAAGCCCTCATTGTGAGGTTAGCTGATTTGCAATCCCAGGTCATGTGTCAGCCTGCCCGGAAAGTGTCTGCAGATAAGGTGAGGGCACTGATTGGTAAAGAATGGGATCCCGTAACCTGGGATGGAGATGTGTGGGAGGACATAGATTCTGATGGGACTGAGGACGCAGAGTTGCCCACTGTCTTGGCCTCTCCATCCTTGTCTGAGGAAAGTGGTTATGCCTCGTCTAAAGAACGCACCCAGCAGGACAAAGCAGATGCTCCTCAGAACCAGTCTTCAACATCCTTAGTTACTTCTGAACCTGTCACCAGACCCAAGTCTCTGTCTGACCTTACAAGTCAGAAACACCGCCATACTAACCATGAACTCAATTTACTTGCTCACTCAAATTGCCAACAGGCAAAGGAACATGCTAGGAAATGGATTTTAAGGGTGTGGGATAATGGTGGGAGGCTCACGATACTGGATCAGATTGAATTTCTCAGTTTAGGTCCTTTGAGCCTTGATAGTGAGTTTAATGTCATAGCCCGCACTGTTGAAGATAATGGTGTGAAGAGTTTGTTTGATTGGTTGGCTGAAGCATGGGTCCAGAGATGGCCTACTACAAGAGAGCTGCAGACGCCTGACACCCTGGAGTGGTATTCTATTGAGGATGGGATTAAAAGGCTTAGGGAACTTGGAATGATAGAGTGGCTTTGTGTAAAAGCTACTTGTCCACAGTGGAGGGGCCCGGAAGATGTACCCATCACGAGAGCTATGAGGATAACTTTTGTCCGGGAAACTGTAGAGACTTGGAAGAGCTTTGTATTTAGCCTCCTCTGTATAAAGGACATAACAGTGGGGAGCGTGGCTGCTCAGTTGCATGATCTAAAAGAATTACGTTTAAATCCAAAGCCAGCCGGGCGTGGTGGCGCACACTTTTAA
